# Selective sweeps on novel and introgressed variation shape mimicry loci in a butterfly adaptive radiation

**DOI:** 10.1371/journal.pbio.3000597

**Published:** 2020-02-06

**Authors:** Markus Moest, Steven M. Van Belleghem, Jennifer E. James, Camilo Salazar, Simon H. Martin, Sarah L. Barker, Gilson R. P. Moreira, Claire Mérot, Mathieu Joron, Nicola J. Nadeau, Florian M. Steiner, Chris D. Jiggins

**Affiliations:** 1 Department of Zoology, University of Cambridge, Cambridge, United Kingdom; 2 Department of Ecology, University of Innsbruck, Innsbruck, Austria; 3 Department of Biology, University of Puerto Rico, Rio Piedras, Puerto Rico; 4 Department of Ecology and Evolutionary Biology, University of Arizona, Tucson, Arizona, United States of America; 5 Biology Program, Faculty of Natural Sciences and Mathematics, Universidad del Rosario, Bogota D.C., Colombia; 6 Institute of Evolutionary Biology, University of Edinburgh, Edinburgh, United Kingdom; 7 Departamento de Zoologia, Universidade Federal do Rio Grande do Sul, Porto Alegre, Brazil; 8 IBIS, Department of Biology, Université Laval, Québec, Canada; 9 Centre d'Ecologie Fonctionnelle et Evolutive, UMR 5175 CNRS—Université de Montpellier—Université Paul Valéry Montpellier—EPHE, Montpellier, France; 10 Department of Animal and Plant Sciences, University of Sheffield, Sheffield, United Kingdom; University of Notre Dame, UNITED STATES

## Abstract

Natural selection leaves distinct signatures in the genome that can reveal the targets and history of adaptive evolution. By analysing high-coverage genome sequence data from 4 major colour pattern loci sampled from nearly 600 individuals in 53 populations, we show pervasive selection on wing patterns in the *Heliconius* adaptive radiation. The strongest signatures correspond to loci with the greatest phenotypic effects, consistent with visual selection by predators, and are found in colour patterns with geographically restricted distributions. These recent sweeps are similar between co-mimics and indicate colour pattern turn-over events despite strong stabilising selection. Using simulations, we compare sweep signatures expected under classic hard sweeps with those resulting from adaptive introgression, an important aspect of mimicry evolution in *Heliconius* butterflies. Simulated recipient populations show a distinct ‘volcano’ pattern with peaks of increased genetic diversity around the selected target, characteristic of sweeps of introgressed variation and consistent with diversity patterns found in some populations. Our genomic data reveal a surprisingly dynamic history of colour pattern selection and co-evolution in this adaptive radiation.

## Abstract 2

Selection scans across multiple populations and species of Heliconius butterfly reveal pervasive selection on novel and introgressed color pattern variation, suggesting high rates of color pattern turn-over events, despite strong stabilizing Muellerian mimicry selection and cases of color pattern co-evolution in this radiation.

## Introduction

Identifying targets of selection and reconstructing their evolutionary history is central to understanding how populations adapt [1–3]. In particular, genome sequences contain a rich source of information about past events in natural populations. The action of recent positive selection can leave a distinct signature known as a ‘selective sweep’, which provides information on the genomic location of targets of positive selection and the timing and strength of selection [4,5]. Although many classic examples of selective sweeps have been found in domesticated populations, such as maize [6], chicken [7], and cattle [8], or in humans [9], increasingly natural populations are also studied. Using genomic data, these latter studies can reveal the genetic architecture and evolutionary history of ecologically relevant traits [10–13] and provide insights into the action of natural selection by complementing field and experimental studies [14–16]. However, to date, few molecular studies of natural populations have used broad sampling in adaptive radiations with varying selection pressures and sources of adaptive variation for the same trait. Such studies will allow the investigation of both complexity and general mechanisms of natural selection in the wild at the genotypic level, especially where there is a priori information on the agents and targets of selection.

Positive selection can rapidly change allele frequencies leaving detectable signatures in a genome. These signals can be traced over ecological and evolutionary time scales, during which they are gradually eroded by new mutations and recombination [1]. However, the observed patterns will depend on the sources and frequency of genetic variation upon which selection acts [5]. For example, a classic ‘hard sweep’ due to selection on a single, novel beneficial mutation [4] or a very rare allele from standing variation [17], is distinct from a ‘soft sweep’ due to selection on standing variation already present at an appreciable frequency [17–20] or recurrent mutations [21,22]. Less well studied in the context of selective sweeps is the possibility that a new variant is introduced by gene flow from a related population or distinct species. Accumulating evidence suggests that this reuse of ancient variants is far more common than was previously envisioned [23–26]. However, the sweep signatures created by selection on introgressed and divergent haplotypes, and the effect of migration rate on these signatures, are largely unexplored (but see Setter and colleagues [27]).

Mimicry systems provide some of the best examples of natural selection and adaptation and thus exceptional opportunities to study selective sweeps. In the unpalatable *Heliconius* butterflies, mimicry of wing patterns is advantageous because resemblance to a common, well-protected pattern confers protection from predator attacks on individuals. The vast majority of pattern diversity seen in this group is controlled by a surprisingly simple genetic system, involving allelic variation at just 4 major effect loci, although additional regulators and modifiers of these mimicry patterns have also been mapped [26,28–34]. Although these regions comprise several genes with a putative function for colour patterning, current evidence suggests a major role for the transcription factors, *optix* [35] and *aristaless*, which comes in 2 tandem copies *al1* and *al2* [28], a signalling ligand, *WntA* [29], and a gene in a family of cell cycle regulators whose exact function remains unclear, *cortex* [30]. We therefore refer to these 4 regions by the name of the respective major colour pattern gene throughout the manuscript without excluding the potential involvement of additional genes within these regions. A complex series of regulatory variants at each of these loci is found in different combinations across populations and species, leading to great diversity of wing patterns. In many cases, candidate noncoding, *cis*-regulatory elements (CREs) are associated with specific wing patterns: CREs in the *optix* region are associated with the red forewing band, hindwing rays, and dennis patch [36–38]; in the *cortex* region with the yellow hindwing bar [30,38,39]; in the *WntA* region with various shape elements of the forewing band [33,38]; and in the *aristaless* region with white versus yellow colour variation [28].

Colour pattern novelty is generated by mutation, introgression, shuffling, and epistatic interaction of existing CREs that generate new pattern combinations [36,38–41]. In fact, adaptive sharing of mimicry colour patterns has been demonstrated across many species and populations within the *H*. *melpomene* and *H*. *erato* clade [36,38,39,42–46]. The *H*. *melpomene* clade comprises the sister clades *H*. *melpomene* and *H*. *cydno/heurippa/timareta*, which split 1 to 1.5 million years ago (Mya) [47–49] and their outgroup silvaniform clade (4 Mya since divergence) [50]. Well-characterised cases of adaptive introgression in this clade include the exchange of red and yellow elements among *H*. *melpomene*, *H*. *timareta*, and the silvaniforms *H*. *elevatus* and *H*. *besckei* [36,44,45], as well as the sharing of elements controlling yellow hindwing colouration between *H*. *melpomene* and *H*. *cydno* [39]. Consequently, we can assess patterns of selection in well-defined genomic intervals with evidence for dated introgression events [36,39]. Likewise, hybridisation is also important within the *Heliconius erato* clade [46,51,52], but there is no evidence for gene flow between these 2 major clades that split around 12 Mya [50]. *Heliconius erato* comprises several colour pattern races that are co-mimics with *H*. *melpomene*, *H*. *timareta*, *H*. *besckei*, and *H*. *elevatus* and is often the more abundant co-mimic [53].

*Heliconius* colour patterns are known to be subject to remarkably strong natural selection in wild populations, which has been demonstrated through pattern manipulations [54], reciprocal transplants across a hybrid zone [55], reciprocal transfers between different co-mimic communities [56], and artificial models [57,58]. In all cases, estimates of selection strength were high with *s* = 0.52–0.64 (Table 1). Indirect estimates of selection strength from hybrid zones generated similarly high values with *s* = 0.23 for each of 3 colour pattern loci containing *optix*, *cortex*, and *WntA*, in *H*. *erato* and *s* = 0.25 for *optix* and *cortex* in *H*. *melpomene* [59–63] but also include cases of substantial variance in selection coefficients [64] (see Table 1 for details).

**Table 1:** Direct and indirect estimates of selection on colour pattern loci. Combined estimates are integrating the effect of all loci involved in warning colouration. Regions/modules associated with *optix*: D, B; with *cortex*: Cr, Yb, N; with *WntA*: Sd, Ac; with *aristaless*: K.

Species	Colour pattern region under consideration	Estimated selection coefficient (*s*)	Method	Source
*H*. *erato*	*optix* (red band)	*s*_*D*_ = 0.22	Pattern manipulation, survival and bird attack rate	Benson [54] (*s* estimate calculated in Mallet and colleagues [65])
*H*. *erato*	*optix/cortex/WntA*	combined *s* = 0.52 avg. per locus *s* = 0.17	Reciprocal transplants, survival	Mallet and Barton [55]
*H*. *erato*	*optix/cortex/WntA*	*s*_*D*_ = 0.33 *s*_*Cr*_ = 0.15 *s*_*Sd*_ = 0.15	Reciprocal transplants, survival	Mallet and colleagues[65]
*H*. *erato* *H*. *melpomene*	*optix/cortex/WntA* *optix/cortex*	avg. per locus *s* = 0.23 avg. per locus *s* = 0.25	Cline and LD analysis in a hybrid zone	Mallet and colleagues[61]
*H*. *erato*	*cortex*	*s*_*Cr*_ = 0.20–0.22	Cline analysis in a hybrid zone	Blum [66]
*H*. *cydno* (polymorphic mimic) *H*. *sapho* (model) *H*. *eleuchia* (model)	*aristaless*	*s* = 0.64	Reciprocal transplant of polymorphic *H*. *cydno*	Kapan [56]
*H*. *erato*	*optix/cortex/WntA*	avg. per locus *s* = 0.22 *s*_*D*_ = 0.38 *s*_*Cr*_ = 0.17 *s*_*Sd*_ = 0.15	Cline and LD analysis in a hybrid zone	Rosser and colleagues[62]
*H*. *melpomene*	*optix/cortex*	avg. per locus s = 0.3 *s*_*D*_ = *s*_*Yb*_ = *s*_*N*_ = 0.31 *s*_*B*_ = 0.19/0.15		
*H*. *erato*	*optix/WntA*	*s*_*D*_ = 0.15 *s*_*Sd*_ = 0.04	Cline analysis in a hybrid zone	Salazar [63]
*H*. *melpomene*	*optix/WntA*	*s*_*D*_ = 0.27 *s*_*Ac*_ = 0.04		
*H*. *erato*	*cortex*	*s*_*Cr*_ = 0.05	Cline analysis in a hybrid zone	Thurman and colleagues [64]

**Abbreviations:** LD, linkage disequilibrium

Although colour pattern loci in *Heliconius* are well studied, and their adaptive significance is apparent, the impact of selection at the molecular level has never been estimated in detail in natural *Heliconius* populations. Genetic studies have shown that populations often cluster by phenotype rather than geography at colour pattern loci [38,67,68], but these approaches may not detect recent adaptive changes. For example, closely related populations show peaks of high differentiation at colour pattern loci [34,69], but previous studies did not reveal strong sweep signatures [31,32,70], and more recent genomic analysis showed only weak evidence for reduced heterozygosity and enhanced linkage disequilibrium [68]. However, these studies have used either few amplicons or genomic data with small sample sizes and therefore potentially had little power to detect selective sweep signatures.

Here, we obtain a large genomic data set across the *H*. *melpomene* radiation, featuring both high coverage and large sample size, and combine simulations with population genomic analysis to investigate natural selection at 4 main colour pattern loci. We use forward-in-time simulations to compare the signal produced by classic and introgressed sweeps in genome scan data, to characterise expected patterns for introgressed sweeps under varying effective migration rate and strength of selection, patterns which have previously been little explored [27]. We parameterise our simulations with demographic estimates representative for *Heliconius* in order to inform inferences about the timing of sweeps detected in *Heliconius* populations. Our empirical data set covers almost the entire biogeographic range of an adaptive radiation and demonstrates clear signatures of selective sweeps across many populations. However, many widespread colour patterns show only modest signals of sweeps, with the strongest signals found in populations with geographically restricted patterns, suggesting recent and strong selection. For adaptive introgression, our simulations demonstrate that the signals have distinct shapes, are strongly affected by effective migration rates, and are more challenging to detect. Nevertheless, we identify sweep signatures among populations with known colour pattern introgression. Moreover, we identify new putative targets of selection around colour pattern genes in some populations. Finally, we also analyse genomic data from *H*. *erato* populations, representing a distinct radiation of similar wing pattern forms, and find evidence for parallel evolution between co-mimetic butterfly species.

## Results

### Phylogeography and demography of the *H*. *melpomene* clade

**Fig 1:**
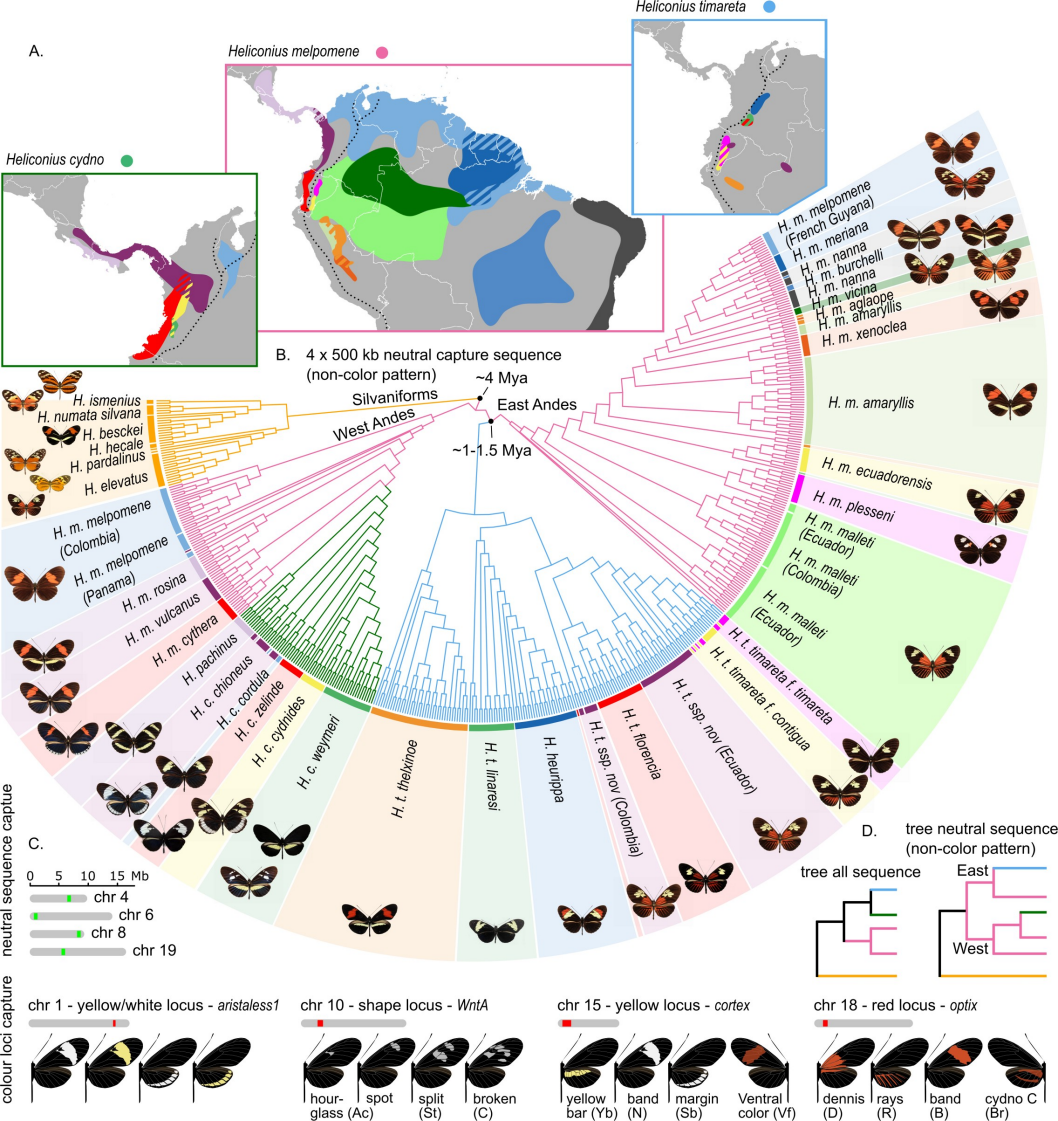
Distribution, phylogenetic relations, major colour pattern loci, and sequence capture targets of the *H*. *melpomene*, *H*. *cydno*, and *H*. *timareta* clade species. (A) Broad distributions of the *H*. *melpomene*, *H*. *cydno*, and *H*. *timareta* colour pattern races and species (based on all known sampling localities; for details, see S2 Fig). Distribution colours match the shadings around the phylogeny and butterfly images in panel B. The dashed line indicates the Andes. Note the distinct clusters formed by individuals sampled from the *H*. *m*. *vicina* population. The cluster grouping with *H*. *timareta* is referred to as *H*. *timareta* ssp. *nov*. (Colombia) (B) FastTree cladogram inferred using capture sequence from putatively neutral loci. Colours in the tree indicate the *H*. *melpomene* (pink), *H*. *cydno* (green), and *H*. *timareta* (blue) clades and match the boxes of the distribution maps in panel A. (C) Sequence information was obtained for 4 putatively neutral regions (green) and 4 regions to which functional variation has been mapped to a yellow/white colour switch (chr 1); forewing band shape (chr 10); yellow/white fore- and hindwing bars, band margins, and ventral colour (chr 15); and red colour pattern elements (chr 18). The various phenotypes controlled by the respective colour pattern loci are depicted. Note that whereas most phenotypes have descriptive names, the red blotch at the base of the forewing was termed ‘dennis’. (D) Phylogenetic relations obtained when building a tree from all captured regions compared to the neutral regions.

We obtained approximately 5.2 Mb of sequence distributed across 8 chromosomes from 473 individuals and 39 populations representing 10 species from the *H*. *melpomene* clade (S1 and S2 Tables). Phylogenetic reconstructions confirmed that *H*. *cydno* populations, with the sole exception of *H*. *c*. *cordula* found east of the Andes and in the Magdalena Valley, and *H*. *timareta* populations from east of the Andes cluster as separate lineages from the *H*. *melpomene* clade (Fig 1B and 1D). Phylogenetic inferences including all sequenced regions agreed with previous multilocus phylogenies, in which *H*. *cydno* and *H*. *timareta* form a sister clade to *H*. *melpomene* (Figs 1D and S1) [44,50]. The tree built using only neutral background data (i.e., regions a priori not suspected to be under mimicry selection, see Materials and methods) largely clustered populations according to geography, i.e., *H*. *cydno* with western *H*. *melpomene* and *H*. *timareta* with eastern *H*. *melpomene* subspecies (Fig 1B and 1D). The neutral topology is consistent with ongoing gene flow between sympatric populations resulting in highly heterogeneous relatedness patterns along the genome [71,72]. Six out of nine individuals with the dennis-ray pattern, sampled from the *H*. *melpomene vicina* population in the Colombian Amazon (Fig 1A and 1C), consistently clustered within *H*. *timareta*. This suggests the presence of a lowland population of *H*. *timareta* considerably further from the Andes than has been detected previously, hereafter referred to as *H*. *timareta* ssp. *nov*. (Colombia).

To assess demographic events, which may affect selection tests, we estimated effective population size across time for all populations with whole-genome data (S1 and S3 Tables). In line with previous studies [51,70], we found that bottlenecks were rare across those populations with the exception of a recent decline in population size in *H*. *heurippa* and older, moderate dips in *H*. *besckei* and *H*. *m*. *nanna* (S3 Fig).

### Signatures and limits of detection of classic sweeps assessed by simulations

We used forward-in-time simulations to investigate differences in the signals produced by classic as compared to introgressed selective sweeps in genome scan data, which have been relatively unexplored [27]. Our simulation results are intended to demonstrate qualitative patterns, but we also parameterise the simulations according to the *Heliconius* populations. This allows us to assess the time period over which sweeps can be detected in real data and place bounds on the timing of selection in natural populations. In our analysis, we primarily use SweepFinder2 (SF2), which is appropriate for our genomic data because it is able to identify the sweep site. This method is also robust to demographic processes [73,74], because these are incorporated in the null model used by SF2 (for more details, see Materials and methods). However, to more qualitatively explore patterns of diversity at sites undergoing selection, we here also present results for Tajima’s *D*.

**Fig 2:**
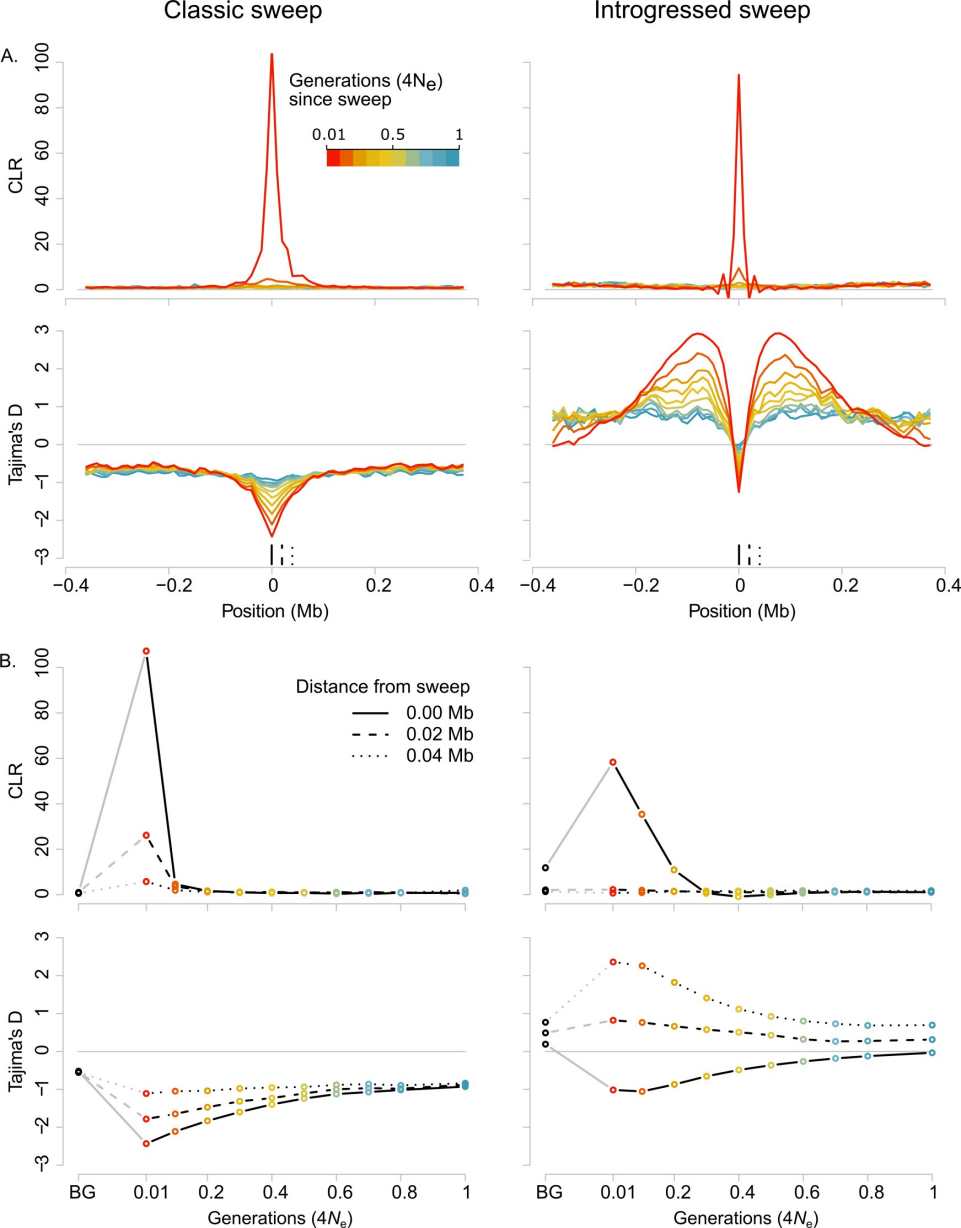
**SFS signatures of selection for simulated classic hard sweeps (left) and introgressed sweeps (right).** (A) CLR statistics (upper panel, [73,74]) and Tajima’s *D* (lower panel) across a simulated chromosome for different time points (0.01, 0.1, 0.2, 0.3, 0.4, 0.5, 0.6, 0.7, 0.8, and 1 in units of scaled generations, i.e., 4*N* generations) after a classic hard (left) or introgressed (right) sweep (effective migration rate *M* = 0.2). The sweep occurs in the centre of the simulated chromosome. Different colours indicate time since sweep. Full, dashed, and dotted vertical black lines in the lower panel indicate positions at different distances from the sweep centre for which time series of CLR and Tajima’s *D* statistics are depicted in panel B in the same style. (B) CLR (upper panel) and Tajima’s *D* (lower panel) statistics over time at 3 positions relative to the sweep centre as shown in panel A. Also shown are neutral background values, BG, calculated over neutral simulations, either without migration (left hand panels, for classic sweeps) or with migration at *M* = 0.2 (right hand panels, for introgressed sweeps). Time is given in units of scaled generations. Data are available from https://github.com/markusmoest/SelectionHeliconius.git. BG, background; CLR, composite likelihood ratio.

The time over which we can expect to detect sweep signals is determined by the time to coalescence and is thus determined by *N*, the (effective) population size. We therefore here report time since the sweep in generations, scaled by 4*N* [75]. Sweep signals are expected to decay rapidly because of the joint effects of mutation, recombination, and drift. Indeed, SF2, which uses the predicted effect of a selective sweep on the local site frequency spectrum (SFS) to infer the probability and location of sweeps [73,74,76], has low power to detect even hard selective sweeps that occurred over 0.25 (scaled) generations ago and cannot localise sweeps older than 0.4 (scaled) generations [74]. Consequently, any detected sweep signals in *H*. *melpomene* are likely under 0.8 Mya, assuming an effective population size of 2 million [70,77] and a generation time of 3 months [78]. These estimates vary with *N*, so the time limit for sweep detection varies among species, from only 0.2 Mya for *H*. *besckei* (*N* approximately 0.5 million) to 1.4 Mya for *H*. *erato* (*N* approximately 3.5 million). We used simulations to further interpret the empirical signatures of selection and explore the limits of detection (Fig 2).

We initially simulated the case of a hard sweep, such that *s* = 0.5, which is appropriate to the very strong selection pressure experienced by the colour pattern loci in *Heliconius* (Table 1). We found that SF2 signals broke down rapidly after the sweep (Fig 2). The magnitude of the CLR peak decreased by an order of magnitude after just 0.1 scaled generations, corresponding to 0.2 Mya for *H*. *melpomene*, and was not distinguishable from background values after 0.2 scaled generations, i.e., 0.4 Mya in *H*. *melpomene* (Welch *t* test, *p =* 0.065). Similarly, the estimated strength of selection calculated with SF2 from our simulations declined rapidly with time. Although the magnitude of the SF2 peak is affected, we find that the time for which we can detect selective sweeps does not change if we vary either the strength of selection (using alternative values of *s* = 0.1 and *s* = 0.25), or the mutation rate, which was scaled up such that levels of neutral diversity in our simulations are equivalent to those seen in our *Heliconius* populations (S4 Fig and S4 Table). Levels of linkage disequilibrium were in the range of the empirical data for all simulated scenarios (S4 and S15–S18 Tables).

### Signatures and limits of detection of introgressed sweeps assessed by simulations

We extended our simulations to explore the expected SFS signature left by an allele undergoing adaptive introgression, by simulating a second population which exchanged migrants with the first, leading to an introgressed sweep in the second population. Adaptive introgression produces a highly distinctive SFS signature. At and very close to the selected site itself there was a reduction in diversity and an excess of rare alleles, similar to the pattern observed for a classic sweep. However, this reduction was narrow and flanked by broad genomic regions with high diversity and an excess of intermediate frequency variants. This is due to variants that have hitchhiked into the recipient population along with the beneficial variant and subsequently recombined before reaching fixation [20,27]. The overall SFS signature covered a considerably wider genomic area than that of a classic sweep (Fig 2).

The introgression signature we observe at the sweep site itself was very similar to that for a classical sweep, and we could detect it for a similar length of time. SF2 managed to detect introgressed sweeps, although it detected only the central region of lowered diversity, producing a high but very narrow CLR peak at the sweep site itself; this contrasts with the peaks for classic selective sweeps, which extended over a wider genomic area (Fig 2). The distribution of CLR values at the sweep site was significantly different from values calculated over neutral regions for up to 0.1 generations after the sweep (*p =* 0.0041). However, as for a classical sweep, the magnitude of the peak decreased rapidly.

In the simulations described above, we used an effective migration rate of *M* = 0.2. Estimates of *M* between hybridising *Heliconius* species vary from 0.08 to 10 migrants per generation [47–49], and so we also explored a broad range of values of *M*, from 0.02 to 200, in order to cover the estimated range for *Heliconius* (S5 Fig). We find that the the reduction of diversity at the introgression site itself is strongly affected by migration rate. As *M* increases, the central reduction in diversity becomes less pronounced, representing an increasingly ‘soft’ introgressed sweep (S5 Fig) [21,79]. Therefore, detecting introgressed sweeps from this central region will be difficult in populations in which *M* is high. However, for values of *M* below 2, varying *M* had little effect on the regions of increased diversity and excess of intermediate frequency variants that flank the sweep locus (S5 Fig).

### Strong signatures of selection across *Heliconius* colour pattern regions

**Fig 3:**
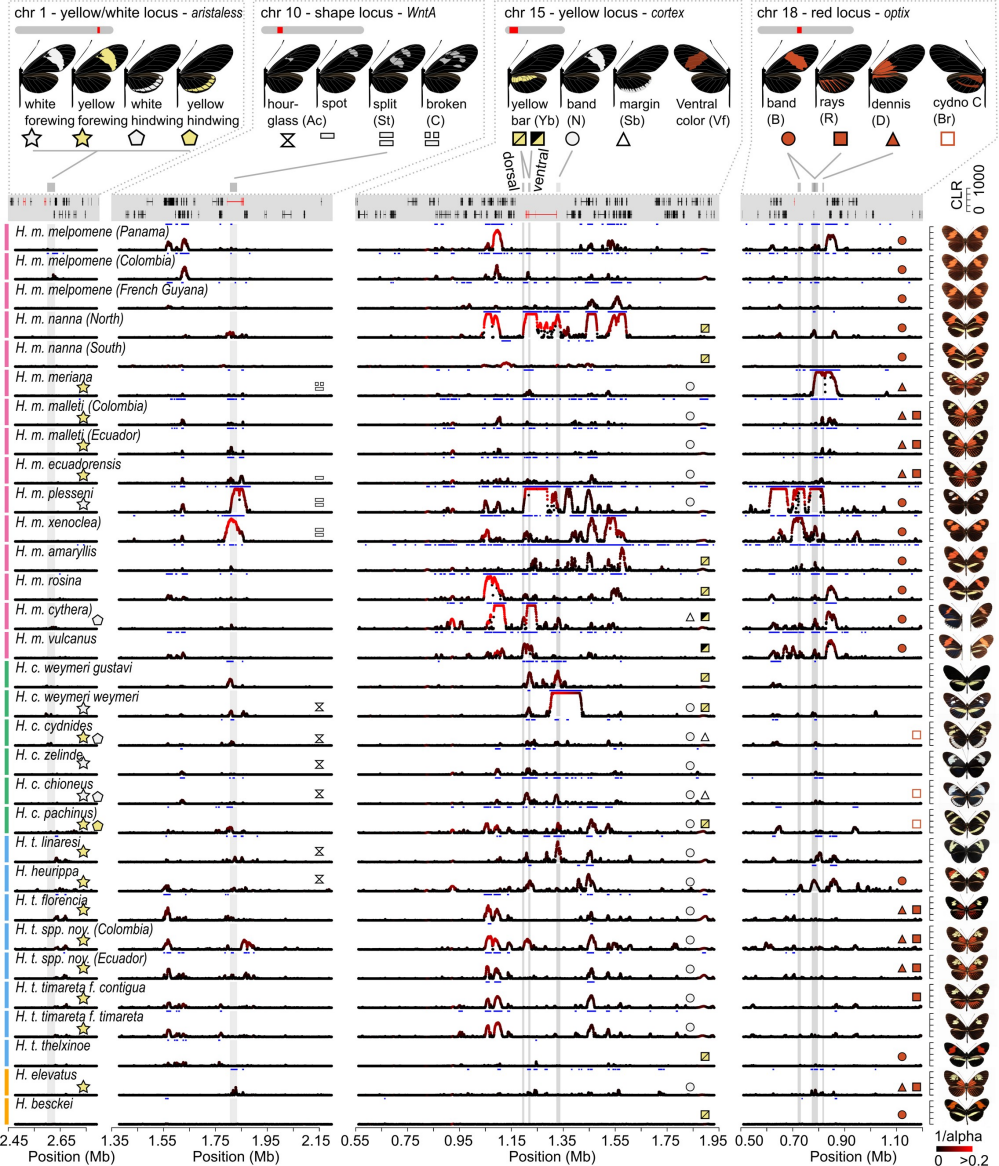
Signature of selection across colour pattern regions in the *H*. *melpomene* clade. The regions containing the tandem copies of *aristaless*, *al1* and *al2*, *WntA*, *cortex*, and *optix* (left to right) are depicted. Colour pattern genes are annotated in red in the gene annotation panel. On the y-axis Sweepfinder2’s CLR statistics is shown (peaks capped at 1,000). The colour gradient indicates the estimated intensity of selection *α* [73] (black = high *α* values, weak selection; red = low *α* values, strong selection). Grey shadings indicate annotated colour pattern CREs [30,36,37,39] (S7–S10 Figs). Blue horizontal bars indicate regions with CLR values above threshold. Top panel shows colour pattern phenotypes and symbols indicate distinct colour pattern elements, and their presence is annotated in population panels. Note that the yellow hindwing bar controlled by the *cortex* region can be expressed on the dorsal and ventral side (yellow/yellow square symbol) or on the ventral side only (black/yellow square symbol) [39]. Moreover, the actual shape of the forewing band can depend on the allelic state of *WntA*. Full, grey lines connect colour pattern elements with annotated CREs. Phenotypes are depicted on the right. Data are available from https://github.com/markusmoest/SelectionHeliconius.git. CLR, composite likelihood ratio; CRE, *cis*-regulatory element.

In our empirical data, SF2 found strong support for positive selection acting across multiple populations and species for all 4 colour pattern loci (Fig 3). In contrast, our background regions, as well as regions flanking the colour pattern associated loci, showed little evidence of sweeps, apart from a few isolated examples (S6 Fig).

This is consistent with previous genome-wide selection scans in *H*. *melpomene* that detected only a few strong sweep signatures [70]. These results therefore lend support to the long-standing assertion that wing patterning loci are among the most strongly selected loci in the genome and have a distinctive evolutionary history [80], without excluding the potential presence of other local sweeps in the respective populations.

Broadly, signals of selection were stronger and more widespread in regions near *cortex* and *optix* and weaker near *WntA* and *aristaless*. For example, all 31 populations showed sweep signals above threshold near *cortex*, 26 near *optix*, 24 near *WntA*, albeit less pronounced in most cases, and only 7 near *aristaless* (Fig 3 and S5–S8 Tables). A similar pattern was reflected in our estimates for strength of selection (*s*) calculated from *α* estimates (Table 2; see Materials and methods for a detailed description and formula for this calculation) with the highest selection strength at colour pattern loci being *s* = 0.141 for the *cortex* (*H*. *m*. *nanna*), *s* = 0.036 for the *optix* (*H*. *m*. *plesseni)*, *s* = 0.049 for the *WntA* (*H*. *m*. *xenoclea*), and *s* = 0.01 (*H*. *t*. *florencia*) for the *aristaless* region (*H*. *t*. *florencia*). These patterns are broadly concordant with the expected phenotypic effects of these loci. For example, in *H*. *cydno*, which has primarily yellow and/or white patterns associated with the *cortex* region [39,81], significant peaks were mostly found at this locus, whereas in *H*. *melpomene*, which has red, yellow, and white patterns, strong signals were seen at both *cortex* and *optix* regions. Consistently, a lower strength of selection was found for the *aristaless* region (*s* < 0.01), which controls a modification of pale patterns from yellow to white that is putatively less salient to predators [82] and may contain fewer potential targets of selection.

**Table 2:** Position, CLR statistics, and estimates for strength of selection (*α*, 2*N*_*e*_*s*, and *s*) for populations and sweeps discussed in detail. Annotated colour pattern genes and CREs that overlap with peaks are given. Positions are given in Hmel2 scaffold coordinates (see S5 and S7 Tables).

Population	Colour pattern region	Position	CLR	α	2*N*_*e*_*s*	*s*	Annotated colour pattern gene or CRE
*H*. *m*. *plesseni*	*WntA*	1829355	1098	6.3	95215	0.035	*WntA* gene, 1. exon^a^
*H*. *m*. *xenoclea*	*WntA*	1811430	971	4.54	118013	0.049	*WntA* exon, 1. exon^a^
*H*. *c*. *weymeri f*. *weymeri*	*cortex*	1337975	2411	5.3	115568	0.065	next to UTR4 of *cortex* gene^b^
	*cortex*	1218021	367	20.74	29538	0.017	*cortex* gene, ventral Yb^c^
*H*. *m*. *meriana*	*optix*	801534	1250	9.45	35360	0.023	*dennis* CRE^d^
*H*. *m*. *plesseni*	*optix*	643924	2174	6.07	48223	0.035	upstream of *optix*
		732278	1638	6.21	47109	0.034	*band* CRE1^e^
		783431	2371	6.97	41978	0.03	*band* CRE2^e^
*H*. *m*. *xenoclea*	*optix*	727532	1182	9.74	37910	0.022	*band* CRE1^e^
*H*. *e*. *notabilis*	*WntA*	4648024	909	14.09	66925	0.011	*Sd* region^f^
*H*. *e*. *notabilis*	*cortex*	2497650	1387	15.2	93112	0.015	*WAS homologue 1*^b^
		1963287	472	49.76	28438	0.005	*Cr1*^f^
*H*. *e*. *demophoon*	*cortex*	2277009	1050	13.99	103964	0.016	*Cr2*^f^
*H*. *e*. *notabilis*	*optix*	1294528	4690	3.03	370210	0.059	*optix* gene and CREs^f^

^a^Mazo-Vargas and colleagues [83]

^b^Nadeau and colleagues [30]

^c^Enciso-Romero and colleagues [39]

^d^Wallbank and colleagues [36]

^e^Hanly [37]

^f^Van Belleghem and colleagues [38]

**Abbreviations:** CLR, composite likelihood ratio; CRE, cis-regulatory element

**Fig 4:**
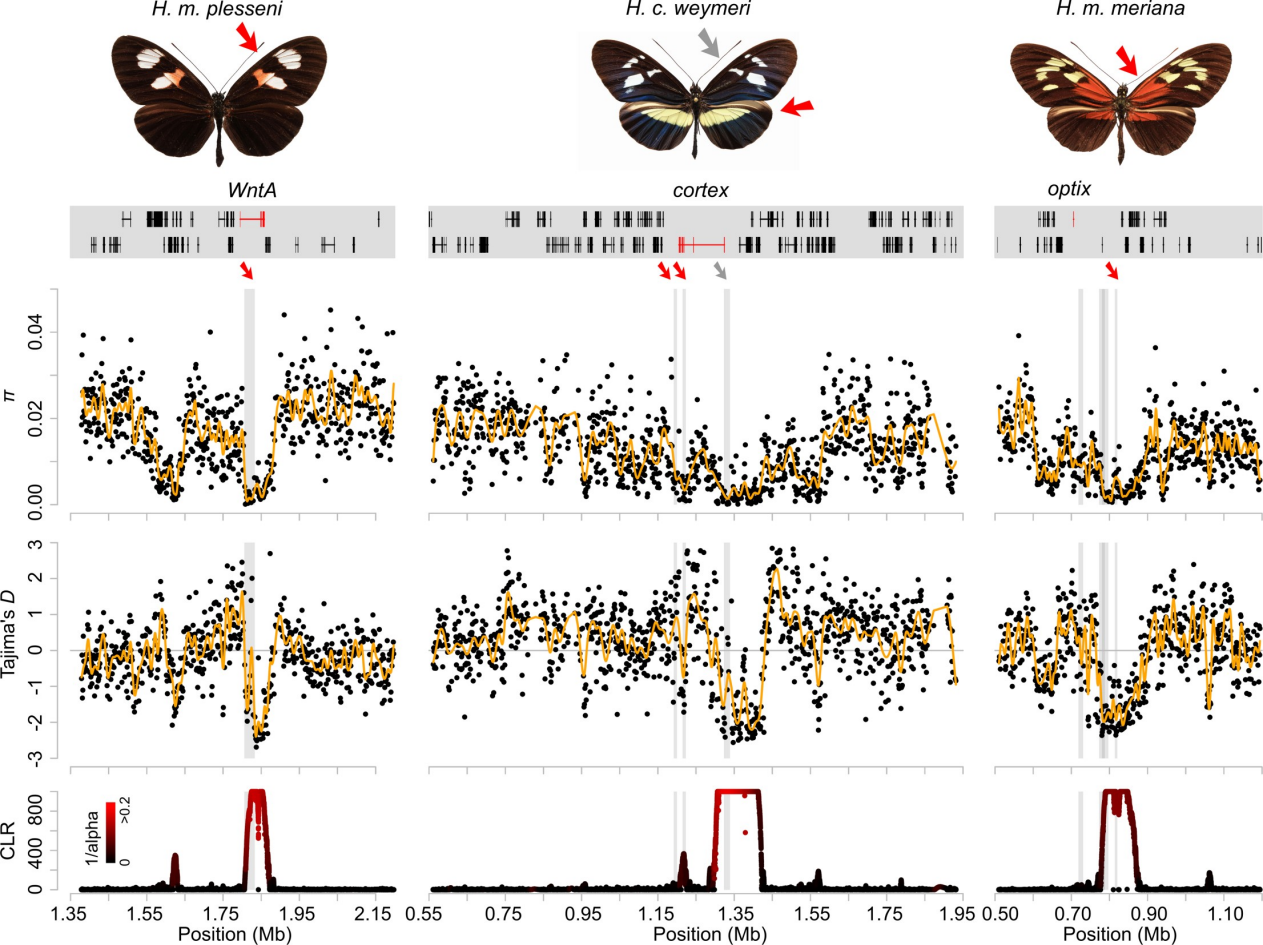
Selected examples of sweeps. The 3 examples show the split forewing band (*WntA* region) in *H*. *m*. *plessini*, the yellow and white patterns (*cortex* region) in *H*. *cydno weymeri f*. *weymeri* and the red dennis patch (*optix* region) in *H*. *m*. *meriana* (left to right). The respective colour pattern elements are indicated with red and grey arrows. Colour patterns and gene annotations in the colour pattern regions are depicted in the top panel. Colour pattern genes are annotated in red. Nucleotide diversity *π*, Tajima’s *D*, and SweepFinder2’s CLR statistics (peaks capped at 1,000) show the signatures of a selective sweep (bottom panels). Loess smoother lines are depicted in yellow. The colour gradient in the CLR panel indicates the estimated intensity of selection *α* [73] (black = high *α* values, weak selection; red = low *α* values, strong selection). Grey shadings indicate annotated CREs, and red and grey arrows depict associations with the respective colour pattern elements in the *H*. *melpomene* clade. Data are available from https://github.com/markusmoest/SelectionHeliconius.git. CLR, composite likelihood ratio; CRE, *cis*-regulatory element.

There were also differences seen across the sampled populations. Widely distributed colour patterns (e.g., *H*. *m*. *melpomene* and *H*. *m*. *malleti*) tended to show only modest evidence for selective sweeps (Figs 3 and S11). Comparisons with our simulated data nonetheless suggest selective events that occurred no more than 400,000 years ago. Although there was no significant general correlation between distributional ranges of populations and evidence for selection (S12 and S13 Figs), the strongest signatures of selection were found in geographically localised patterns and likely reflect sweeps within the last 100,000 years (Fig 4 and Table 2). For example, *H*. *m*. *plesseni* is exclusively found in the upper Pastaza valley in Ecuador and shows a unique split red-white forewing band (Figs 1 and 4). This population showed strong selection at 3 colour pattern regions—*optix*, *cortex*, and *WntA*—suggesting recent selection acting on the entire pattern (*s*_*cortex*_ = 0.074, *s*_*WntA*_ = 0.035, and *s*_*optix*_ = 0.035), and patterns of both nucleotide diversity and Tajima’s *D* are consistent with strong classic sweeps (Figs 3, 4 and S11 and S5 Table). *H*. *m*. *xenoclea*, also found on the eastern slopes of the Andes but further south in Peru, shows the same split forewing band associated with the *WntA* region and again a very strong selection signal at this locus (*s*_*WntA*_ = 0.049), as well as weaker signatures at *cortex* (*s*_*cortex*_ = 0.04) and *optix* (*s*_*optix*_ = 0.022; Figs 3 and S11 and S5 Table). The clear signatures of recent and strong selection pressure perhaps indicate that the split forewing band is a novel and highly salient signal. Additionally, *H*. *m*. *meriana* from the Guiana shield revealed a striking signature of selection at *optix* (*s*_*optix*_ = 0.023). Its dennis-only pattern (Fig 4) has previously been shown to have arisen through recombination between adjacent dennis and ray regulatory modules at *optix*, and the signature of selection at this locus, which encompasses both of these regulatory modules, implies a recent sweep of this recombinant allele [36] (Figs 3, 4 and S11 and S5 Table).

In light of our simulations of introgressed sweeps, there were cases in our data in which previously well-documented adaptive introgression events showed signatures characteristic of introgressed sweeps. The hindwing yellow bar pattern was shown to have introgressed from *H*. *melpomene* into *H*. *c*. *weymeri* and then back again into the races *H*. *m*. *vulcanus* and *H*. *m*. *cythera* [39]. Accordingly, we found narrow SF2 peaks and an increase in Tajima’s *D* at surrounding sites at these modules in the *cortex* region in *H*. *m*. *cythera*, *H*. *m*. *vulcanus*, and *H*. *c*. *weymeri*, consistent with introgressed sweeps (Fig 3 and S11 Fig). *H*. *c*. *weymeri f*. *weymeri* also had a second, striking signature further upstream more typical of a classic sweep (Figs 3 and 4), at a region associated with the yellow forewing band in *H*. *melpomene* and *H*. *timareta* [30]. This is consistent with evidence for a role of *cortex* in controlling the white forewing band in *H*. *cydno* [81] and the presence of this band in the *weymeri* morph, which could therefore represent a recent evolutionary innovation. Other loci previously implicated as having introgressed include the *optix* region in *H*. *heurippa* and *H*. *elevatus*, which both showed signals coinciding with regions previously associated with the respective phenotypes [36,37]. In contrast, there was a lack of clear introgressed sweep signals in dennis-ray *H*. *timareta*, which is one of the best documented examples of introgression. This could be explained by the age of the sweeps and/or high rates of migration, which our simulations show can reduce the sweep signal in the recipient population (S5 Fig). We also performed scans with VolcanoFinder, a new method designed to detect SFS signatures created by introgressed sweeps [27]. Similar to SF2, VolcanoFinder detected strong signatures of selection in colour pattern regions in the respective populations but not in the neutral background regions (S14–S16 and S19 Figs). However, the estimated divergence values (D) did not allow for a clear distinction of introgressed from classic sweeps in our data.

### Novel targets of selection in colour pattern regions

Many of the signals of selection we detected overlap with previously identified regulatory regions associated with colour pattern variation. However, our analysis also found additional nearby regions showing consistent signals of selection that may also be involved in colour pattern evolution (Figs 3 and S17). For example, in the first intron of the *WntA* gene, we found a consistent signal across several *H*. *melpomene*, *H*. *timareta*, and *H*. *cydno* populations (S17B Fig). Within this region (Hmel210004:1806000–1833000), phylogenetic clustering of the 2 split forewing band races *H*. *m*. *plesseni* and *H*. *m*. *xenoclea* indicates a common origin of the split band in these currently disjunct populations (S7 Fig). Additionally, 2 strong selection signatures are frequently found in a region approximately 200 kb upstream of *WntA* (S17B Fig; Hmel210004:1550000–1650000), which suggests additional loci involved in colour pattern regulation.

Near *cortex*, selection signatures at closely linked genes support findings from previous studies. Several populations show distinct peaks upstream and downstream of *cortex* and broadly coincide with a wider region, possibly containing several genes involved in colour pattern regulation [30,84] (S17C Fig). Multiple peaks are located upstream of *cortex* within an array of genes that all showed significant associations with yellow colour pattern variation [30] (S9 Table). A particular concentration of signals fell near the serine/threonine-protein kinase gene *LMTK1* (HMEL000033; Hmel215006:1,418,342–1,464,802) and close to *washout*. The latter gene is involved in actin cytoskeleton organization in *Drosophila* [85] and previously showed a strong association with the yellow forewing band [30] as well as differential expression patterns between different *H*. *numata* morphs [84]. Likewise, selection signals clustered downstream of *cortex* in a region containing additional candidate genes identified previously (S9 Table). In the *optix* region, consistent signals across several populations indicated that several as yet uncharacterised elements may be under mimicry selection. Intriguingly, a *kinesin* motorprotein gene, which shows an association of expression with the red forewing band [86,87], was among these (S17D Fig).

### Parallel selective sweep signatures between mimetic species

**Fig 5:**
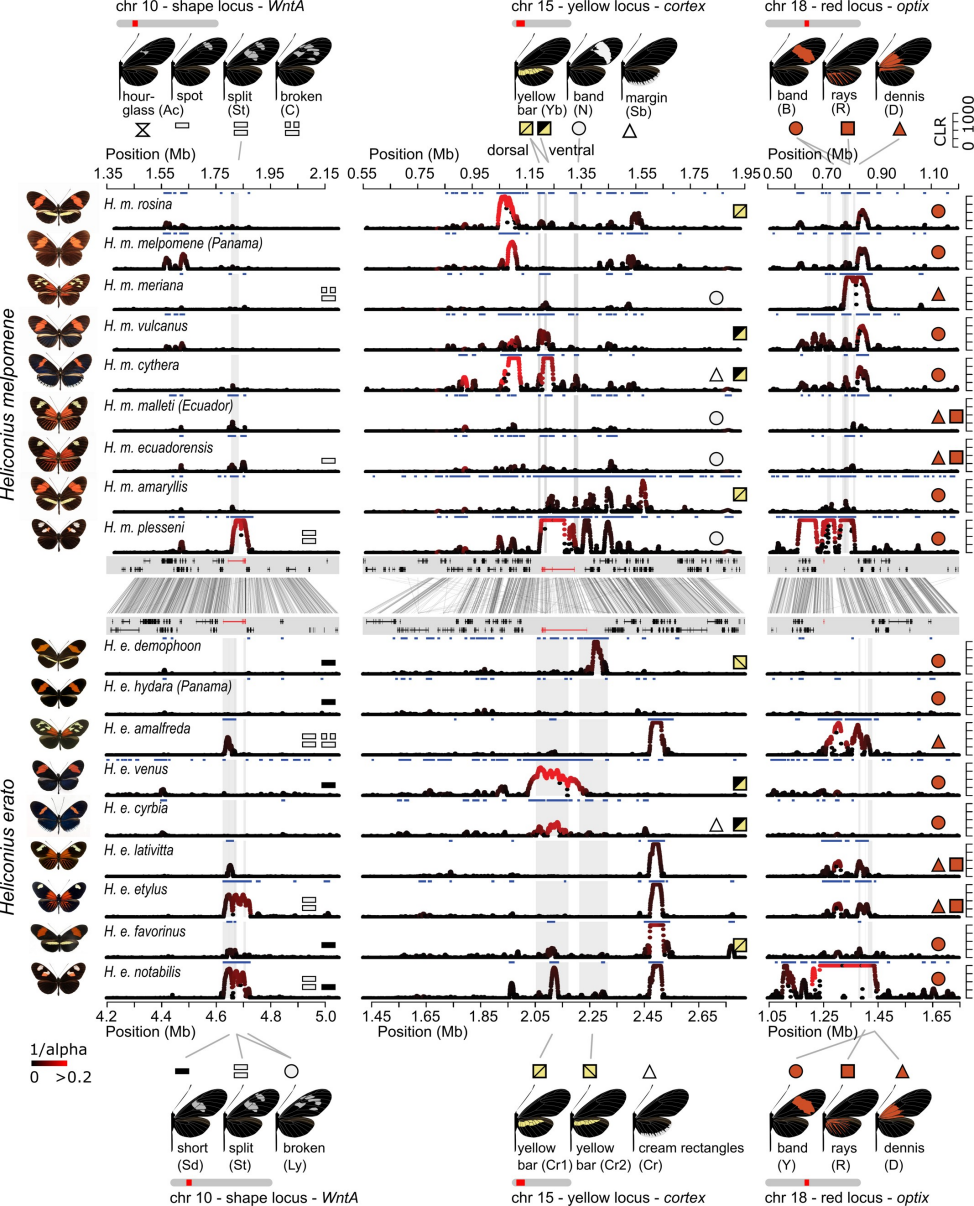
**Signatures of selection in the co-mimic populations of *H*. *melpomene* (upper panels) and *H*. *erato* (lower panels).** The regions containing *WntA*, *cortex*, and *optix* are shown (left to right). Co-mimics in *H*. *melpomene* and *H*. *erato* are depicted in the same order with phenotypes on the left. The y-axis indicates CLR statistics across each region (capped at 1,000). The colour gradient indicates the estimated intensity of selection *α* [73] (black = high *α* values, weak selection; red = low *α* values, strong selection). Grey shadings indicate annotated colour pattern CREs [30,36,37,39] (S7–S10 Figs) and blue horizontal bars indicate regions with CLR statistics above threshold. The central panel shows an alignment of the respective regions in *H*. *melpomene* and *H*. *erato* and gene annotations with colour pattern genes in red. Top and bottom panel show colour pattern phenotypes, and symbols indicate distinct colour pattern elements and their presence in each population panel. Note that the yellow hindwing bar controlled by the *cortex* region can be expressed on the dorsal and ventral side (yellow/yellow square symbol) or on the ventral side only (black/yellow square symbol) [39]. Full, grey lines connect colour pattern elements with annotated CREs. Note that the genetics of the yellow forewing band differs between *H*. *erato*¸ in which it involves the *WntA* and *optix* locus, and *H*. *melpomene*, in which the band is controlled by the *cortex* and its shape by the *WntA* region. Data are available from https://github.com/markusmoest/SelectionHeliconius.git. CLR, composite likelihood ratio; CRE, *cis*-regulatory element.

There has been considerable interest in whether the *H*. *erato* and *H*. *melpomene* co-mimics have co-diverged and simultaneously converged onto the same colour pattern [88–91] or whether one species evolved towards diverse phenotypes of the other, i.e., advergence [67,92–94]. Homologous genes control corresponding phenotypes [30,35,95,96], but there is no allele sharing between the *melpomene* and *erato* clade [67,68]. We used published genomic data for *H*. *erato* (Van Belleghem and colleagues, 2017; S10 Table) to obtain 8.9 Mb of sequence homologous to the regions studied in the *H*. *melpomene* clade for 103 individuals from 13 populations and 3 species in the *H*. *erato* radiation and scanned for selective sweeps. Generally, a comparison of the location of selection peaks between *H*. *melpomene* and *H*. *erato* across several co-mimetic races suggests a rather simple and concordant regulatory architecture in the 2 species at the *WntA* locus. However, in the *cortex* and *optix* regions, this architecture appears to be more complex and differs more strongly between the 2 clades (Figs 5, S17 and S18).

Similar to the *melpomene* clade radiation, we found strong signatures of selection across the *optix*, *cortex*, and *WntA* regions (Figs 5 and S20–S22 and Tables 2 and S11–S14). Most notably, *H*. *e*. *notabilis* from Ecuador showed strong signals of selection at 3 colour pattern loci (*s*_*optix*_ = 0.06, *s*_*cortex*_ = 0.015, *s*_*WntA*_ = 0.015) similar to its co-mimic *H*. *m*. *plesseni* (Table 2). In both cases, selection across the 3 major loci represented some of the strongest signals in both species. Additionally, *H*. *e*. *amalfreda*, co-mimic with the red dennis-only race *H*. *m*. *meriana*, showed one of the strongest selection signals at *optix*. This suggests that these phenotypes are recent innovations in both species, consistent with co-divergence. Other geographically localised variants controlled by *WntA* also showed strong signals of selection, indicating a recent origin. For example, *H*. *e*. *etylus*, like *H*. *m*. *ecuadoriensis*, has a restricted forewing band shape that corresponds to the more distal element of the *notabilis* forewing band (*s*_*WntA*_ = 0.015). Clear, narrow, and very similar selection signals were found near *WntA* in *H*. *e*. *amalfreda* and *H*. *e*. *erato* (*s*_*WntA*_ = 0.006 in each), both with a broken forewing band, as well as *H*. *e*. *emma* (*s*_*WntA*_ = 0.003) and *H*. *e*. *lativitta* (*s*_*WntA*_ = 0.004), both with a narrow forewing band (S11 Table).

More broadly across the *H*. *erato* populations, there was a clear difference between the Amazonian dennis-ray races (i.e., *H*. *e*. *amalfreda*, *H*. *e*. *erato*, *H*. *e*. *emma*, *H*. *e*. *etylus*, and *H*. *e*. *lativitta*), all exhibiting a similar selection pattern at *optix*, and red forewing band races (*H*. *e*. *favorinus*, *H*. *e*. *venus*, *H*. *e*. *cyrbia* and *H*. *e*. *hydara* in Panama, and *H*. *e*. *demophoon*) which showed little or no signature of selection. This is in agreement with the hypothesis that the widespread dennis-ray phenotype at *optix* has a more recent origin as compared with the red band phenotype [67]. One notable exception to this pattern was *H*. *e*. *hydara* in French Guiana, the only red banded *H*. *erato* form with a strong signal at *optix* (*s*_*optix*_ = 0.09). There are slight variations across the range in the band phenotype, and perhaps a recent modification of the band phenotype swept in this population. The pattern in *H*. *melpomene* is less clear, possibly due to the age of the alleles and the considerably lower effective population size in *H*. *melpomene*.

At the *cortex* locus, there was a consistent peak centred on *lethal (2)* just next to the cytokine receptor gene *domeless*, which in *Drosophila* is essential for the JAK/STAT signalling pathway controlling embryonic segmentation and trachea specification [97], and *washout* (annotated in S18 Fig). However, surprisingly, the signal is almost identical across populations with a variety of different yellow colour pattern phenotypes (*H*. *e*. *amalfreda*, *H*. *e*. *erato*, *H*. *e*. *hydara* in French Guiana, *H*. *e*. *emma*, *H*. *e*. *etylus*, *H*. *e*. *lativitta*, *H*. *e*. *notabilis*, *H*. *e*. *favorinus*, *H*. *himera*) and completely absent in Northwestern populations (*H*. *e*. *cyrbia*, *H*. *e*. *venus*, *H*. *e*. *hydara* in Panama, *H*. *e*. *demophoon*; S20 Fig). The sweep signal therefore shows little obvious association with any particular wing pattern phenotype but may still indicate a locus involved in the colour pattern pathway. In addition, we detected very distinct signals between *H*. *e*. *favorinus* (*Cr1*) and *H*. *e*. *demophoon (Cr2)* consistent with previous studies [30,38,98] that found evidence for independent evolution of the yellow hindwing bar on either side of the Andes. Although *H*. *e*. *favorinus* lacks any signature at *Cr2* and shows a weak signal at *Cr1*, a clear peak was found for *H*. *e*. *demophoon* at *Cr2* indicating that this allele may be more recent (Figs 5, S18 and S20).

## Discussion

**Fig 6:**
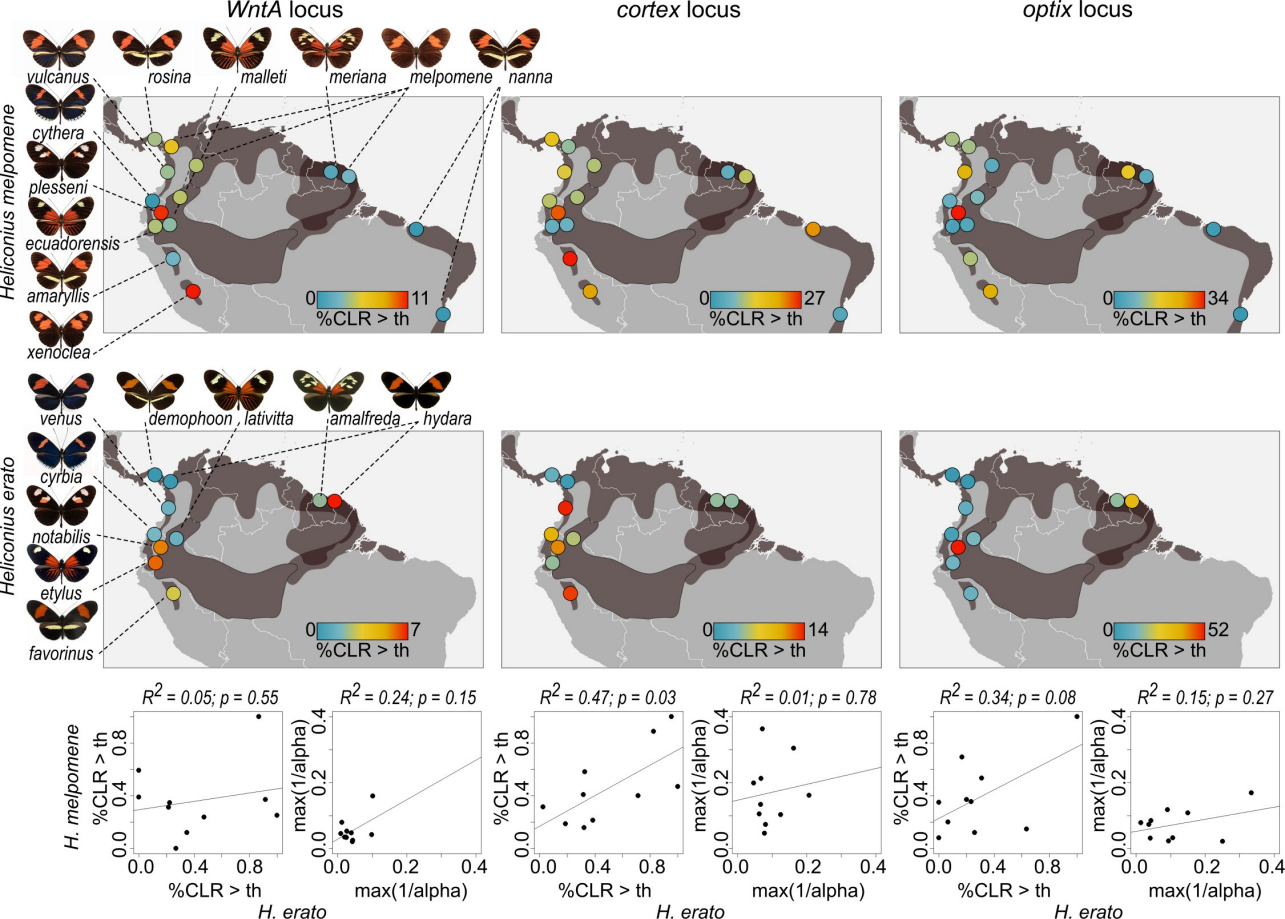
**Geographic mapping of colour pattern selection in *H*. *melpomene* (top) and *H*. *erato* (middle).** Dark grey shadings indicate distributional ranges of the depicted colour patterns. Coloured circles indicate the colour pattern selection summarised as percentage of CLR values across the colour pattern region that are above the CLR threshold [%CLR>th] scaled by the maximum value for *WntA*, *cortex*, and *optix* regions (left to right) in *H*. *melpomene* (top) and *H*. *erato* (middle). The bottom panel shows correlations for percentage CLR values above threshold [%CLR>th] and maximum intensity of selection *α* [73] (max(1/*α*)) between *H*. *melpomene* and *H*. *erato*. Data are available from https://github.com/markusmoest/SelectionHeliconius.git. CLR, composite likelihood ratio.

Elucidating the evolutionary history and spread of advantageous variants in natural populations lies at the heart of evolutionary research, ever since Wallace [99] and Darwin [100] established the theory of evolution by natural selection. However, detecting and quantifying selection has been a challenge, particularly in wild populations [3]. We have combined a large data set of high coverage genomic data with novel theoretical analyses to identify molecular signatures of recent selection at genes known to control adaptive wing patterning traits in *Heliconius* butterflies. We demonstrate that these strongly selected loci have been subject to recent bouts of natural selection even within the last 100,000 years, with geography and phenotype standing out as strong predictors of selection (Fig 6).

Many studies have used naive genome scans to identify selection in natural populations, but such an approach can lead to false positives [101]. More integrative approaches, which combine selection scans with information on phenotypic selection in the wild and genetic trait mapping, can give a more complete picture of how selection shapes specific loci and phenotypes [10,12,14,16,102]. Such studies are increasingly common but with few exceptions focus on a single locus, or a limited set of populations or phenotypes, often because of the high sampling and sequencing effort required. We take advantage of 150 years of *Heliconius* research, including field selection experiments, hybrid zone studies, detailed dissection of the genetics of colour pattern elements, and introgression studies, to survey genomic signatures of selective sweeps across many populations and loci. With our study design, we reconcile large geographic sampling and high-coverage sequence data by targeting well-defined regions in the genome. This combination of ‘top-down’ and ‘bottom-up’ approaches, as defined by Linnen and Hoekstra [1], reveals pervasive evidence for the action of natural selection on mimicry loci in an adaptive radiation associated with a great diversity of phenotypes.

We have shown a pervasive pattern of strong selection acting on mimicry colour patterns, which contrasts strongly with the regions flanking the selected loci and neutral background genome regions. This supports the assertion of ‘contrasted modes of evolution in the genome’, first formulated by John R. G. Turner 40 years ago [80], who concluded that mimicry genes and neutral parts of the genome were subject to different modes of evolution. Of course, our data do not preclude the existence of other strongly selected loci not associated with mimicry in the genome. The frequency of evidence for selection is consistent with the large effective population sizes in *Heliconius* that preserve the signature of selective sweeps over a relatively long period of time. Our estimates of selection strength indicate strong selection acting on mimicry genotypes, which is in line with field and hybrid zone studies on the colour pattern phenotypes (Tables 1, S6 and S11) and strong selection on colour polymorphisms in other species [1,10,103]. *Heliconius* butterflies therefore join a small group of systems for which strong natural selection on ecologically important traits has been documented in detail at both the phenotypic and molecular level [1,2]. Other examples include Darwin’s finches, in which climate-driven changes in seed size and hardness imposed strong selection on beak size and body weight [15,104,105], industrial melanism in the peppered moth *Biston betularia* [103,106], the body armour locus *Eda* in sticklebacks [107], and crypsis in *Peromyscus maniculatus* deer mice controlled by the *Agouti* pigment locus [16].

However, both strength and direction of selection can vary substantially in time and space, and a snapshot of a single population may be misleading about the action of selection in the wild [105,107–109]. One way to account for this variation is by studying patterns of selection in geographically widespread adaptive radiations, comprising ecological replicates. This approach allows us to describe general patterns in the action of selection on a continental scale. For example, there is consistently stronger selection on the *optix* and *cortex* loci across the range of these species, consistent with the greater phenotypic effect of alleles at these loci. In addition, we also identify what seem to be more recent phenotypes showing a stronger signature of selection, such as the split band phenotype in the Andes and the dennis-only phenotype on the Guiana shield (Fig 6).

One of the defining characteristics of the *Heliconius* radiation has been the importance of adaptive introgression and recombination of pre-existing variants in generating novelty [36,39,44]. We used simulations to explore the expected patterns resulting from both new mutations and introgressed selective sweeps. These demonstrated a distinct signature of selection on introgressed variation, consistent with recent theory [27], and revealed that depending on the frequency of the acquired variant, introgressed sweeps show a range of characteristics reminiscent of classic sweeps. Consistently, we found that tests designed for detecting classic sweeps can also detect introgressed sweeps, but the signal becomes narrower, and the time window for detection decreases. In addition, the power to detect selection decreases with increasing effective migration rate between hybridising species. These conclusions may explain the scarcity of selection signatures in the *H*. *timareta* populations that represent well-documented recipients of adaptive introgression but also show strong genome-wide admixture, suggesting relatively high migration rates with *H*. *melpomene* [36,44,72]. Nonetheless, we detected putative introgressed sweeps in *H*. *c*. *weymeri*, *H*. *m*. *cythera*, *H*. *m*. *vulcanus*, and *H*. *heurippa*, for which acquisition of colour pattern phenotypes via adaptive introgression has been demonstrated and introgressed genomic intervals were identified [39,87,110]. We also attempted to implement a new method for detecting introgressed sweeps directly (VolcanoFinder), but although this method detected signatures of selection (S14–S16 and S19 Figs), it did not strongly differentiate classic and introgressed sweeps in our data [27]. The signatures were broader but largely congruent with the SF2 results. Although VolcanoFinder found strong signals for most *H*. *timareta* populations as well as the *cortex* region in *H*. *cydno* and *H*. *melpomene* populations West of the Andes, the estimated divergence values were inconclusive, most likely a consequence of low divergence between donor and recipient, ongoing admixture, and a complex history of selective events in our particular system. Therefore, combining prior phylogenetic evidence for introgression with scans for selection is likely to remain a powerful means to study adaptive introgression [111,112].

Our results imply a complex history in which multiple bouts of selection have occurred at the same loci. Although recurrent sweeps can alter or even eradicate previous signatures [5], there is nonetheless evidence for sweeps, both at previously characterised genomic regions and in novel locations. Previously, regulatory loci have been identified based on association studies across divergent populations [36,39,38], and many of these regions indeed show strong signatures of selection providing further support for their functional roles. However, consistent signatures of selection are also found at nearby loci, suggesting additional targets of selection, some of which had not previously been identified using top-down approaches. Some caution is required, because the signatures of selective sweeps are notoriously stochastic and can be misleading in their precise localisation because of linkage. Nonetheless, there are consistent patterns across multiple populations suggesting additional targets of selection that may represent regulatory elements affecting already characterised genes [36,39], similar to multiple mutations under selection at the *Agouti* gene in deer mice (*Peromyscus maniculatus*) [10]. In addition, however, some of these signals may represent selection at linked genes, and the architecture of colour pattern in *Heliconius* may be comparable to the situation in *Antirrhinum* snapdragons in which loci encoding flower pattern differences, i.e., *ROSEA* and *ELUTA*, are in tight linkage.[12]. Further functional studies will be required to unravel the roles of these loci, but theory suggests that physical linkage between genes contributing to the same adaptive trait can be favoured [113,12]. Intriguingly, *Heliconius* butterflies show both unlinked colour pattern loci, as well as tightly linked CREs and genes within loci, putatively preserving locally adaptive allelic combinations. It is conceivable that this architecture provides a high degree of flexibility that has facilitated the radiation of colour patterns in *Heliconius*.

Müllerian mimics can exert mutual selection pressures, offering the rare opportunity to study replicated selection in a co-evolutionary context. The diversity of mimicry alleles between *H*. *melpomene* and *H*. *erato* evolved independently [67,68], but several co-mimics between the 2 radiations show signatures of selection in homologous colour pattern regions, demonstrating repeated action of natural selection between co-mimics over recent time. Our findings also contribute to long-standing arguments on the origin and spread of the colour patterns [67,88–94]. Signatures of selection at the *optix* locus, particularly in *H*. *erato*, are consistent with the hypothesis that the red forewing band is ancestral and the dennis-ray is a younger innovation that spread through the Amazon. However, in contrast to this ‘recent Amazon’ hypothesis, we find the strongest signatures of selection in some of the unique and geographically restricted phenotypes found in Andean populations suggesting novel colour patterns have experienced strong recent selection in both species, consistent with co-divergence and ongoing co-evolution (Fig 6). The most striking examples are *H*. *e*. *notabilis* and *H*. *m*. *plesseni*, which show imperfect mimicry (see Fig 5) and are possibly still evolving towards an adaptive optimum. In summary, our results provide evidence for co-divergence and the potential for co-evolution in the sense of mutual evolutionary convergence [93] but do not rule out advergence in other cases.

To conclude, understanding the adaptive process that creates biodiversity requires knowledge of the phenotypes under selection, of their underlying genetic basis, and estimates of phenotypic and genotypic strength and timing of selection [1]. Although decades of *Heliconius* research have resulted in a detailed understanding of most of these levels, our study fills a gap by providing estimates of the distribution and strength of genotypic selection across 2 radiations and dozens of populations. However, our results not only highlight the complexity of mimicry selection across the *Heliconius* radiation but also reveal a surprisingly dynamic turnover in colour pattern evolution, in particular in geographically peripheral patterns (Fig 6). This is in stark contrast to the predicted evolutionary inertia of mimicry patterns due to strong stabilizing selection pressure exerted by mimicry selection [53]. We provide evidence that colour patterns are actively evolving under both classic and introgressed sweeps. Many of the detected sweep signatures are considerably younger than estimates of the age of colour pattern alleles based on phylogenetic patterns [36,39], suggesting ongoing improvement, innovation, and local switching between combinations of pattern elements. This is also consistent with observations of phenotypically distinct colour patterns restricted to the 5,000-year-old islands Ilha de Marajó in the South of Brazil and a few documented cases of rapid, local colour pattern turnover [114]. Therefore, our study offers a new perspective to the long-standing discussion of the paradox: How and why do new colour patterns arise? More generally, we here demonstrate that by considering selection across populations and species of an entire radiation, comparative information can capture spatial and temporal variability of genotypic selection and help to gain a more comprehensive understanding of the dynamics of adaptation in the wild.

## Methods

### Ethics statement

Panamanian specimens were collected under permit SE/AP-14-18 issued by the Ministerio de Ambiente de Panamá. Samples from Ecuador were collected with permission of the Ministerio del Ambiente under permits number 006-2012-IC-FAU-DPL-MA, 002-16-IC_FLO_FAU_DNB/MA, 033-10-IC_FAU/FLO_DPN/MA, and 0007-IC-FAU/FLO-DPPZ/MA. Colombian specimens were collected under the permit IDB0199/No16 and permit 530 granted to Universidad del Rosario by the Autoridad Nacional de Liencias Ambientales (ANLA-Colombia). Samples from Peru were collected under permit N°0148-2011-AG-DGFFS-DGEFFS and N°0236-2012-AG-DGFFS-DGEFFS from the Ministerio de la Agricultura, Peru. Samples from Suriname were collected and exported under a permit (No. 10865) from the Nature Conservation Division of the Suriname Forest Service. Field collections in Brazil were made under IBAMA/ICMBio license number 2024629 granted to GRPM. Recommendations of Animal Care and Use Committee (CEUA) of the Federal University of Rio Grande do Sul (UFRGS) were followed during laboratory procedures, including DNA extractions.

### Sampling and DNA extraction

Our sampling covers most of the distribution and colour pattern variation of the *Heliconius* radiation in South and Central America. Specimens were sampled or provided by collaborators with the respective sampling permissions and stored in salt saturated DMSO or ethanol at −20°C until further processing. For DNA extractions, thorax muscle tissue was dissected, disrupted and digested, and DNA was extracted using a TissueLyser II bead mill together with the DNeasy Blood and Tissue Kit (Qiagen, Hilden, Germany) following supplier recommendations.

### Targeted capture and sequencing

For hybridisation-based target enrichment a NimbleGen SeqCap EZ Library SR capture probes library was designed and synthesized by the provider (Roche NimbleGen Inc, United States). The templates for designing probes for 4 colour pattern regions (approximately 3.2 Mb) and 4 genomic background regions (approximately 2 Mb) were assembled and curated using the *H*. *melpomene* genome assembly Hmel1 [44], available BAC walks [31,115], fosmid data [69], and alignments from Wallbank and colleagues [36]. The neutral background regions were chosen to represent the average genome. We therefore excluded regions with extended stretches of extreme values for diversity and/or divergence, and we only considered regions located on a single, well-assembled scaffold.

Sample DNA was sheared with an ultrasonicator (Covaris Inc, Massachusetts, United States) and adapter-ligated libraries with insert sizes of 200 to 250 bp were generated using the Custom NEXTflex-96 Pre-Capture Combo Kit (Bioo Scientific Corporation, United States). For sequence capture, 24 libraries each were pooled into a capture library, hybridised with blocking oligos and the biotinylated capture library probes, and subsequently captured with streptavidin-coated magnetic capture beads using the NimbleGen SeqCap EZ Kits (Roche NimbleGen Inc, Wisconsin, United States). After capture and clean-up, 3 capture library pools were combined each. For the resulting sequencing pools of 72 samples, Illumina 100 or 150 bp paired-end short read data were generated on Illumina’s HiSeq 2000 (BGI, China) and HiSeq 4000 (Novogene Co. Ltd, China), respectively (S1 Table).

### Whole-genome data

Whole-genome resequencing data available for the *melpomene* clade from previously published work were also included [30,39,42,44,45,51,70–72]. For a few additional samples, 100 to 150 bp paired-end whole-genome resequencing data were generated on an Illumina X Ten platform (Novogene Co. Ltd, China; S1 Table). In addition, we downloaded, processed, and analysed a publicily available data set for *H*. *cydno galanthus* [49] with a more moderate depth of coverage (for results see S14 Fig). For the *erato* clade already published, whole-genome-resequencing data were used [38] (S10 Table).

The whole-genome data were mainly used for demographic reconstructions, whereas, for other analyses, the regions matching the capture regions were used.

### Genotyping

For *melpomene* clade data, sequenced reads were aligned to the *H*. *melpomene* version 2 reference genome (Hmel2, [137]), using BWA-mem version 0.7 [116]. PCR duplicated reads were removed using Picard version 2.2.4 (http://picard.sourceforge.net), and reads were sorted using SAMtools version 1.3.1 [117]. Genotypes for variant and invariant sites were called using the Genome Analysis Tool Kit’s (GATK) Haplotypecaller version 3.5 [118]. Individual genomic VCF records (gVCF) were jointly genotyped per population using GATK’s genotypeGVCFs version 3.5 [118]. Genotype calls were only considered in downstream analyses if they had a minimum depth (DP) ≥ 10, and for variant calls, a minimum genotype quality (GQ) ≥ 30, and indels were removed. Filtering was done with bcftools version 1.4 [117], and for downstream calculations of summary statistics and creating SF2 input, VCF files were parsed into tab delimited genotype files (scripts available at https://github.com/simonhmartin). For the *erato* clade, read data were mapped to the *H*. *erato demophoon* version 1 genome reference [38] and further processed as described above.

### Phasing

SHAPEIT2 [119] was used to phase haplotypes using both population information and paired read information. First, monomorphic and biallelic sites were filtered with GQ ≥ 30 and DP ≥ 10, and sites with less than 20% of sample genotypes were removed.

Next, phase informative reads (PIRs) with a minimum base-quality and read quality of 20 were extracted from individual BAM files using the extractPIRs tool. These BAM files were obtained from BWA-mem [116] mappings to the *H*. *melpomene* version 2 genome, with duplicates removed.

Finally, SHAPEIT2 was run with PIR information and default parameters on each scaffold using samples from single populations, which resulted in a haplotype file that was transformed into VCF format. Sites with no genotype information were imputed.

### Phylogenetic reconstruction

FastTree2 [120] was run using default parameters to infer approximate maximum likelihood phylogenies. Separate phylogenies for a concatenated SNP data set comprising neutral background regions only and for the full data set including the colour pattern regions to account for the effect of including regions putatively under strong selection were produced.

### Population historical demography

Changes in the historical population size were inferred from individual consensus whole-genome sequences (S3 Table) using Pairwise Sequentially Markovian Coalescent (PSMC’) analyses as implemented in MSMC [121]. This method fits a model of changing population size by estimating the distribution of times to the most recent common ancestor along diploid genomes. When used on single diploid genomes, this method is similar to PSMC analyses [122]. Genotypes were inferred from BWA version 0.7 [116] mapped reads separately from previous genotyping analysis using SAMtools version 0.1.19 [117]. This involved a minimum mapping (-q) and base (-Q) quality of 20 and adjustment of mapping quality (-C) 50. A mask file was generated for regions of the genome with a minimum coverage depth of 30× and was provided together with heterozygosity calls to the MSMC tool. MSMC was run on heterozygosity calls from all contiguous scaffolds longer than 500 kb, excluding scaffolds on the Z chromosome. We scaled the PSMC’ estimates using a generation time of 0.25 years and a mutation rate of 2×10^−9^ estimated for *H*. *melpomene* [47,77].

### SLiM simulations

Simulations were conducted to compare the genomic signatures of classical selective sweeps and sweeps that occur via adaptive introgression using SLiM (version 2) forward-in-time population simulation software [123,124]. Because SLiM tracks mutations and individuals through time, we were able to track individual beneficial alleles going to fixation and post sweep; however, it is computationally intractable to simulate very large populations with SLiM, and so we instead simulated smaller populations and rescaled population genetic parameters, *N* and *μ*, such that our results are applicable to *Heliconius* (as is commonly done [124,125]). Two populations of *N* = 1,000 were simulated with a neutral mutation rate *μ* of 6×10^−7^ such that the expected level of neutral diversity in the population was 0.0024, which is within an order of magnitude of that observed in our *Heliconius* populations [38,70] (S15–S18 Tables). Each individual in our simulated populations was represented by a single diploid recombining chromosome (recombination rate was also scaled such that *NR* is within the values of those observed in *Heliconius*, 4×10^−7^, or 40 cM/Mb) of length 750,000 bp. We also ran simulations on a shorter length of chromosome (50,000 bp) with an higher value of *μ*, raising levels of neutral diversity to those observed within *Heliconius*, to ensure our results are consistent for higher values of *μ*.

Our simulations were first allowed to equilibrate for a burn-in phase of 10*N* generations, after which we introduced a single strongly advantageous mutation of *s* = 0.5 in the centre of the chromosome in order to simulate a ‘classical’ hard selective sweep in the population (which we will refer to as p1). We also ran our simulations with 2 lower values of *s* (*s* = 0.1 and *s* = 0.25). Only those simulations in which the mutation went to fixation were kept; if the beneficial mutation was lost during the course of a simulation, the simulation was reset to a point just after the burn-in phase and the mutation was reintroduced. The simulations were then allowed to run for a further 5*N* generations. During this time, p1 does not experience any migration or population size change. In order to simulate an introgressed sweep, we simulated an additional neutrally evolving population, p2, which exchanges migrants with population p1 at a constant rate of 0.0001 migrants per generation, which allowed the beneficial mutation fixed in p1 to introgress into p2. The simulations were then allowed to run for a further 10*N* generations with a constant migration rate. For each set of parameters, we ran our simulations 100 times.

For both populations, a complete sample of the segregating neutral mutations was taken every 100 generations after the burn-in phase and prior to the introduction of the beneficial mutation, and every 50 generations after the introduction of the beneficial mutation. We also tracked the change in frequency over time of the beneficial mutation during the simulations. From these results we calculated 2 summary statistics, Tajima’s *D* and *π*, in windows of 10,000 bp across our simulated chromosomes for a range of time points. Time points are as follows, in 4*N* generations post sweep: 0.01, 0.1, 0.2, 0.3, 0.4, 0.5, 0.6, 0.7, 0.8, 1, and 2 background rates: one post burn-in, during which populations are not experiencing any migration, and one post sweep, during which the populations are exchanging migrants. Values were then averaged across simulations. Additionally, to model the effect of changing effective migration rates on the introgression sweep signal, we ran simulations with different levels of migration, using the following 4 values of *M*: 200, 2, 0.2, and 0.02, with recombination rate = 4cM/Mb and *s* = 0.1. The simulations were otherwise set up as before, with 30 simulation runs generated for each set of parameters.

We used these results to generate SF2 [76] input files, after first subsampling the number of mutations down, such that our simulated SF2 files for each population represent a sample of 500 simulated individuals. This step is necessary because SweepFinder has an upper limit on the number of sequences that can be included per sample [126]. We then ran SF2 using mode–lg 100 for each simulation for each of the time points, using 1 of 2 precomputed site frequency spectra as appropriate: one calculated across multiple neutral simulations without migration and one calculated across multiple neutral simulations with migration (these neutral simulations correspond to the 2 background rates described above). Further details of SF2 and its various run modes are included in the ‘SF2’ section.

### Phylogenetic weighting

A phylogenetic weighting approach was used to evaluate the support for alternative phylogenetic hypotheses across colour pattern loci using *Twisst* [127]. Given a tree and a set of predefined groups, in this case *Heliconius* populations sharing specific colour pattern elements, *Twisst* determines a weighting for each possible topology describing the relationship of the groups. The weightings thus represent to what extent loci cluster according to phenotype, rather than geographic relatedness of populations. Topology weightings are determined by sampling a single member of each group and identifying the topology matched by the resulting subtree. This process is iterated over a large number of subtrees, and weightings are calculated as the frequency of occurrence of each topology. Weightings were estimated from 1,000 sampling iterations over trees produced by RAxML version 8.0.2681 [128] for 50 SNP windows with a stepping size of 20 SNPs. For phylogenetic weighting along the *WntA* interval, weightings of topologies that grouped populations with the split forewing band phenotype or, alternatively, the hourglass shape were assessed (S7 Fig). For the region containing the *aristaless* genes, we focussed on topologies that clustered populations with white or yellow colour phenotypes (S8 Fig). For the *cortex* region, we focussed on topologies grouping populations showing the ventral and dorsal yellow hindwing bar, respectively (S9 Fig). Finally, for the *optix* interval, we assessed topologies grouping populations according to the absence or presence of the red dennis patch, the red hindwing rays, or the red forewing band and repeated the analysis for different geographic settings (S10 Fig). To obtain weightings for hypothesized phylogenetic groupings of specific colour pattern forms, we summed the counts of all topologies that were consistent with the hypothesized grouping.

### Inference of selection and summary statistics in sliding windows

Summary statistics informative on diversity and selection patterns were calculated. From the unphased data, nucleotide diversity, Kelly’s *Z*_*nS*_, Tajima’s *D*, and number of sites genotyped for each population were calculated in 1 kb nonoverlapping sliding windows with at least 100 sites genotyped for at least 75% of all individuals within that population using custom python scripts and the EggLib library version 3[129]. Scans for selection using signals of extended haploptype homozygosity and calculation of the pooled integrated haplotype homozygosity score (iHH12) [11,130] were performed using the program selscan1.2 [131] and our phased data set.

### SF2

To detect local distortions of the SFS that are indicative of selective sweeps, SF2, an extension of Nielsen and colleagues’ [73] SweepFinder program, with increased sensitivity and robustness [74,76] was used. The SweepFinder framework builds on a composite likelihood ratio test using the SFS to compare the likelihood for a model with a selective sweep versus the likelihood for a model without a sweep. Huber and colleagues [74] showed that including substitutions, i.e., fixed differences relative to an outgroup, increases power while maintaining robustness to variation in mutation rate. SF2 also permits the use of recombination maps. The use of polarised sites increases power and we therefore polarised sites when possible.

We filtered our data set for biallelic sites only and initially tested different input data sets and parameter settings and created 2 types of data sets for this purpose; one using polymorphic sites only with both polarised and unpolarised sites and one with polymorphic sites and substitutions that contained only polarised sites. As an outgroup, *H*. *numata* was used for the *melpomene* clade and *H*. *hermathena* for the *erato* clade. We used biallelic sites only that were present in ≥75% of the focal populations and polarised sites by randomly drawing an outgroup allele from sites with a minimum number of outgroup samples with genotype data of either one (−OM1) or 3 (−OM3) of 4 for the *melpomene* clade and one (−OM1) or 2 (−OM2) of 3 for the *erato* clade.

SF2 was then run in 2 modes for each data set: with flag -s, calculating the likelihoods from the SFS of the respective region and with flag -l, using a SFS precalculated either from the background regions only or from background regions and colour pattern regions combined. These precalculated SFSs are used by SF2 as null models that incorporate the underlying demography of the populations of interest, making SF2 sensitive to selective sweeps even in populations that are not at equilibrium [132]. For the *melpomene* clade, recombination rate information from a fine scale recombination map was included (flag -r) [133]. To create a recombination file, recombination map coordinates were transferred to Hmel2 coordinates, and between sites recombination rates were calculated.

SF2 test runs for different grid spaces (flag–g; tested values: −g1, −g5, −g50, −g100, −g1000) were performed to find a setting allowing for reasonable runtimes without loss of accuracy and based on these test CLR and α were calculated for every 50th site (−g50) across all populations and regions.

Generally, the results were largely consistent among the different runs and data sets. As expected, power to detect sweeps was higher when including substitutions [74], and the minimum number of outgroup samples had only marginal effects. We therefore focussed on the results for data sets with outgroup minimum 1 (−OM1) and background SFS calculated from background regions and background regions and colour pattern regions combined, respectively. Including the colour pattern regions inflates the estimated background SFS with regions affected by selective sweeps which results in slightly lower CLR and higher α estimates. Because selective sweeps across the genome have been found to be rare in *H*. *melpomene* [70], these estimates represent a lower bound, and the estimates derived with background SFS from the background regions only are most likely a better approximation. Only CLR peaks exceeding a threshold defined as the 99.9th percentile of the distribution of CLR values across all background regions were considered as evidence for selection.

To obtain estimates for strength of selection (s) we calculated *s* as *s* = *r*×ln(2*N_e_*)/*α* [132,134] with region- and population-specific estimates of effective population size (*N*_*e*_) estimated from the data using the mutation rate given in Keightley and colleagues [77] and per chromosome recombination rate estimates (r) from Davey and colleagues [133] and Van Belleghem and colleagues [38].

### VolcanoFinder

We also tested the new software VolcanoFinder on our data, described in a recent preprint, which is specifically designed to detect introgression sweeps but can also detect classic sweeps [27]. As for the SF2 runs, we used data sets with outgroup minimum 1 (−OM1) and background SFS calculated from background regions to generate the allele frequency files and the required unnormalized site frequency spectrum. We then ran VolcanoFinder with the following specifications: Model 1 and *P* = 0.

## Supporting information

S1 Fig Phylogenetic reconstruction of the *H*. *melpomene* clade. Phylogenetic reconstruction for *H*. *melpomene* clade samples used in this study including all sequenced region, i.e., colour pattern regions and neutral background regions. *H*. *cydno* (green) and *H*. *timareta* (blue) cluster together and form a sister clade to *H*. *melpomene* (red). The ‘silvaniforms’ outgroup is shown in orange. A high-resolution version can be found here: https://github.com/markusmoest/SelectionHeliconius.git.
(PNG)

S2 Fig Distributional ranges as obtained from Rosser and colleagues [136] and samples localities of this study. Colour coding representing populations corresponds to colour coding in Fig 1A in the main text.
(PNG)

S3 Fig Demographic history of *H*. *melpomene* clade populations. Demographic histories for populations in the *H*. *melpomene* clade for which whole-genome data were available reconstructed with PSMC’ [121]. Additional demographic histories for *Heliconius* species considered in this study are already published [38]. PMSC’, Pairwise Sequentially Markovian Coalescent.
(PNG)

S4 Fig CLR statistic (SF2 [74,76]), over time at 3 positions relative to the sweep centre. Plotted is the CLR statistic over time at 3 chromosome positions relative to the sweep centre, which correspond to the sweep site itself (dark blue), 0.02 Mb from the sweep (mid blue), and 0.04 Mb from the sweep (light blue), for 4 different simulation parameters. Selection coefficient, *s* = 0.25, neutral mutation rate, *μ* = 6e-07 corresponds to Fig 2, with average SF2 values calculated over 100 simulation runs, along with their standard errors. We also explored changes in s and μ in our simulations. Averages over 20 simulation runs are shown, along with their standard errors. Time is given in units of scaled generations. CLR, composite likelihood ratio; SF2, SweepFinder2
(PNG)

S5 Fig Effect of effective migration rate on introgressed sweep signatures. SFS signatures of simulated introgressed sweeps across a chromosome for different time points summarised as Tajima’s *D* statistics. The sweep occurs in the centre of the simulated chromosome. Different colours indicate patterns at different time points since sweep (0.01, 0.1, 0.5, 0.8, and 1 scaled generations, i.e., 4*N* generations). Simulated data for 4 different effective migration rates are shown (*M* = 200, 2, 0.2, and 0.002). SFS, site frequency spectrum
(PNG)

S6 Fig Signatures of selection across neutral background regions in the *H*. *melpomene* clade. Genes are annotated in the top gene annotation panel. On the y-axis SF2’s [74,76] CLR statistics is shown (peaks are capped at CLR = 1,000). The colour gradient indicates estimated intensity of selection (black = high *α* values, weak selection; red = low *α* values, strong selection). Blue horizontal bars indicate regions with CLR values above threshold. CLR, composite likelihood ratio; SF2, SweepFinder2
(PNG)

S7 Fig Tree weighting (Twisst [127]) analysis of the *WntA* gene region. Topology weightings for topologies clustering the split forewing band phenotype (magenta) and the hourglass shape phenotype (blue) are shown. (ama = *H*. *m*. *amaryllis*, ecu = *H*. *m*. *ecuadoriensis*, ple = *H*. *m*. *plesseni*, xen = *H*. *m*. *xenoclea*, cyd = *H*. *cydnides*, wey = *H*. *c*. *weymeri f*. *weymeri*, gus = *H*. *c*. *weymeri f*. *gustavi*, zel = *H*. *c*. *zelinde*).
(PNG)

S8 Fig Tree weighting (Twisst [127]) analysis of the *aristaless* genes region. Topology weightings for topologies clustering the white (chi = *H*. *c*. *chioneus*, zel = *H*. *c*. *zelinde*) and yellow (ecu = *H*. *m*. *ecuadoriensis*, ple = *H*. *m*. *plesseni*, heu = *H*. *heurippa*, flo = *H*. *t*. *florencia*, cyd = *H*. *cydnides*, pac = *H*. *pachinus*) colour phenotypes (magenta) are shown.
(PNG)

S9 Fig Tree weighting (Twisst [127]) analysis of the *cortex* gene regions. Topology weightings for topologies clustering the dorsal yellow hindwing bar (magenta) and ventral yellow hindwing bar (blue) phenotypes are shown (cyt = *H*. *m*. *cythera*, bur = *H*. *m burchelli*, nan = *H*. *m*. *nanna*, ros = *H*. *m*. *rosina*, vul = *H*. *m*. vulcanus, chi = *H*. *c*. *chioneus*, wey = *H*. *c*. *weymeri f*. *weymeri*, gus *= H*. *c*. *weymeri f*. *gustavi*, zel = *H*. *c*. *zelinde*, pac = *H*. *pachinus*).
(PNG)

S10 Fig Tree weighting (Twisst [127]) analysis of the *optix* gene regions. Topology weightings for topologies clustering the dennis (magenta), rays (blue), and band (brown) phenotypes. Including different red banded populations shows different phylogenetic clustering and thus potentially a different genetic basis underlying this trait among populations. (A) Tree weighting including the Peruvian red banded population *H*. *t*. *thelxinoe*. (B) Tree weighting including red banded populations from East Brazil, *H*. *m*. *burchelli*, *H*. *m*. *nanna* and *H*. *besckei*. (bur = *H*. *m burchelli*, malE = *H*. *m*. *malleti* (ECU), melG = *H*. *m*, *melpomene* (FG), mer = *H*. *m*. *meriana*, nan = *H*. *m*. *nanna*, ros = *H*. *m*. *rosina*, vul = *H*. *m*. vulcanus, heu = *H*. *heurippa*, flo = *H*. *t*. *florencia*, lin = *H*. *t*. *linaresi*, the = *H*. *t*. *thelxinoe*, tim = *H*. *t*. *timareta f*. *timareta*, con = *H*. *t*. *timareta f*. *contigua*, ele = *H*. *elevatus*, bes = *H*. *besckei*, silvana = *H*. *numata silvana*).
(PNG)

S11 Fig Summary and selection statistics across colour pattern regions for all populations analysed in the *H*. *melpomene* clade. For each population genotyping coverage (calculated as proportion of retained genotypes after quality filtering in 500 bp windows), nucleotide diversity, Kelly’s *Z*_*nS*_, Tajima’s *D*, pooled integrated haplotype homozygosity score, and SweepFinder2’s [74,76] composite likelihood ratio statistics across each colour pattern region are shown (top to bottom). File names contain population and colour pattern region identifiers (Hmel201011 = *aristaless* scaffold, Hmel210004 = *WntA* scaffold, Hmel215006 = *cortex* scaffold, Hmel218003 = *optix* scaffold). The 120 single figures have been uploaded to GitHub:https://github.com/markusmoest/SelectionHeliconius/tree/master/S11_Fig_H_melpomene.
(PDF)

S12 Fig Correlation between portion of genomic loci under selection and geographic range of co-mimicking *H*. *melpomene* (above) and *H*. *erato* (below) races. Portion of genomic loci under selection is summarised as percentage of CLR values across the colour pattern region which are above the CLR threshold [%CLR>th] scaled by the maximum value for *WntA*, *cortex*, and *optix* regions. Areas were calculated from distribution data obtained from [136] using an alpha hull polygon (code available at https://github.com/StevenVB12/Sample-distributions).
(PNG)

S13 Fig Correlation between maximum intensity of selection [max(1/α)] and geographic range of co-mimicking *H*. *melpomene* (above) and *H*. *erato* (below) races. Areas were calculated from distribution data obtained from Rosser and colleagues [136] using a alpha hull polygon (code available at https://github.com/StevenVB12/Sample-distributions).
(PNG)

S14 Fig Additional SweepFinder2 [74,76] and VolcanoFinder [27] analyses of publicly available data for *H*. *c*. *galanthus* [49]. The regions containing the tandem copies of *aristaless*, *al1* and *al2*, *WntA*, *cortex*, and *optix* (left to right) are depicted. Colour pattern genes are annotated in red in the gene annotation panel. On the y-axis Sweepfinder2’s and VolcanoFinder’s CLR statistics are shown (peaks capped at 1,000). The colour gradient indicates the estimated intensity of selection *α* (black…high *α* values, weak selection; red…low *α* values, strong selection). Grey shadings indicate annotated colour pattern CREs [28,30,36,37,39] (S7–S10 Figs). Coloured horizontal bars indicate regions with CLR values above threshold and for VolcanoFinder results, the colour gradient indicates the estimated *D* value. Top panel shows colour pattern phenotypes, and symbols indicate distinct colour pattern elements and their presence is annotated in population panels. Note that the yellow hindwing bar controlled by the *cortex* region can be expressed on the dorsal and ventral side (yellow/yellow square symbol) or on the ventral side only (black/yellow square symbol) [39]. Moreover, the actual shape of the forewing band can depend on the allelic state of *WntA*. Full, grey lines connect colour pattern elements with annotated CREs. The *H*. *c*. *galanthus* phenotype is depicted on the right. CLR, composite likelihood ratio; CRE, *cis-*regulatory element.
(PNG)

S15 Fig VolcanoFinder [27] scans across colour pattern regions in the *H*. *melpomene* clade. The regions containing the tandem copies of *aristaless*, *al1* and *al2*, *WntA*, *cortex*, and *optix* (left to right) are depicted. Colour pattern genes are annotated in red in the gene annotation panel. On the y-axis VolcanoFinder’s CLR statistics is shown (peaks capped at 1,000). The colour gradient indicates the estimated intensity of selection *α* (black…high *α* values, weak selection; red…low *α* values, strong selection). Grey shadings indicate annotated colour pattern CREs [28,30,36,37,39] (S7–S10 Figs). Coloured horizontal bars indicate regions with CLR values above threshold and the colour gradient indicates the estimated *D* value. Top panel shows colour pattern phenotypes and symbols indicate distinct colour pattern elements and their presence is annotated in population panels. Note that the yellow hindwing bar controlled by the *cortex* region can be expressed on the dorsal and ventral side (yellow/yellow square symbol) or on the ventral side only (black/yellow square symbol) [39]. Moreover, the actual shape of the forewing band can depend on the allelic state of *WntA*. Full, grey lines connect colour pattern elements with annotated CREs. CLR, composite likelihood ratio; CRE, *cis*-regulatory element.
(PNG)

S16 Fig VolcanoFinder [27] scans across neutral background regions in the *H*. *melpomene* clade. Genes are annotated in in the top gene annotation panel. On the y-axis VolcanoFinder’s CLR statistics is shown (peaks are capped at 1,000). The colour gradient indicates the estimated intensity of selection *α* (black…high *α* values, weak selection; red…low *α* values, strong selection). Coloured horizontal bars indicate regions with CLR values above threshold and the colour gradient indicates the estimated *D* value. CLR, composite likelihood ratio
(PNG)

S17 Fig Superposition of SweepFinder2’s [74,76] composite likelihood ratio peaks of all *H*. *melpomene* clade populations for each of the 4 colour pattern regions. Superimposed, semitransparent SweepFinder2 peaks are depicted in grey. Colour pattern genes (yellow), known CREs (red), and additional genes with evidence for a putative role in colour patterning (blue and green for genes discussed in the main text) are highlighted and assigned a number in the top row. The scale on the x-axes differs and the y-axis is capped at CLR = 1,500. (A) *aristaless1* (yellow, 2), *aristaless1* CRE (red, 3) [28], *aristaless2* (blue, 1); (B) *WntA* (yellow, 4), CRE associated with split forewing band identified in this study (red, 5); (C) *cortex* (yellow, 10), CREs for dorsal (11) and ventral (12) hindwing topology [39], a region containing SNPs with strongest association with forewing band [30] (13) (red), additional genes with evidence for wing patterning control [30] (blue: 7, 8, 9, 14, 15, 16, 18, 19, 21, 22, 23; green: 17 (*LMTK1* /HM00033), 20 (*washout/WAS* homologue *1*/HM00036); also see S9 Table); (D) *optix* (yellow, 23), CREs for ‘band1’(24), ‘band2’(26), ‘rays’(25) and ‘dennis’(27) (red) [36,37], *kinesin* (green, 28) [86,87]. A genome viewer in which these regions and accession can be viewed in detail is available at http://lepbase.org/. CLR, composite likelihood ratio; CRE, *cis-*regulatory element; SNP, single-nucleotide polymorphism
(PNG)

S18 Fig Superposition of SweepFinder2 [74,76] composite likelihood ratio peaks of all *H*. *erato* clade populations for each of the 4 colour pattern regions. Superimposed, semitransparent SweepFinder2 peaks are depicted in grey. Colour pattern genes (yellow), known CREs (red), and additional genes with evidence for a putative role in colour patterning (blue and green for genes discussed in the main text) are highlighted and assigned a number in the top row. The scale on the x-axes differs and the y-axis is capped at CLR = 1,500. (A) *WntA* (yellow,1), CREs associated with ‘Sd1’(2), ‘Sd2’(3), ‘St’(4), ‘Ly1’(5) and ‘Ly2’(6) elements (red); (B) *cortex* (yellow, 8), ‘Cr1’(7) and ‘Cr2’(9) regions (red) [38], and additional genes with evidence for wing patterning control [30] (blue: 10,12; green; 11 (*washout*/*WAS homologue 1*/HERA000061), 13 (*lethal (2)*/HERA000062); also see S9 Table; (C) *optix* (yellow,14), CREs for ‘rays’(15), ‘band’ Y1(16)/ Y2(18), and ‘dennis’ D1(17)/ D2(19) elements (red) [38]. A genome viewer in which these regions and accession can be viewed in detail is available at http://lepbase.org/. CLR, composite likelihood ratio; CRE, colour pattern regulatory element.
(PNG)

S19 Fig Superposition of VolcanoFinder2’s [27] composite likelihood ratio peaks of all *H*. *melpomene* clade populations for each of the 4 colour pattern regions. Superimposed, semitransparent VolcanoFinder2 peaks are depicted in grey. Colour pattern genes (yellow), known CREs (red), and additional genes with evidence for a putative role in colour patterning (blue and green for genes discussed in the main text) are highlighted and assigned a number in the top row. The scale on the x-axes differs and the y-axis is capped at CLR = 2,000. (A) *aristaless1* (yellow, 2), *aristaless1* CRE (red, 3) [28], *aristaless2* (blue, 1); (B) *WntA* (yellow, 4), CRE associated with split forewing band identified in this study (red, 5); (C) *cortex* (yellow, 10), CREs for dorsal (11) and ventral (12) hindwing topology [39], a region containing SNPs with strongest association with forewing band [30] (13) (red), additional genes with evidence for wing patterning control [30] (blue: 7, 8, 9, 14, 15, 16, 18, 19, 21, 22, 23; green: 17 (*LMTK1* /HM00033), 20 (*washout/WAS* homologue *1*/HM00036); also see S9 Table); (D) *optix* (yellow, 23), CREs for ‘band1’(24), ‘band2’(26), ‘rays’(25) and ‘dennis’(27) (red) [36,37], *kinesin* (green, 28) [86,87]. A genome viewer in which these regions and accession can be viewed in detail is available at http://lepbase.org/. CLR, composite likelihood ratio; CRE, *cis-*regulatory element.
(PNG)

S20 Fig Signature of selection across colour pattern regions in the *H*. *erato* clade. The regions containing *WntA*, *cortex*, and *optix* (left to right) are depicted. Colour pattern genes are annotated in red in the gene annotation panel. On the y-axis Sweepfinder2’s [74,76] CLR statistics is shown (peaks are capped at CLR = 1,000). The colour gradient indicates the estimated intensity of selection (black = high *α* values, weak selection; red = low *α* values, strong selection). Blue horizontal bars indicate regions above the CLR threshold value. CLR, composite likelihood ratio
(PNG)

S21 Fig Signature of selection across neutral background regions in the *H*. *erato* clade. Genes are annotated in the top gene annotation panel. On the y-axis Sweepfinder2’s [74,76] CLR statistics is shown (peaks are capped at 1,000). The colour gradient indicates the estimated intensity of selection (black = high *α* values, weak selection; red = low *α* values, strong selection). Blue horizontal bars indicate regions above the CLR threshold value. CLR, composite likelihood ratio
(PNG)

S22 Fig Summary and selection statistics across colour pattern regions for all populations analysed in the *Heliconius erato* clade. For each population genotyping coverage (calculated as proportion of retained genotypes after quality filtering in 500 bp windows), nucleotide diversity, Kelly’s *Z*_*nS*_, Tajima’s *D*, pooled integrated haplotype homozygosity score, and SweepFinder2’s [74,76] CLR statistics across each colour pattern region are shown (top to bottom). File names contain population and colour pattern region identifiers (Herato1001 = *WntA* scaffold, Herato1505 = *cortex* scaffold, Herato1801 = *optix* scaffold). The 18 single figures have been uploaded to GitHub:https://github.com/markusmoest/SelectionHeliconius/tree/master/S22_Fig_H_erato. CLR, composite likelihood ratio.
(PDF)

S1 Table Sample information and genotyping statistics for all samples from the *H*. *melpomene* clade. (PDF)

S2 Table Per-population sample sizes for the *H*. *melpomene* clade and the *H*. *erato* clade used in the respective analyses. (PDF)

S3 Table Sample information for whole-genome sequence data used for PSMC’ analysis. PSMC’, Pairwise Sequentially Markovian Coalescent.
(PDF)

S4 Table Average neutral equilibrium values of nucleotide site diversity (*pi*) Tajima’s *D* and Kelly’s *Z_nS_* for our simulated populations, both without migration (i.e., the simulated population at equilibrium prior to experiencing a classic sweep) and with migration (i.e., the simulated population at equilibrium after an introgressed sweep). Values are labelled by the parameter values of the simulations from which they were generated (*s* = selection coefficient; *μ* = mutation rate per base pair/generation).
(PDF)

S5 Table Position, CLR statistics and strength of selection (*α*, 2*N_e_s*, and *s*) for the highest CLR and the smallest *α* value on each colour pattern scaffold (*α_min_*) for the *H*. *melpomene* clade. Additional relevant peaks on scaffolds are also given. Data are from SweepFinder2 [74,76] runs with background SFS estimated from background scaffolds. CLR, composite likelihood ratio; SFS, site frequency spectrum.
(PDF)

S6 Table Position, CLR statistics and strength of selection (*α*, 2*N_e_s*, and *s*) for the highest CLR and the smallest *α* value on each background scaffold (*α_min_*) for the *H*. *melpomene* clade. Data are from SweepFinder2 [74,76] runs with background SFS estimated from background scaffolds. CLR, composite likelihood ratio; SFS, site frequency spectrum.
(PDF)

S7 Table Position, CLR statistics and strength of selection (*α*, 2*N_e_s*, and *s*) for the highest CLR and the smallest *α* value on each colour pattern scaffold (*α_min_*) for the *H*. *melpomene* clade. Additional relevant peaks on scaffolds are also given. Data are from SweepFinder2 [74,76] runs with background SFS estimated from background and colour pattern scaffolds. CLR, composite likelihood ratio; SFS, site frequency spectrum.
(PDF)

S8 Table Position, CLR statistics and strength of selection (*α*, 2*N_e_s*, and *s*) for the highest CLR and the smallest *α* value on each background scaffold (*α_min_*) for the *H*. *melpomene* clade. Data are from SweepFinder2 [74,76] runs with background SFS estimated from background and colour pattern scaffolds. CLR, composite likelihood ratio; SFS, site frequency spectrum.
(PDF)

S9 Table List of additional genes with significant colour pattern associations on the cortex scaffold from Nadeau and colleagues [30] that overlap with or are in proximity of selection signatures detected in this study. (PDF)

S10 Table Sample information and genotyping statistics for all samples from the *H*. *erato* clade from Van Belleghem and colleagues [38]. (PDF)

S11 Table Position, CLR statistics and strength of selection (*α*, 2*N_e_s*, and *s*) for the highest CLR and the smallest *α* value on each colour pattern scaffold (*α_min_*) for *H*. *erato*. Additional relevant peaks on scaffolds are also given. Data are from SweepFinder2 [74,76] runs with background SFS estimated from background scaffolds. CLR, composite likelihood ratio; SFS, site frequency spectrum.
(PDF)

S12 Table Position, CLR statistics and strength of selection (*α*, 2*N_e_s*, and *s*) for the highest CLR and the smallest *α* value on each background scaffold (*α_min_*) for *H*. *erato*. Data are from SweepFinder2 [74,76] runs with background SFS estimated from background scaffolds. CLR, composite likelihood ratio; SFS, site frequency spectrum
(PDF)

S13 Table Position, CLR statistics and strength of selection (*α*, 2*N_e_s*, and *s*) for the highest CLR and the smallest *α* value on each colour pattern scaffold (*α_min_*) for *H*. *erato*. Additional relevant peaks on scaffolds are also given. Data are from SweepFinder2 [74,76] runs with background SFS estimated from background and colour pattern scaffolds. CLR, composite likelihood ratio; SFS, site frequency spectrum.
(PDF)

S14 Table Position, CLR statistics and strength of selection (*α*, 2*N_e_s*, and *s*) for the highest CLR and the smallest *α* value on each background scaffold (*α_min_*) for *H*. *erato*. Data are from SweepFinder2 [74,76] runs with background SFS estimated from background and colour pattern scaffolds. CLR, composite likelihood ratio; SFS, site frequency spectrum.
(PDF)

S15 Table Per-population and per-scaffold summary statistics estimates and standard deviation for colour pattern scaffolds in the *H*. *melpomene* clade. (PDF)

S16 Table Per-population and per-scaffold summary statistics estimates and standard deviation for neutral background scaffolds in the *H*. *melpomene* clade. (PDF)

S17 Table Per-population and per-scaffold summary statistics estimates and standard deviation for colour pattern scaffolds in the *H*. *erato* clade. (PDF)

S18 Table Per-population and per-scaffold summary statistics estimates and standard deviation for neutral background scaffolds in the *H*. *erato* clade. (PDF)

## Abbreviations

CLR: composite likelihood ratio
CRE: *cis*-regulatory element
iHH12: integrated haplotype homozygosity score
JAK/STAT: Janus kinase/signal transducer and activator of transcription
Mya: million years ago
PIR: phase informative read
PSMC: Pairwise Sequentially Markovian Coalescent
SFS: site frequency spectrum
SF2: SweepFinder2
