# Supplementary figures and images for: Selective sweeps on novel and introgressed variation shape mimicry loci in a butterfly adaptive radiation
Source: PLoS Biol. 2020 Feb 6;18(2):e3000597. doi: 10.1371/journal.pbio.3000597 (PMC7029882; doi:10.1371/journal.pbio.3000597)

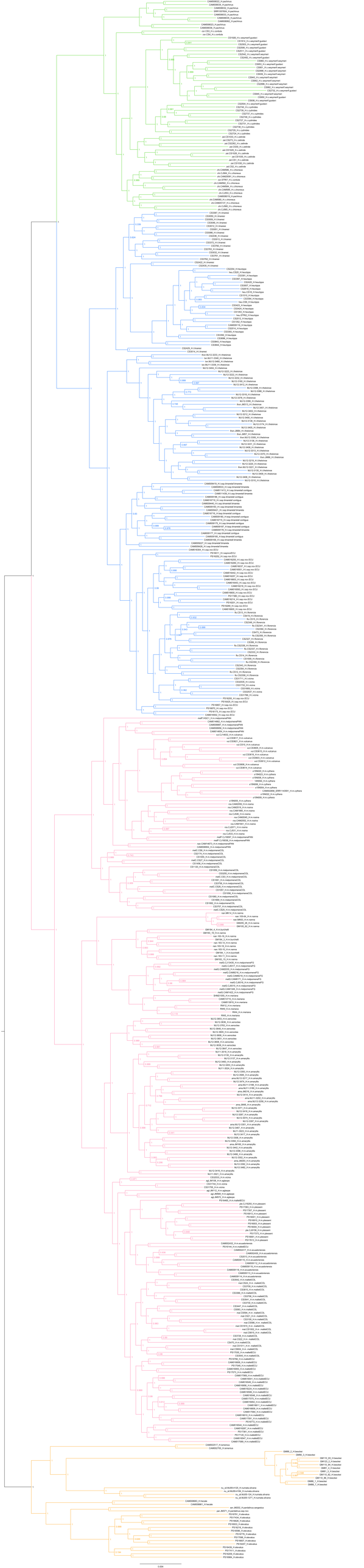

Supplement: S1 Fig — Phylogenetic reconstruction for H. melpomene clade samples used in this study including all sequenced region, i.e., colour pattern regions and neutral background regions. H. cydno (green) and H. timareta (blue) cluster together and form a sister clade to H. melpomene (red). The ‘silvaniforms’ outgroup is shown in orange. A high-resolution version can be found here: https://github.com/markusmoest/SelectionHeliconius.git. (PNG) [file pbio.3000597.s001.png]

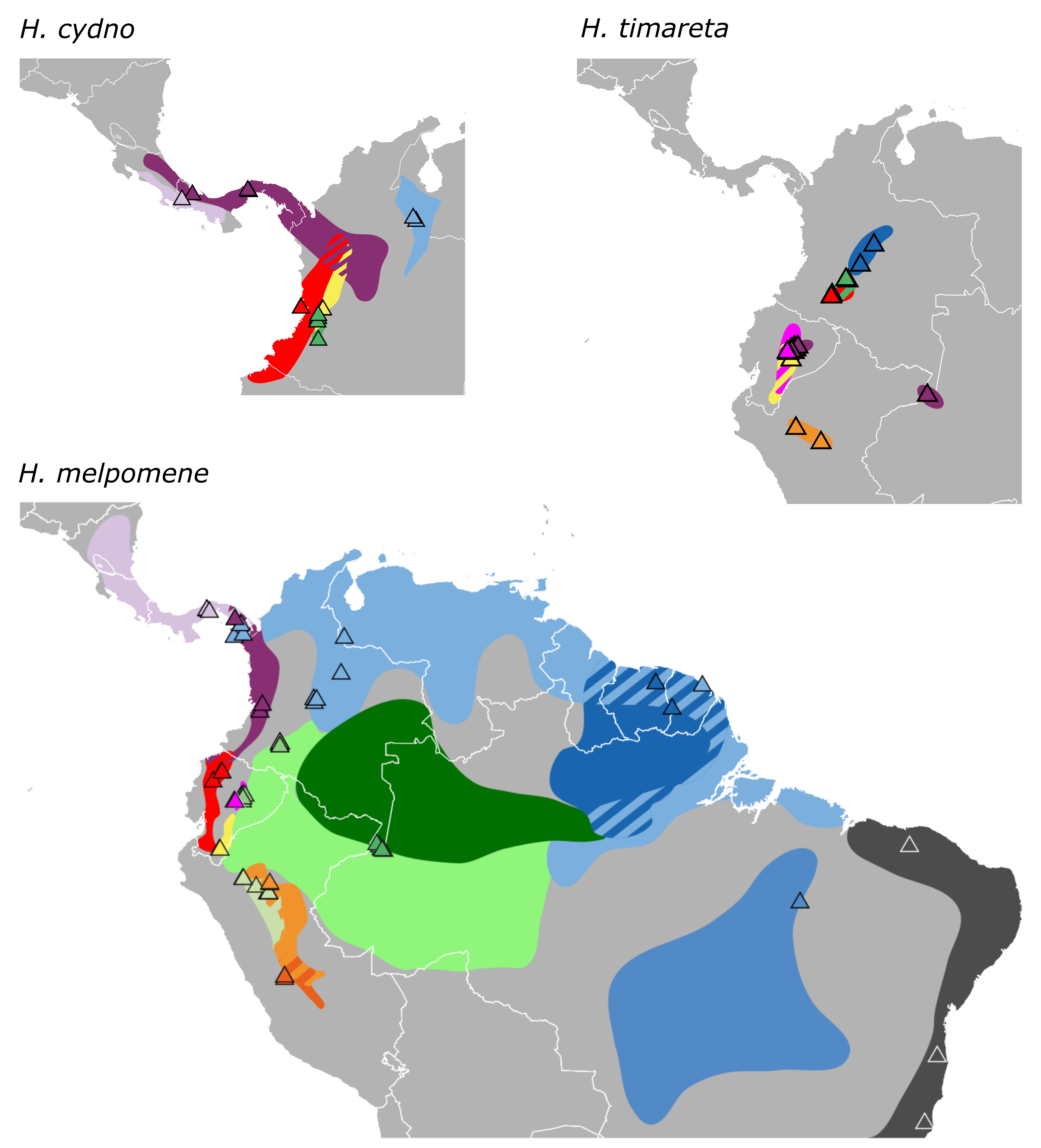

Supplement: S2 Fig — Colour coding representing populations corresponds to colour coding in Fig 1A in the main text. (PNG) [file pbio.3000597.s002.png]

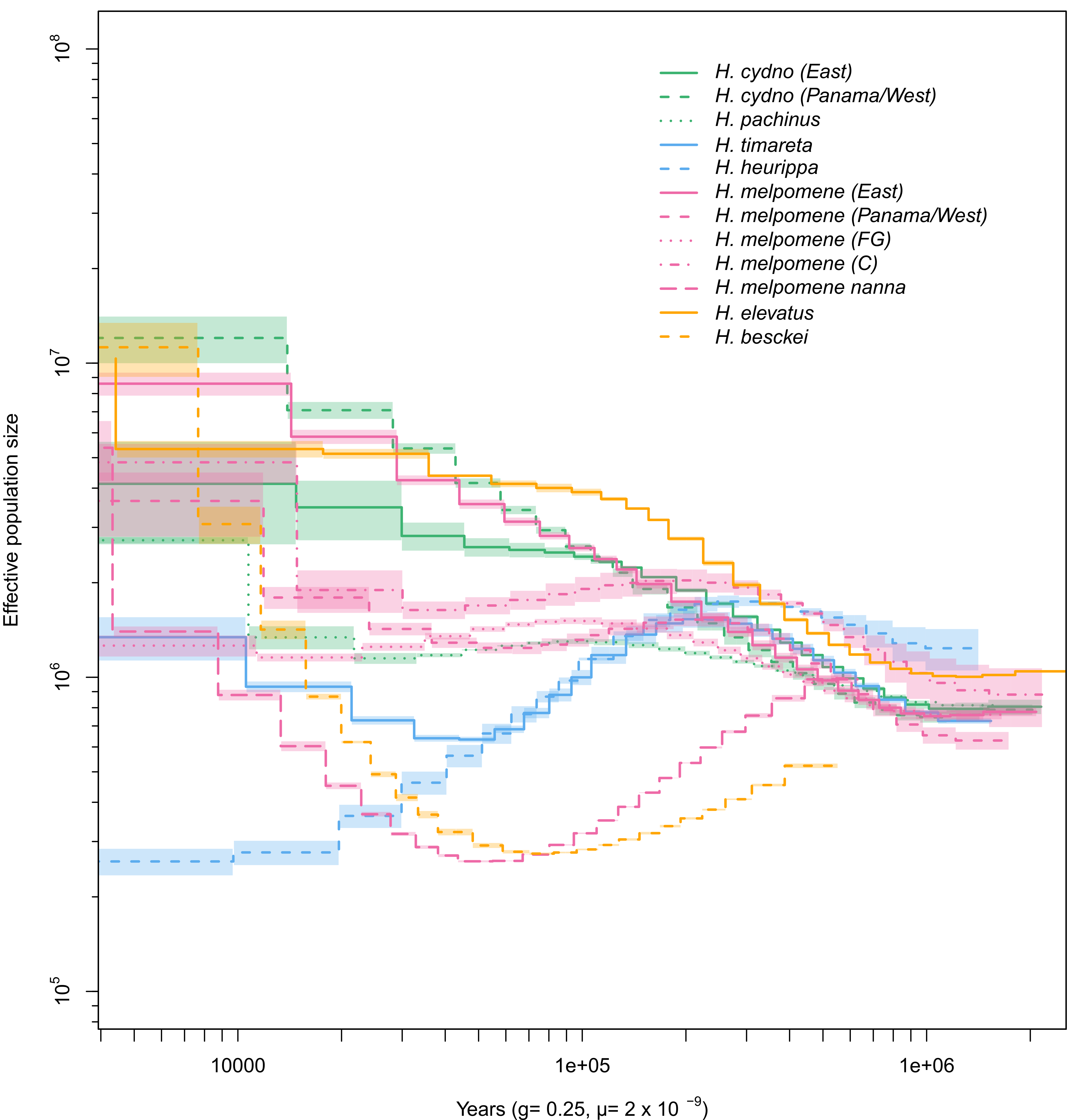

Supplement: S3 Fig — Demographic histories for populations in the H. melpomene clade for which whole-genome data were available reconstructed with PSMC’ [121]. Additional demographic histories for Heliconius species considered in this study are already published [38]. PMSC’, Pairwise Sequentially Markovian Coalescent. (PNG) [file pbio.3000597.s003.png]

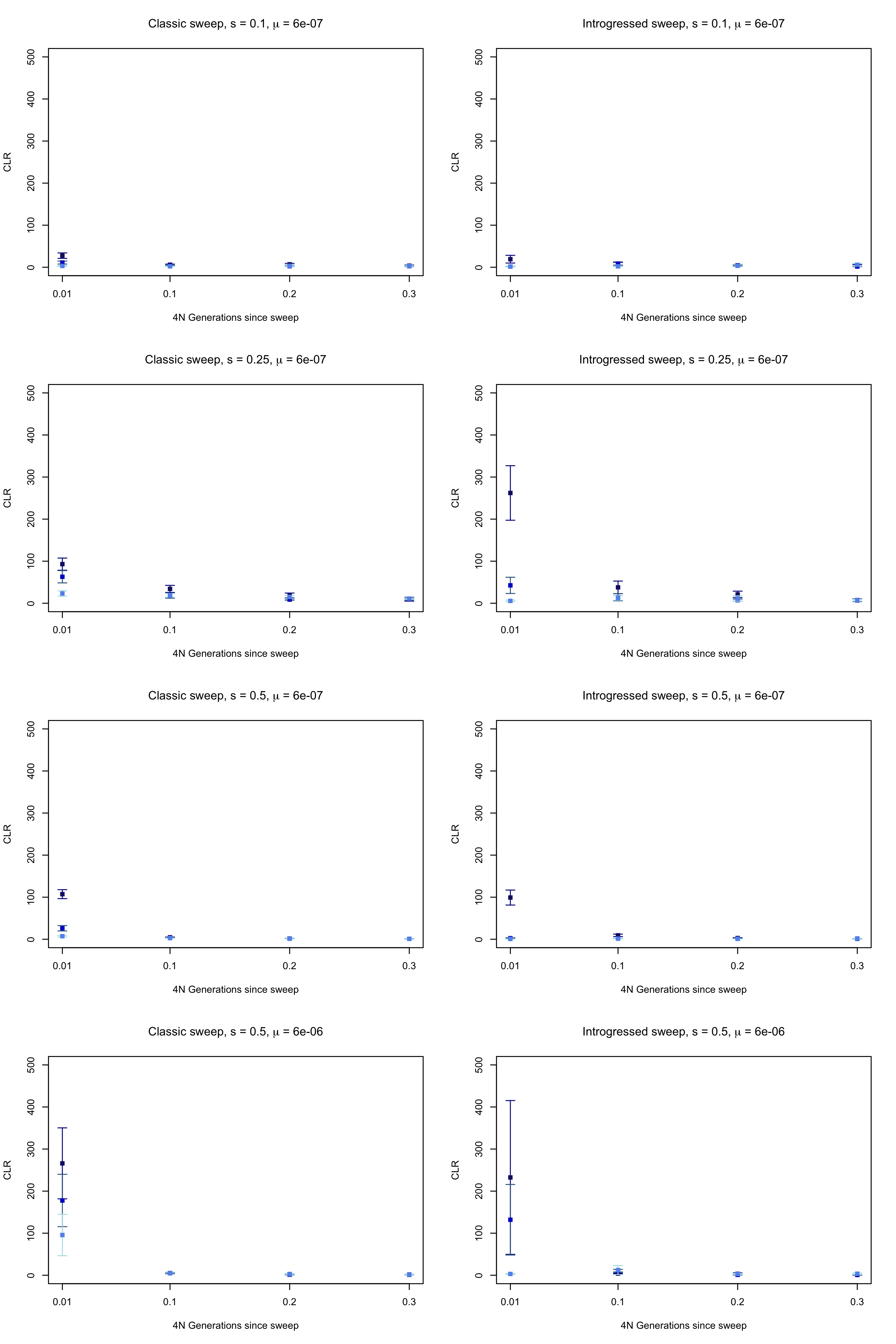

Supplement: S4 Fig — Plotted is the CLR statistic over time at 3 chromosome positions relative to the sweep centre, which correspond to the sweep site itself (dark blue), 0.02 Mb from the sweep (mid blue), and 0.04 Mb from the sweep (light blue), for 4 different simulation parameters. Selection coefficient, s = 0.25, neutral mutation rate, μ = 6e-07 corresponds to Fig 2, with average SF2 values calculated over 100 simulation runs, along with their standard errors. We also explored changes in s and μ in our simulations. Averages over 20 simulation runs are shown, along with their standard errors. Time is given in units of scaled generations. CLR, composite likelihood ratio; SF2, SweepFinder2 (PNG) [file pbio.3000597.s004.png]

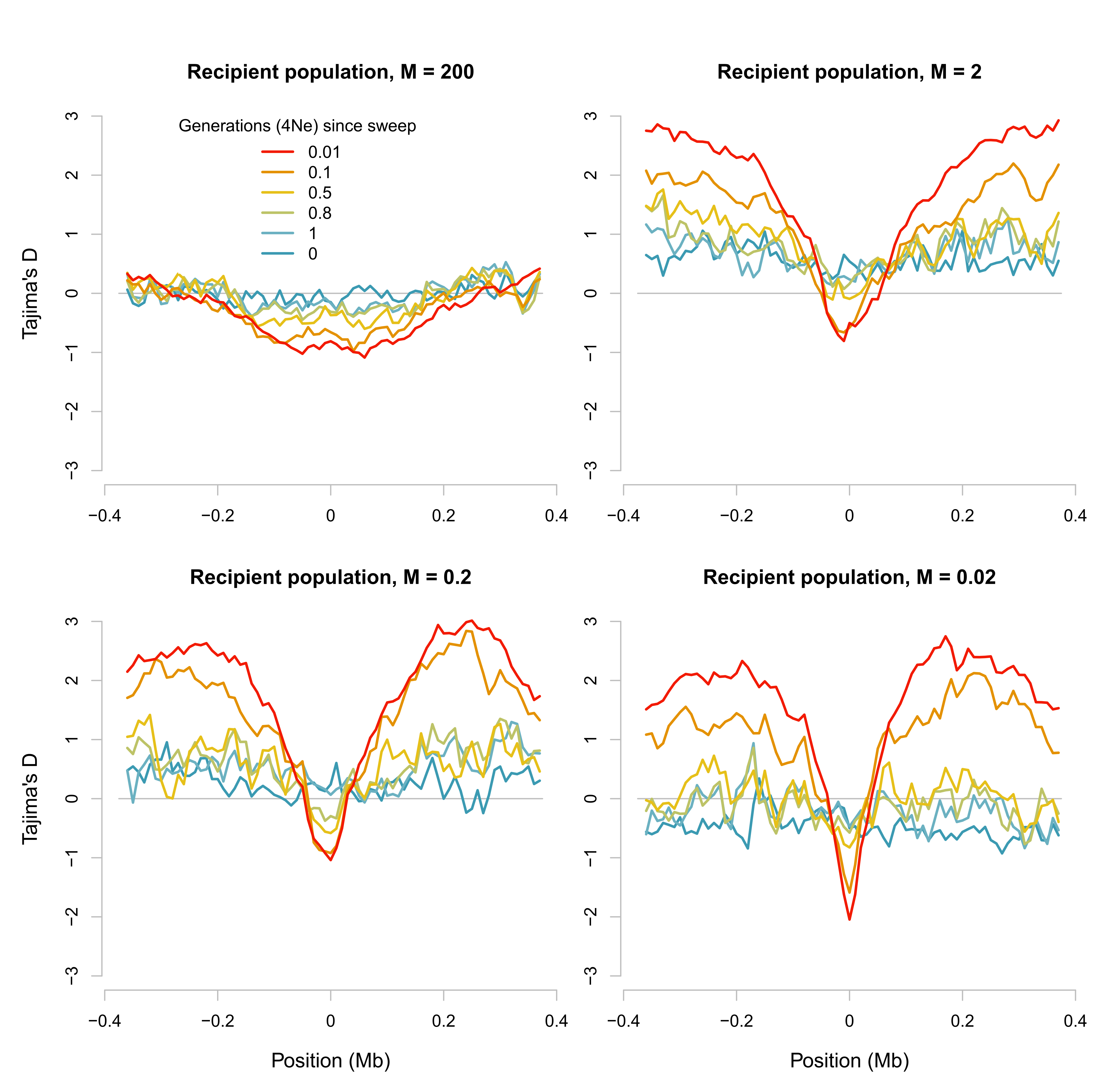

Supplement: S5 Fig — SFS signatures of simulated introgressed sweeps across a chromosome for different time points summarised as Tajima’s D statistics. The sweep occurs in the centre of the simulated chromosome. Different colours indicate patterns at different time points since sweep (0.01, 0.1, 0.5, 0.8, and 1 scaled generations, i.e., 4N generations). Simulated data for 4 different effective migration rates are shown (M = 200, 2, 0.2, and 0.002). SFS, site frequency spectrum (PNG) [file pbio.3000597.s005.png]

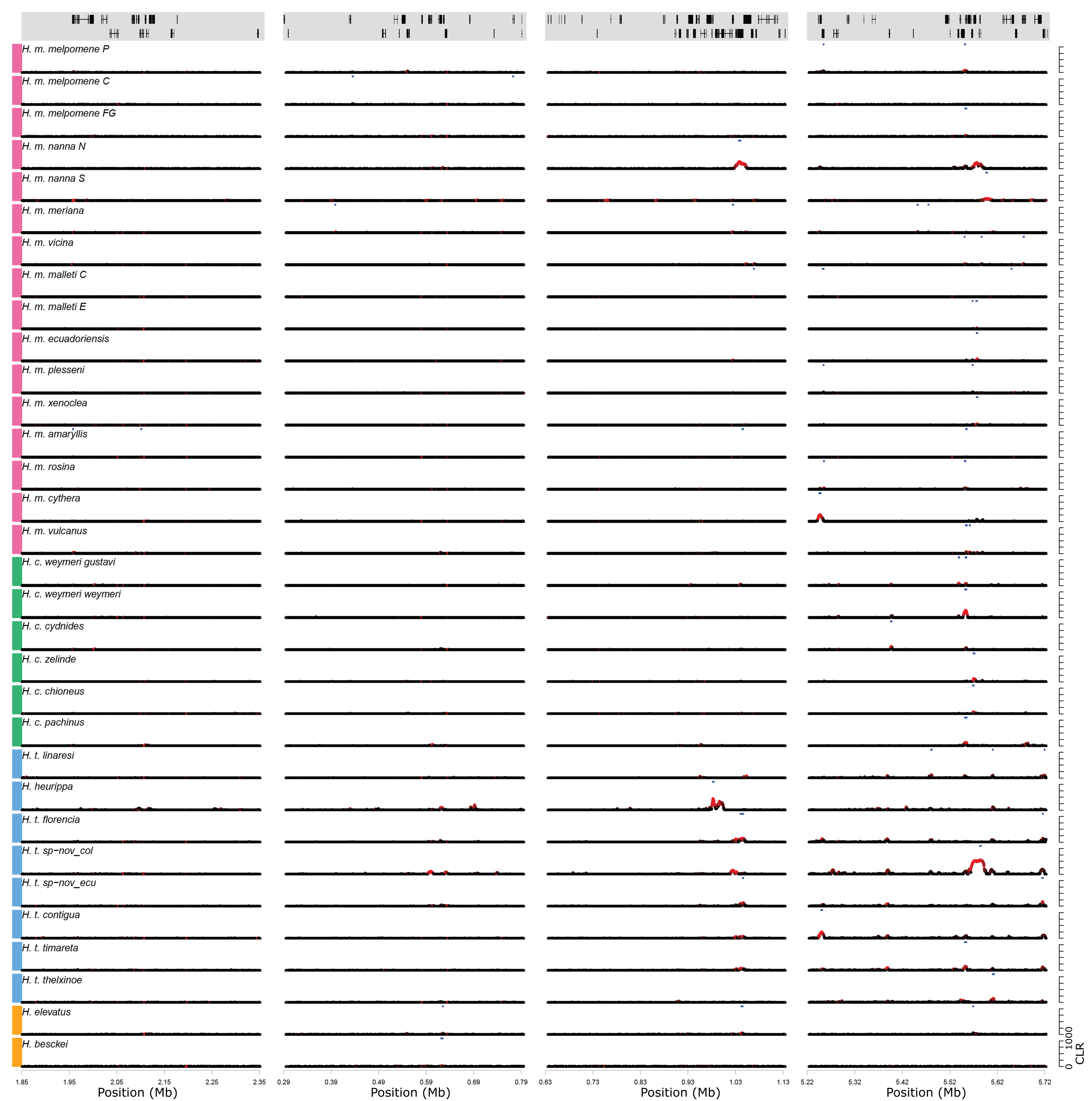

Supplement: S6 Fig — Genes are annotated in the top gene annotation panel. On the y-axis SF2’s [74,76] CLR statistics is shown (peaks are capped at CLR = 1,000). The colour gradient indicates estimated intensity of selection (black = high α values, weak selection; red = low α values, strong selection). Blue horizontal bars indicate regions with CLR values above threshold. CLR, composite likelihood ratio; SF2, SweepFinder2 (PNG) [file pbio.3000597.s006.png]

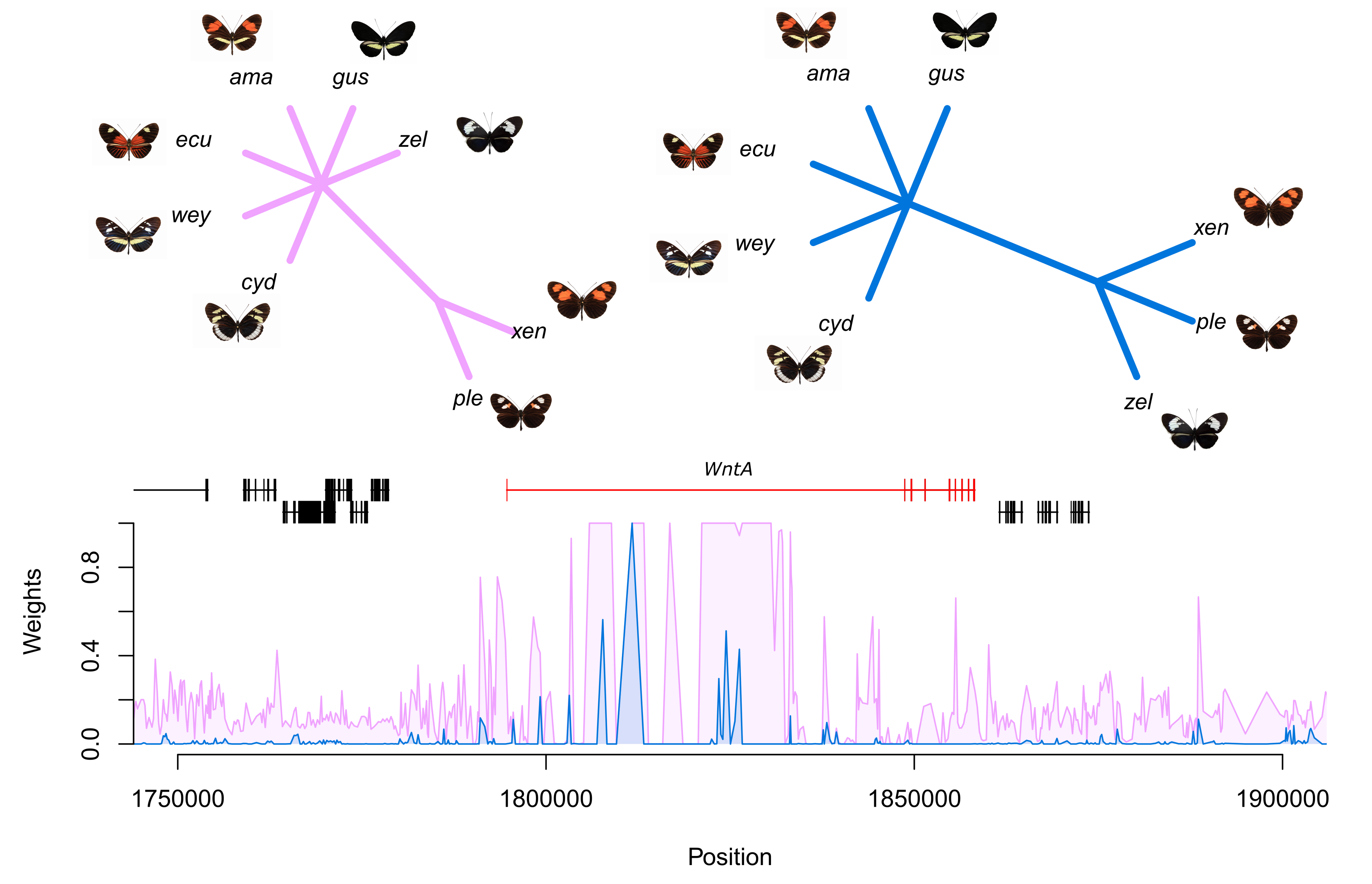

Supplement: S7 Fig — Topology weightings for topologies clustering the split forewing band phenotype (magenta) and the hourglass shape phenotype (blue) are shown. (ama = H. m. amaryllis, ecu = H. m. ecuadoriensis, ple = H. m. plesseni, xen = H. m. xenoclea, cyd = H. cydnides, wey = H. c. weymeri f. weymeri, gus = H. c. weymeri f. gustavi, zel = H. c. zelinde). (PNG) [file pbio.3000597.s007.png]

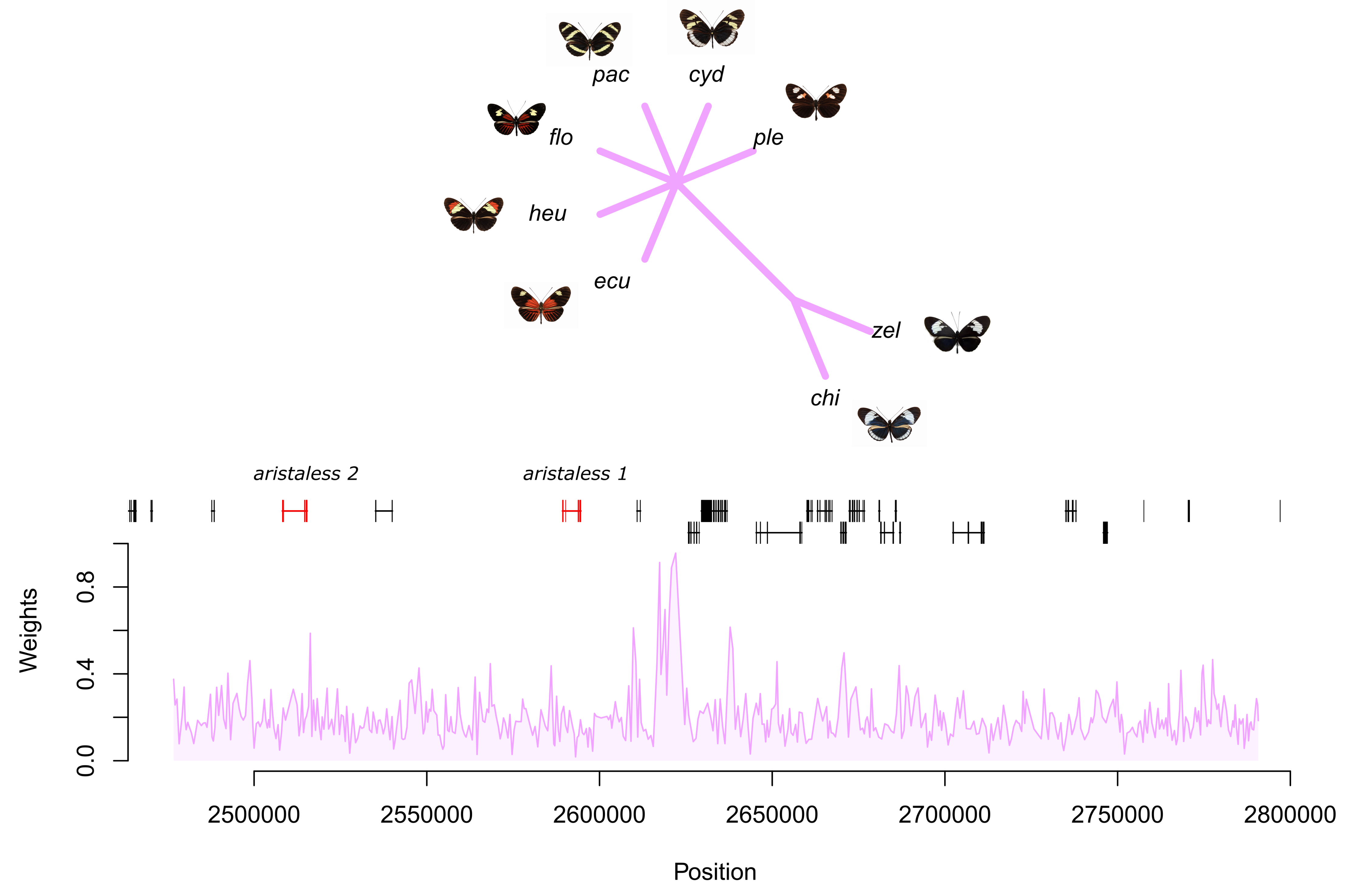

Supplement: S8 Fig — Topology weightings for topologies clustering the white (chi = H. c. chioneus, zel = H. c. zelinde) and yellow (ecu = H. m. ecuadoriensis, ple = H. m. plesseni, heu = H. heurippa, flo = H. t. florencia, cyd = H. cydnides, pac = H. pachinus) colour phenotypes (magenta) are shown. (PNG) [file pbio.3000597.s008.png]

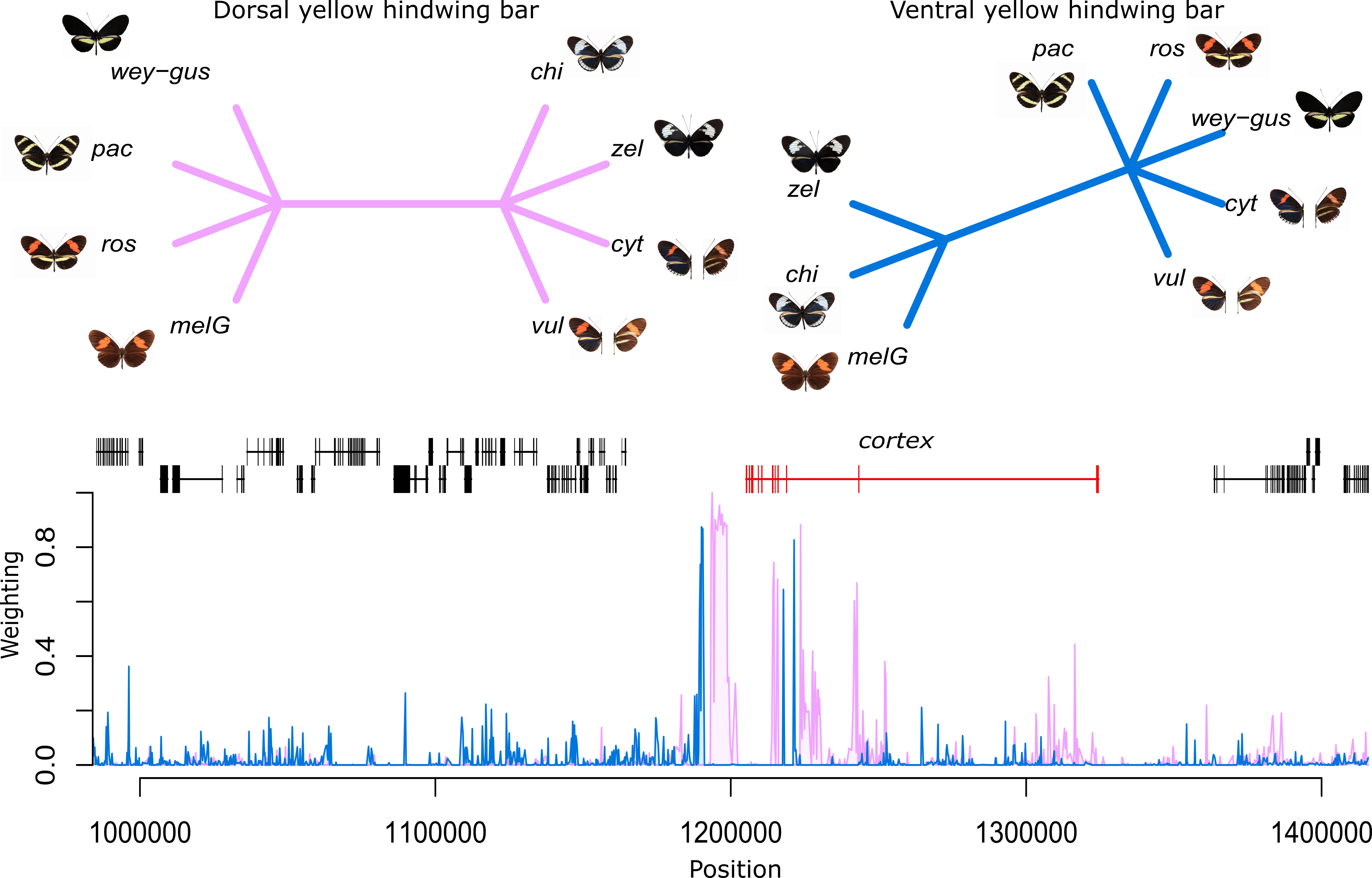

Supplement: S9 Fig — Topology weightings for topologies clustering the dorsal yellow hindwing bar (magenta) and ventral yellow hindwing bar (blue) phenotypes are shown (cyt = H. m. cythera, bur = H. m burchelli, nan = H. m. nanna, ros = H. m. rosina, vul = H. m. vulcanus, chi = H. c. chioneus, wey = H. c. weymeri f. weymeri, gus = H. c. weymeri f. gustavi, zel = H. c. zelinde, pac = H. pachinus). (PNG) [file pbio.3000597.s009.png]

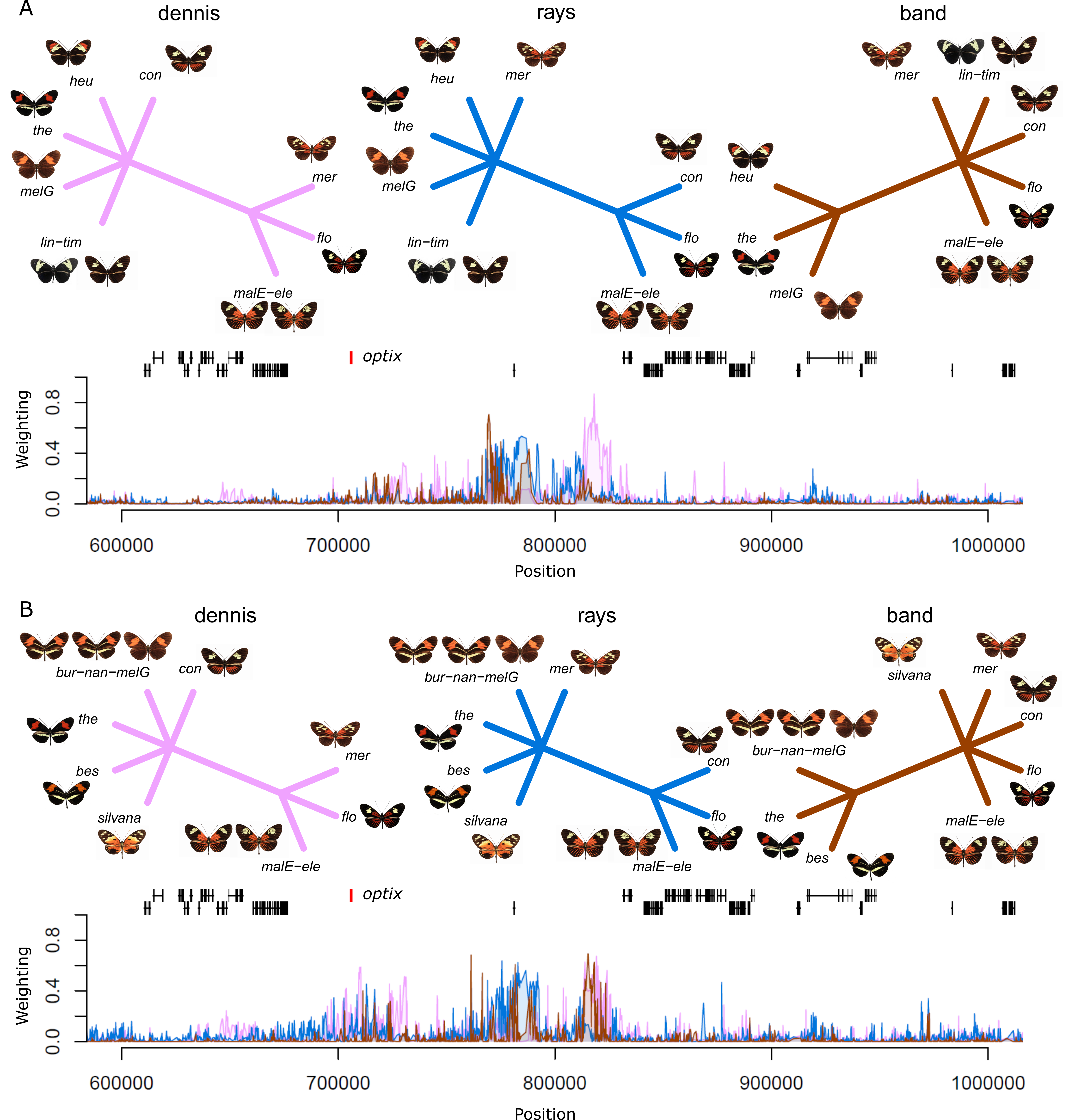

Supplement: S10 Fig — Topology weightings for topologies clustering the dennis (magenta), rays (blue), and band (brown) phenotypes. Including different red banded populations shows different phylogenetic clustering and thus potentially a different genetic basis underlying this trait among populations. (A) Tree weighting including the Peruvian red banded population H. t. thelxinoe. (B) Tree weighting including red banded populations from East Brazil, H. m. burchelli, H. m. nanna and H. besckei. (bur = H. m burchelli, malE = H. m. malleti (ECU), melG = H. m, melpomene (FG), mer = H. m. meriana, nan = H. m. nanna, ros = H. m. rosina, vul = H. m. vulcanus, heu = H. heurippa, flo = H. t. florencia, lin = H. t. linaresi, the = H. t. thelxinoe, tim = H. t. timareta f. timareta, con = H. t. timareta f. contigua, ele = H. elevatus, bes = H. besckei, silvana = H. numata silvana). (PNG) [file pbio.3000597.s010.png]

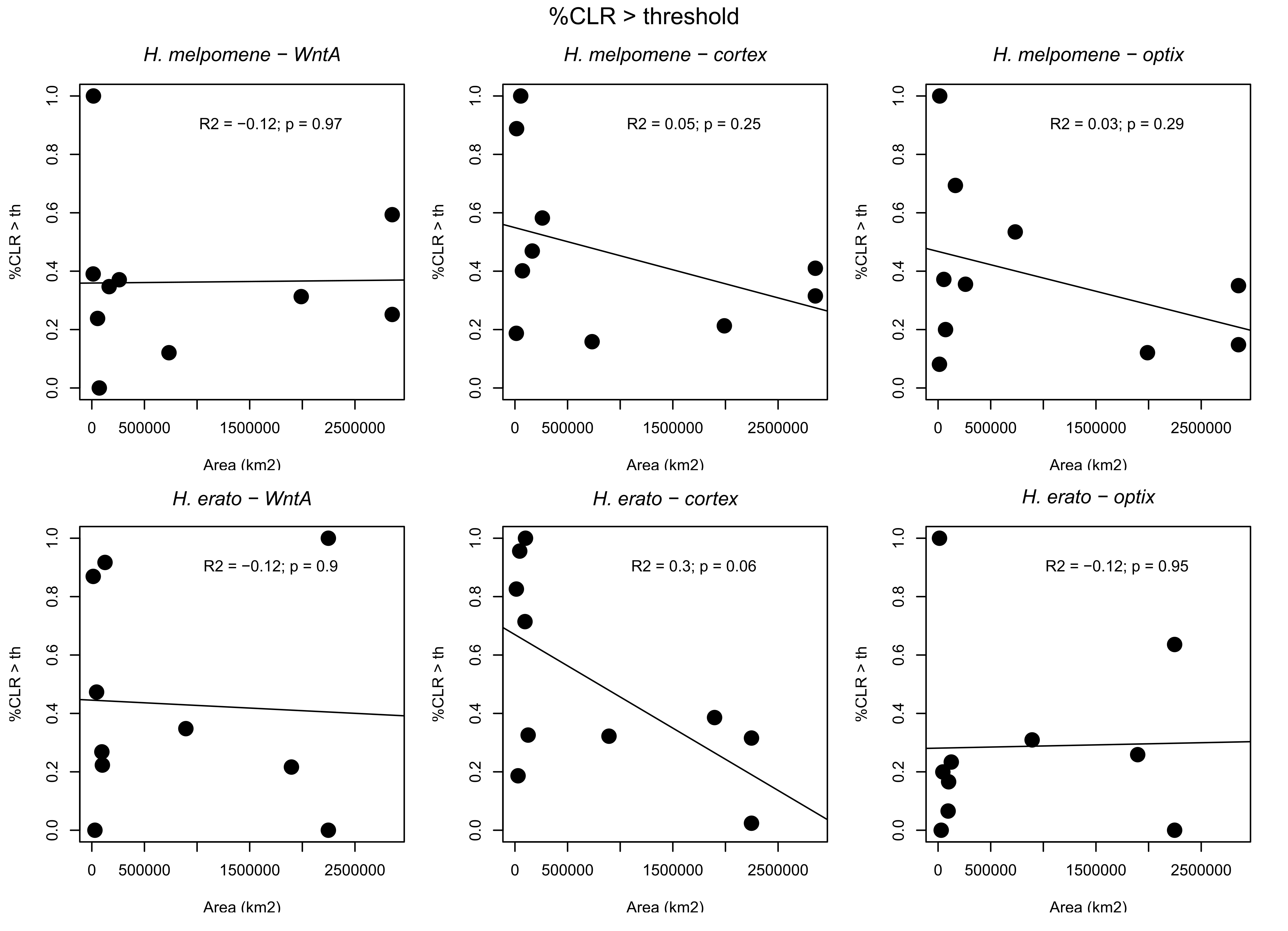

Supplement: S12 Fig — Portion of genomic loci under selection is summarised as percentage of CLR values across the colour pattern region which are above the CLR threshold [%CLR>th] scaled by the maximum value for WntA, cortex, and optix regions. Areas were calculated from distribution data obtained from [136] using an alpha hull polygon (code available at https://github.com/StevenVB12/Sample-distributions). (PNG) [file pbio.3000597.s012.png]

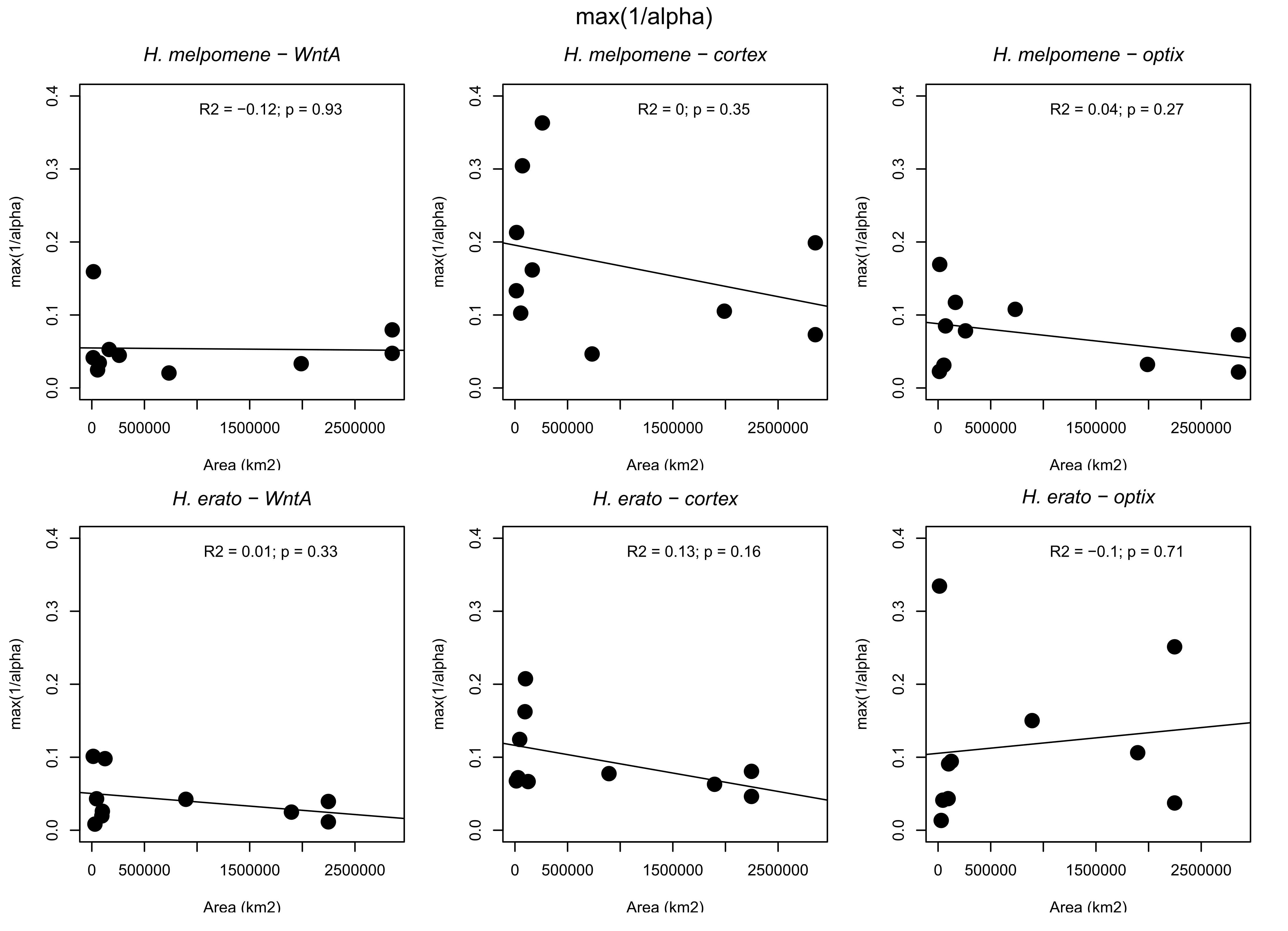

Supplement: S13 Fig — Areas were calculated from distribution data obtained from Rosser and colleagues [136] using a alpha hull polygon (code available at https://github.com/StevenVB12/Sample-distributions). (PNG) [file pbio.3000597.s013.png]

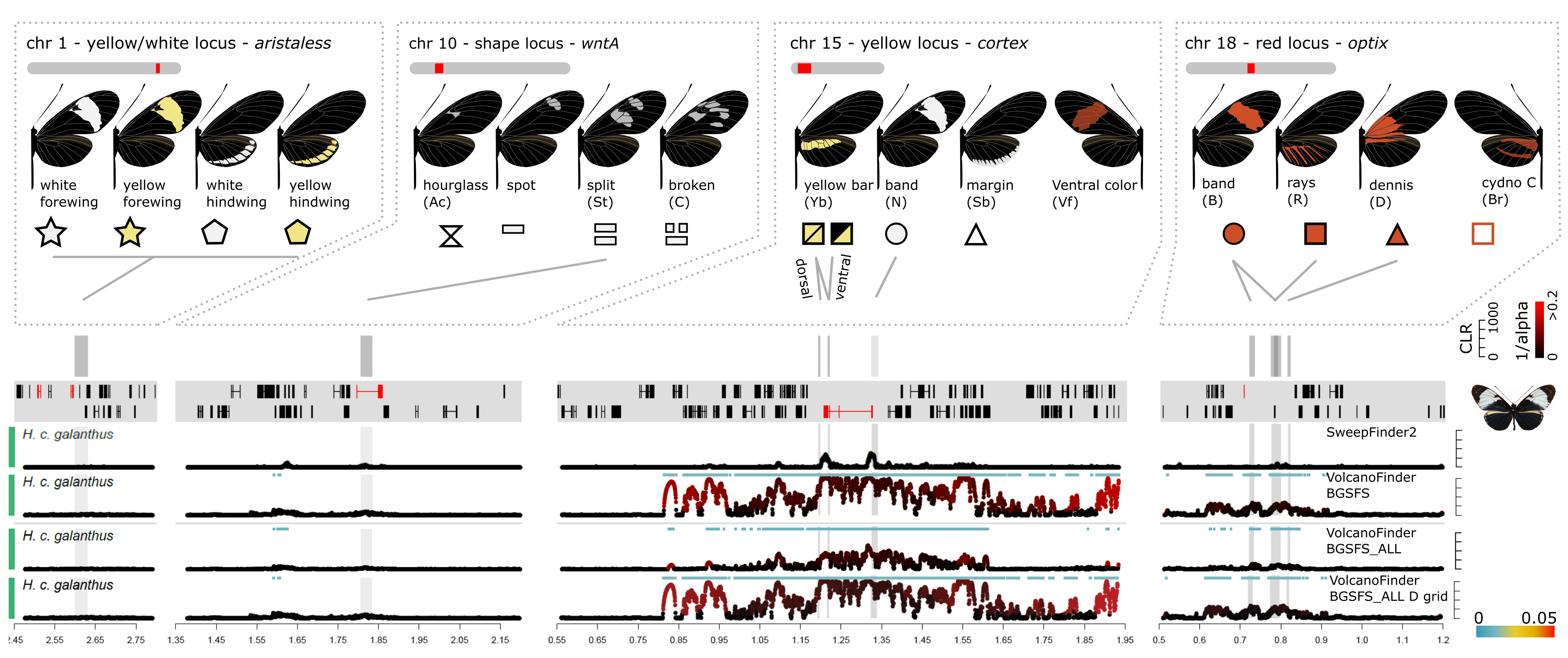

Supplement: S14 Fig — The regions containing the tandem copies of aristaless, al1 and al2, WntA, cortex, and optix (left to right) are depicted. Colour pattern genes are annotated in red in the gene annotation panel. On the y-axis Sweepfinder2’s and VolcanoFinder’s CLR statistics are shown (peaks capped at 1,000). The colour gradient indicates the estimated intensity of selection α (black…high α values, weak selection; red…low α values, strong selection). Grey shadings indicate annotated colour pattern CREs [28,30,36,37,39] (S7–S10 Figs). Coloured horizontal bars indicate regions with CLR values above threshold and for VolcanoFinder results, the colour gradient indicates the estimated D value. Top panel shows colour pattern phenotypes, and symbols indicate distinct colour pattern elements and their presence is annotated in population panels. Note that the yellow hindwing bar controlled by the cortex region can be expressed on the dorsal and ventral side (yellow/yellow square symbol) or on the ventral side only (black/yellow square symbol) [39]. Moreover, the actual shape of the forewing band can depend on the allelic state of WntA. Full, grey lines connect colour pattern elements with annotated CREs. The H. c. galanthus phenotype is depicted on the right. CLR, composite likelihood ratio; CRE, cis-regulatory element. (PNG) [file pbio.3000597.s014.png]

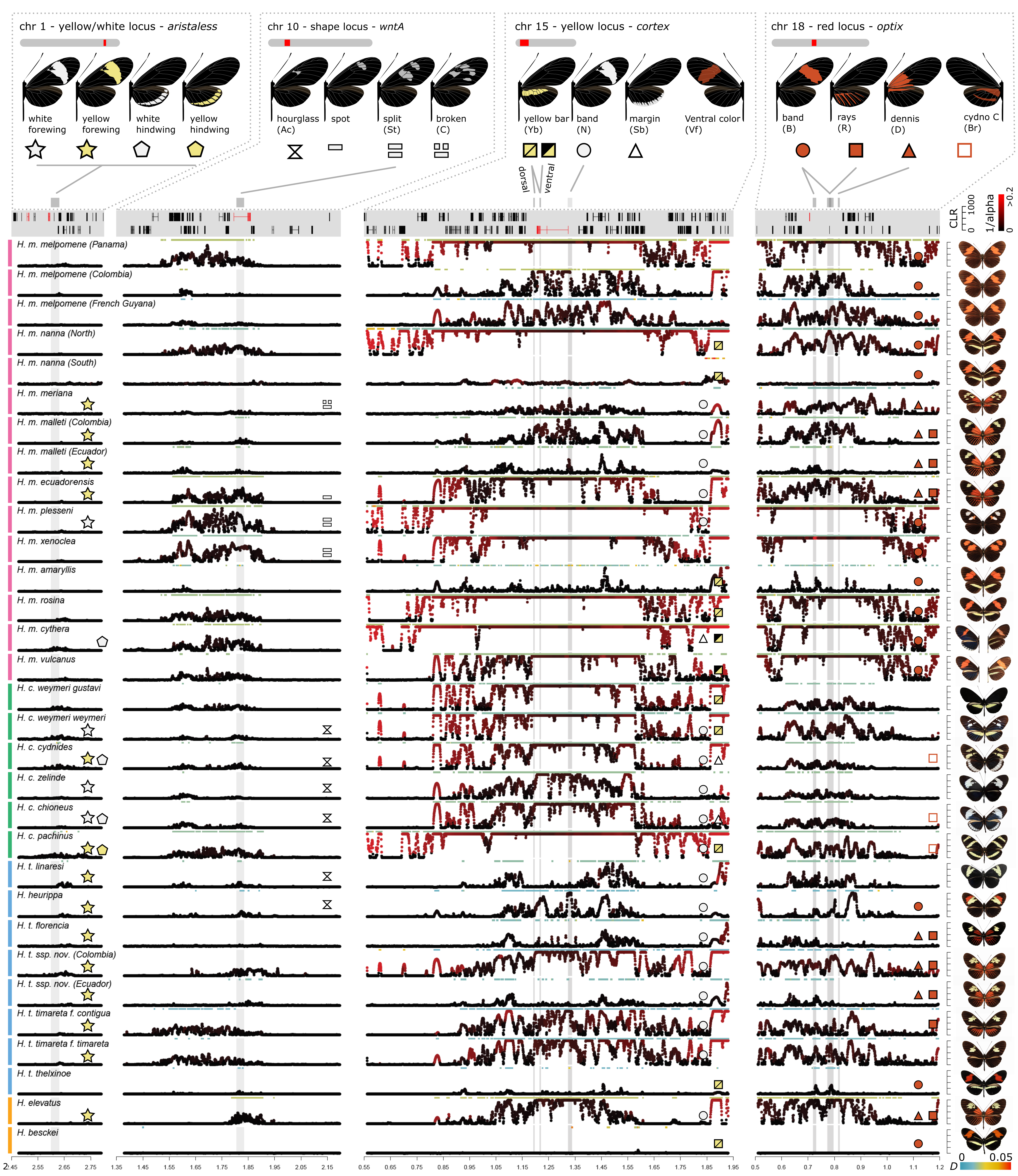

Supplement: S15 Fig — The regions containing the tandem copies of aristaless, al1 and al2, WntA, cortex, and optix (left to right) are depicted. Colour pattern genes are annotated in red in the gene annotation panel. On the y-axis VolcanoFinder’s CLR statistics is shown (peaks capped at 1,000). The colour gradient indicates the estimated intensity of selection α (black…high α values, weak selection; red…low α values, strong selection). Grey shadings indicate annotated colour pattern CREs [28,30,36,37,39] (S7–S10 Figs). Coloured horizontal bars indicate regions with CLR values above threshold and the colour gradient indicates the estimated D value. Top panel shows colour pattern phenotypes and symbols indicate distinct colour pattern elements and their presence is annotated in population panels. Note that the yellow hindwing bar controlled by the cortex region can be expressed on the dorsal and ventral side (yellow/yellow square symbol) or on the ventral side only (black/yellow square symbol) [39]. Moreover, the actual shape of the forewing band can depend on the allelic state of WntA. Full, grey lines connect colour pattern elements with annotated CREs. CLR, composite likelihood ratio; CRE, cis-regulatory element. (PNG) [file pbio.3000597.s015.png]

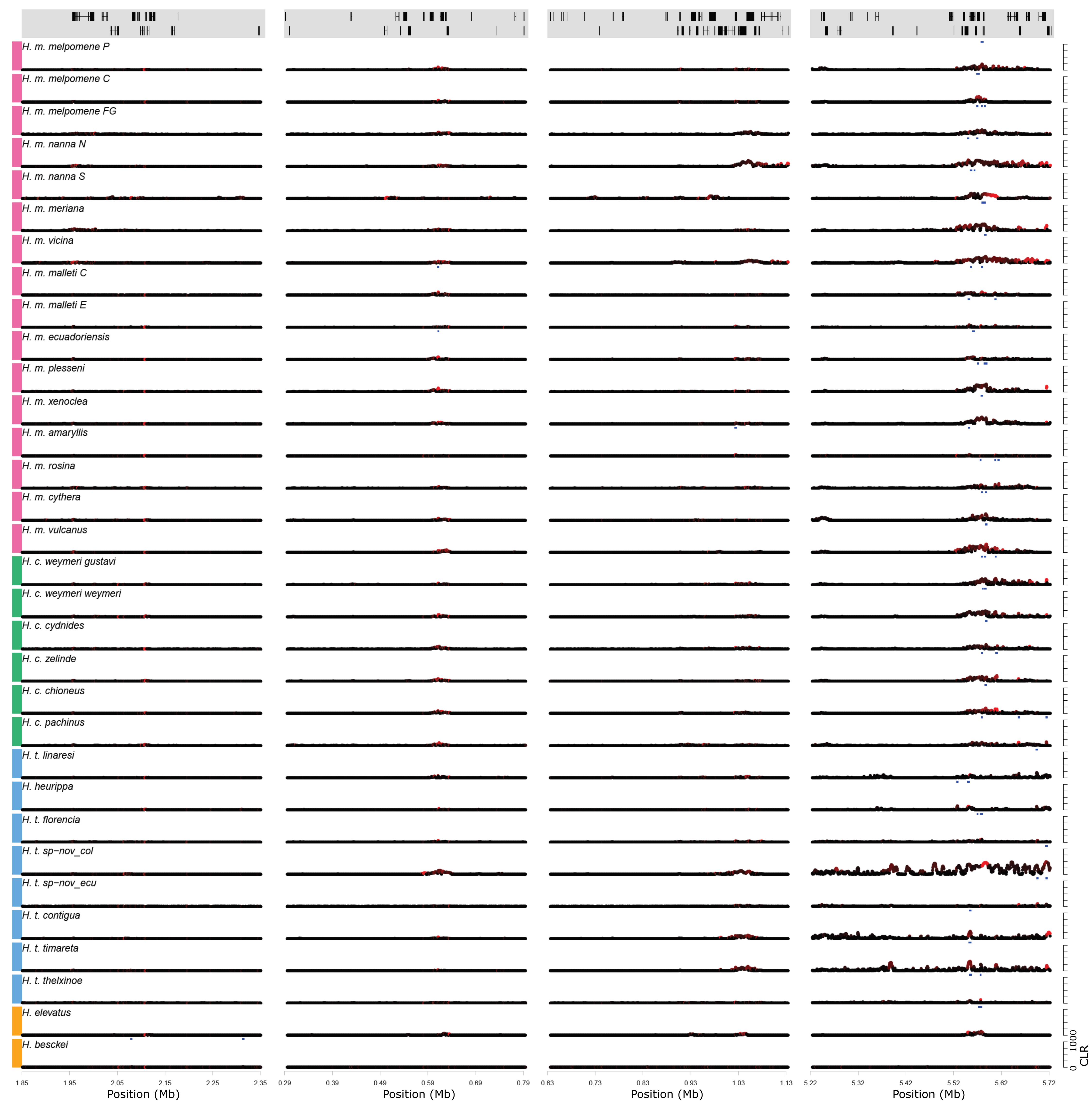

Supplement: S16 Fig — Genes are annotated in in the top gene annotation panel. On the y-axis VolcanoFinder’s CLR statistics is shown (peaks are capped at 1,000). The colour gradient indicates the estimated intensity of selection α (black…high α values, weak selection; red…low α values, strong selection). Coloured horizontal bars indicate regions with CLR values above threshold and the colour gradient indicates the estimated D value. CLR, composite likelihood ratio (PNG) [file pbio.3000597.s016.png]

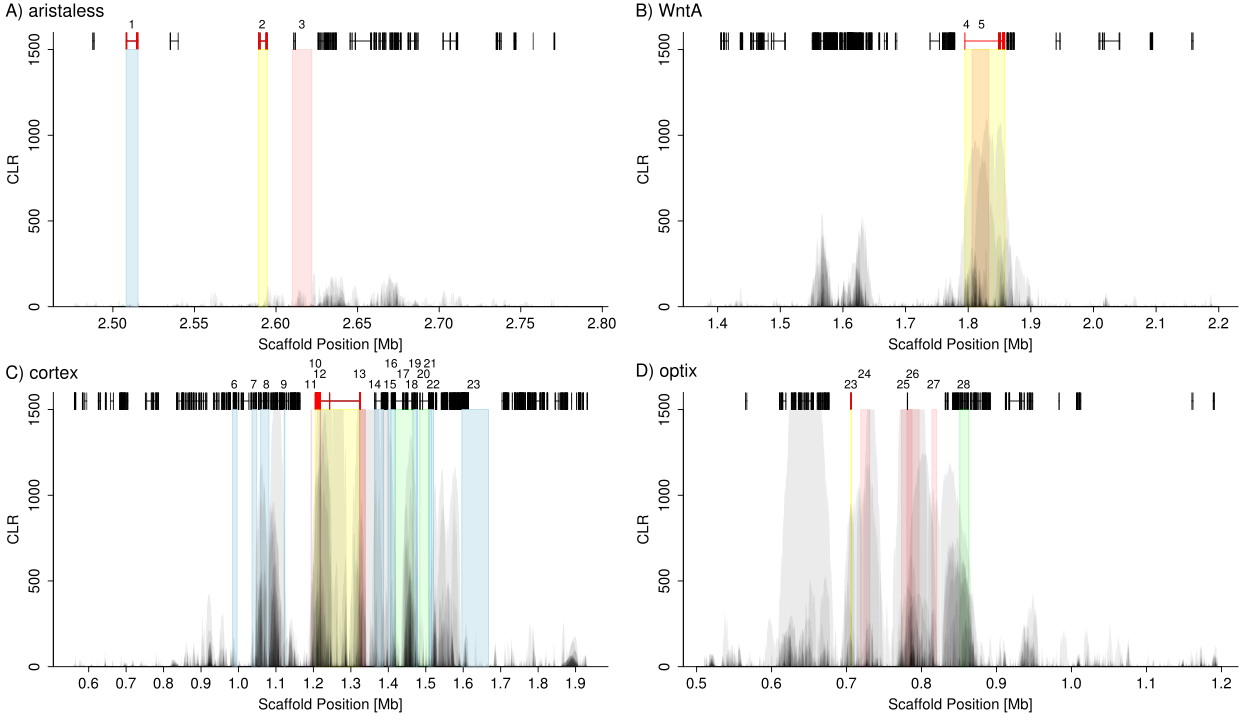

Supplement: S17 Fig — Superimposed, semitransparent SweepFinder2 peaks are depicted in grey. Colour pattern genes (yellow), known CREs (red), and additional genes with evidence for a putative role in colour patterning (blue and green for genes discussed in the main text) are highlighted and assigned a number in the top row. The scale on the x-axes differs and the y-axis is capped at CLR = 1,500. (A) aristaless1 (yellow, 2), aristaless1 CRE (red, 3) [28], aristaless2 (blue, 1); (B) WntA (yellow, 4), CRE associated with split forewing band identified in this study (red, 5); (C) cortex (yellow, 10), CREs for dorsal (11) and ventral (12) hindwing topology [39], a region containing SNPs with strongest association with forewing band [30] (13) (red), additional genes with evidence for wing patterning control [30] (blue: 7, 8, 9, 14, 15, 16, 18, 19, 21, 22, 23; green: 17 (LMTK1 /HM00033), 20 (washout/WAS homologue 1/HM00036); also see S9 Table); (D) optix (yellow, 23), CREs for ‘band1’(24), ‘band2’(26), ‘rays’(25) and ‘dennis’(27) (red) [36,37], kinesin (green, 28) [86,87]. A genome viewer in which these regions and accession can be viewed in detail is available at http://lepbase.org/. CLR, composite likelihood ratio; CRE, cis-regulatory element; SNP, single-nucleotide polymorphism (PNG) [file pbio.3000597.s017.png]

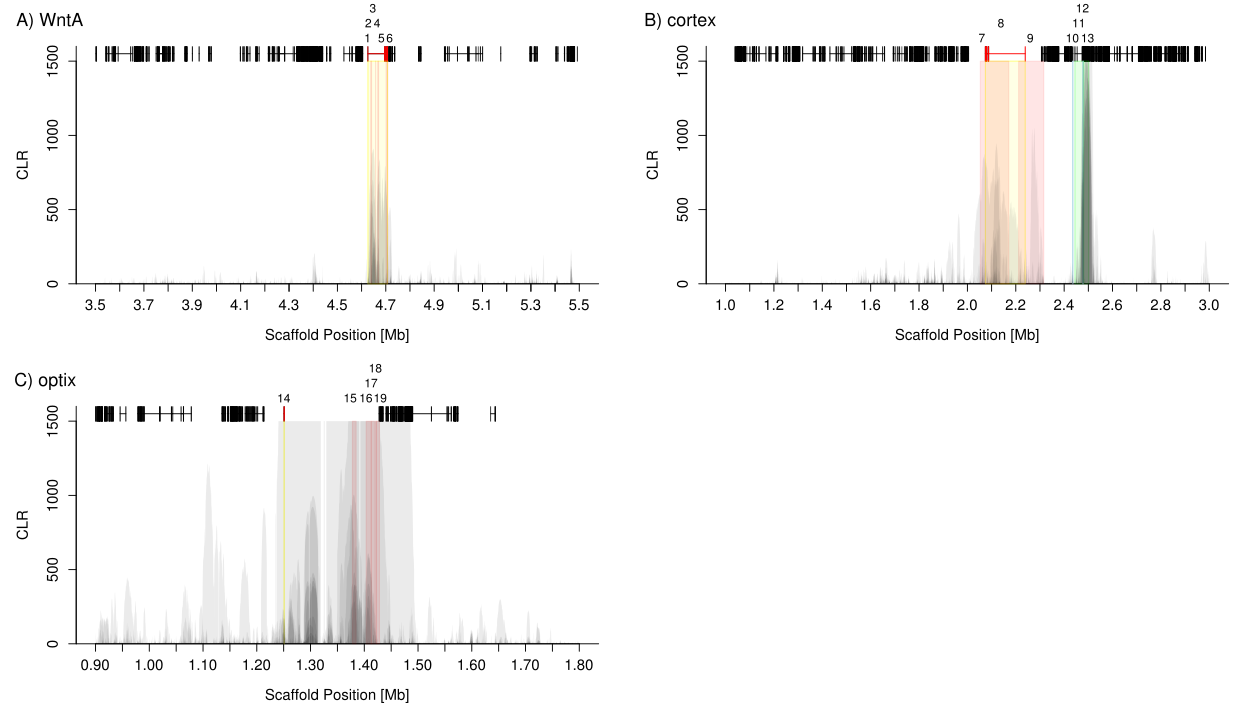

Supplement: S18 Fig — Superimposed, semitransparent SweepFinder2 peaks are depicted in grey. Colour pattern genes (yellow), known CREs (red), and additional genes with evidence for a putative role in colour patterning (blue and green for genes discussed in the main text) are highlighted and assigned a number in the top row. The scale on the x-axes differs and the y-axis is capped at CLR = 1,500. (A) WntA (yellow,1), CREs associated with ‘Sd1’(2), ‘Sd2’(3), ‘St’(4), ‘Ly1’(5) and ‘Ly2’(6) elements (red); (B) cortex (yellow, 8), ‘Cr1’(7) and ‘Cr2’(9) regions (red) [38], and additional genes with evidence for wing patterning control [30] (blue: 10,12; green; 11 (washout/WAS homologue 1/HERA000061), 13 (lethal (2)/HERA000062); also see S9 Table; (C) optix (yellow,14), CREs for ‘rays’(15), ‘band’ Y1(16)/ Y2(18), and ‘dennis’ D1(17)/ D2(19) elements (red) [38]. A genome viewer in which these regions and accession can be viewed in detail is available at http://lepbase.org/. CLR, composite likelihood ratio; CRE, colour pattern regulatory element. (PNG) [file pbio.3000597.s018.png]

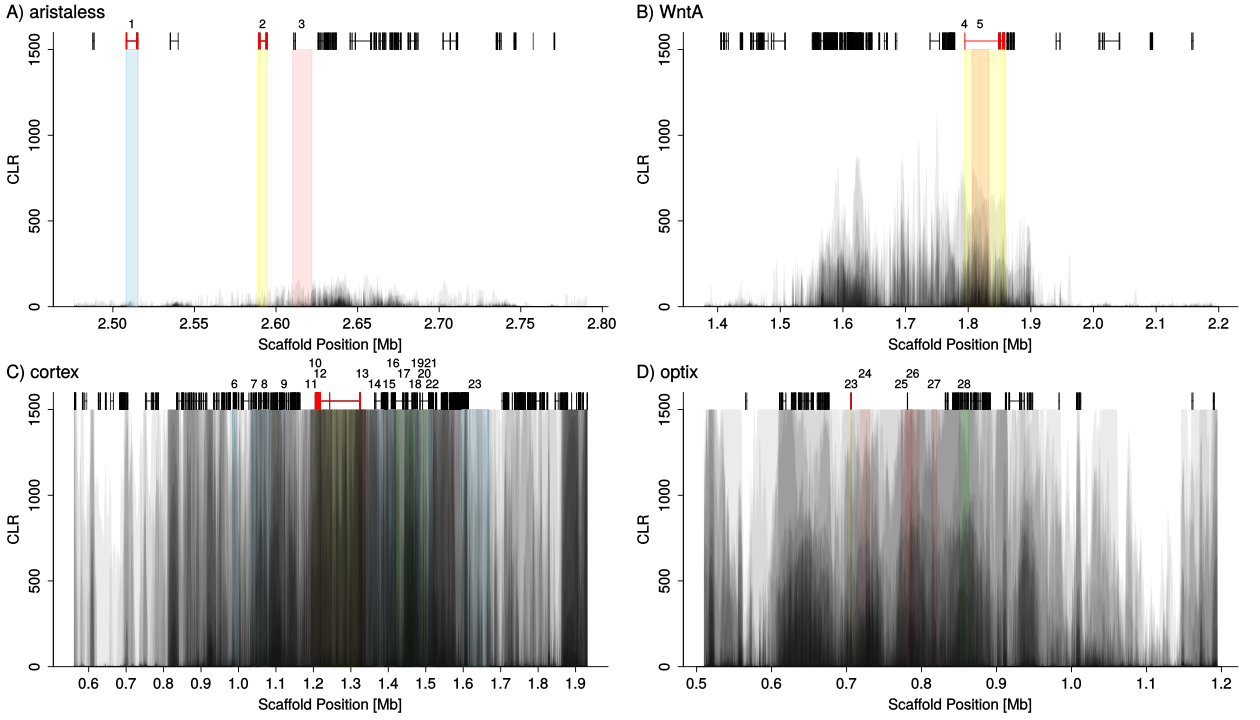

Supplement: S19 Fig — Superimposed, semitransparent VolcanoFinder2 peaks are depicted in grey. Colour pattern genes (yellow), known CREs (red), and additional genes with evidence for a putative role in colour patterning (blue and green for genes discussed in the main text) are highlighted and assigned a number in the top row. The scale on the x-axes differs and the y-axis is capped at CLR = 2,000. (A) aristaless1 (yellow, 2), aristaless1 CRE (red, 3) [28], aristaless2 (blue, 1); (B) WntA (yellow, 4), CRE associated with split forewing band identified in this study (red, 5); (C) cortex (yellow, 10), CREs for dorsal (11) and ventral (12) hindwing topology [39], a region containing SNPs with strongest association with forewing band [30] (13) (red), additional genes with evidence for wing patterning control [30] (blue: 7, 8, 9, 14, 15, 16, 18, 19, 21, 22, 23; green: 17 (LMTK1 /HM00033), 20 (washout/WAS homologue 1/HM00036); also see S9 Table); (D) optix (yellow, 23), CREs for ‘band1’(24), ‘band2’(26), ‘rays’(25) and ‘dennis’(27) (red) [36,37], kinesin (green, 28) [86,87]. A genome viewer in which these regions and accession can be viewed in detail is available at http://lepbase.org/. CLR, composite likelihood ratio; CRE, cis-regulatory element. (PNG) [file pbio.3000597.s019.png]

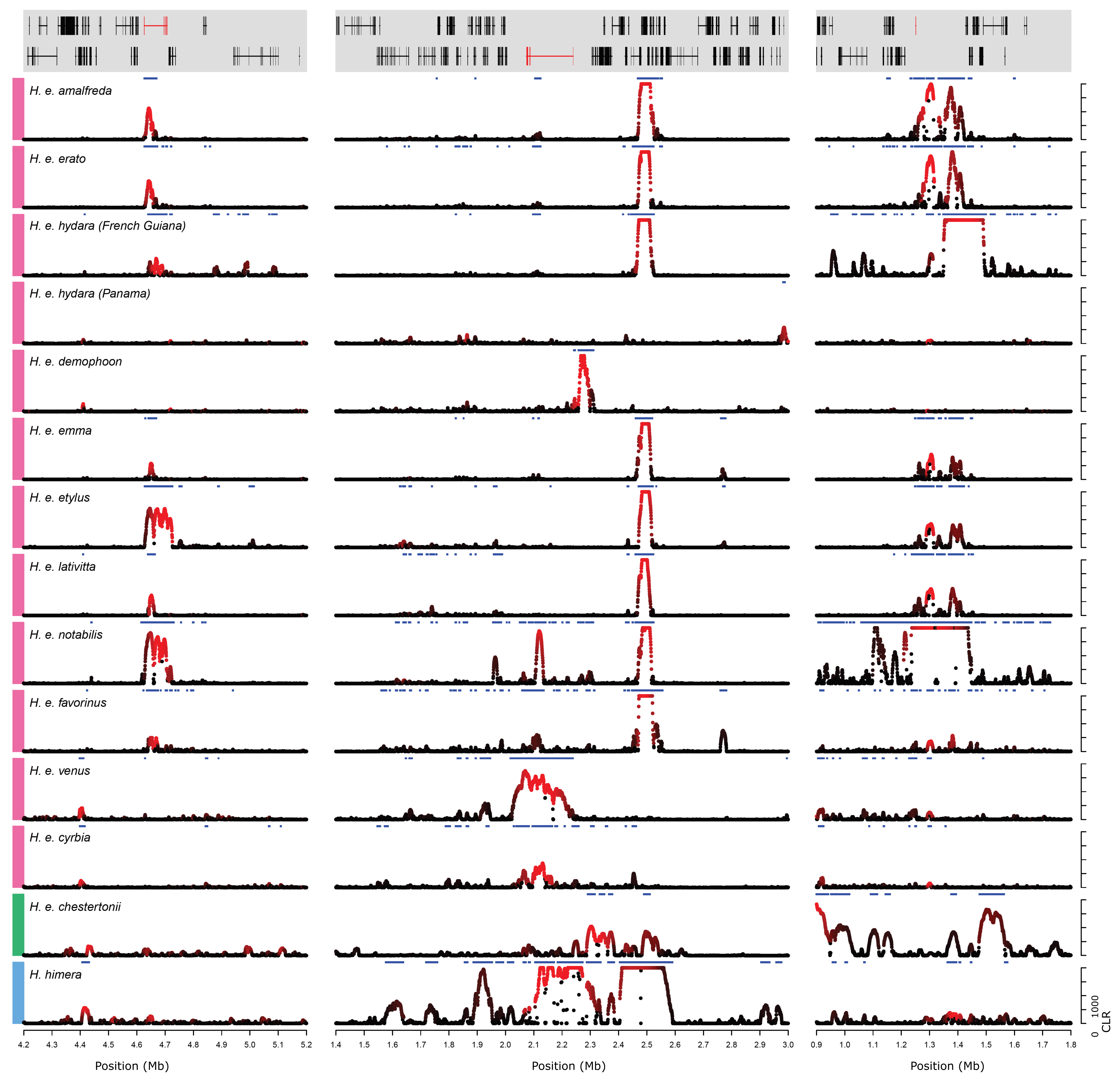

Supplement: S20 Fig — The regions containing WntA, cortex, and optix (left to right) are depicted. Colour pattern genes are annotated in red in the gene annotation panel. On the y-axis Sweepfinder2’s [74,76] CLR statistics is shown (peaks are capped at CLR = 1,000). The colour gradient indicates the estimated intensity of selection (black = high α values, weak selection; red = low α values, strong selection). Blue horizontal bars indicate regions above the CLR threshold value. CLR, composite likelihood ratio (PNG) [file pbio.3000597.s020.png]

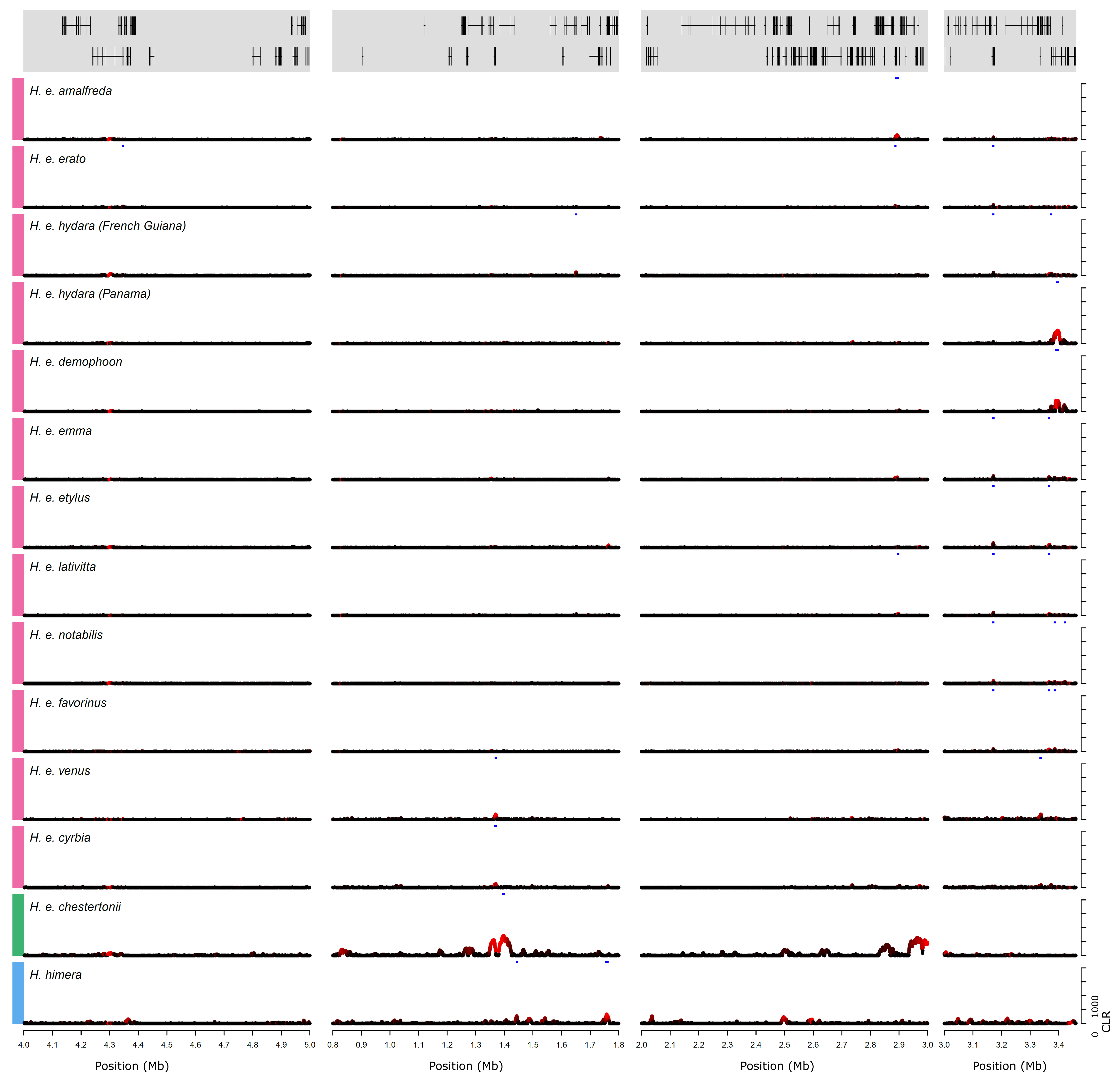

Supplement: S21 Fig — Genes are annotated in the top gene annotation panel. On the y-axis Sweepfinder2’s [74,76] CLR statistics is shown (peaks are capped at 1,000). The colour gradient indicates the estimated intensity of selection (black = high α values, weak selection; red = low α values, strong selection). Blue horizontal bars indicate regions above the CLR threshold value. CLR, composite likelihood ratio (PNG) [file pbio.3000597.s021.png]
