# Supplementary material for: Selective sweeps on novel and introgressed variation shape mimicry loci in a butterfly adaptive radiation
Source: PLoS Biol. 2020 Feb 6;18(2):e3000597. doi: 10.1371/journal.pbio.3000597 (PMC7029882; doi:10.1371/journal.pbio.3000597)
Supplement: S11 Fig — For each population genotyping coverage (calculated as proportion of retained genotypes after quality filtering in 500 bp windows), nucleotide diversity, Kelly’s ZnS, Tajima’s D, pooled integrated haplotype homozygosity score, and SweepFinder2’s [74,76] composite likelihood ratio statistics across each colour pattern region are shown (top to bottom). File names contain population and colour pattern region identifiers (Hmel201011 = aristaless scaffold, Hmel210004 = WntA scaffold, Hmel215006 = cortex scaffold, Hmel218003 = optix scaffold). The 120 single figures have been uploaded to GitHub:https://github.com/markusmoest/SelectionHeliconius/tree/master/S11_Fig_H_melpomene. (PDF) [file pbio.3000597.s011.pdf]

S11 Fig. Summary and selection statistics across colour pattern regions for all populations analysed in the *Heliconius melpomene*-clade. For each population genotyping coverage (calculated as proportion of retained genotypes after quality filtering in 500 bp windows), nucleotide diversity, Kelly's Z_{nS}, Tajima's *D*, pooled integrated haplotype homozygosity score, and SweepFinder2's [74,76] composite likelihood ratio statistics across each colour pattern region are shown (top to bottom). File names contain population and colour pattern region identifiers (Hmel201011 = *aristaless* scaffold, Hmel210004 = *WntA* scaffold, Hmel215006 = *cortex* scaffold, Hmel218003 = *optix* scaffold). The 120 single figures have been uploaded to GitHub:
https://github.com/markusmoest/SelectionHeliconius/tree/master/S11_Fig_H_melpomene
