# Supplementary material for: Selective sweeps on novel and introgressed variation shape mimicry loci in a butterfly adaptive radiation
Source: PLoS Biol. 2020 Feb 6;18(2):e3000597. doi: 10.1371/journal.pbio.3000597 (PMC7029882; doi:10.1371/journal.pbio.3000597)
Supplement: S22 Fig — For each population genotyping coverage (calculated as proportion of retained genotypes after quality filtering in 500 bp windows), nucleotide diversity, Kelly’s ZnS, Tajima’s D, pooled integrated haplotype homozygosity score, and SweepFinder2’s [74,76] CLR statistics across each colour pattern region are shown (top to bottom). File names contain population and colour pattern region identifiers (Herato1001 = WntA scaffold, Herato1505 = cortex scaffold, Herato1801 = optix scaffold). The 18 single figures have been uploaded to GitHub:https://github.com/markusmoest/SelectionHeliconius/tree/master/S22_Fig_H_erato. CLR, composite likelihood ratio. (PDF) [file pbio.3000597.s022.pdf]

S22 Fig. Summary and selection statistics across colour pattern regions for all populations analysed in the *Heliconius erato*-clade. For each population genotyping coverage (calculated as proportion of retained genotypes after quality filtering in 500 bp windows), nucleotide diversity, Kelly's Z_{nS}, Tajima's D, pooled integrated haplotype homozygosity score, and SweepFinder2's [74,76] composite likelihood ratio statistics across each colour pattern region are shown (top to bottom). File names contain population and colour pattern region identifiers (Herato1001 = *WntA* scaffold, Herato1505 = *cortex* scaffold, Herato1801 = *optix* scaffold). The 18 single figures have been uploaded to GitHub:
https://github.com/markusmoest/SelectionHeliconius/tree/master/S22_Fig_H_erato
