# Supplementary material for: Selective sweeps on novel and introgressed variation shape mimicry loci in a butterfly adaptive radiation
Source: PLoS Biol. 2020 Feb 6;18(2):e3000597. doi: 10.1371/journal.pbio.3000597 (PMC7029882; doi:10.1371/journal.pbio.3000597)
Supplement: S1 Table — (PDF) [file pbio.3000597.s023.pdf]

S1 Table. Sample information and genotyping statistics for all samples from the *Heliconius melpomene*-clade.

SequenceID	EarthCapeID	Taxon Name	Country	Latitude	Longitude	Accession	Publication	Called sites	Geno-typed sites after filters (%)	Proport-ion of hetero-zygous genotypes (%)	Data Type (WGS…whole genome re-sequencing, CS…capture sequencing)
GM110_86	CAM019819	*Heliconius besckei*	Brazil	-26.2500	-49.3836	ERR3652638		3,140,439	60.08	0.80	CS
GM110_83	CAM019818	*Heliconius besckei*	Brazil	-26.2500	-49.3836	ERR3652637		3,246,473	62.07	0.85	CS
GM110_82	CAM019817	*Heliconius besckei*	Brazil	-26.2500	-49.3836	ERR3652635		3,220,010	61.59	0.82	CS
GM123_2	CAM019816	*Heliconius besckei*	Brazil	-29.4423	-50.5798	ERR3652633		3,164,769	60.55	0.79	CS
GM81_3	CAM019815	*Heliconius besckei*	Brazil	-25.0462	-51.5395	ERR3652632		3,120,441	59.71	0.77	CS
GM90_3	CAM019814	*Heliconius besckei*	Brazil	-20.1515	-44.2011	ERR3652630		3,189,219	60.99	0.82	CS
GM89_2	CAM019813	*Heliconius besckei*	Brazil	-19.8226	-43.6762	ERR3652629		3,200,508	61.26	0.74	CS
GM110_84	CAM019812	*Heliconius besckei*	Brazil	-26.2500	-49.3836	ERR3652655		3,189,985	61.05	0.76	CS
GM81_4	CAM019811	*Heliconius besckei*	Brazil	-25.0462	-51.5395	ERR3652653		3,319,062	63.48	0.82	CS
GM88_7	CAM019810	*Heliconius besckei*	Brazil	-19.8853	-43.6644	ERR3652651		3,224,622	61.69	0.79	CS
GM88_1	CAM019809	*Heliconius besckei*	Brazil	-19.8853	-43.6644	ERR3652650		3,245,428	62.10	0.78	CS
chi.CAM584	CAM000584	*Heliconius cydno ssp. chioneus*	Panamá	9.1366	-79.7236	SAMEA104585046		3,277,148	61.75	2.29	WGS
chi.CAM580	CAM000580	*Heliconius cydno ssp. chioneus*	Panamá	9.1366	-79.7236	SAMEA104585044	Van Belleghem *et al.*[51]	3,534,220	66.61	2.26	WGS
chi.CAM582	CAM000582	*Heliconius cydno ssp. chioneus*	Panamá	9.1366	-79.7236	SAMEA104585045	Van Belleghem *et al.*[51]	3,654,172	68.82	2.33	WGS
chi.CAM585	CAM000585	*Heliconius cydno ssp. chioneus*	Panamá	9.1366	-79.7236	SAMEA104585047	Van Belleghem *et al.*[51]	3,469,652	65.38	2.29	WGS
chi.CAM586	CAM000586	*Heliconius cydno ssp. chioneus*	Panamá	9.1366	-79.7236	SAMEA104585048	Van Belleghem *et al.*[51]	3,538,969	66.67	2.31	WGS
chi.CAM25091	CAM025091	*Heliconius cydno ssp. chioneus*	Panamá	9.1200	-79.7020	SAMEA104585050	Van Belleghem *et al.*[51]	3,449,544	64.97	2.33	WGS
chi.CAM25137	CAM025137	*Heliconius cydno ssp. chioneus*	Panamá	9.1200	-79.7020	SAMEA104585051	Van Belleghem *et al.*[51]	3,548,034	66.82	2.35	WGS
chi.CJ553	CAM000553	*Heliconius cydno ssp. chioneus*	Panamá	9.1366	-79.7236	SAMEA1919256	Martin *et al.*[71]	3,666,518	69.06	2.33	WGS
chi.CJ565	CAM000565	*Heliconius cydno ssp. chioneus*	Panamá	9.1714	-79.7573	SAMEA1919262	Martin *et al.*[71]	3,799,061	71.53	2.36	WGS
chi.CJ560	CAM000560	*Heliconius cydno ssp. chioneus*	Panamá	9.1366	-79.7236	SAMEA1919265	Martin *et al.*[71]	3,694,123	69.60	2.30	WGS
chi.CJ564	CAM000564	*Heliconius cydno ssp. chioneus*	Panamá	9.1366	-79.7236	SAMEA1919278	Martin *et al.*[71]	3,704,117	69.75	2.36	WGS
cor.CS3	CS002167	*Heliconius cydno ssp. cordula*	Venezuela	7.9406	-72.3406	SAMEA104585052	Van Belleghem *et al.*[51]	3,783,632	71.33	2.24	WGS
cor.CS4	CS002258	*Heliconius cydno ssp. cordula*	Venezuela	7.9406	-72.3406	SAMEA104585053	Van Belleghem *et al.*[51]	3,775,592	71.22	2.18	WGS
cor.STRI7	STRI_007	*Heliconius cydno ssp. cordula*	Venezuela	7.7980	-72.2047	SAMEA3670511	Wallbank *et al.*[36]	3,780,111	71.17	2.37	WGS
CS2737	CS002737	*Heliconius cydno ssp. cydnides*	Colombia	3.8665	-76.3834	ERR3652413		3,818,575	72.17	1.99	CS
CS2729	CS002729	*Heliconius cydno ssp. cydnides*	Colombia	3.8525	-76.4269	ERR3652589		3,822,998	72.30	1.93	CS
CS2727	CS002727	*Heliconius cydno ssp. cydnides*	Colombia	3.8525	-76.4269	ERR3652576		3,822,358	72.29	1.93	CS
CS2748	CS002748	*Heliconius cydno ssp. cydnides*	Colombia	3.8665	-76.3834	ERR3652422		3,812,649	72.08	1.96	CS
CS2740	CS002740	*Heliconius cydno ssp. cydnides*	Colombia	3.8665	-76.3834	ERR3652419		3,857,734	72.86	2.06	CS
CS2738	CS002738	*Heliconius cydno ssp. cydnides*	Colombia	3.8665	-76.3834	ERR3652407		3,856,673	72.84	2.06	CS
CS2724	CS002724	*Heliconius cydno ssp. cydnides*	Colombia	3.8525	-76.4269	ERR3652416		3,841,436	72.58	2.03	CS
CS2731	CS002731	*Heliconius cydno ssp. cydnides*	Colombia	3.8525	-76.4269	ERR3652580		3,846,873	72.68	2.02	CS

SequenceID	EarthCapeID	Taxon Name	Country	Latitude	Longitude	Accession	Publication	Called sites	Geno-typed sites after filters (%)	Proport-ion of hetero-zygous genotypes (%)	Data Type (WGS...whole genome re-sequencing, CS...capture sequencing)
CS2739	CS002739	*Heliconius cydno ssp. cydnides*	Colombia	3.8665	-76.3834	ERR3652586		3,825,523	72.33	1.96	CS
CS2732	CS002732	*Heliconius cydno ssp. cydnides*	Colombia	3.8525	-76.4269	ERR3652410		3,840,359	72.72	1.81	CS
CS936	CS000936	*Heliconius cydno ssp. weymeri f. gustavi*	Colombia	3.4834	-76.6158	ERR3652600		3,840,229	72.86	1.62	CS
CS2492	CS002492	*Heliconius cydno ssp. weymeri f. gustavi*	Colombia	3.3242	-76.6364	ERR3652603		3,873,417	73.27	1.91	CS
CS2511	CS002511	*Heliconius cydno ssp. weymeri f. gustavi*	Colombia	3.6106	-76.5986	ERR3652615		3,866,621	73.24	1.7	CS
CS2506	CS002506	*Heliconius cydno ssp. weymeri f. gustavi*	Colombia	3.6106	-76.5986	ERR3652613		3,802,771	71.98	1.85	CS
CS1914	CS001914	*Heliconius cydno ssp. weymeri f. gustavi*	Colombia	2.5133	-76.5942	ERR3652595		3,821,081	72.38	1.78	CS
CS953	CS000953	*Heliconius cydno ssp. weymeri f. gustavi*	Colombia	3.4834	-76.6158	ERR3652591		3,838,035	72.73	1.74	CS
CS950	CS000950	*Heliconius cydno ssp. weymeri f. gustavi*	Colombia	3.4834	-76.6158	ERR3652597		3,823,525	72.35	1.88	CS
CS1926	CS001926	*Heliconius cydno ssp. weymeri f. gustavi*	Colombia	2.5133	-76.5942	ERR3652605		3,862,132	73.14	1.80	CS
CS2503	CS002503	*Heliconius cydno ssp. weymeri f. gustavi*	Colombia	2.5133	-76.5942	ERR3652607		3,845,743	72.86	1.76	CS
CS2504	CS002504	*Heliconius cydno ssp. weymeri f. gustavi*	Colombia	3.6106	-76.5986	ERR3652609		3,835,440	72.40	2.11	CS
CS982	CS000982	*Heliconius cydno ssp. weymeri f. weymeri*	Colombia	3.4834	-76.6158	ERR3652383		3,820,791	72.47	1.65	CS
CS942	CS000942	*Heliconius cydno ssp. weymeri f. weymeri*	Colombia	3.4834	-76.6158	ERR3652621		3,880,634	73.44	1.87	CS
CS2996	CS002996	*Heliconius cydno ssp. weymeri f. weymeri*	Colombia	3.4834	-76.6158	ERR3652386		3,827,233	72.48	1.80	CS
CS2998	CS002998	*Heliconius cydno ssp. weymeri f. weymeri*	Colombia	3.4834	-76.6158	ERR3652619		3,859,920	72.97	1.97	CS
CS943	CS000943	*Heliconius cydno ssp. weymeri f. weymeri*	Colombia	3.4834	-76.6158	ERR3656235		3,852,736	72.88	1.90	CS
CS951	CS000951	*Heliconius cydno ssp. weymeri f. weymeri*	Colombia	3.4834	-76.6158	ERR3652611		3,857,073	73.09	1.73	CS
CS960	CS000960	*Heliconius cydno ssp. weymeri f. weymeri*	Colombia	3.4834	-76.6158	ERR3652377		3,827,645	72.52	1.75	CS
CS945	CS000945	*Heliconius cydno ssp. weymeri f. weymeri*	Colombia	3.4834	-76.6158	ERR3652624		3,879,725	73.38	1.93	CS
CS2542	CS002542	*Heliconius cydno ssp. weymeri f. weymeri*	Colombia	3.3242	-76.6364	ERR3652617		3,844,853	72.78	1.84	CS
CS939	CS000939	*Heliconius cydno ssp. weymeri f. weymeri*	Colombia	3.4834	-76.6158	ERR3652380		3,826,346	72.55	1.68	CS
zel.CS1	CS002242	*Heliconius cydno ssp. zelinde*	Colombia	3.9394	-77.3689	SAMEA104106540	Enciso-Romero *et al.*[39]	3,921,772	74.40	1.62	WGS
zel.CS2	CS002261	*Heliconius cydno ssp. zelinde*	Colombia	3.9394	-77.3689	SAMEA104106542	Enciso-Romero *et al.*[39]	3,932,964	74.03	2.40	WGS
zel.CS30	CS002260	*Heliconius cydno ssp. zelinde*	Colombia	3.9394	-77.3689	SAMEA104106543	Enciso-Romero *et al.*[39]	3,958,948	74.78	2.05	WGS
zel.CS1028	CS001028	*Heliconius cydno ssp. zelinde*	Colombia	3.9394	-77.3689	SAMEA104585054	Van Belleghem *et al.*[51]	3,901,819	73.44	2.39	WGS
zel.CS1029	CS001029	*Heliconius cydno ssp. zelinde*	Colombia	3.9583	-77.3733	SAMEA104585055	Van Belleghem *et al.*[51]	3,571,047	67.27	2.32	WGS
zel.CS1030	CS001030	*Heliconius cydno ssp. zelinde*	Colombia	3.9583	-77.3733	SAMEA104585056	Van Belleghem *et al.*[51]	3,559,106	67.05	2.30	WGS
zel.CS1033	CS001033	*Heliconius cydno ssp. zelinde*	Colombia	3.9394	-77.3689	SAMEA104585057	Van Belleghem *et al.*[51]	3,644,672	68.61	2.38	WGS

SequenceID	EarthCapeID	Taxon Name	Country	Latitude	Longitude	Accession	Publication	Called sites	Geno-typed sites after filters (%)	Proport-ion of hetero-zygous genotypes (%)	Data Type (WGS...whole genome re-sequencing, CS...capture sequencing)
zel.CS1035	CS001035	*Heliconius cydno ssp. zelinde*	Colombia	3.9394	-77.3689	SAMEA104585058	Van Belleghem *et al.*[51]	3,830,391	72.15	2.33	WGS
zel.CS273	CS000273	*Heliconius cydno ssp. zelinde*	Colombia	3.9394	-77.3689	SAMEA104585059	Van Belleghem *et al.*[51]	3,841,140	72.49	2.14	WGS
zel.CS2262	CS002262	*Heliconius cydno ssp. zelinde*	Colombia	3.9394	-77.3689	SAMEA3670517	Enciso-Romero *et al.*[39]	3,249,828	61.23	2.29	WGS
PS17434	CAM017434	*Heliconius elevatus*	Ecuador	-1.2908	-77.8419	ERR3650582		3,420,534	64.04	2.92	CS
PS17411	CAM017411	*Heliconius elevatus*	Ecuador	-1.0157	-77.5975	ERR3650580		3,442,881	64.69	2.57	CS
PS17098	CAM017098	*Heliconius elevatus*	Ecuador	-1.0614	-77.6684	ERR3650528		3,403,379	63.73	2.89	CS
PS16984	CAM016984	*Heliconius elevatus*	Ecuador	-1.1878	-77.8311	ERR3652622		3,418,205	64.08	2.78	CS
PS16933	CAM016933	*Heliconius elevatus*	Ecuador	-1.1156	-77.7783	ERR3652620		3,409,748	63.99	2.68	CS
PS16781	CAM016781	*Heliconius elevatus*	Ecuador	-1.3382	-77.8354	ERR3650705		3,403,394	63.81	2.78	CS
PS16776	CAM016776	*Heliconius elevatus*	Ecuador	-1.2519	-77.8196	ERR3652585		3,413,732	63.89	2.95	CS
PS16626	CAM016626	*Heliconius elevatus*	Ecuador	-1.1156	-77.7783	ERR3650525		3,400,423	63.68	2.89	CS
PS16607	CAM016607	*Heliconius elevatus*	Ecuador	-1.1156	-77.7783	ERR3652618		3,423,138	64.13	2.85	CS
PS16596	CAM016596	*Heliconius elevatus*	Ecuador	-1.1156	-77.7783	ERR3650679		3,411,721	63.91	2.86	CS
PS16439	CAM016439	*Heliconius elevatus*	Ecuador	-1.1878	-77.8311	ERR3650587		3,375,448	63.24	2.84	CS
PS16216	CAM016216	*Heliconius elevatus*	Ecuador	-1.1156	-77.7783	ERR3652588		3,409,726	63.83	2.92	CS
PS16209	CAM016209	*Heliconius elevatus*	Ecuador	-1.1156	-77.7783	ERR3650585		3,393,480	63.77	2.55	CS
PS16207	CAM016207	*Heliconius elevatus*	Ecuador	-1.1156	-77.7783	ERR3652616		3,417,159	63.96	2.93	CS
CAM008861	CAM008861	*Heliconius hecale*	Panamá	7.7568	-77.6841	ERR3653121		3,467,332	65.32	2.30	CS
CAM008683	CAM008683	*Heliconius hecale*	Peru	-6.2897	-76.2289	ERR3653122		3,431,006	64.70	2.22	CS
heu.CS18	CS00CH18	*Heliconius heurippa*	Colombia	4.1750	-73.6781	ERR3653294		3,999,662	76.08	1.36	WGS
CS2014	CS002014	*Heliconius heurippa*	Colombia	4.1750	-73.6781	ERR3651489		3,876,614	73.70	1.42	CS
CS2420	CS002420	*Heliconius heurippa*	Colombia	4.1750	-73.6781	ERR3656290		3,852,612	73.37	1.24	CS
CS3394	CS003394	*Heliconius heurippa*	Colombia	4.1750	-73.6781	ERR3652415		3,843,551	73.12	1.35	CS
CS3424	CS003424	*Heliconius heurippa*	Colombia	4.1750	-73.6781	ERR3652412		3,837,343	73.07	1.26	CS
CS1510	CS001510	*Heliconius heurippa*	Colombia	4.1750	-73.6781	ERR3652409		3,838,220	72.95	1.45	CS
CS3392	CS003392	*Heliconius heurippa*	Colombia	4.1750	-73.6781	ERR3652403		3,846,603	73.24	1.26	CS
CS3843	CS003843	*Heliconius heurippa*	Colombia	3.5667	-74.0761	ERR3652584		3,849,937	73.16	1.45	CS
CS3391	CS003391	*Heliconius heurippa*	Colombia	4.1750	-73.6781	ERR3651475		3,880,909	73.93	1.22	CS
CS3696	CS003696	*Heliconius heurippa*	Colombia	4.1750	-73.6781	ERR3652406		3,812,208	72.53	1.35	CS
CS3393	CS003393	*Heliconius heurippa*	Colombia	4.1750	-73.6781	ERR3651487		3,864,380	73.45	1.44	CS
CS3423	CS003423	*Heliconius heurippa*	Colombia	4.1750	-73.6781	ERR3651427		3,887,467	73.92	1.40	CS
CS1353	CS001353	*Heliconius heurippa*	Colombia	4.1750	-73.6781	ERR3651492		3,877,700	73.79	1.32	CS
CS2012	CS002012	*Heliconius heurippa*	Colombia	4.1750	-73.6781	ERR3651482		3,880,389	74.07	1.01	CS
CS2618	CS002618	*Heliconius heurippa*	Colombia	4.1750	-73.6781	ERR3652418		3,837,340	73.02	1.33	CS
CS3007	CS003007	*Heliconius heurippa*	Colombia	4.1750	-73.6781	ERR3651479		3,858,645	73.33	1.45	CS
CS1352	CS001352	*Heliconius heurippa*	Colombia	4.1750	-73.6781	ERR3651483		3,858,710	73.53	1.18	CS
CS3844	CS003844	*Heliconius heurippa*	Colombia	3.5667	-74.0761	ERR3652577		3,848,242	73.03	1.60	CS
CS3397	CS003397	*Heliconius heurippa*	Colombia	4.1750	-73.6781	ERR3651478		3,881,108	73.43	1.89	CS

SequenceID	EarthCapeID	Taxon Name	Country	Latitude	Longitude	Accession	Publication	Called sites	Geno-typed sites after filters (%)	Proport-ion of hetero-zygous genotypes (%)	Data Type (WGS...whole genome re-sequencing, CS...capture sequencing)
CS2204	CS002204	*Heliconius heurippa*	Colombia	4.1750	-73.6781	ERR3652421		3,824,158	72.79	1.30	CS
heu.CS20	CS00CH21	*Heliconius heurippa*	Colombia	4.1750	-73.6781	SAMEA1045 85060	Van Belleghem *et al.*[51]	3,926,742	74.58	1.51	WGS
CS000CH9	CS000CH9	*Heliconius heurippa*	Colombia	4.1750	-73.6781	SAMEA1322 902	The Heliconius Consortium [44]; RAD	4,080,230	77.72	1.23	WGS
heu.STRI2	STRI_002	*Heliconius heurippa*	Colombia	4.1750	-73.6781	SAMEA3670 535	Wallbank *et al.*[36]	3,754,542	71.42	1.36	WGS
CS2013	CS002013	*Heliconius heurippa*	Colombia	4.1750	-73.6781	ERR3651485		3,883,113	73.32	2.09	CS
CAM002700	CAM002700	*Heliconius ismenius*	Panamá	9.1366	-79.7236	ERR3653123		3,268,849	62.18	1.36	CS
CAM002517	CAM002517	*Heliconius ismenius*	Panamá	9.1366	-79.7236	ERR3653124		3,396,394	64.28	1.86	CS
melP.HGC1	gen_ref	*Heliconius melpomene reference genome*	-	-	-	SAMN007943 86	The Heliconius Consortium [44]	4,418,717	84.76	0.53	WGS
agl.JM108	JM-09-108	*Heliconius melpomene ssp. aglaope*	Peru	-5.9103	-76.2256	SAMEA1919 251	Martin *et al.*[71]	3,705,462	69.73	2.42	WGS
agl.JM572	JM-11-572	*Heliconius melpomene ssp. aglaope*	Peru	-5.9717	-76.2319	SAMEA1919 259	Martin *et al.*[71]	3,777,573	71.08	2.43	WGS
agl.JM112	JM-09-112	*Heliconius melpomene ssp. aglaope*	Peru	-5.9103	-76.2256	SAMEA1919 264	Martin *et al.*[71]	3,785,847	71.25	2.41	WGS
agl.JM569	JM-11-569	*Heliconius melpomene ssp. aglaope*	Peru	-5.9717	-76.2319	SAMEA1919 274	Martin *et al.*[71]	3,823,969	71.97	2.40	WGS
MJ12-3414	MJ12-3414	*Heliconius melpomene ssp. amaryllis*	Peru	-6.4540	-76.3002	ERR3650522		3,537,813	66.92	1.91	CS
MJ12-3397	MJ12-3397	*Heliconius melpomene ssp. amaryllis*	Peru	-6.4528	-76.2862	ERR3650521		3,511,652	66.44	1.89	CS
MJ12-3302	MJ12-3302	*Heliconius melpomene ssp. amaryllis*	Peru	-6.4528	-76.2862	ERR3650487		3,586,055	67.84	1.90	CS
MJ12-3074	MJ12-3074	*Heliconius melpomene ssp. amaryllis*	Peru	-6.4528	-76.2862	ERR3650470		3,356,271	63.55	1.81	CS
MJ12-3371	MJ12-3371	*Heliconius melpomene ssp. amaryllis*	Peru	-6.4528	-76.2862	ERR3650513		3,513,745	66.47	1.90	CS
MJ12-3442	MJ12-3442	*Heliconius melpomene ssp. amaryllis*	Peru	-6.4547	-76.2994	ERR3650529		3,554,687	67.20	1.97	CS
MJ12-3416	MJ12-3416	*Heliconius melpomene ssp. amaryllis*	Peru	-6.4537	-76.2981	ERR3650524		3,491,823	65.99	2.00	CS
MJ12-3393	MJ12-3393	*Heliconius melpomene ssp. amaryllis*	Peru	-6.4528	-76.2862	ERR3650516		3,584,960	67.85	1.86	CS
MJ12-3417	MJ12-3417	*Heliconius melpomene ssp. amaryllis*	Peru	-6.4537	-76.2981	ERR3650526		3,547,133	67.04	1.99	CS
MJ12-3267	MJ12-3267	*Heliconius melpomene ssp. amaryllis*	Peru	-6.4528	-76.2862	ERR3656073		3,360,799	63.64	1.80	CS
MJ12-3474	MJ12-3474	*Heliconius melpomene ssp. amaryllis*	Peru	-6.4537	-76.2981	ERR3650584		3,575,687	67.61	1.94	CS
MJ12-3467	MJ12-3467	*Heliconius melpomene ssp. amaryllis*	Peru	-6.4528	-76.2862	ERR3650583		3,555,664	67.22	1.97	CS
MJ12-3482	MJ12-3482	*Heliconius melpomene ssp. amaryllis*	Peru	-6.4524	-76.2869	ERR3650586		3,568,507	67.46	1.97	CS
MJ12-3306	MJ12-3306	*Heliconius melpomene ssp. amaryllis*	Peru	-6.4572	-76.2986	ERR3650488		3,570,395	67.53	1.91	CS
MJ11-3018	MJ11-3018	*Heliconius melpomene ssp. amaryllis*	Peru	-6.4567	-76.2845	ERR3650480		3,409,538	64.54	1.85	CS
MJ12-3418	MJ12-3418	*Heliconius melpomene ssp. amaryllis*	Peru	-6.4537	-76.2981	ERR3650527		3,577,439	67.60	2.01	CS
MJ12-3585	MJ12-3585	*Heliconius melpomene ssp. amaryllis*	Peru	-6.4528	-76.2862	ERR3650472		3,428,919	64.91	1.83	CS

SequenceID	EarthCapeID	Taxon Name	Country	Latitude	Longitude	Accession	Publication	Called sites	Geno-typed sites after filters (%)	Proport-ion of hetero-zygous genotypes (%)	Data Type (WGS...whole genome re-sequencing, CS...capture sequencing)
MJ12-3466	MJ12-3466	*Heliconius melpomene ssp. amaryllis*	Peru	-6.4530	-76.2876	ERR3650581		3,580,238	67.72	1.92	CS
MJ12-3137	MJ12-3137	*Heliconius melpomene ssp. amaryllis*	Peru	-6.4547	-76.2994	ERR3650486		3,195,835	60.21	2.30	CS
MJ12-3394	MJ12-3394	*Heliconius melpomene ssp. amaryllis*	Peru	-6.4528	-76.2862	ERR3650518		3,426,831	64.84	1.89	CS
MJ12-3453	MJ12-3453	*Heliconius melpomene ssp. amaryllis*	Peru	-6.4537	-76.2981	ERR3650579		3,521,043	66.65	1.84	CS
MJ12-3392	MJ12-3392	*Heliconius melpomene ssp. amaryllis*	Peru	-6.4528	-76.2862	ERR3650515		3,585,104	67.79	1.94	CS
MJ11-3021	MJ11-3021	*Heliconius melpomene ssp. amaryllis*	Peru	-6.4555	-76.2843	ERR3650481		3,354,395	63.45	1.92	CS
MJ11-3024	MJ11-3024	*Heliconius melpomene ssp. amaryllis*	Peru	-6.4555	-76.2843	ERR3650483		3,399,058	64.35	1.83	CS
MJ11-3023	MJ11-3023	*Heliconius melpomene ssp. amaryllis*	Peru	-6.4555	-76.2843	ERR3650482		3,388,398	64.12	1.86	CS
MJ12-3483	MJ12-3483	*Heliconius melpomene ssp. amaryllis*	Peru	-6.4528	-76.2862	ERR3650471		3,398,807	64.32	1.87	CS
MJ12-3396	MJ12-3396	*Heliconius melpomene ssp. amaryllis*	Peru	-6.4528	-76.2862	ERR3650519		3,575,104	67.60	1.94	CS
MJ12-3130	MJ12-3130	*Heliconius melpomene ssp. amaryllis*	Peru	-6.4572	-76.2986	ERR3650485		3,475,149	65.79	1.83	CS
ama.MJ11-3188	MJ11-3188	*Heliconius melpomene ssp. amaryllis*	Peru	-5.6728	-77.7195	SAMEA104585061	Van Belleghem *et al.*[51]	3,432,288	64.83	2.05	WGS
ama.MJ11-3189	MJ11-3189	*Heliconius melpomene ssp. amaryllis*	Peru	-5.6728	-77.7195	SAMEA104585062	Van Belleghem *et al.*[51]	3,502,389	66.25	1.92	WGS
ama.MJ11-3202	MJ11-3202	*Heliconius melpomene ssp. amaryllis*	Peru	-5.6745	-77.6711	SAMEA104585063	Van Belleghem *et al.*[51]	3,365,693	63.43	2.28	WGS
ama.MJ12-3217	MJ12-3217	*Heliconius melpomene ssp. amaryllis*	Peru	-6.4547	-76.2994	SAMEA104585064	Van Belleghem *et al.*[51]	3,742,548	70.64	2.13	WGS
ama.MJ12-3258	MJ12-3258	*Heliconius melpomene ssp. amaryllis*	Peru	-6.4530	-76.2876	SAMEA104585065	Van Belleghem *et al.*[51]	3,648,380	68.72	2.32	WGS
ama.MJ12-3301	MJ12-3301	*Heliconius melpomene ssp. amaryllis*	Peru	-6.4528	-76.2862	SAMEA104585066	Van Belleghem *et al.*[51]	3,655,176	69.06	2.02	WGS
ama.JM160	JM-11-160	*Heliconius melpomene ssp. amaryllis*	Peru	-6.4645	-76.3514	SAMEA1919275	Martin *et al.*[71]	3,911,236	73.79	2.17	WGS
ama.JM216	JM-09-216	*Heliconius melpomene ssp. amaryllis*	Peru	-5.6745	-77.6748	SAMEA1919261	Martin *et al.*[71]	3,823,200	72.18	2.10	WGS
ama.JM48	JM-11-48	*Heliconius melpomene ssp. amaryllis*	Peru	-6.0960	-76.9774	SAMEA1919269	Martin *et al.*[71]	3,945,310	74.40	2.21	WGS
ama.JM293	JM-11-293	*Heliconius melpomene ssp. amaryllis*	Peru	-6.4667	-76.3347	SAMEA1919277	Martin *et al.*[71]	3,935,343	74.27	2.13	WGS
GM184_4	CAM019822	*Heliconius melpomene ssp. burchelli*	Brazil	-6.9639	-46.6799	ERR3652645		3,841,610	72.76	1.78	CS
GM184_3	CAM019821	*Heliconius melpomene ssp. burchelli*	Brazil	-6.9639	-46.6799	ERR3652643		3,852,678	72.93	1.84	CS
GM184_1	CAM019820	*Heliconius melpomene ssp. burchelli*	Brazil	-6.9639	-46.6799	ERR3652640		3,852,984	72.96	1.81	CS
s15N005	15N005	*Heliconius melpomene ssp. cythera*	Ecuador	0.1753	-78.9075	ERR3651486		4,051,459	76.58	1.98	CS
s15N006	15N006	*Heliconius melpomene ssp. cythera*	Ecuador	0.1937	-78.8587	ERR3651488		4,062,354	76.87	1.88	CS
s15N009	15N009	*Heliconius melpomene ssp. cythera*	Ecuador	0.1753	-78.9075	ERR3652194		4,097,078	77.41	2.02	CS
s15N022	15N022	*Heliconius melpomene ssp. cythera*	Ecuador	0.1850	-78.8530	ERR3656234		4,061,051	76.72	2.04	CS
14N594	14N594	*Heliconius melpomene ssp. cythera*	Ecuador	0.1659	-78.8882	ERR3651428		3,984,318	75.31	1.98	CS

SequenceID	EarthCapeID	Taxon Name	Country	Latitude	Longitude	Accession	Publication	Called sites	Geno-typed sites after filters (%)	Proport-ion of hetero-zygous genotypes (%)	Data Type (WGS...whole genome re-sequencing, CS...capture sequencing)
s15N008	15N008	*Heliconius melpomene ssp. cythera*	Ecuador	0.1850	-78.8530	ERR3651491		4,065,406	76.90	1.91	CS
s15N023	15N023	*Heliconius melpomene ssp. cythera*	Ecuador	0.1937	-78.8587	ERR3652351		4,074,163	77.15	1.81	CS
s15N020	15N020	*Heliconius melpomene ssp. cythera*	Ecuador	0.1850	-78.8530	ERR3652348		4,064,674	76.85	1.96	CS
s15N004	15N004	*Heliconius melpomene ssp. cythera*	Ecuador	0.1753	-78.9075	ERR3651484		4,077,502	77.11	1.93	CS
CAM002856_ERR1143591	CAM002856	*Heliconius melpomene ssp. cythera*	Ecuador	-0.3197	-79.3370	SAMEA3670542	Wallbank *et al.*[36]	3,223,375	60.67	2.39	WGS
CAM009116	CAM009116	*Heliconius melpomene ssp. ecuadoriensis*	Ecuador	-4.0439	-78.9861	ERR3652382		3,878,044	73.89	1.20	CS
CAM009119	CAM009119	*Heliconius melpomene ssp. ecuadoriensis*	Ecuador	-4.0439	-78.9861	ERR3652387		3,857,233	72.72	2.23	CS
CAM009118	CAM009118	*Heliconius melpomene ssp. ecuadoriensis*	Ecuador	-4.0439	-78.9861	ERR3652385		3,883,754	73.22	2.23	CS
CAM009115	CAM009115	*Heliconius melpomene ssp. ecuadoriensis*	Ecuador	-4.0439	-78.9861	ERR3652379		3,866,026	72.92	2.19	CS
CAM009114	CAM009114	*Heliconius melpomene ssp. ecuadoriensis*	Ecuador	-4.0439	-78.9861	ERR3652376		3,887,362	73.28	2.24	CS
CAM009113	CAM009113	*Heliconius melpomene ssp. ecuadoriensis*	Ecuador	-4.0439	-78.9861	ERR3651418		3,864,300	72.87	2.22	CS
CAM002432	CAM002432	*Heliconius melpomene ssp. ecuadoriensis*	Ecuador	-4.0653	-78.9587	ERR3651415		3,869,756	73.02	2.15	CS
CAM002430	CAM002430	*Heliconius melpomene ssp. ecuadoriensis*	Ecuador	-4.0653	-78.9587	ERR3652372		3,910,420	73.75	2.20	CS
CAM002417	CAM002417	*Heliconius melpomene ssp. ecuadoriensis*	Ecuador	-4.0653	-78.9587	ERR3651374		3,874,823	73.06	2.23	CS
CAM009112	CAM009112	*Heliconius melpomene ssp. ecuadoriensis*	Ecuador	-4.0439	-78.9861	ERR3653529 (SAMEA1322914)	The Heliconius Consortium[44]; RAD; SureSelect	3,872,864	73.07	2.17	CS
CS3447	CS003447	*Heliconius melpomene ssp. malleti*	Colombia	1.8033	-75.6553	ERR3652346		3,738,051	70.61	2.05	CS
CS3706	CS003706	*Heliconius melpomene ssp. malleti*	Colombia	1.8033	-75.6553	ERR3652358		3,791,670	71.98	1.56	CS
CS3542	CS003542	*Heliconius melpomene ssp. malleti*	Colombia	1.7506	-75.6319	ERR3652352		3,790,119	71.49	2.19	CS
CS3728	CS003728	*Heliconius melpomene ssp. malleti*	Colombia	1.8033	-75.6553	ERR3652364		3,779,425	71.29	2.18	CS
CS583	CS000583	*Heliconius melpomene ssp. malleti*	Colombia	1.8033	-75.6553	ERR3651426		3,675,883	69.34	2.18	CS
CS3109	CS003109	*Heliconius melpomene ssp. malleti*	Colombia	1.7108	-75.7089	ERR3652399		3,823,774	72.05	2.29	CS
CS3366	CS003366	*Heliconius melpomene ssp. malleti*	Colombia	1.7108	-75.7089	ERR3656292		3,791,556	71.50	2.21	CS
CS3541	CS003541	*Heliconius melpomene ssp. malleti*	Colombia	1.7506	-75.6319	ERR3652349		3,795,645	71.58	2.21	CS
CS3543	CS003543	*Heliconius melpomene ssp. malleti*	Colombia	1.7506	-75.6319	ERR3652355		3,816,480	71.98	2.20	CS
CS3815	CS003815	*Heliconius melpomene ssp. malleti*	Colombia	1.8033	-75.6553	ERR3652374		3,725,648	70.29	2.17	CS
CS3730	CS003730	*Heliconius melpomene ssp. malleti*	Colombia	1.8033	-75.6553	ERR3652368		3,780,839	71.51	1.92	CS
CS470	CS000470	*Heliconius melpomene ssp. malleti*	Colombia	1.8033	-75.6553	ERR3651419		3,627,455	68.43	2.18	CS
CS3709	CS003709	*Heliconius melpomene ssp. malleti*	Colombia	1.8033	-75.6553	ERR3652361		3,718,116	70.24	2.03	CS

SequenceID	EarthCapeID	Taxon Name	Country	Latitude	Longitude	Accession	Publication	Called sites	Geno-typed sites after filters (%)	Proport-ion of hetero-zygous genotypes (%)	Data Type (WGS...whole genome re-sequencing, CS...capture sequencing)
mal.CS1002	CS001002	*Heliconius melpomene ssp. malleti*	Colombia	1.8033	-75.6553	SAMEA1045 85067	Van Belleghem *et al.*[51]	3,648,672	68.78	2.24	WGS
mal.CS1011	CS001011	*Heliconius melpomene ssp. malleti*	Colombia	1.8033	-75.6553	SAMEA1045 85068	Van Belleghem *et al.*[51]	3,425,148	64.49	2.36	WGS
mal.CS1815	CS001815	*Heliconius melpomene ssp. malleti*	Colombia	1.8033	-75.6553	SAMEA1045 85069	Van Belleghem *et al.*[51]	3,439,461	64.91	2.14	WGS
mal.CS586	CS000586	*Heliconius melpomene ssp. malleti*	Colombia	1.8033	-75.6553	SAMEA1045 85071	Van Belleghem *et al.*[51]	3,350,546	63.08	2.37	WGS
mal.CS594	CS000594	*Heliconius melpomene ssp. malleti*	Colombia	1.8033	-75.6553	SAMEA1045 85072	Van Belleghem *et al.*[51]	3,889,131	73.23	2.36	WGS
mal.CS604	CS000604	*Heliconius melpomene ssp. malleti*	Colombia	1.8033	-75.6553	SAMEA1045 85073	Van Belleghem *et al.*[51]	3,785,826	71.33	2.30	WGS
mal.CS615	CS000615	*Heliconius melpomene ssp. malleti*	Colombia	1.8033	-75.6553	SAMEA1045 85074	Van Belleghem *et al.*[51]	3,614,972	68.10	2.31	WGS
mal.CS21	CS002311	*Heliconius melpomene ssp. malleti*	Colombia	1.8136	-75.6686	SAMEA3723 397	Enciso-Romero *et al.*[39]	3,918,174	73.70	2.46	WGS
mal.CS22	CS001286	*Heliconius melpomene ssp. malleti*	Colombia	1.6097	-75.6669	SAMEA3723 398	Enciso-Romero *et al.*[39]	3,899,819	73.41	2.39	WGS
mal.CS24	CS001321	*Heliconius melpomene ssp. malleti*	Colombia	1.7506	-75.6319	SAMEA3723 399	Enciso-Romero *et al.*[39]	3,845,940	72.38	2.41	WGS
PS17381	CAM017381	*Heliconius melpomene ssp. malleti*	Ecuador	-1.0983	-77.5839	ERR3652414		3,695,732	69.70	2.20	CS
PS17120	CAM017120	*Heliconius melpomene ssp. malleti*	Ecuador	-1.0916	-77.7200	ERR3652601		3,726,292	70.25	2.24	CS
CAM017091	CAM017091	*Heliconius melpomene ssp. malleti*	Ecuador	-1.0614	-77.6684	ERR3653410		3,579,661	67.51	2.20	CS
CAM017089	CAM017089	*Heliconius melpomene ssp. malleti*	Ecuador	-1.0614	-77.6684	ERR3653411		3,593,930	67.74	2.26	CS
PS17073	CAM017073	*Heliconius melpomene ssp. malleti*	Ecuador	-1.1684	-77.7811	ERR3652598		3,719,819	70.14	2.23	CS
CAM017070	CAM017070	*Heliconius melpomene ssp. malleti*	Ecuador	-1.1684	-77.7811	ERR3653412		3,603,436	67.92	2.26	CS
CAM017069	CAM017069	*Heliconius melpomene ssp. malleti*	Ecuador	-1.1684	-77.7811	ERR3653413		3,611,832	68.07	2.27	CS
CAM017064	CAM017064	*Heliconius melpomene ssp. malleti*	Ecuador	-1.1684	-77.7811	ERR3653414		3,579,409	67.50	2.21	CS
PS17049	CAM017049	*Heliconius melpomene ssp. malleti*	Ecuador	-1.1684	-77.7811	ERR3652594		3,701,041	69.80	2.21	CS
PS17030	CAM017030	*Heliconius melpomene ssp. malleti*	Ecuador	-1.0916	-77.7200	ERR3652592		3,706,478	69.91	2.19	CS
CAM016956	CAM016956	*Heliconius melpomene ssp. malleti*	Ecuador	-1.1156	-77.7783	ERR3653415		3,557,390	67.11	2.17	CS
CAM016955	CAM016955	*Heliconius melpomene ssp. malleti*	Ecuador	-1.1156	-77.7783	ERR3653416		3,624,964	68.34	2.24	CS
CAM016954	CAM016954	*Heliconius melpomene ssp. malleti*	Ecuador	-1.1156	-77.7783	ERR3653417		3,586,392	67.65	2.19	CS
PS16772	CAM016772	*Heliconius melpomene ssp. malleti*	Ecuador	-1.2519	-77.8196	ERR3652411		3,697,550	69.70	2.25	CS
PS16766	CAM016766	*Heliconius melpomene ssp. malleti*	Ecuador	-1.2519	-77.8196	ERR3652408		3,730,622	70.35	2.21	CS
CAM016611	CAM016611	*Heliconius melpomene ssp. malleti*	Ecuador	-1.1156	-77.7783	ERR3653419		3,635,301	68.54	2.23	CS
CAM016610	CAM016610	*Heliconius melpomene ssp. malleti*	Ecuador	-1.1156	-77.7783	ERR3653420		3,606,744	68.01	2.22	CS
CAM016609	CAM016609	*Heliconius melpomene ssp. malleti*	Ecuador	-1.1156	-77.7783	ERR3653421		3,592,637	67.74	2.22	CS
CAM016606	CAM016606	*Heliconius melpomene ssp. malleti*	Ecuador	-1.1156	-77.7783	ERR3653422		3,575,042	67.39	2.26	CS

SequenceID	EarthCapeID	Taxon Name	Country	Latitude	Longitude	Accession	Publication	Called sites	Geno-typed sites after filters (%)	Proport-ion of hetero-zygous genotypes (%)	Data Type (WGS…whole genome re-sequencing, CS…capture sequencing)
CAM016599	CAM016599	*Heliconius melpomene ssp. malleti*	Ecuador	-1.1156	-77.7783	ERR3653423		3,613,904	68.14	2.22	CS
CAM016549	CAM016549	*Heliconius melpomene ssp. malleti*	Ecuador	-1.0614	-77.6684	ERR3653424		3,623,370	68.30	2.25	CS
CAM016547	CAM016547	*Heliconius melpomene ssp. malleti*	Ecuador	-1.0614	-77.6684	ERR3653425		3,645,314	68.69	2.28	CS
CAM016546	CAM016546	*Heliconius melpomene ssp. malleti*	Ecuador	-1.0614	-77.6684	ERR3653427		3,615,315	68.10	2.32	CS
CAM016544	CAM016544	*Heliconius melpomene ssp. malleti*	Ecuador	-1.0614	-77.6684	ERR3653428		3,593,545	67.75	2.24	CS
CAM016541	CAM016541	*Heliconius melpomene ssp. malleti*	Ecuador	-1.0614	-77.6684	ERR3653429		3,604,024	67.93	2.26	CS
PS16465	CAM016465	*Heliconius melpomene ssp. malleti*	Ecuador	-1.1878	-77.8311	ERR3652405		3,699,981	69.74	2.26	CS
CAM016267	CAM016267	*Heliconius melpomene ssp. malleti*	Ecuador	-1.2510	-77.6989	ERR3653409		3,627,980	68.39	2.24	CS
CAM016224	CAM016224	*Heliconius melpomene ssp. malleti*	Ecuador	-1.1156	-77.7783	ERR3653430		3,533,002	66.66	2.16	CS
PS16144	CAM016144	*Heliconius melpomene ssp. malleti*	Ecuador	-1.4161	-77.7290	ERR3652402		3,711,697	70.00	2.20	CS
CS1456	CS001456	*Heliconius melpomene ssp. melpomene*	Colombia	4.1789	-73.6494	ERR3652363		4,016,701	75.81	2.13	CS
CS1055	CS001055	*Heliconius melpomene ssp. melpomene*	Colombia	4.0052	-73.7775	ERR3652360		4,016,080	75.83	2.09	CS
CS1669	CS001669	*Heliconius melpomene ssp. melpomene*	Colombia	4.2133	-73.8028	ERR3652384		3,985,424	75.67	1.55	CS
CS1120	CS001120	*Heliconius melpomene ssp. melpomene*	Colombia	4.0052	-73.7775	ERR3652388		4,028,453	76.02	2.14	CS
CS1057	CS001057	*Heliconius melpomene ssp. melpomene*	Colombia	4.2133	-73.8028	ERR3652381		4,003,690	75.55	2.15	CS
CS1058	CS001058	*Heliconius melpomene ssp. melpomene*	Colombia	4.2133	-73.8028	ERR3652366		3,978,857	75.20	2.00	CS
CS3255	CS003255	*Heliconius melpomene ssp. melpomene*	Colombia	4.2133	-73.8028	ERR3656291		4,015,755	75.89	2.00	CS
CS3758	CS003758	*Heliconius melpomene ssp. melpomene*	Colombia	4.2133	-73.8028	ERR3652400		4,005,712	75.68	2.03	CS
CS1059	CS001059	*Heliconius melpomene ssp. melpomene*	Colombia	4.2133	-73.8028	ERR3652354		4,007,649	75.75	1.99	CS
CS1060	CS001060	*Heliconius melpomene ssp. melpomene*	Colombia	4.2133	-73.8028	ERR3656289		3,985,438	75.49	1.77	CS
CS1061	CS001061	*Heliconius melpomene ssp. melpomene*	Colombia	4.2133	-73.8028	ERR3652357		4,012,207	75.76	2.08	CS
CS3757	CS003757	*Heliconius melpomene ssp. melpomene*	Colombia	4.2133	-73.8028	ERR3652397		4,000,033	75.56	2.05	CS
CS1062	CS001062	*Heliconius melpomene ssp. melpomene*	Colombia	4.2133	-73.8028	ERR3652378		4,016,089	76.03	1.83	CS
melC.CS3	CS000CM3	*Heliconius melpomene ssp. melpomene*	Colombia	4.2133	-73.8028	SAMEA3723393	Martin *et al.*[71]	4,161,140	78.65	1.99	WGS
melC.CS6	CS000CM6	*Heliconius melpomene ssp. melpomene*	Colombia	5.6169	-72.3000	SAMEA3723394	Martin *et al.*[71]	4,210,293	79.39	2.22	WGS
melC.CS25	CS000CM4	*Heliconius melpomene ssp. melpomene*	Colombia	4.2133	-73.8028	SAMEA3723400	Martin *et al.*[71]	4,102,616	77.43	2.13	WGS
melC.CS26	CS000CM5	*Heliconius melpomene ssp. melpomene*	Colombia	4.2133	-73.8028	SAMEA3723401	Martin *et al.*[71]	4,073,969	77.06	1.91	WGS
melC.CS27	CS00CM10	*Heliconius melpomene ssp. melpomene*	Colombia	5.6169	-72.3000	SAMEA3723402	Martin *et al.*[71]	4,198,256	79.16	2.22	WGS
melG.CAM8215	CAM008215	*Heliconius melpomene ssp. melpomene*	French Guiana	4.7890	-52.4040	SAMEA104585079		1,228,623	23.43	1.12	WGS

SequenceID	EarthCapeID	Taxon Name	Country	Latitude	Longitude	Accession	Publication	Called sites	Geno-typed sites after filters (%)	Proport-ion of hetero-zygous genotypes (%)	Data Type (WGS...whole genome re-sequencing, CS...capture sequencing)
melG.CAM1349	CAM001349	*Heliconius melpomene ssp. melpomene*	French Guiana	4.9632	-52.4200	SAMEA104585075	Van Belleghem *et al.*[51]	3,507,398	66.40	1.83	WGS
melG.CAM1422	CAM001422	*Heliconius melpomene ssp. melpomene*	French Guiana	4.9632	-52.4200	SAMEA104585076	Van Belleghem *et al.*[51]	3,217,943	60.93	1.82	WGS
melG.CAM2035	CAM002035	*Heliconius melpomene ssp. melpomene*	French Guiana	4.9632	-52.4200	SAMEA104585077	Van Belleghem *et al.*[51]	3,687,629	69.79	1.86	WGS
melG.CAM8171	CAM008171	*Heliconius melpomene ssp. melpomene*	French Guiana	4.9632	-52.4200	SAMEA104585078	Van Belleghem *et al.*[51]	3,466,269	65.62	1.84	WGS
melG.CAM8216	CAM008216	*Heliconius melpomene ssp. melpomene*	French Guiana	4.7890	-52.4040	SAMEA104585080	Van Belleghem *et al.*[51]	3,654,987	69.19	1.84	WGS
melG.CAM8218	CAM008218	*Heliconius melpomene ssp. melpomene*	French Guiana	4.7890	-52.4040	SAMEA104585081	Van Belleghem *et al.*[51]	3,464,056	65.56	1.86	WGS
melG.CJ9316	CAM009316	*Heliconius melpomene ssp. melpomene*	French Guiana	4.9632	-52.4200	SAMEA1919252	Martin *et al.*[71]	3,238,215	61.34	1.77	WGS
melG.CJ9317	CAM009317	*Heliconius melpomene ssp. melpomene*	French Guiana	4.9632	-52.4200	SAMEA1919267	Martin *et al.*[71]	3,798,559	71.89	1.86	WGS
melG.CJ9315	CAM009315	*Heliconius melpomene ssp. melpomene*	French Guiana	4.9632	-52.4200	SAMEA1919270	Martin *et al.*[71]	3,349,594	63.43	1.81	WGS
melG.CJ13435	CAM013435	*Heliconius melpomene ssp. melpomene*	French Guiana	4.9632	-52.4200	SAMEA1919276	Martin *et al.*[71]	3,827,918	72.44	1.87	WGS
nan.CAM14673	CAM014673	*Heliconius melpomene ssp. melpomene*	Panamá	8.6136	-78.1398	SAMEA104585082		4,131,231	77.88	2.25	WGS
CAM014662	CAM014662	*Heliconius melpomene ssp. melpomene*	Panamá	8.6136	-78.1398	ERR3652652		4,102,605	77.51	2.03	CS
CAM014654	CAM014654	*Heliconius melpomene ssp. melpomene*	Panamá	8.6136	-78.1398	ERR3652647		4,110,765	77.69	2.00	CS
CAM008956	CAM008956	*Heliconius melpomene ssp. melpomene*	Panamá	7.6362	-78.1897	ERR3652656		4,112,268	77.60	2.15	CS
CAM008954	CAM008954	*Heliconius melpomene ssp. melpomene*	Panamá	7.6362	-78.1897	ERR3652649		4,109,799	77.65	2.02	CS
CAM008887	CAM008887	*Heliconius melpomene ssp. melpomene*	Panamá	7.7568	-77.6841	ERR3652654		4,116,541	77.77	2.04	CS
melP.CJ18038	CAM018038	*Heliconius melpomene ssp. melpomene*	Panamá	8.6136	-78.1398	SAMEA1919255	Martin *et al.*[71]	4,127,165	77.92	2.09	WGS
melP.CJ18097	CAM018097	*Heliconius melpomene ssp. melpomene*	Panamá	8.2797	-77.8098	SAMEA1919258	Martin *et al.*[71]	2,597,633	49.02	2.13	WGS
CS2174	CS002174	*Heliconius melpomene ssp. melpomene*	Venezuela	7.5878	-72.1275	ERR3652375		4,020,251	75.92	2.08	CS
CAM013819	CAM013819	*Heliconius melpomene ssp. meriana*	French Guiana	3.6883	-54.0825	ERR3653533 (SAMEA3670554)	Martin *et al.*[71]	3,812,465	72.12	1.91	CS
CAM013715	CAM013715	*Heliconius melpomene ssp. meriana*	French Guiana	3.6883	-54.0825	ERR3653532 (SAMEA3670555)	Martin *et al.*[71]	3,828,840	72.51	1.79	CS
SHM21055	CAM021055	*Heliconius melpomene ssp. meriana*	Suriname	5.0818	-54.9791	ERR3652641		3,874,135	73.31	1.88	CS
RW12	CAM019804	*Heliconius melpomene ssp. 'meriana'*	England	52.1905	-1.7037	ERR3653118		3,860,439	73.04	1.88	CS
RW9	CAM019803	*Heliconius melpomene ssp. 'meriana'*	England	52.1905	-1.7037	ERR3653119		3,814,479	72.17	1.89	CS
RW5	CAM019801	*Heliconius melpomene ssp. 'meriana'*	England	52.1905	-1.7037	ERR3653120		3,760,066	71.32	1.64	CS
RW4	CAM019800	*Heliconius melpomene ssp. 'meriana'*	England	52.1905	-1.7037	ERR3652642		3,884,713	73.88	1.38	CS
nan.183-18	CAM019828	*Heliconius melpomene ssp. nanna*	Brazil	-3.8692	-41.0192	ERR3653304		3,947,974	74.67	1.92	WGS
nan.183-16	CAM019827	*Heliconius melpomene ssp. nanna*	Brazil	-3.8692	-41.0192	ERR3653393		3,930,012	74.39	1.85	WGS
nan.183-14	CAM019826	*Heliconius melpomene ssp. nanna*	Brazil	-3.8692	-41.0192	ERR3653400		3,625,799	68.79	1.61	WGS

SequenceID	EarthCapeID	Taxon Name	Country	Latitude	Longitude	Accession	Publication	Called sites	Geno-typed sites after filters (%)	Proport-ion of hetero-zygous genotypes (%)	Data Type (WGS...whole genome re-sequencing, CS...capture sequencing)
nan.183-11	CAM019825	*Heliconius melpomene ssp. nanna*	Brazil	-3.8692	-41.0192	ERR3653404		3,207,112	60.92	1.49	WGS
nan.183-10	CAM019824	*Heliconius melpomene ssp. nanna*	Brazil	-3.8692	-41.0192	ERR3653408		3,844,584	72.75	1.88	WGS
GM209_26	CAM019808	*Heliconius melpomene ssp. nanna*	Brazil	-15.4201	-39.4964	ERR3652648		3,860,711	74.03	0.56	CS
GM183_19	CAM019807	*Heliconius melpomene ssp. nanna*	Brazil	-3.8692	-41.0192	ERR3652646		3,707,040	70.20	1.80	CS
GM183_15	CAM019806	*Heliconius melpomene ssp. nanna*	Brazil	-3.8692	-41.0192	ERR3652644		3,885,848	73.57	1.83	CS
MK000014	MK000014	*Heliconius melpomene ssp. nanna*	Brazil	-19.0984	-40.1862	SAMN04407968	Zhang *et al.*[45]	3,301,446	63.27	0.63	WGS
MK000062	MK000062	*Heliconius melpomene ssp. nanna*	Brazil	-19.0984	-40.1862	ERR3653534; (SAMN04407969)	Zhang *et al.*[45]	3,845,064	73.68	0.63	CS
MK000063	MK000063	*Heliconius melpomene ssp. nanna*	Brazil	-19.0984	-40.1862	SAMN04407970	Zhang *et al.*[45]	3,710,880	71.16	0.56	WGS
MK000064	MK000064	*Heliconius melpomene ssp. nanna*	Brazil	-19.0984	-40.1862	SAMN04407971	Zhang *et al.*[45]	3,984,097	76.34	0.64	WGS
PS17613	CAM017613	*Heliconius melpomene ssp. plesseni*	Ecuador	-1.4224	-78.1729	ERR3652353		3,868,444	73.25	1.81	CS
PS17373	CAM017373	*Heliconius melpomene ssp. plesseni*	Ecuador	-1.4600	-78.0728	ERR3652365		3,897,348	73.57	2.11	CS
PS17363	CAM017363	*Heliconius melpomene ssp. plesseni*	Ecuador	-1.4600	-78.0728	ERR3652362		3,888,631	73.47	2.02	CS
PS17357	CAM017357	*Heliconius melpomene ssp. plesseni*	Ecuador	-1.4600	-78.0728	ERR3652359		3,896,339	73.61	2.04	CS
PS16913	CAM016913	*Heliconius melpomene ssp. plesseni*	Ecuador	-1.4371	-78.1229	ERR3650523		3,839,335	72.55	2.01	CS
PS16910	CAM016910	*Heliconius melpomene ssp. plesseni*	Ecuador	-1.4371	-78.1229	ERR3650520		3,842,150	72.59	2.03	CS
PS16691	CAM016691	*Heliconius melpomene ssp. plesseni*	Ecuador	-1.3980	-78.1781	ERR3652356		3,862,444	73.00	2.00	CS
PS16004	CAM016004	*Heliconius melpomene ssp. plesseni*	Ecuador	-1.4224	-78.1729	ERR3652579		3,859,802	72.95	2.00	CS
PS16003	CAM016003	*Heliconius melpomene ssp. plesseni*	Ecuador	-1.4224	-78.1729	ERR3656232		3,879,223	73.31	2.00	CS
PS16001	CAM016001	*Heliconius melpomene ssp. plesseni*	Ecuador	-1.4224	-78.1729	ERR3652350		3,902,878	73.92	1.79	CS
ple.CJ9156	CAM009156	*Heliconius melpomene ssp. plesseni*	Ecuador	-1.3980	-78.1781	SAMEA3670556	Martin *et al.*[71]	2,789,820	52.74	1.96	WGS
ple.CJ16293	CAM016293	*Heliconius melpomene ssp. plesseni*	Ecuador	-1.4600	-78.0728	SAMEA3670557	Martin *et al.*[71]	2,610,868	49.33	2.02	WGS
ros.CAM1841	CAM001841	*Heliconius melpomene ssp. rosina*	Panamá	9.0760	-79.6590	SAMEA104585083	Van Belleghem *et al.*[51]	3,937,058	74.32	2.11	WGS
ros.CAM1880	CAM001880	*Heliconius melpomene ssp. rosina*	Panamá	9.0760	-79.6590	SAMEA104585084	Van Belleghem *et al.*[51]	3,970,697	74.99	2.07	WGS
ros.CAM2045	CAM002045	*Heliconius melpomene ssp. rosina*	Panamá	9.1103	-79.6907	SAMEA104585085	Van Belleghem *et al.*[51]	4,105,191	77.53	2.06	WGS
ros.CAM2059	CAM002059	*Heliconius melpomene ssp. rosina*	Panamá	9.1103	-79.6907	SAMEA104585086	Van Belleghem *et al.*[51]	4,154,613	78.51	2.01	WGS
ros.CAM2519	CAM002519	*Heliconius melpomene ssp. rosina*	Panamá	9.0109	-79.5477	SAMEA104585087	Van Belleghem *et al.*[51]	4,157,116	78.45	2.14	WGS
ros.CAM2552	CAM002552	*Heliconius melpomene ssp. rosina*	Panamá	9.0109	-79.5477	SAMEA104585088	Van Belleghem *et al.*[51]	3,520,446	66.52	2.02	WGS
ros.CJ2071	CAM002071	*Heliconius melpomene ssp. rosina*	Panamá	9.1103	-79.6907	SAMEA1919257	Martin *et al.*[71]	4,089,428	77.27	2.02	WGS

SequenceID	EarthCapeID	Taxon Name	Country	Latitude	Longitude	Accession	Publication	Called sites	Geno-typed sites after filters (%)	Proport-ion of hetero-zygous genotypes (%)	Data Type (WGS...whole genome re-sequencing, CS...capture sequencing)
ros.CJ533	CAM000533	*Heliconius melpomene ssp. rosina*	Panamá	9.1103	-79.6907	SAMEA1919260	Martin *et al.*[71]	3,442,199	65.09	1.94	WGS
ros.CJ531	CAM000531	*Heliconius melpomene ssp. rosina*	Panamá	9.1103	-79.6907	SAMEA1919271	Martin *et al.*[71]	3,556,153	67.21	1.99	WGS
ros.CJ546	CAM000546	*Heliconius melpomene ssp. rosina*	Panamá	9.1253	-79.6932	SAMEA1919279	Martin *et al.*[71]	3,445,216	65.14	1.96	WGS
CS32533	CS032533	*Heliconius melpomene ssp. vicina*	Colombia	-4.1003	-70.0417	ERR3652420		3,927,898	74.07	2.21	CS
CS31744	CS031744	*Heliconius melpomene ssp. vicina*	Colombia	-4.1334	-69.9415	ERR3652417		3,957,710	74.58	2.29	CS
CS31755	CS031755	*Heliconius melpomene ssp. vicina*	Colombia	-4.1334	-69.9415	ERR3656233		3,932,967	74.13	2.26	CS
vul.CS3603	CS003603	*Heliconius melpomene ssp. vulcanus*	Colombia	3.5175	-76.7572	SAMEA104585091	Van Belleghem *et al.*[51]	4,205,795	79.50	1.98	WGS
vul.CS3605	CS003605	*Heliconius melpomene ssp. vulcanus*	Colombia	3.5175	-76.7572	SAMEA104585092	Van Belleghem *et al.*[51]	4,193,786	79.25	2.01	WGS
vul.CS3606	CS003606	*Heliconius melpomene ssp. vulcanus*	Colombia	3.5175	-76.7572	SAMEA104585093	Van Belleghem *et al.*[51]	4,162,523	78.55	2.14	WGS
vul.CS3612	CS003612	*Heliconius melpomene ssp. vulcanus*	Colombia	3.5175	-76.7572	SAMEA104585094	Van Belleghem *et al.*[51]	4,201,547	79.77	1.54	WGS
vul.CS3614	CS003614	*Heliconius melpomene ssp. vulcanus*	Colombia	3.5175	-76.7572	SAMEA104585095	Van Belleghem *et al.*[51]	3,861,940	72.92	2.09	WGS
vul.CS3615	CS003615	*Heliconius melpomene ssp. vulcanus*	Colombia	3.5175	-76.7572	SAMEA104585096	Van Belleghem *et al.*[51]	3,717,208	70.41	1.78	WGS
vul.CS3617	CS003617	*Heliconius melpomene ssp. vulcanus*	Colombia	3.5175	-76.7572	SAMEA104585097	Van Belleghem *et al.*[51]	3,805,245	71.87	2.06	WGS
vul.CS3618	CS003618	*Heliconius melpomene ssp. vulcanus*	Colombia	3.5175	-76.7572	SAMEA104585098	Van Belleghem *et al.*[51]	3,820,701	72.46	1.66	WGS
vul.CS3621	CS003621	*Heliconius melpomene ssp. vulcanus*	Colombia	3.5175	-76.7572	SAMEA104585099	Van Belleghem *et al.*[51]	3,742,085	70.61	2.15	WGS
vul.CS10	CS000710	*Heliconius melpomene ssp. vulcanus*	Colombia	3.9000	-76.6325	SAMEA3723391	Enciso-Romero *et al.*[39]; Martin *et al.*[71]	4,255,149	80.43	1.98	WGS
vul.CJ14632	CAM014632	*Heliconius melpomene ssp. vulcanus*	Panamá	8.6136	-78.1398	SAMEA3670560	Wallbank *et al.*[36]	2,402,825	45.39	2.03	WGS
MJ12-3636	MJ12-3636	*Heliconius melpomene ssp. xenoclea*	Peru	-11.0338	-75.4091	ERR3650418		3,516,391	66.53	1.89	CS
MJ12-3653	MJ12-3653	*Heliconius melpomene ssp. xenoclea*	Peru	-11.0338	-75.4091	ERR3650415		3,659,935	69.21	1.93	CS
MJ12-3605	MJ12-3605	*Heliconius melpomene ssp. xenoclea*	Peru	-11.1745	-75.4035	ERR3650416		3,608,285	68.26	1.90	CS
MJ12-3648	MJ12-3648	*Heliconius melpomene ssp. xenoclea*	Peru	-11.0338	-75.4091	ERR3650388		3,603,354	68.16	1.92	CS
MJ12-3647	MJ12-3647	*Heliconius melpomene ssp. xenoclea*	Peru	-11.0446	-75.4133	ERR3650469		3,538,622	66.93	1.92	CS
MJ12-3763	MJ12-3763	*Heliconius melpomene ssp. xenoclea*	Peru	-11.0338	-75.4091	ERR3650414		3,584,335	67.83	1.86	CS
MJ12-3651	MJ12-3651	*Heliconius melpomene ssp. xenoclea*	Peru	-11.0338	-75.4091	ERR3650387		3,399,714	64.38	1.80	CS
MJ12-3608	MJ12-3608	*Heliconius melpomene ssp. xenoclea*	Peru	-11.1745	-75.4035	ERR3650386		3,379,925	64.01	1.80	CS
MJ12-3606	MJ12-3606	*Heliconius melpomene ssp. xenoclea*	Peru	-11.1745	-75.4035	ERR3650417		3,527,682	66.74	1.89	CS
MJ12-3638	MJ12-3638	*Heliconius melpomene ssp. xenoclea*	Peru	-11.0364	-75.4080	ERR3650468		3,593,153	67.99	1.88	CS
nu_sil.MJ09-4125	MJ09-4125	*Heliconius numata ssp. numata*	French Guiana	4.0833	-52.6753	SAMEA3888884	Nadeau *et al.*[30]	3,429,209	64.05	3.15	WGS
nu_sil.MJ09-4184	MJ09-4184	*Heliconius numata ssp. silvana*	French Guiana	4.0833	-52.6753	SAMEA3888889	Nadeau *et al.*[30]	3,514,463	65.52	3.33	WGS

SequenceID	EarthCapeID	Taxon Name	Country	Latitude	Longitude	Accession	Publication	Called sites	Geno-typed sites after filters (%)	Proport-ion of hetero-zygous genotypes (%)	Data Type (WGS...whole genome re-sequencing, CS...capture sequencing)
nu_sil.MJ05-1240	JM-05-1240	*Heliconius numata ssp. silvana*	Peru	-6.4782	-76.3258	SAMEA3888886	Nadeau *et al.*[30]	3,133,564	39.94	2.93	WGS
nu_sil.MJ05-1271	JM-05-1271	*Heliconius numata ssp. silvana*	Peru	-6.4782	-76.3258	SAMEA3888887	Nadeau *et al.*[30]	3,383,291	63.11	3.28	WGS
nu_sil.MJ05-124	MJ05-124	*Heliconius numata ssp. silvana*	Peru	-6.4782	-76.3258	SAMEA3888888	Nadeau *et al.*[30]	3,336,988	62.22	3.32	WGS
MK000516	MK000516	*Heliconius pachinus*	Costa Rica	9.8500	-84.3167	SAMN02384468	Kronforst *et al.*[49]	3,399,813	64.39	1.78	WGS
CAM008062	CAM008062	*Heliconius pachinus*	Panamá	8.8388	-82.7148	ERR3652658		3,803,511	72.07	1.75	CS
CAM008036	CAM008036	*Heliconius pachinus*	Panamá	8.8388	-82.7148	ERR3653117		3,813,469	72.02	2.07	CS
CAM008034	CAM008034	*Heliconius pachinus*	Panamá	8.8388	-82.7148	ERR3653097		3,810,903	72.01	2.02	CS
CAM008033	CAM008033	*Heliconius pachinus*	Panamá	8.8388	-82.7148	ERR3652657		3,789,692	71.80	1.75	CS
CAM008032	CAM008032	*Heliconius pachinus*	Panamá	8.8388	-82.7148	ERR3652659		3,812,716	72.26	1.73	CS
CAM008023	CAM008023	*Heliconius pachinus*	Panamá	8.8388	-82.7148	ERR3653098		3,782,656	71.58	1.87	CS
CAM008022	CAM008022	*Heliconius pachinus*	Panamá	8.8388	-82.7148	ERR3652661		3,824,865	72.43	1.81	CS
CAM008019	CAM008019	*Heliconius pachinus*	Panamá	8.8388	-82.7148	ERR3652660		3,800,262	71.62	2.28	CS
CAM008035	CAM008035	*Heliconius pachinus*	Panamá	8.8388	-82.7148	ERR3653536	Wallbank *et al.*[36]	3,804,700	72.10	1.73	CS
ser.JM202	JM-09-202	*Heliconius pardalinus ssp. sergestus*	Peru	-6.4667	-76.3347	SAMEA1919268	Martin *et al.*[71]	3,723,595	71.15	0.92	WGS
par.JM371	JM-09-371	*Heliconius pardalinus ssp. ssp.nov.P*	Peru	-8.3422	-74.5922	SAMEA1919253	Martin *et al.*[71]	3,672,441	68.65	3.06	WGS
CS2394	CS002394	*Heliconius timareta ssp. florencia*	Colombia	1.7108	-75.7089	ERR3652193		3,794,165	71.82	1.84	CS
CS2343	CS002343	*Heliconius timareta ssp. florencia*	Colombia	1.8136	-75.6686	ERR3650514		3,878,536	73.42	1.83	CS
CS2333	CS002333	*Heliconius timareta ssp. florencia*	Colombia	1.7108	-75.7089	ERR3650498		3,835,744	72.64	1.79	CS
CS2327	CS002327	*Heliconius timareta ssp. florencia*	Colombia	1.7108	-75.7089	ERR3650495		3,849,177	72.91	1.78	CS
CS419	CS000419	*Heliconius timareta ssp. florencia*	Colombia	1.8033	-75.6553	ERR3651477		3,736,703	70.74	1.83	CS
CS399	CS000399	*Heliconius timareta ssp. florencia*	Colombia	1.8033	-75.6553	ERR3651476		3,698,884	70.15	1.66	CS
CS2349	CS002349	*Heliconius timareta ssp. florencia*	Colombia	1.7108	-75.7089	ERR3650517		3,836,299	72.64	1.81	CS
CS1009	CS001009	*Heliconius timareta ssp. florencia*	Colombia	1.8033	-75.6553	ERR3650489		3,847,891	72.85	1.82	CS
CS473	CS000473	*Heliconius timareta ssp. florencia*	Colombia	1.8033	-75.6553	ERR3650492		3,828,399	72.39	1.95	CS
CS2354	CS002354	*Heliconius timareta ssp. florencia*	Colombia	1.7108	-75.7089	ERR3652347		3,871,665	73.42	1.67	CS
flo.CS12	CS002395	*Heliconius timareta ssp. florencia*	Colombia	1.7097	-75.6976	SAMEA104585100	Van Belleghem *et al.*[51]	3,897,565	73.76	1.87	WGS
flo.CS13	CS002402	*Heliconius timareta ssp. florencia*	Colombia	1.7097	-75.6976	SAMEA104585101	Van Belleghem *et al.*[51]	3,872,164	73.23	1.93	WGS
flo.CS14	CS002403	*Heliconius timareta ssp. florencia*	Colombia	1.7097	-75.6976	SAMEA104585102	Van Belleghem *et al.*[51]	3,910,873	73.99	1.89	WGS
flo.CS15	CS002406	*Heliconius timareta ssp. florencia*	Colombia	1.7097	-75.6976	SAMEA104585103	Van Belleghem *et al.*[51]	3,904,353	73.79	1.99	WGS
flo.CS2337	CS002337	*Heliconius timareta ssp. florencia*	Colombia	1.7108	-75.7089	SAMEA104585104	Van Belleghem *et al.*[51]	3,681,700	69.62	1.94	WGS
flo.CS2338	CS002338	*Heliconius timareta ssp. florencia*	Colombia	1.7108	-75.7089	SAMEA104585105	Van Belleghem *et al.*[51]	3,723,102	70.48	1.83	WGS
flo.CS2341	CS002341	*Heliconius timareta ssp. florencia*	Colombia	1.8136	-75.6686	SAMEA104585106	Van Belleghem *et al.*[51]	3,579,194	67.68	1.94	WGS
flo.CS2350	CS002350	*Heliconius timareta ssp. florencia*	Colombia	1.7108	-75.7089	SAMEA104585107	Van Belleghem *et al.*[51]	3,741,211	70.75	1.93	WGS
flo.CS2358	CS002358	*Heliconius timareta ssp. florencia*	Colombia	1.7108	-75.7089	SAMEA104585108	Van Belleghem *et al.*[51]	3,829,666	72.45	1.89	WGS
flo.CS2359	CS002359	*Heliconius timareta ssp. florencia*	Colombia	1.7108	-75.7089	SAMEA104585109	Van Belleghem *et al.*[51]	3,693,632	69.88	1.90	WGS

SequenceID	EarthCapeID	Taxon Name	Country	Latitude	Longitude	Accession	Publication	Called sites	Geno-typed sites after filters (%)	Proport-ion of hetero-zygous genotypes (%)	Data Type (WGS…whole genome re-sequencing, CS…capture sequencing)
CS3009	CS003009	*Heliconius timareta ssp. linaresi*	Colombia	2.6844	-74.8881	ERR3652401		3,820,489	72.41	1.71	CS
CS2429	CS002429	*Heliconius timareta ssp. linaresi*	Colombia	2.6090	-74.7776	ERR3656288		3,797,759	71.93	1.78	CS
CS2436	CS002436	*Heliconius timareta ssp. linaresi*	Colombia	2.6090	-74.7776	ERR3652599		3,822,561	72.41	1.76	CS
CS3014	CS003014	*Heliconius timareta ssp. linaresi*	Colombia	2.6844	-74.8881	ERR3652392		3,781,757	71.51	1.95	CS
CS3012	CS003012	*Heliconius timareta ssp. linaresi*	Colombia	2.6844	-74.8881	ERR3656282		3,800,749	71.99	1.78	CS
CS3760	CS003760	*Heliconius timareta ssp. linaresi*	Colombia	2.6844	-74.8881	ERR3652608		3,793,257	72.04	1.51	CS
CS3050	CS003050	*Heliconius timareta ssp. linaresi*	Colombia	2.6844	-74.8881	ERR3652395		3,814,986	72.15	1.93	CS
CS3032	CS003032	*Heliconius timareta ssp. linaresi*	Colombia	2.6844	-74.8881	ERR3652593		3,821,017	72.40	1.74	CS
CS2422	CS002422	*Heliconius timareta ssp. linaresi*	Colombia	2.6090	-74.7776	ERR3652596		3,819,912	72.39	1.74	CS
CS3013	CS003013	*Heliconius timareta ssp. linaresi*	Colombia	2.6844	-74.8881	ERR3652404		3,827,502	72.40	1.91	CS
CS3046	CS003046	*Heliconius timareta ssp. linaresi*	Colombia	2.6844	-74.8881	ERR3652587		3,741,134	70.90	1.72	CS
CS3762	CS003762	*Heliconius timareta ssp. linaresi*	Colombia	2.6844	-74.8881	ERR3652604		3,803,044	72.08	1.71	CS
CS3367	CS003367	*Heliconius timareta ssp. linaresi*	Colombia	2.6844	-74.8881	ERR3652390		3,804,131	72.04	1.79	CS
CS3763	CS003763	*Heliconius timareta ssp. linaresi*	Colombia	2.6844	-74.8881	ERR3652602		3,822,941	72.35	1.86	CS
CS3372	CS003372	*Heliconius timareta ssp. linaresi*	Colombia	2.6844	-74.8881	ERR3656283		3,805,156	72.26	1.53	CS
CS3051	CS003051	*Heliconius timareta ssp. linaresi*	Colombia	2.6844	-74.8881	ERR3652590		3,820,696	72.31	1.86	CS
CS3761	CS003761	*Heliconius timareta ssp. linaresi*	Colombia	2.6844	-74.8881	ERR3652606		3,823,361	72.53	1.63	CS
CS3086	CS003086	*Heliconius timareta ssp. linaresi*	Colombia	2.6844	-74.8881	ERR3652398		3,821,604	72.35	1.82	CS
CS2435	CS002435	*Heliconius timareta ssp. linaresi*	Colombia	2.6090	-74.7776	ERR3653535 (SAMEA1094 485)	Nadeau *et al.*[69]; SureSelect	3,833,747	72.55	1.87	CS
PS16870	CAM016870	*Heliconius timareta ssp. nov. ECU*	Ecuador	-1.3712	-77.8574	ERR3652396		3,750,493	71.04	1.78	CS
PS16857	CAM016857	*Heliconius timareta ssp. nov. ECU*	Ecuador	-1.4021	-77.7974	ERR3652394		3,764,862	71.30	1.80	CS
CAM016605	CAM016605	*Heliconius timareta ssp. nov. ECU*	Ecuador	-1.1156	-77.7783	ERR3653402		3,685,825	69.87	1.71	CS
CAM016603	CAM016603	*Heliconius timareta ssp. nov. ECU*	Ecuador	-1.1156	-77.7783	ERR3653392		3,611,835	68.46	1.71	CS
CAM016600	CAM016600	*Heliconius timareta ssp. nov. ECU*	Ecuador	-1.1156	-77.7783	ERR3653311		3,668,367	69.47	1.80	CS
CAM016593	CAM016593	*Heliconius timareta ssp. nov. ECU*	Ecuador	-1.1156	-77.7783	ERR3653405		3,662,851	69.38	1.77	CS
CAM016502	CAM016502	*Heliconius timareta ssp. nov. ECU*	Ecuador	-1.2908	-77.8419	ERR3653394		3,675,153	69.63	1.76	CS
CAM016501	CAM016501	*Heliconius timareta ssp. nov. ECU*	Ecuador	-1.2908	-77.8419	ERR3653395		3,692,687	69.93	1.80	CS
CAM016443	CAM016443	*Heliconius timareta ssp. nov. ECU*	Ecuador	-1.1878	-77.8311	ERR3653403		3,679,600	69.67	1.82	CS
CAM016442	CAM016442	*Heliconius timareta ssp. nov. ECU*	Ecuador	-1.1878	-77.8311	ERR3653396		3,670,424	69.55	1.74	CS
CAM016304	CAM016304	*Heliconius timareta ssp. nov. ECU*	Ecuador	-1.3333	-77.9341	ERR3653397		3,724,010	70.50	1.83	CS
PS16269	CAM016269	*Heliconius timareta ssp. nov. ECU*	Ecuador	-1.2510	-77.6989	ERR3651369		3,764,038	71.37	1.68	CS
CAM016260	CAM016260	*Heliconius timareta ssp. nov. ECU*	Ecuador	-1.2510	-77.6989	ERR3653398		3,695,564	70.04	1.72	CS
CAM016257	CAM016257	*Heliconius timareta ssp. nov. ECU*	Ecuador	-1.2510	-77.6989	ERR3653399		3,694,177	70.01	1.72	CS
CAM016255	CAM016255	*Heliconius timareta ssp. nov. ECU*	Ecuador	-1.2510	-77.6989	ERR3653401		3,688,248	69.89	1.74	CS
PS16252	CAM016252	*Heliconius timareta ssp. nov. ECU*	Ecuador	-1.8176	-77.9601	ERR3652391		3,754,683	71.13	1.76	CS
CAM016218	CAM016218	*Heliconius timareta ssp. nov. ECU*	Ecuador	-1.1156	-77.7783	ERR3653406		3,654,949	69.19	1.83	CS
CAM016214	CAM016214	*Heliconius timareta ssp. nov. ECU*	Ecuador	-1.1156	-77.7783	ERR3653310		3,682,926	69.78	1.75	CS
PS16201	CAM016201	*Heliconius timareta ssp. nov. ECU*	Ecuador	-1.1156	-77.7783	ERR3651366		3,756,272	71.14	1.79	CS
PS16200	CAM016200	*Heliconius timareta ssp. nov. ECU*	Ecuador	-1.1156	-77.7783	ERR3652389		3,746,590	70.99	1.74	CS

SequenceID	EarthCapeID	Taxon Name	Country	Latitude	Longitude	Accession	Publication	Called sites	Geno-typed sites after filters (%)	Proport-ion of hetero-zygous genotypes (%)	Data Type (WGS...whole genome re-sequencing, CS...capture sequencing)
PS16173	CAM016173	*Heliconius timareta ssp. nov. ECU*	Ecuador	-1.2408	-77.9609	ERR3651362		3,755,655	71.09	1.85	CS
CAM016037	CAM016037	*Heliconius timareta ssp. nov. ECU*	Ecuador	-1.2908	-77.8419	ERR3653407		3,696,783	69.94	1.89	CS
PS16011	CAM016011	*Heliconius timareta ssp. nov. ECU*	Ecuador	-1.3333	-77.9341	ERR3650738		3,765,599	71.30	1.82	CS
PS16425	CAM016425	*Heliconius timareta ssp. nov. ECU*	Ecuador	-1.1878	-77.8311	ERR3653530 ; (SAMEA2240 078)	Nadeau *et al.*[34]; RAD	3,754,500	71.13	1.75	CS
PS17380	CAM017380	*Heliconius timareta ssp. nov. ECU*	Ecuador	-1.0983	-77.5839	ERR3653531 ; (SAMEA2240 096)	Nadeau *et al.*[34]; RAD	3,759,205	71.20	1.78	CS
CS31766	CS031766	*Heliconius timareta ssp. nov. 'vicina'*	Colombia	-4.1334	-69.9415	ERR3652639		3,881,511	73.68	1.57	CS
CS31733	CS031733	*Heliconius timareta ssp. nov. 'vicina'*	Colombia	-3.7701	-70.3398	ERR3652634		3,917,016	74.32	1.61	CS
CS31809	CS031809	*Heliconius timareta ssp. nov. 'vicina'*	Colombia	-3.7701	-70.3398	ERR3652575		3,876,918	73.61	1.54	CS
CS31711	CS031711	*Heliconius timareta ssp. nov. 'vicina'*	Colombia	-3.7701	-70.3398	ERR3652631		3,911,288	74.17	1.66	CS
CS32537	CS032537	*Heliconius timareta ssp. nov. 'vicina'*	Colombia	-3.8833	-70.1881	ERR3652626		3,898,267	73.99	1.57	CS
CS32535	CS032535	*Heliconius timareta ssp. nov. 'vicina'*	Colombia	-4.0406	-70.0997	ERR3652628		3,864,120	73.25	1.70	CS
MJ12-3388	MJ12-3388	*Heliconius timareta ssp. thelxinoe*	Peru	-6.4515	-76.2977	ERR3650728		3,681,338	69.66	1.88	CS
MJ12-3310	MJ12-3310	*Heliconius timareta ssp. thelxinoe*	Peru	-6.4519	-76.2985	ERR3650479		3,462,285	65.77	1.50	CS
MJ12-3232	MJ12-3232	*Heliconius timareta ssp. thelxinoe*	Peru	-6.4515	-76.2977	ERR3650588		3,664,260	69.42	1.76	CS
MJ12-3412	MJ12-3412	*Heliconius timareta ssp. thelxinoe*	Peru	-6.4537	-76.2981	ERR3651417		3,746,879	70.93	1.84	CS
MJ12-3160	MJ12-3160	*Heliconius timareta ssp. thelxinoe*	Peru	-6.4519	-76.2985	ERR3650494		3,692,631	69.94	1.78	CS
MJ12-3212	MJ12-3212	*Heliconius timareta ssp. thelxinoe*	Peru	-6.4547	-76.2994	ERR3650497		3,657,709	69.37	1.65	CS
MJ12-3402	MJ12-3402	*Heliconius timareta ssp. thelxinoe*	Peru	-6.4528	-76.2862	ERR3651368		3,684,144	69.69	1.91	CS
MJ12-3222	MJ12-3222	*Heliconius timareta ssp. thelxinoe*	Peru	-6.4519	-76.2985	ERR3650512		3,690,826	69.90	1.79	CS
MJ12-3138	MJ12-3138	*Heliconius timareta ssp. thelxinoe*	Peru	-6.4547	-76.2994	ERR3650493		3,682,422	69.97	1.47	CS
MJ12-3398	MJ12-3398	*Heliconius timareta ssp. thelxinoe*	Peru	-6.4515	-76.2977	ERR3651361		3,678,752	69.61	1.87	CS
MJ12-3220	MJ12-3220	*Heliconius timareta ssp. thelxinoe*	Peru	-6.4515	-76.2977	ERR3650474		3,492,663	66.21	1.70	CS
MJ12-3135	MJ12-3135	*Heliconius timareta ssp. thelxinoe*	Peru	-6.4547	-76.2994	ERR3650490		3,708,004	70.29	1.70	CS
MJ12-3405	MJ12-3405	*Heliconius timareta ssp. thelxinoe*	Peru	-6.4540	-76.3002	ERR3651371		3,653,870	69.17	1.84	CS
MJ12-3409	MJ12-3409	*Heliconius timareta ssp. thelxinoe*	Peru	-6.4537	-76.2981	ERR3651416		3,671,396	69.59	1.70	CS
MJ12-3231	MJ12-3231	*Heliconius timareta ssp. thelxinoe*	Peru	-6.4515	-76.2977	ERR3650478		3,449,406	65.42	1.66	CS
MJ12-3219	MJ12-3219	*Heliconius timareta ssp. thelxinoe*	Peru	-6.4519	-76.2985	ERR3650499		3,673,933	69.66	1.68	CS
MJ12-3370	MJ12-3370	*Heliconius timareta ssp. thelxinoe*	Peru	-6.4528	-76.2862	ERR3650692		3,662,416	69.24	1.96	CS
MJ12-3408	MJ12-3408	*Heliconius timareta ssp. thelxinoe*	Peru	-6.4519	-76.2985	ERR3651412		3,737,857	70.86	1.70	CS
MJ12-3136	MJ12-3136	*Heliconius timareta ssp. thelxinoe*	Peru	-6.4547	-76.2994	ERR3650491		3,563,214	67.57	1.67	CS
MJ12-3223	MJ12-3223	*Heliconius timareta ssp. thelxinoe*	Peru	-6.4515	-76.2977	ERR3650477		3,450,844	65.47	1.62	CS
MJ12-3401	MJ12-3401	*Heliconius timareta ssp. thelxinoe*	Peru	-6.4528	-76.2862	ERR3651367		3,695,405	70.18	1.52	CS
MJ12-3313	MJ12-3313	*Heliconius timareta ssp. thelxinoe*	Peru	-6.4547	-76.2994	ERR3650673		3,433,603	64.96	1.89	CS
MJ12-3400	MJ12-3400	*Heliconius timareta ssp. thelxinoe*	Peru	-6.4515	-76.2977	ERR3651364		3,728,573	70.66	1.72	CS
MJ12-3318	MJ12-3318	*Heliconius timareta ssp. thelxinoe*	Peru	-6.4547	-76.2994	ERR3650677		3,598,328	68.15	1.79	CS
MJ12-3378	MJ12-3378	*Heliconius timareta ssp. thelxinoe*	Peru	-6.4519	-76.2985	ERR3650708		3,691,987	69.98	1.72	CS
MJ12-3404	MJ12-3404	*Heliconius timareta ssp. thelxinoe*	Peru	-6.4540	-76.3002	ERR3651370		3,687,744	69.92	1.68	CS
MJ12-3406	MJ12-3406	*Heliconius timareta ssp. thelxinoe*	Peru	-6.4537	-76.2981	ERR3651375		3,704,412	70.16	1.79	CS

**SequenceID**	**EarthCapeID**	**Taxon Name**	**Country**	**Latitude**	**Longitude**	**Accession**	**Publication**	**Called sites**	**Geno-typed sites after filters (%)**	**Proport-ion of hetero-zygous genotypes (%)**	**Data Type** **(WGS...whole genome re-sequencing, CS...capture sequencing)**
MJ12-3174	MJ12-3174	*Heliconius timareta ssp. thelxinoe*	Peru	-6.4547	-76.2994	ERR3650496		3,658,409	69.17	1.95	CS
MJ12-3390	MJ12-3390	*Heliconius timareta ssp. thelxinoe*	Peru	-6.4528	-76.2862	ERR3650748		3,699,234	70.01	1.86	CS
thxn.MJ12-3221	MJ12-3221	*Heliconius timareta ssp. thelxinoe*	Peru	-6.4519	-76.2985	SAMEA104585110	Van Belleghem *et al.*[51]	3,819,091	72.32	1.81	WGS
thxn.MJ12-3233	MJ12-3233	*Heliconius timareta ssp. thelxinoe*	Peru	-5.6546	-77.6938	SAMEA104585111	Van Belleghem *et al.*[51]	3,792,490	71.77	1.86	WGS
thxn.MJ12-3308	MJ12-3308	*Heliconius timareta ssp. thelxinoe*	Peru	-6.4519	-76.2985	SAMEA104585112	Van Belleghem *et al.*[51]	3,760,503	71.06	2.02	WGS
txn.MJ11-3339	MJ12-3339	*Heliconius timareta ssp. thelxinoe*	Peru	-5.6546	-77.6938	SAMEA104585113	Van Belleghem *et al.*[51]	3,523,213	66.66	1.89	WGS
txn.MJ11-3340	MJ12-3340	*Heliconius timareta ssp. thelxinoe*	Peru	-5.6546	-77.6938	SAMEA104585114	Van Belleghem *et al.*[51]	3,437,195	65.07	1.83	WGS
txn.MJ12-3460	MJ12-3460	*Heliconius timareta ssp. thelxinoe*	Peru	-5.6546	-77.6938	SAMEA104585115	Van Belleghem *et al.*[51]	3,651,061	69.03	1.96	WGS
thxn.JM57	JM-09-57	*Heliconius timareta ssp. thelxinoe*	Peru	-6.4528	-76.2987	SAMEA1919254	Martin *et al.*[71]	3,964,602	74.96	1.95	WGS
thxn.JM86	JM-09-86	*Heliconius timareta ssp. thelxinoe*	Peru	-6.4528	-76.2987	SAMEA1919263	Martin *et al.*[71]	3,969,848	74.91	2.15	WGS
thxn.JM313	JM-09-313	*Heliconius timareta ssp. thelxinoe*	Peru	-6.4584	-76.2877	SAMEA1919266	Martin *et al.*[71]	3,953,763	74.65	2.09	WGS
thxn.JM84	JM-09-84	*Heliconius timareta ssp. thelxinoe*	Peru	-6.4528	-76.2987	SAMEA1919273	Martin *et al.*[71]	3,819,757	72.27	1.89	WGS
CAM009187	CAM009187	*Heliconius timareta ssp. timareta f. contigua*	Ecuador	-1.3980	-78.1781	ERR3653301		3,755,103	71.11	1.80	CS
CAM009186	CAM009186	*Heliconius timareta ssp. timareta f. contigua*	Ecuador	-1.3980	-78.1781	ERR3653305		3,714,942	70.37	1.77	CS
CAM009185	CAM009185	*Heliconius timareta ssp. timareta f. contigua*	Ecuador	-1.3980	-78.1781	ERR3653300		3,724,200	70.56	1.76	CS
CAM009183	CAM009183	*Heliconius timareta ssp. timareta f. contigua*	Ecuador	-1.3980	-78.1781	ERR3653298		3,732,851	70.71	1.77	CS
CAM009177	CAM009177	*Heliconius timareta ssp. timareta f. contigua*	Ecuador	-1.3980	-78.1781	ERR3653306		3,768,012	71.35	1.80	CS
CAM009175	CAM009175	*Heliconius timareta ssp. timareta f. contigua*	Ecuador	-1.3980	-78.1781	ERR3653307		3,742,771	70.91	1.75	CS
CAM009180	CAM009180	*Heliconius timareta ssp. timareta f. contigua*	Ecuador	-1.3980	-78.1781	ERR3653543; (SAMEA1322935)	The Heliconius Consortium[44]; RAD; SureSelect	3,752,197	71.02	1.85	CS
CAM016719	CAM016719	*Heliconius timareta ssp. timareta f. contigua*	Ecuador	-1.3980	-78.1781	ERR3653309		3,759,873	71.19	1.82	CS
CAM016716	CAM016716	*Heliconius timareta ssp. timareta f. contigua*	Ecuador	-1.3980	-78.1781	ERR3653302		3,730,788	70.61	1.85	CS
CAM016715	CAM016715	*Heliconius timareta ssp. timareta f. contigua*	Ecuador	-1.3980	-78.1781	ERR3653303		3,599,854	68.22	1.73	CS
CAM011413	CAM011413	*Heliconius timareta ssp. timareta f. contigua*	Ecuador	-1.3980	-78.1781	ERR3653308		3,726,722	70.57	1.80	CS
CAM011439	CAM011439	*Heliconius timareta ssp. timareta f. timareta*	Ecuador	-1.3980	-78.1781	ERR3653297		3,789,280	71.74	1.83	CS
CAM009440	CAM009440	*Heliconius timareta ssp. timareta f. timareta*	Ecuador	-1.3980	-78.1781	ERR3653292		3,822,542	72.44	1.73	CS
CAM009426	CAM009426	*Heliconius timareta ssp. timareta f. timareta*	Ecuador	-1.3980	-78.1781	ERR3653295		3,816,404	72.23	1.86	CS
CAM009421	CAM009421	*Heliconius timareta ssp. timareta f. timareta*	Ecuador	-1.3980	-78.1781	ERR3653293		3,801,719	72.11	1.64	CS
CAM009419	CAM009419	*Heliconius timareta ssp. timareta f. timareta*	Ecuador	-1.3980	-78.1781	ERR3653125		3,788,443	71.73	1.81	CS
CAM009227	CAM009227	*Heliconius timareta ssp. timareta f. timareta*	Ecuador	-1.4530	-78.1070	ERR3653299		3,726,694	70.61	1.75	CS
CAM009169	CAM009169	*Heliconius timareta ssp. timareta f. timareta*	Ecuador	-1.3980	-78.1781	ERR3653296		3,756,296	71.12	1.81	CS

SequenceID	EarthCapeID	Taxon Name	Country	Latitude	Longitude	Accession	Publication	Called sites	Geno-typed sites after filters (%)	Proport-ion of hetero-zygous genotypes (%)	Data Type (WGS...whole genome re-sequencing, CS...capture sequencing)
CAM008533	CAM008533	*Heliconius timareta ssp. timareta f. timareta*	Ecuador	-1.3980	-78.1781	ERR3653537 (SAMEA1094484)	Nadeau *et al.*[69]; SureSelect	3,841,553	72.69	1.88	CS
CAM009178	CAM009178	*Heliconius timareta ssp. timareta f. timareta*	Ecuador	-1.3980	-78.1781	ERR3653539 (SAMEA3670574)	Wallbank *et al.*[36]	3,678,631	69.72	1.73	CS
