# Supplementary material for: Selective sweeps on novel and introgressed variation shape mimicry loci in a butterfly adaptive radiation
Source: PLoS Biol. 2020 Feb 6;18(2):e3000597. doi: 10.1371/journal.pbio.3000597 (PMC7029882; doi:10.1371/journal.pbio.3000597)
Supplement: S2 Table — (PDF) [file pbio.3000597.s024.pdf]

S2 Table. Per-population sample sizes for the *H. melpomene*-clade and the *H. erato*–clade used in the respective analyses.

Taxon Name	Location	Sequencing	Tree	pi/TajD/ihh12	SF	VF	PSMC'	Comment
Heliconius besckei	Brazil	11	11	11	11	11	4	
Heliconius cydno ssp. chioneus	Panamá	11	11	11	11	11	10	
Heliconius cydno ssp. cordula	Venezuela	3	3	NA	NA	NA	3	
Heliconius cydno ssp. cydnides	Colombia	10	10	10	10	10	NA	
Heliconius cydno ssp. weymeri f. gustavi	Colombia	10	10	10	10	10	NA	
Heliconius cydno ssp. weymeri f. weymeri	Colombia	10	10	10	10	10	NA	
Heliconius cydno ssp. zelinde	Colombia	10	10	10	10	10	10	
Heliconius elevatus	Ecuador	14	14	14	14	14	3	
Heliconius hecale	Panamá/Peru	2	2	NA	NA	NA	NA	
Heliconius heurippa	Colombia	23	23	23	23	23	3	
Heliconius ismenius	Panamá	2	2	NA	NA	NA	NA	
Heliconius melpomene ssp. aglaope	Peru	4	4	4	4	4	4	
Heliconius melpomene ssp. amaryllis	Peru	38	38	38	38	38	10	
Heliconius melpomene ssp. burchelli	Brazil	3	3	NA	NA	NA	NA	
Heliconius melpomene ssp. cythera	Ecuador	10	10	10	10	10	NA	
Heliconius melpomene ssp. ecuadorensis	Ecuador	10	10	10	10	10	NA	
Heliconius melpomene ssp. meriana	Suriname/French Guiana	10	10	10	10	10	2	
Heliconius melpomene ssp. malleti	Ecuador	29	29	29	29	29	NA	
Heliconius melpomene ssp. malleti	Colombia	23	23	23	23	23	10	
Heliconius melpomene ssp. melpomene	Panamá	8	8	8	8	8	3	
Heliconius melpomene ssp. melpomene	French Guiana	11	11	11	11	11	10	
Heliconius melpomene ssp. melpomene	Colombia	18	18	18	18	18	5	
Heliconius melpomene ssp. nanna	Brazil	12	12	12	12	12	4	
Heliconius melpomene ssp. plesseni	Ecuador	12	12	12	12	12	3	
Heliconius melpomene ssp. rosina	Panamá	10	10	10	10	10	10	
Heliconius melpomene ssp. vicina	Colombia	3	3	NA	NA	NA	NA	
Heliconius melpomene ssp. vulcanus	Panama/Colombia	11	11	11	11	11	10	
Heliconius melpomene ssp. xenoclea	Peru	10	10	10	10	10	NA	
Heliconius numata ssp. numata	French Guiana	1	1	1	outgroup	outgroup	NA	
Heliconius numata ssp. silvana	Peru/French Guiana	4	4	4	outgroup	outgroup	NA	
Heliconius pachinus	Panamá	10	10	10	10	10	2	
Heliconius pardalinus ssp. sergestus	Peru	1	1	1	NA	NA	NA	
Heliconius pardalinus ssp. ssp.nov.P	Peru	1	1	1	NA	NA	NA	
Heliconius timareta ssp. timareta f. timareta	Ecuador	9	9	9	9	9	NA	
Heliconius timareta ssp. timareta f. contigua	Ecuador	11	11	11	11	11	NA	
Heliconius timareta ssp. nov.	Colombia	6	6	6	6	6	NA	
Heliconius timareta ssp. florencia	Colombia	20	20	20	20	20	10	
Heliconius timareta ssp. linaresi	Colombia	19	19	19	19	19	NA	
Heliconius timareta ssp. nov.	Ecuador	25	25	25	25	25	NA	
Heliconius timareta ssp. thelxinoe	Peru	39	39	39	39	39	10	

Taxon Name	Location	Sequencing	Tree	pi/TajD/ihh12	SF	VF	PSMC'	Comment
Heliconius erato amalfreda	Suriname	5	NA	5	5	NA	5	PSMC' published in [51]
Heliconius erato cyrbia	Ecuador	4	NA	4	4	NA	4	PSMC' published in [51]
Heliconius erato demophoon	Panama	10	NA	10	10	NA	10	PSMC' published in [51]
Heliconius erato emma	Peru	11	NA	11	11	NA	11	PSMC' published in [51]
Heliconius erato	French Guiana	6	NA	6	6	NA	6	PSMC' published in [51]
Heliconius erato etylus	Ecuador	5	NA	5	5	NA	5	PSMC' published in [51]
Heliconius erato favorinus	Peru	12	NA	12	12	NA	12	PSMC' published in [51]
Heliconius erato hydara	French Guiana	5	NA	5	5	NA	5	PSMC' published in [51]
Heliconius erato hydara	Panama	6	NA	6	6	NA	6	PSMC' published in [51]
Heliconius erato lativitta	Ecuador	5	NA	5	5	NA	5	PSMC' published in [51]
Heliconius erato notabilis	Ecuador	10	NA	10	10	NA	10	PSMC' published in [51]
Heliconius erato venus	Colombia	5	NA	5	5	NA	5	PSMC' published in [51]
Heliconius himera	Ecuador/Peru	9	NA	9	9	NA	9	PSMC' published in [51]
Heliconius erato chestertonii	Colombia	7	NA	7	7	NA	7	PSMC' published in [51]
Heliconius hermathena	Brazil	3	NA	3	outgroup	NA	NA	PSMC' published in [51]
