# Supplementary material for: Selective sweeps on novel and introgressed variation shape mimicry loci in a butterfly adaptive radiation
Source: PLoS Biol. 2020 Feb 6;18(2):e3000597. doi: 10.1371/journal.pbio.3000597 (PMC7029882; doi:10.1371/journal.pbio.3000597)
Supplement: S3 Table — PSMC’, Pairwise Sequentially Markovian Coalescent. (PDF) [file pbio.3000597.s025.pdf]

S3 Table. Sample information for whole-genome sequence data used for PSMC' analysis.

SequenceID	EarthCapeID	Taxon name	Country	Sex	Longitude	Latitude	Accession
agl.JM108	JM-09-108	*Heliconius melpomene aglaope*	Peru	m	-5.9103	-76.2258	SAMEA1919251
agl.JM112	JM-09-112	*Heliconius melpomene aglaope*	Peru	m	-5.9103	-76.2258	SAMEA1919264
agl.JM569	JM-11-569	*Heliconius melpomene aglaope*	Peru	m	-5.9458	-76.2453	SAMEA1919274
agl.JM572	JM-11-572	*Heliconius melpomene aglaope*	Peru	m	-5.9458	-76.2466	SAMEA1919259
ama.JM160	JM-11-160	*Heliconius melpomene amaryllis*	Peru	f	-5.6756	-77.6747	SAMEA1919261
ama.JM216	JM-09-216	*Heliconius melpomene amaryllis*	Peru	m	-6.4685	-76.3533	SAMEA1919261
ama.JM293	JM-11-293	*Heliconius melpomene amaryllis*	Peru	f	-6.4703	-76.3473	SAMEA1919277
ama.JM48	JM-11-48	*Heliconius melpomene amaryllis*	Peru	m	-6.096	-76.9774	SAMEA1919269
ama.MJ11-3188	MJ11-3188	*Heliconius melpomene amaryllis*	Peru	m	-5.67284	-77.71947	SAMEA104585061
ama.MJ11-3189	MJ11-3189	*Heliconius melpomene amaryllis*	Peru	m	-5.67284	-77.71947	SAMEA104585062
ama.MJ11-3202	MJ11-3202	*Heliconius melpomene amaryllis*	Peru	m	-5.67453	-77.67114	SAMEA104585063
ama.MJ12-3217	MJ12-3217	*Heliconius melpomene amaryllis*	Peru	m	-6.45474	-76.29944	SAMEA104585064
ama.MJ12-3258	MJ12-3258	*Heliconius melpomene amaryllis*	Peru	m	-6.45296	-76.28762	SAMEA104585065
ama.MJ12-3301	MJ12-3301	*Heliconius melpomene amaryllis*	Peru	m	-6.45283	-76.286215	SAMEA104585066
chi.CAM25091	CAM025091	*Heliconius cydno chioneus*	Panama	f	9.12	-79.702	SAMEA104585050
chi.CAM25137	CAM025137	*Heliconius cydno chioneus*	Panama	f	9.12	-79.702	SAMEA104585051
chi.CAM580	CAM000580	*Heliconius cydno chioneus*	Panama	m	9.12	-79.702	SAMEA104585044
chi.CAM582	CAM000582	*Heliconius cydno chioneus*	Panama	m	9.12	-79.702	SAMEA104585045
chi.CAM585	CAM000585	*Heliconius cydno chioneus*	Panama	m	9.12	-79.702	SAMEA104585047
chi.CAM586	CAM000586	*Heliconius cydno chioneus*	Panama	m	9.12	-79.702	SAMEA104585048
chi.CJ553	CAM000553	*Heliconius cydno chioneus*	Panama	m	9.1714	-79.7573	SAMEA1919256
chi.CJ560	CAM0060	*Heliconius cydno chioneus*	Panama	m	9.1714	-79.7573	SAMEA1919265
chi.CJ564	CAM000564	*Heliconius cydno chioneus*	Panama	m	9.1714	-79.7573	SAMEA1919278
chi.CJ565	CAM000565	*Heliconius cydno chioneus*	Panama	m	9.1714	-79.7573	SAMEA1919262
cor.CS3	CS002167	*Heliconius cydno cordula*	Venezuela	m	7.798888	-72.198888	SAMEA104585052
cor.CS4	CS002258	*Heliconius cydno cordula*	Venezuela	m	7.798888	-72.198888	SAMEA104585053
cor.STRI7	STRI_007	*Heliconius cydno cordula*	Venezuela	m	7.798888	-72.198888	SAMEA3670511
flo.CS12	CS002395	*Heliconius timareta florencia*	Colombia	m	1.70965	-75.697583	SAMEA104585100
flo.CS13	CS002402	*Heliconius timareta florencia*	Colombia	m	1.70965	-75.697583	SAMEA104585101
flo.CS14	CS002403	*Heliconius timareta florencia*	Colombia	m	1.70965	-75.697583	SAMEA104585102
flo.CS15	CS002406	*Heliconius timareta florencia*	Colombia	m	1.70965	-75.697583	SAMEA104585103
flo.CS2337	CS002337	*Heliconius timareta florencia*	Colombia	m	1.710833	-75.708889	SAMEA104585104
flo.CS2338	CS002338	*Heliconius timareta florencia*	Colombia	m	1.710833	-75.708889	SAMEA104585105
flo.CS2341	CS002341	*Heliconius timareta florencia*	Colombia	m	1.813611	-75.668611	SAMEA104585106
flo.CS2350	CS002350	*Heliconius timareta florencia*	Colombia	m	1.710833	-75.708889	SAMEA104585107
flo.CS2358	CS002358	*Heliconius timareta florencia*	Colombia	m	1.710833	-75.708889	SAMEA104585108
flo.CS2359	CS002359	*Heliconius timareta florencia*	Colombia	m	1.710833	-75.708889	SAMEA104585109
heu.CS20	CS00CH21	*Heliconius heurippa*	Colombia	f	4.175	-73.678056	SAMEA104585060
heu.CS9	CS002020	*Heliconius heurippa*	Colombia	m	4.175	-73.678056	SAMEA1322902
heu.STRI2	STRI_002	*Heliconius heurippa*	Colombia	f	4.175	-73.678056	SAMEA3670535
mal.CS1002	CS001002	*Heliconius melpomene malleti*	Colombia	m	1.803333	-75.655277	SAMEA104585067
mal.CS1011	CS001011	*Heliconius melpomene malleti*	Colombia	m	1.803333	-75.655277	SAMEA104585068
mal.CS1815	CS001815	*Heliconius melpomene malleti*	Colombia	m	1.803333	-75.655277	SAMEA104585069

SequenceID	EarthCapeID	Taxon name	Country	Sex	Longitude	Latitude	Accession
mal.CS21	CS002311	*Heliconius melpomene malleti*	Colombia	m	*1.813611*	*-75.668611*	SAMEA3723397
mal.CS22	CS001286	*Heliconius melpomene malleti*	Colombia	m	*1.609722*	*-75.666944*	SAMEA3723398
mal.CS24	CS001321	*Heliconius melpomene malleti*	Colombia	m	*1.750556*	*-75.631944*	SAMEA3723399
mal.CS586	CS000586	*Heliconius melpomene malleti*	Colombia	m	1.803333	-75.655277	SAMEA104585071
mal.CS594	CS000594	*Heliconius melpomene malleti*	Colombia	f	1.803333	-75.655277	SAMEA104585072
mal.CS604	CS000604	*Heliconius melpomene malleti*	Colombia	m	1.803333	-75.655277	SAMEA104585073
mal.CS615	CS000615	*Heliconius melpomene malleti*	Colombia	m	1.803333	-75.655277	SAMEA104585074
melC.CS25	CS000CM4	*Heliconius melpomene melpomene*	Colombia	m	4.924724	-68.925111	SAMEA3723400
melC.CS26	CS000CM5	*Heliconius melpomene melpomene*	Colombia	m	4.213	-73.803	SAMEA3723401
melC.CS27	CS00CM10	*Heliconius melpomene melpomene*	Colombia	m	5.617	-72.3	SAMEA3723402
melC.CS3	CM3	*Heliconius melpomene melpomene*	Colombia	m	4.213	-73.803	SAMEA3723393
melC.CS6	CS000CM6	*Heliconius melpomene melpomene*	Colombia	m	5.617	-72.3	SAMEA3723394
melG.CAM1349	CAM001349	*Heliconius melpomene melpomene*	French Guiana	f	2.522209	-51.193404	SAMEA104585075
melG.CAM1422	CAM001422	*Heliconius melpomene melpomene*	French Guiana	m	2.522209	-51.193404	SAMEA104585076
melG.CAM2035	CAM002035	*Heliconius melpomene melpomene*	French Guiana	m	2.522209	-51.193404	SAMEA104585077
melG.CAM8171	CAM008171	*Heliconius melpomene melpomene*	French Guiana	f	2.522209	-51.193404	SAMEA104585078
melG.CAM8216	CAM008216	*Heliconius melpomene melpomene*	French Guiana	m	2.522209	-51.193404	SAMEA104585080
melG.CAM8218	CAM008218	*Heliconius melpomene melpomene*	French Guiana	m	2.522209	-51.193404	SAMEA104585081
melG.CJ13435	CAM013435	*Heliconius melpomene melpomene*	French Guiana	m	2.522209	-51.193404	SAMEA1919276
melG.CJ9315	CAM009315	*Heliconius melpomene melpomene*	French Guiana	m	2.522209	-51.193404	SAMEA1919270
melG.CJ9316	CAM009316	*Heliconius melpomene melpomene*	French Guiana	m	2.522209	-51.193404	SAMEA1919252
melG.CJ9317	CAM009317	*Heliconius melpomene melpomene*	French Guiana	m	2.522209	-51.193404	SAMEA1919267
melP.CJ18038	CAM018038	*Heliconius melpomene melpomene*	Panama	f	8.6136	-78.1398	SAMEA1919255
melP.CJ18097	CAM018097	*Heliconius melpomene melpomene*	Panama	m	8.2797	-77.8098	SAMEA1919258
melP.HGC1	gen_ref	*Heliconius melpomene melpomene*	Panama	f	*NA*	*NA*	SAMN00794386
melPle.CJ16042	CAM016042	*Heliconius melpomene plesseni*	Ecuador	m	-1.371267	-77.874517	SAMEA3670548
nan.MK14	MK000014	*Heliconius melpomene nanna*	Brazil	m	-12.374009	-48.189253	SAMN04407968
nan.MK62	MK000062	*Heliconius melpomene nanna*	Brazil	m	-12.374009	-48.189253	SAMN04407969
nan.MK63	MK000063	*Heliconius melpomene nanna*	Brazil	m	-12.374009	-48.189253	SAMN04407970
nan.MK64	MK000064	*Heliconius melpomene nanna*	Brazil	m	-12.374009	-48.189253	SAMN04407961
ple.CJ16293	CAM016293	*Heliconius melpomene plesseni*	Ecuador	m	-1.459967	-78.0728	SAMEA3670557
ple.CJ9156	CAM009156	*Heliconius melpomene plesseni*	Ecuador	m	-1.39801	-78.17813	SAMEA3670556
ros.CAM1841	CAM001841	*Heliconius melpomene rosina*	Panama	m	9.076	-79.659	SAMEA104585083
ros.CAM1880	CAM001880	*Heliconius melpomene rosina*	Panama	m	9.076	-79.659	SAMEA104585084
ros.CAM2045	CAM002045	*Heliconius melpomene rosina*	Panama	m	9.1103	-79.6907	SAMEA104585085
ros.CAM2059	CAM002059	*Heliconius melpomene rosina*	Panama	m	9.1103	-79.6907	SAMEA104585086
ros.CAM2519	CAM002519	*Heliconius melpomene rosina*	Panama	m	9.0109	-79.5477	SAMEA104585087
ros.CAM2552	CAM002552	*Heliconius melpomene rosina*	Panama	m	9.0109	-79.5477	SAMEA104585088
ros.CJ2071	CAM002071	*Heliconius melpomene rosina*	Panama	m	9.1206	-79.6969	SAMEA1919257
ros.CJ531	CAM000531	*Heliconius melpomene rosina*	Panama	m	9.1206	-79.6969	SAMEA1919271
ros.CJ533	CAM000533	*Heliconius melpomene rosina*	Panama	m	9.1206	-79.6969	SAMEA1919260
ros.CJ546	CAM000546	*Heliconius melpomene rosina*	Panama	m	9.1206	-79.6969	SAMEA1919279
thxn.JM313	JM-09-313	*Heliconius timareta thelxinoe*	Peru	m	-6.4584	-76.2877	SAMEA1919266
thxn.JM57	JM-09-57	*Heliconius timareta thelxinoe*	Peru	m	-6.4528	-76.2987	SAMEA1919254

SequenceID	EarthCapeID	Taxon name	Country	Sex	Longitude	Latitude	Accession
thxn.JM84	JM-09-84	*Heliconius timareta thelxinoe*	Peru	m	-6.4528	-76.2987	SAMEA1919273
thxn.JM86	JM-09-86	*Heliconius timareta thelxinoe*	Peru	m	-6.4528	-76.2987	SAMEA1919263
thxn.MJ12-3221	MJ12-3221	*Heliconius timareta thelxinoe*	Peru	m	-5.65464	-77.69375	SAMEA104585110
thxn.MJ12-3233	MJ12-3233	*Heliconius timareta thelxinoe*	Peru	m	-6.4519	-76.29846	SAMEA104585111
thxn.MJ12-3308	MJ12-3308	*Heliconius timareta thelxinoe*	Peru	m	-5.65464	-77.69375	SAMEA104585112
txn.MJ11-3339	MJ11-3339	*Heliconius timareta thelxinoe*	Peru	m	-5.65464	-77.69375	SAMEA104585113
txn.MJ11-3340	MJ11-3340	*Heliconius timareta thelxinoe*	Peru	m	-5.65464	-77.69375	SAMEA104585114
txn.MJ11-3460	MJ11-3460	*Heliconius timareta thelxinoe*	Peru	m	-5.65464	-77.69375	SAMEA104585115
vul.CS10	CS000710	*Heliconius melpomene vulcanus*	Colombia	m	*3.9*	*-76.6325*	SAMEA3723391
vul.CS3603	CS003603	*Heliconius melpomene vulcanus*	Colombia	m	3.5175	-76.757222	SAMEA104585091
vul.CS3605	CS003605	*Heliconius melpomene vulcanus*	Colombia	m	3.5175	-76.757222	SAMEA104585092
vul.CS3606	CS003606	*Heliconius melpomene vulcanus*	Colombia	m	3.5175	-76.757222	SAMEA104585093
vul.CS3612	CS003612	*Heliconius melpomene vulcanus*	Colombia	m	3.5175	-76.757222	SAMEA104585094
vul.CS3614	CS003614	*Heliconius melpomene vulcanus*	Colombia	m	3.5175	-76.757222	SAMEA104585095
vul.CS3615	CS003615	*Heliconius melpomene vulcanus*	Colombia	m	3.5175	-76.757222	SAMEA104585096
vul.CS3617	CS003617	*Heliconius melpomene vulcanus*	Colombia	m	3.5175	-76.757222	SAMEA104585097
vul.CS3618	CS003618	*Heliconius melpomene vulcanus*	Colombia	m	3.5175	-76.757222	SAMEA104585098
vul.CS3621	CS003621	*Heliconius melpomene vulcanus*	Colombia	m	3.5175	-76.757222	SAMEA104585099
zel.CS1	CS002242	*Heliconius cydno zelinde*	Colombia	m	3.939444	-77.368889	SAMEA104106540
zel.CS1028	CS001028	*Heliconius cydno zelinde*	Colombia	m	3.958333	-77.373333	SAMEA104585054
zel.CS1029	CS001029	*Heliconius cydno zelinde*	Colombia	m	3.939444	-77.368889	SAMEA104585055
zel.CS1030	CS001030	*Heliconius cydno zelinde*	Colombia	m	3.939444	-77.368889	SAMEA104585056
zel.CS1033	CS001033	*Heliconius cydno zelinde*	Colombia	m	3.958333	-77.373333	SAMEA104585057
zel.CS1035	CS001035	*Heliconius cydno zelinde*	Colombia	m	3.958333	-77.373333	SAMEA104585058
zel.CS2	CS002261	*Heliconius cydno zelinde*	Colombia	m	3.939444	-77.368889	SAMEA104106542
zel.CS2262	CS002262	*Heliconius cydno zelinde*	Colombia	f	3.958333	-77.373333	SAMEA3670517
zel.CS273	CS000273	*Heliconius cydno zelinde*	Colombia	m	3.958333	-77.373333	SAMEA104585059
zel.CS30	CS002260	*Heliconius cydno zelinde*	Colombia	f	3.939444	-77.368889	SAMEA104106543
besSRR3102011		*Heliconius melpomene besckei*	Brasil				SRR3102011
besSRR3102026		*Heliconius melpomene besckei*	Brasil				SRR3102026
besSRR3102061		*Heliconius melpomene besckei*	Brasil				SRR3102061
besSRR3102131		*Heliconius melpomene besckei*	Brasil				SRR3102131
eleERR1143573		*Heliconius elevatus*					ERR1143573
eleERR1143574		*Heliconius elevatus*					ERR1143574
eleERR1143575		*Heliconius elevatus*					ERR1143575
merERR1143603		*Heliconnius melpomene meriana*	French Guiana				ERR1143603
merERR1143604		*Heliconnius melpomene meriana*	French Guiana				ERR1143604
pacERR1143613		*Heliconius pachinus*	Panama				ERR1143613
pacERR1143614		*Heliconius pachinus*	Panama				ERR1143614
