# Supplementary material for: Selective sweeps on novel and introgressed variation shape mimicry loci in a butterfly adaptive radiation
Source: PLoS Biol. 2020 Feb 6;18(2):e3000597. doi: 10.1371/journal.pbio.3000597 (PMC7029882; doi:10.1371/journal.pbio.3000597)
Supplement: S4 Table — Values are labelled by the parameter values of the simulations from which they were generated (s = selection coefficient; μ = mutation rate per base pair/generation). (PDF) [file pbio.3000597.s026.pdf]

S4 Table. Average neutral equilibrium values of nucleotide site diversity (*pi*) Tajima's *D* and Kelly's Z_{nS} for our simulated populations, both without migration (*i.e.* the simulated population at equilibrium prior to experiencing a classic sweep) and with migration (*i.e.* the simulated population at equilibrium after an introgressed sweep). Values are labelled by the parameter values of the simulations from which they were generated (s = selection coefficient; μ = mutation rate per base pair/generation).

**s = 0.5, μ = 6e-07**	***pi***	**Taj*D***	Z_{nS}	**s = 0.5, μ = 6e-06**	***pi***	**Taj*D***	Z_{nS}
**Classic sweep**	0.00238	-0.51947	0.06131	**Classic sweep**	0.02236	-0.64343	0.07627
**Introgressed sweep**	0.00516	0.60160	0.13391	**Introgressed sweep**	0.04147	0.24140	0.12005
**s = 0.25, μ = 6e-07**	***pi***	**Taj*D***	Z_{nS}				
**Classic sweep**	0.00231	-0.48267	0.08545				
**Introgressed sweep**	0.00437	0.33448	0.14655				
**s = 0.1, μ = 6e-07**	***pi***	**Taj*D***	Z_{nS}				
**Classic sweep**	0.00233	-0.45685	0.07473				
**Introgressed sweep**	0.00447	0.20732	0.14413				
