# Supplementary material for: Selective sweeps on novel and introgressed variation shape mimicry loci in a butterfly adaptive radiation
Source: PLoS Biol. 2020 Feb 6;18(2):e3000597. doi: 10.1371/journal.pbio.3000597 (PMC7029882; doi:10.1371/journal.pbio.3000597)
Supplement: S5 Table — Additional relevant peaks on scaffolds are also given. Data are from SweepFinder2 [74,76] runs with background SFS estimated from background scaffolds. CLR, composite likelihood ratio; SFS, site frequency spectrum. (PDF) [file pbio.3000597.s027.pdf]

S5 Table. Position, composite likelihood-ratio statistics (CLR) and strength of selection (α, $2N_es$, and s) for the highest CLR and the smallest α value on each colour pattern scaffold (α_{min}) for the *H. melpomene*-clade. Additional relevant peaks on scaffolds are also given. Data are from SweepFinder2 [74,76] runs with background site frequency spectrum estimated from background scaffolds.

Population	Locus	Scaffold	Position	CLR	α	$2N_es$	s	Position (α_{min})	CLR (α_{min})	α_{min}	$2N_es$ (α_{min})	s (α_{min})
H. besckei	*aristaless*	Hmel201011	2488332	14	244.24	848	0.001	2623214	8	79.09	2618	0.003
H. c. chioneus	*aristaless*	Hmel201011	2637747	41	129.13	4529	0.002	2638447	38	126.42	4626	0.002
H. c. cydnides	*aristaless*	Hmel201011	2613872	106	63	8052	0.004	2613822	101	62.97	8056	0.004
H. c. weymeri gustavi	*aristaless*	Hmel201011	2598794	64	109.51	4750	0.002	2600094	39	87.83	5923	0.003
H. c. weymeri weymeri	*aristaless*	Hmel201011	2595343	122	74.63	6265	0.003	2600194	68	52.06	8981	0.004
H. c. zelinde	*aristaless*	Hmel201011	2637640	39	132.71	4674	0.002	2637640	39	132.71	4674	0.002
H. elevatus Ecuador	*aristaless*	Hmel201011	2671769	64	140.74	6525	0.002	2671769	64	140.74	6525	0.002
H. heurippa	*aristaless*	Hmel201011	2648749	125	43.49	7946	0.005	2647499	97	40.42	8550	0.006
H. m. amaryllis	*aristaless*	Hmel201011	2638339	19	254.65	3387	0.001	2725509	7	198.75	4340	0.001
H. m. cythera	*aristaless*	Hmel201011	2616846	80	71.96	8271	0.003	2623247	72	30.58	19461	0.008
H. m. ECU	*aristaless*	Hmel201011	2641567	31	202.82	3773	0.001	2641617	31	201.69	3794	0.001
H. m. malleti COL	*aristaless*	Hmel201011	2563116	33	436.43	1688	0.001	2725394	15	214.26	3439	0.001
H. m. malleti ECU	*aristaless*	Hmel201011	2643054	25	606.14	1501	0	2725514	4	194.61	4675	0.001
H. m. melpomene COL	*aristaless*	Hmel201011	2623375	200	33.27	22438	0.007	2623575	196	33.07	22575	0.007
H. m. melpomene FG	*aristaless*	Hmel201011	2518352	27	489.17	1083	0	2583809	7	171.11	3097	0.001
H. m. melpomene PAN	*aristaless*	Hmel201011	2601790	44	216.07	3070	0.001	2600890	41	111.65	5942	0.002
H. m. meriana	*aristaless*	Hmel201011	2584942	16	983.69	388	0	2483584	1	251.96	1516	0.001
H. m. nanna NORTH	*aristaless*	Hmel201011	2542627	46	158.72	3705	0.001	2545828	21	120.49	4881	0.002
H. m. nanna SOUTH	*aristaless*	Hmel201011	2753296	30	82.03	7169	0.003	2636583	27	64.35	9140	0.004
H. m. plesseni	*aristaless*	Hmel201011	2641847	21	276.52	2612	0.001	2740705	9	264.91	2727	0.001
H. m. rosina	*aristaless*	Hmel201011	2648668	57	164.68	3222	0.001	2661869	28	95.48	5558	0.002
H. m. vicina	*aristaless*	Hmel201011	2641654	33	94.72	7881	0.003	2641504	31	93.92	7948	0.003
H. m. vulcanus	*aristaless*	Hmel201011	2669703	43	249.33	1953	0.001	2661802	13	142.8	3410	0.002
H. m. xenoclea	*aristaless*	Hmel201011	2642090	24	233.79	2904	0.001	2641640	21	162.04	4189	0.001
H. pachinus	*aristaless*	Hmel201011	2702891	99	37.43	14517	0.006	2704441	44	35.6	15263	0.007
H. t. florencia	*aristaless*	Hmel201011	2669550	193	23.58	23345	0.01	2668800	108	23.34	23584	0.01
			2640195	180	38.35	14352	0.006	2640045	176	37.69	14603	0.006
H. t. linaresi	*aristaless*	Hmel201011	2634480	180	27.08	18189	0.009	2634180	127	26.92	18297	0.009
H. t. ssp. nov. ECU	*aristaless*	Hmel201011	2666249	159	35.77	16212	0.007	2664749	140	31.57	18367	0.007
H. t. thelxinoe	*aristaless*	Hmel201011	2674171	59	103.18	4818	0.002	2631367	37	98.32	5056	0.002
H. t. timareta f. contigua	*aristaless*	Hmel201011	2674005	146	35.27	14908	0.007	2673455	115	34.82	15102	0.007
H. t. timareta f. timareta	*aristaless*	Hmel201011	2674076	77	58.86	9727	0.004	2631017	64	47.35	12090	0.005
H. t. ssp. nov. COL	*aristaless*	Hmel201011	2669779	164	26.14	17359	0.009	2672479	77	23.57	19257	0.01
H. besckei	*WntA*	Hmel210004	1559377	27	108.61	1983	0.002	1566878	19	40.6	5304	0.005

**Population**	**Locus**	**Scaffold**	**Position**	**CLR**	α	$2N_e s$	s	**Position** (α_{min})	**CLR** (α_{min})	α_{min}	$2N_e s$ (α_{min})	s (α_{min})
H. c. chioneus	*WntA*	Hmel210004	1620799	161	28.33	18989	0.008	1626550	119	25.19	21352	0.009
H. c. cydnides	*WntA*	Hmel210004	1811842	173	27.05	21129	0.008	1817892	143	22.09	25874	0.01
H. c. weymeri gustavi	*WntA*	Hmel210004	1806398	348	13.78	38564	0.016	1805348	344	13.57	39160	0.016
H. c. weymeri weymeri	*WntA*	Hmel210004	1810765	237	17.17	32190	0.013	1811415	222	17.04	32431	0.013
H. c. zelinde	*WntA*	Hmel210004	1620832	128	31.44	18426	0.007	1620482	120	31.14	18602	0.007
H. elevatus ECU	*WntA*	Hmel210004	1828231	339	25.25	33900	0.009	1827631	324	24.76	34574	0.009
H. heurippa	*WntA*	Hmel210004	1560084	247	21.52	15644	0.01	1567084	239	20.14	16718	0.011
H. m. amaryllis	*WntA*	Hmel210004	1809664	186	44.02	17940	0.005	1810014	180	40.31	19589	0.006
H. m. cythera	*WntA*	Hmel210004	1811224	174	29.08	19900	0.008	1811424	171	28.97	19975	0.008
H. m. ecuadoriensis	*WntA*	Hmel210004	1848718	304	32.77	20532	0.007	1809867	113	24.11	27915	0.009
			1808717	226	24.59	27368	0.009	1809867	113	24.11	27915	0.009
H. m. malleti COL	*WntA*	Hmel210004	1621626	235	24.39	28389	0.009	1621326	170	24.26	28533	0.009
H. m. malleti ECU	*WntA*	Hmel210004	1809624	306	30.86	27223	0.007	1810125	302	30.03	27982	0.008
H. m. melpomene COL	*WntA*	Hmel210004	1631681	531	12.33	58771	0.018	1630681	251	12.3	58890	0.018
H. m. melpomene FG	*WntA*	Hmel210004	1568298	101	21.42	19513	0.01	1567298	96	21.08	19829	0.01
H. m. melpomene PAN	*WntA*	Hmel210004	1629713	479	12.61	51277	0.018	1629363	444	12.56	51484	0.018
H. m. meriana	*WntA*	Hmel210004	1855774	100	93.21	3613	0.002	1623664	80	48.71	6914	0.004
H. m. nanna NORTH	*WntA*	Hmel210004	1806251	237	10.14	53581	0.022	1807451	183	10.06	54028	0.022
H. m. nanna SOUTH	*WntA*	Hmel210004	1783553	58	15.1	35991	0.015	1784553	41	15.01	36193	0.015
H. m. plesseni	*WntA*	Hmel210004	1829355	1098	6.3	95215	0.035	1830905	1081	6.28	95549	0.035
H. m. rosina	*WntA*	Hmel210004	1568042	195	24.31	18977	0.009	1627296	44	22.33	20659	0.01
H. m. vicina	*WntA*	Hmel210004	1622166	179	24.43	29654	0.009	1620566	151	23.21	31214	0.01
H. m. vulcanus	*WntA*	Hmel210004	1625201	231	19.04	21328	0.011	1624201	126	18.97	21418	0.011
H. m. xenoclea	*WntA*	Hmel210004	1811430	971	4.54	118013	0.049	1812130	965	4.54	118070	0.049
H. pachinus	*WntA*	Hmel210004	1805390	245	11.06	47778	0.02	1805340	245	11.06	47780	0.02
H. t. florencia	*WntA*	Hmel210004	1566574	542	11.94	43044	0.018	1566474	542	11.93	43059	0.018
H. t. linaresi	*WntA*	Hmel210004	1825099	215	23.03	21135	0.01	1825249	215	23.01	21150	0.01
H. t. ssp. nov. ECU	*WntA*	Hmel210004	1571479	414	20.96	22770	0.01	1566878	397	16.37	29147	0.013
H. t. thelxinoe	*WntA*	Hmel210004	1765448	170	23.36	17044	0.009	1625441	113	14.67	27142	0.015
H. t. timareta f. contigua	*WntA*	Hmel210004	1572726	216	20.37	18943	0.011	1629379	78	12.71	30349	0.017
H. t. timareta f. timareta	*WntA*	Hmel210004	1567741	300	13.42	31394	0.016	1568691	221	13.38	31496	0.016
H. t. ssp. nov. COL	*WntA*	Hmel210004	1567277	421	12.56	37584	0.017	1880146	149	9.41	50157	0.023
H. besckei	*cortex*	Hmel215006	662442	28	56.91	5712	0.006	925699	11	10.58	30722	0.031
H. c. chioneus	*cortex*	Hmel215006	1209780	459	16.81	50815	0.021	924667	29	13.46	63454	0.026
H. c. cydnides	*cortex*	Hmel215006	1238215	236	35.36	22363	0.01	824294	8	15.26	51828	0.023
H. c. weymeri gustavi	*cortex*	Hmel215006	1329024	696	9.75	68950	0.036	1326374	343	9.57	70208	0.036
			1220571	627	14.4	46666	0.024	1225721	265	14.1	47661	0.025
H. c. weymeri weymeri	*cortex*	Hmel215006	1337975	2411	5.3	115568	0.065	1334724	2289	5.28	116071	0.065
			1218021	367	20.74	29538	0.017	1215221	325	20.08	30501	0.017
H. c. zelinde	*cortex*	Hmel215006	1218589	262	25.2	35877	0.014	924529	71	9.32	97037	0.038

**Population**	**Locus**	**Scaffold**	**Position**	**CLR**	**α**	**$2N_es$**	**s**	**Position (α_{min})**	**CLR (α_{min})**	**α_{min}**	**$2N_es$ (α_{min})**	**s (α_{min})**
H. elevatus ECU	*cortex*	Hmel215006	1563763	242	55.24	25155	0.007	923334	34	17	81744	0.021
H. heurippa	*cortex*	Hmel215006	1446977	724	16.71	26945	0.02	924205	211	7.68	58659	0.044
H. m. amaryllis	*cortex*	Hmel215006	1575917	979	10.69	75277	0.033	1581867	835	9.75	82558	0.036
H. m. cythera	*cortex*	Hmel215006	1100490	1713	3.35	205197	0.104	1090939	1369	3.29	209382	0.106
			1229846	1484	6.58	104613	0.053	1224346	1323	6.48	106226	0.054
H. m. ecuadoriensis	*cortex*	Hmel215006	1459579	335	30.74	30944	0.012	924906	110	7.51	126705	0.047
H. m. malleti COL	*cortex*	Hmel215006	1105290	335	16.2	60827	0.022	923581	88	10.88	90534	0.033
H. m. malleti ECU	*cortex*	Hmel215006	1098623	234	29.97	40361	0.012	924714	145	9.5	127251	0.038
H. m. melpomene COL	*cortex*	Hmel215006	1095016	604	12.97	68498	0.027	1095166	602	12.96	68537	0.027
H. m. melpomene FG	*cortex*	Hmel215006	1557309	506	18.61	36692	0.019	826094	12	13.69	49863	0.025
H. m. melpomene PAN	*cortex*	Hmel215006	1096916	857	5.36	164465	0.066	1089216	722	5.03	175240	0.07
H. m. meriana	*cortex*	Hmel215006	1220307	235	28.78	17789	0.012	830538	3	21.45	23866	0.016
H. m. nanna NORTH	*cortex*	Hmel215006	1460407	1350	6.79	108135	0.051	1057899	1141	2.47	297164	0.141
			1226202	1316	4.29	171062	0.081	1230152	811	4.25	172801	0.082
			1062599	1198	2.49	295257	0.14	1057899	1141	2.47	297164	0.141
			1577710	1170	6.83	107604	0.051	1570059	938	6.7	109603	0.052
H. m. nanna SOUTH	*cortex*	Hmel215006	1130468	173	7.86	93493	0.044	1123618	75	7.69	95532	0.045
H. m. plesseni	*cortex*	Hmel215006	1237265	2989	4.7	157635	0.074	1235965	2981	4.7	157702	0.074
			1366372	1090	17.92	41316	0.02	1304818	214	7.93	93420	0.044
			1447976	958	25.36	29196	0.014	1380022	742	23.55	31445	0.015
H. m. rosina	*cortex*	Hmel215006	1069455	1051	2.77	225945	0.125	1066255	871	2.75	227241	0.126
H. m. vicina	*cortex*	Hmel215006	1105326	372	6.1	145612	0.058	1091675	317	5.39	164792	0.066
H. m. vulcanus	*cortex*	Hmel215006	1200605	615	12.93	47085	0.027	1088852	171	6.18	98467	0.056
H. m. xenoclea	*cortex*	Hmel215006	1543692	1166	8.72	91594	0.04	1055866	486	6.56	121755	0.054
			1459887	1045	12.73	62761	0.028	1459687	1044	12.72	62769	0.028
H. pachinus	*cortex*	Hmel215006	1458120	570	16.83	44000	0.021	925602	111	6.76	109519	0.052
H. t. florencia	*cortex*	Hmel215006	1060718	639	8.09	93800	0.043	1068818	462	7.89	96195	0.044
H. t. linaresi	*cortex*	Hmel215006	1328608	871	11.56	60838	0.03	1329908	838	11.47	61324	0.03
H. t. ssp. nov. ECU	*cortex*	Hmel215006	1056672	532	8.3	87549	0.042	1057872	444	8.18	88791	0.043
H. t. thelxinoe	*cortex*	Hmel215006	1245780	189	69.92	9208	0.005	926617	21	19.85	32434	0.017
H. t. timareta f. contigua	*cortex*	Hmel215006	1458076	523	21.79	29909	0.016	930404	21	8.21	79348	0.042
H. t. timareta f. timareta	*cortex*	Hmel215006	1098821	613	8.35	77661	0.041	1091971	572	7.51	86375	0.046
H. t. ssp. nov. COL	*cortex*	Hmel215006	1459519	599	16.07	39527	0.022	1070451	554	5.89	107840	0.059
H. besckei	*optix*	Hmel218003	736567	26	211.68	905	0.001	868677	22	66.88	2865	0.003
H. c. chioneus	*optix*	Hmel218003	788367	132	45.54	11564	0.005	789017	129	45.12	11671	0.005
H. c. cydnides	*optix*	Hmel218003	637588	155	46.8	10480	0.005	789794	131	42.03	11670	0.005
H. c. weymeri gustavi	*optix*	Hmel218003	624737	176	38.09	11148	0.006	625137	173	38	11175	0.006
H. c. weymeri weymeri	*optix*	Hmel218003	1019652	220	102.97	3284	0.002	786128	122	53.71	6297	0.004
H. c. zelinde	*optix*	Hmel218003	789114	116	48.94	10602	0.004	789764	112	48.27	10749	0.005
H. elevatus ECU	*optix*	Hmel218003	787076	224	44.11	15829	0.005	857131	68	39.13	17844	0.006

**Population**	**Locus**	**Scaffold**	**Position**	**CLR**	α	**2$N_e s$**	s	**Position (α_{min})**	**CLR (α_{min})**	α_{min}	**2$N_e s$ (α_{min})**	s **(α_{min})**
H. heurippa	*optix*	Hmel218003	857729	525	18.23	11544	0.011	853979	489	17.77	11848	0.012
			781223	481	27.33	7701	0.008	785574	435	24.94	8438	0.008
H. m. amaryllis	*optix*	Hmel218003	786183	357	32.58	14300	0.007	784733	279	32.07	14528	0.007
H. m. cythera	*optix*	Hmel218003	838145	736	11.82	33955	0.018	833645	509	11.76	34137	0.018
H. m. ECU	*optix*	Hmel218003	811137	200	47.73	10123	0.005	842388	17	44.12	10951	0.005
H. m. malleti COL	*optix*	Hmel218003	814383	329	28.73	17414	0.008	843034	206	22.26	22480	0.01
H. m. malleti ECU	*optix*	Hmel218003	814624	255	53.69	12199	0.004	843427	189	31.04	21102	0.007
H. m. melpomene COL	*optix*	Hmel218003	672284	119	138.06	3609	0.002	623580	95	55.09	9046	0.004
H. m. melpomene FG	*optix*	Hmel218003	649462	141	49.87	7416	0.004	645511	80	45.72	8089	0.005
H. m. melpomene PAN	*optix*	Hmel218003	838170	647	13.74	33226	0.016	837219	529	13.72	33277	0.016
H. m. meriana	*optix*	Hmel218003	801534	1250	9.45	35360	0.023	811585	826	9.28	35993	0.023
H. m. nanna NORTH	*optix*	Hmel218003	782525	306	38.5	12092	0.006	646316	90	27.67	16823	0.008
H. m. nanna SOUTH	*optix*	Hmel218003	726637	41	65.72	7084	0.003	727838	38	62.99	7391	0.003
H. m. plesseni	*optix*	Hmel218003	783431	2371	6.97	41978	0.03	655174	1937	5.91	49501	0.036
			643924	2174	6.07	48223	0.035	655174	1937	5.91	49501	0.036
			732278	1638	6.21	47109	0.034	728328	1466	6.17	47423	0.034
H. m. rosina	*optix*	Hmel218003	847932	585	13.15	26357	0.016	840982	463	12.78	27129	0.017
H. m. vicina	*optix*	Hmel218003	790999	475	10.58	47091	0.021	796800	320	10.33	48254	0.021
H. m. vulcanus	*optix*	Hmel218003	848005	759	8.83	32135	0.024	840154	609	8.53	33270	0.025
H. m. xenoclea	*optix*	Hmel218003	727532	1182	9.74	37910	0.022	725882	917	9.73	37935	0.022
H. pachinus	*optix*	Hmel218003	648265	289	30.16	14716	0.007	646315	193	29.92	14838	0.007
H. t. florencia	*optix*	Hmel218003	705381	201	84.98	4616	0.003	674830	70	44.94	8729	0.005
H. t. linaresi	*optix*	Hmel218003	803436	408	27.11	12138	0.008	802136	218	26.9	12235	0.008
H. t. ssp. nov. ECU	*optix*	Hmel218003	705531	186	99.32	3833	0.002	671379	46	56.44	6745	0.004
H. t. thelxinoe	*optix*	Hmel218003	788276	66	117.15	2773	0.002	788276	66	117.15	2773	0.002
H. t. timareta f. contigua	*optix*	Hmel218003	941544	152	52.4	6196	0.004	941244	147	52.33	6205	0.004
H. t. timareta f. timareta	*optix*	Hmel218003	864840	157	50.27	6313	0.004	864840	157	50.27	6313	0.004
H. t. ssp. nov. COL	*optix*	Hmel218003	597476	284	36.8	8193	0.006	616977	186	24.29	12411	0.009
