# Supplementary material for: Selective sweeps on novel and introgressed variation shape mimicry loci in a butterfly adaptive radiation
Source: PLoS Biol. 2020 Feb 6;18(2):e3000597. doi: 10.1371/journal.pbio.3000597 (PMC7029882; doi:10.1371/journal.pbio.3000597)
Supplement: S6 Table — Data are from SweepFinder2 [74,76] runs with background SFS estimated from background scaffolds. CLR, composite likelihood ratio; SFS, site frequency spectrum. (PDF) [file pbio.3000597.s028.pdf]

S6 Table. Position, composite likelihood-ratio statistics (CLR) and strength of selection (*α*, 2*N*$_e$*s*, and *s*) for the highest CLR and the smallest *α* value on each background scaffold ($α_{min}$) for the *H. melpomene*-clade. Data are from SweepFinder2 [74,76] runs with background site frequency spectrum estimated from background scaffolds.

Population	Scaffold	Position	CLR	$α$	$2N_es$	s	Position ($α_{min}$)	CLR ($α_{min}$)	$α_{min}$	$2N_es$ ($α_{min}$)	s ($α_{min}$)
H. besckei	Hmel204017	2202171	10	549.99	649	0.001	2198120	7	42.25	8445	0.008
H. c. chioneus	Hmel204017	2299755	24	461.75	2371	0.001	1959329	13	163.28	6705	0.002
H. c. cydnides	Hmel204017	2298643	25	194.08	6303	0.002	1958116	20	89.26	13704	0.004
H. c. weymeri gustavi	Hmel204017	2003132	40	121.06	10105	0.003	2003332	35	118.42	10330	0.003
H. c. weymeri weymeri	Hmel204017	2019842	42	192.6	6144	0.002	2004090	29	91.35	12954	0.004
H. c. zelinde	Hmel204017	2016795	31	193.11	5709	0.002	2053101	10	75.43	14614	0.005
H. elevatus Ecuador	Hmel204017	2255583	12	484.36	2327	0.001	1958500	4	103.58	10880	0.004
H. heurippa	Hmel204017	2117854	35	206.51	8319	0.002	2109703	18	67.29	25532	0.006
H. m. amaryllis	Hmel204017	2095349	92	72.69	11239	0.005	2110351	4	54.96	14865	0.007
H. m. cythera	Hmel204017	1957699	23	226.1	6867	0.002	2109212	3	101.71	15266	0.004
H. m. ECU	Hmel204017	2115425	28	163.27	7507	0.002	2109574	11	77.18	15879	0.005
H. m. malleti COL	Hmel204017	2119953	19	540.47	2674	0.001	1958384	14	99.86	14472	0.004
H. m. malleti ECU	Hmel204017	1940231	23	406.94	3468	0.001	1955983	10	149.31	9451	0.003
H. m. melpomene COL	Hmel204017	1940194	21	490.45	3550	0.001	2108911	1	111.29	15644	0.003
H. m. melpomene FG	Hmel204017	2070510	17	1052.51	1326	0	2054159	6	159.26	8763	0.002
H. m. melpomene PAN	Hmel204017	1958471	37	102.29	13645	0.004	1958371	37	101.91	13696	0.004
H. m. meriana	Hmel204017	1938159	36	314.03	1732	0.001	2054221	4	67.64	8043	0.005
H. m. nanna NORTH	Hmel204017	1962630	17	206.08	5183	0.002	2110187	1	104.45	10226	0.004
H. m. nanna SOUTH	Hmel204017	1987449	23	85.41	12506	0.004	1959797	20	30.51	35006	0.012
H. m. plesseni	Hmel204017	1960131	24	138	10351	0.003	1958431	14	107.91	13237	0.004
H. m. rosina	Hmel204017	2070277	19	565.94	1716	0.001	2197537	1	191.2	5078	0.002
H. m. vicina	Hmel204017	2028490	25	250.5	5571	0.002	1957880	21	69.35	20124	0.005
H. m. vulcanus	Hmel204017	1958592	45	68.73	12826	0.005	1958592	45	68.73	12826	0.005
H. m. xenoclea	Hmel204017	1940108	20	427.87	3013	0.001	1957909	7	220.11	5858	0.002
H. pachinus	Hmel204017	2115316	40	102.78	11624	0.004	2109316	37	44.2	27031	0.008
H. t. florencia	Hmel204017	1967332	37	143.92	7590	0.003	2065692	10	108.37	10080	0.003
H. t. linaresi	Hmel204017	1859422	44	75.94	15138	0.005	1859622	41	74.32	15468	0.005
H. t. ssp. nov. ECU	Hmel204017	1967446	23	164.5	6944	0.002	2053904	2	142.86	7996	0.003
H. t. thelxinoe	Hmel204017	1992794	13	370.4	2639	0.001	2200268	0	205.27	4762	0.002
H. t. timareta f. contigua	Hmel204017	1967333	29	177.71	5673	0.002	1894174	27	153.85	6553	0.002
H. t. timareta f. timareta	Hmel204017	1967317	29	139.95	7689	0.003	1967917	28	129.38	8316	0.003
H. t. ssp. nov. COL	Hmel204017	1967382	31	129.08	7875	0.003	2066095	26	67.81	14992	0.005
H. besckei	Hmel206006	623821	32	96.67	2936	0.003	446344	6	78.83	3600	0.003
H. c. chioneus	Hmel206006	552281	35	206.11	4284	0.001	635186	13	106.84	8264	0.003
H. c. cydnides	Hmel206006	621246	49	185.05	4819	0.001	636047	17	90.71	9830	0.003

Population	Scaffold	Position	CLR	α	$2N_es$	s	Position (α_{min})	CLR (α_{min})	α_{min}	$2N_es$ (α_{min})	s (α_{min})
H. c. weymeri gustavi	Hmel206006	551591	24	285.25	2877	0.001	635597	6	141.7	5793	0.002
H. c. weymeri weymeri	Hmel206006	358310	38	313.61	2521	0.001	602687	4	211.68	3735	0.001
H. c. zelinde	Hmel206006	625531	20	322.96	2516	0.001	547874	12	195.33	4160	0.001
H. elevatus Ecuador	Hmel206006	625339	67	165.96	7938	0.002	625089	56	158.23	8326	0.002
H. heurippa	Hmel206006	692174	209	68.12	8410	0.004	622815	113	56	10232	0.005
H. m. amaryllis	Hmel206006	792036	13	6498.34	199	0	602713	3	266.71	4857	0.001
H. m. cythera	Hmel206006	327697	35	313.66	2881	0.001	582730	1	294.86	3064	0.001
H. m. ECU	Hmel206006	754627	18	1234.34	843	0	604468	6	289.65	3594	0.001
H. m. malleti COL	Hmel206006	519734	18	772.31	1331	0	330318	6	330.27	3112	0.001
H. m. malleti ECU	Hmel206006	520036	23	608.97	2203	0	604299	11	168.4	7967	0.002
H. m. melpomene COL	Hmel206006	435316	77	286.56	3472	0.001	635727	14	142	7006	0.002
H. m. melpomene FG	Hmel206006	748824	30	400.07	1622	0.001	603065	21	82.02	7913	0.003
H. m. melpomene PAN	Hmel206006	550698	67	79.7	12387	0.003	550848	42	79.35	12441	0.003
H. m. meriana	Hmel206006	398903	43	178.98	2537	0.001	399353	32	168.46	2696	0.002
H. m. nanna NORTH	Hmel206006	624824	52	71.95	11590	0.004	624874	52	71.88	11601	0.004
H. m. nanna SOUTH	Hmel206006	697150	29	65.89	12655	0.004	593689	20	55.63	14990	0.005
H. m. plesseni	Hmel206006	542803	28	533.96	1896	0.001	604409	9	195.65	5174	0.001
H. m. rosina	Hmel206006	435387	31	485.67	1638	0.001	485890	11	311.11	2558	0.001
H. m. vicina	Hmel206006	596917	19	304.47	3268	0.001	635572	8	102.6	9697	0.003
H. m. vulcanus	Hmel206006	620645	58	160.28	4176	0.002	635647	13	137.72	4860	0.002
H. m. xenoclea	Hmel206006	602412	24	189.92	5286	0.001	602712	20	173.66	5781	0.002
H. pachinus	Hmel206006	602775	56	87.16	9762	0.003	603575	53	82.35	10333	0.003
H. t. florencia	Hmel206006	619768	71	144.46	5701	0.002	603667	7	91.83	8967	0.003
H. t. linaresi	Hmel206006	647619	44	266.93	2946	0.001	647569	41	266.8	2947	0.001
H. t. ssp. nov. ECU	Hmel206006	622609	76	118.17	7126	0.002	604357	24	103.87	8107	0.003
H. t. thelxinoe	Hmel206006	619714	48	198.45	3860	0.001	619764	47	197.9	3871	0.001
H. t. timareta f. contigua	Hmel206006	739720	27	395.99	1932	0.001	736870	5	207.58	3685	0.001
H. t. timareta f. timareta	Hmel206006	739820	24	384.53	2082	0.001	503640	7	342.81	2335	0.001
H. t. ssp. nov. COL	Hmel206006	600649	106	54.24	12566	0.005	599899	105	53.68	12697	0.005
H. besckei	Hmel208051	754245	17	64.59	5134	0.006	755845	11	47.33	7006	0.008
H. c. chioneus	Hmel208051	1042680	28	137.66	8279	0.003	911421	19	66.71	17083	0.006
H. c. cydnides	Hmel208051	1043501	33	101.13	10528	0.004	1043451	30	99.58	10692	0.004
H. c. weymeri gustavi	Hmel208051	1039926	62	59.32	17382	0.007	1040376	4	55.87	18455	0.007
H. c. weymeri weymeri	Hmel208051	1040038	29	146.11	6744	0.003	638197	13	123.9	7953	0.003
H. c. zelinde	Hmel208051	1042582	23	171.2	6554	0.002	911322	8	87.76	12785	0.005
H. elevatus Ecuador	Hmel208051	1044514	68	86.19	18877	0.005	929499	23	61.66	26386	0.007
H. heurippa	Hmel208051	982755	447	11.85	63379	0.033	984105	268	11.46	65530	0.034
H. m. amaryllis	Hmel208051	1048965	19	674.01	2446	0.001	1045265	13	186.14	8856	0.002
H. m. cythera	Hmel208051	846537	27	395.77	3243	0.001	955998	25	117.17	10955	0.003
H. m. ECU	Hmel208051	1024299	40	82.15	16900	0.005	1024299	40	82.15	16900	0.005

Population	Scaffold	Position	CLR	α	$2N_es$	s	Position (α_{min})	CLR (α_{min})	α_{min}	$2N_es$ (α_{min})	s (α_{min})
H. m. malleti COL	Hmel208051	1049946	19	183.34	7932	0.002	1068899	14	182.57	7965	0.002
H. m. malleti ECU	Hmel208051	1050196	14	908.1	1964	0	1023894	2	115.65	15426	0.004
H. m. melpomene COL	Hmel208051	1031498	23	184.04	7475	0.002	745420	4	161.2	8534	0.003
H. m. melpomene FG	Hmel208051	1022462	13	136.64	6828	0.003	1023112	8	118.53	7871	0.003
H. m. melpomene PAN	Hmel208051	992145	28	374.68	3580	0.001	905289	12	124.84	10743	0.003
H. m. meriana	Hmel208051	1024688	41	84.4	8036	0.005	1053241	34	77.92	8705	0.005
H. m. nanna NORTH	Hmel208051	1039701	268	15.12	71234	0.026	1042701	165	14.81	72711	0.027
H. m. nanna SOUTH	Hmel208051	1073274	25	40.14	26835	0.01	761403	14	18.38	58609	0.022
H. m. plesseni	Hmel208051	1049999	19	230.55	6532	0.002	1023996	18	91.79	16404	0.004
H. m. rosina	Hmel208051	903527	24	205.05	4694	0.002	904777	6	132.63	7256	0.003
H. m. vicina	Hmel208051	1053006	45	48.28	28493	0.008	1053256	43	47.58	28915	0.009
H. m. vulcanus	Hmel208051	986477	43	59.01	14524	0.007	986427	43	58.93	14544	0.007
H. m. xenoclea	Hmel208051	1049965	26	185.01	7211	0.002	910549	4	86.3	15459	0.005
H. pachinus	Hmel208051	957473	52	62.25	17632	0.006	957473	52	62.25	17632	0.006
H. t. florencia	Hmel208051	1044370	163	27.82	36817	0.014	1034118	78	25.36	40393	0.016
H. t. linaresi	Hmel208051	1053301	78	61.85	16854	0.006	1048751	37	58.96	17679	0.007
H. t. ssp. nov. ECU	Hmel208051	1045877	144	29.56	35705	0.014	1046027	73	29.51	35764	0.014
H. t. thelxinoe	Hmel208051	909967	59	61.29	14753	0.006	910717	55	51.43	17581	0.008
H. t. timareta f. contigua	Hmel208051	1045354	83	42.15	21556	0.009	1045254	82	42.07	21597	0.009
H. t. timareta f. timareta	Hmel208051	1043033	87	34.74	28376	0.011	1042433	35	34.64	28465	0.011
H. t. ssp. nov. COL	Hmel208051	1024784	151	23.07	40454	0.017	1031634	97	22.06	42310	0.018
H. besckei	Hmel219003	5392785	10	250.9	995	0.001	5656167	7	239.49	1043	0.001
H. c. chioneus	Hmel219003	5569780	86	88.48	8060	0.003	5569830	86	88.47	8061	0.003
H. c. cydnides	Hmel219003	5396768	123	85.52	7800	0.003	5396818	123	85.49	7803	0.003
H. c. weymeri gustavi	Hmel219003	5538911	101	112.36	5544	0.002	5286426	32	98.65	6314	0.002
H. c. weymeri weymeri	Hmel219003	5553535	283	38.09	15583	0.006	5554435	278	37.93	15647	0.006
H. c. zelinde	Hmel219003	5570079	125	62.53	11161	0.004	5570329	82	62.3	11203	0.004
H. elevatus Ecuador	Hmel219003	5568930	92	252.97	4436	0.001	5576831	37	143.22	7835	0.002
H. heurippa	Hmel219003	5611280	129	142.67	2523	0.002	5428619	118	91.69	3926	0.002
H. m. amaryllis	Hmel219003	5555079	39	336.76	3004	0.001	5254364	7	224.1	4514	0.001
H. m. cythera	Hmel219003	5246608	277	39.17	19565	0.006	5245458	210	38.8	19754	0.006
H. m. ECU	Hmel219003	5576937	87	80.03	11156	0.003	5577787	84	78.91	11315	0.003
H. m. malleti COL	Hmel219003	5252414	33	289.02	2908	0.001	5254464	30	157.13	5350	0.002
H. m. malleti ECU	Hmel219003	5567800	50	384.41	2743	0.001	5576601	42	116.57	9045	0.002
H. m. melpomene COL	Hmel219003	5588298	31	788.28	1086	0	5286460	2	201.48	4249	0.001
H. m. melpomene FG	Hmel219003	5554226	57	154.81	3433	0.002	5554426	54	154.6	3438	0.002
H. m. melpomene PAN	Hmel219003	5552111	99	99.07	8155	0.002	5254367	91	95	8505	0.003
H. m. meriana	Hmel219003	5475294	51	322.83	1212	0.001	5244725	34	142.27	2750	0.002
H. m. nanna NORTH	Hmel219003	5576562	243	25.98	25617	0.009	5573512	121	25.67	25933	0.009
H. m. nanna SOUTH	Hmel219003	5597249	88	51.91	12823	0.005	5607750	49	42.09	15816	0.006

**Population**	**Scaffold**	**Position**	**CLR**	**α**	**$2N_es$**	**s**	**Position (α_{min})**	**CLR (α_{min})**	**α_{min}**	**$2N_es$ (α_{min})**	**s (α_{min})**
H. m. plesseni	Hmel219003	5254487	56	99.24	8569	0.002	5254537	56	99.09	8582	0.002
H. m. rosina	Hmel219003	5551670	76	138.55	4565	0.002	5255279	55	120.95	5229	0.002
H. m. vicina	Hmel219003	5550947	50	111.2	7699	0.002	5551797	47	107.56	7960	0.002
H. m. vulcanus	Hmel219003	5553765	71	200.24	2712	0.001	5577768	39	135.95	3994	0.002
H. m. xenoclea	Hmel219003	5576910	52	59	13791	0.004	5577810	49	57.61	14123	0.004
H. pachinus	Hmel219003	5551837	138	78.62	8787	0.003	5555187	133	66.15	10443	0.004
H. t. florencia	Hmel219003	5715417	155	86.73	6303	0.003	5251087	83	44.97	12156	0.005
H. t. linaresi	Hmel219003	5481153	128	135.29	3870	0.002	5388590	90	77.48	6757	0.003
H. t. ssp. nov. ECU	Hmel219003	5715333	177	78.73	7015	0.003	5251188	57	59.79	9238	0.004
H. t. thelxinoe	Hmel219003	5611298	177	88.47	4333	0.003	5611048	161	88.12	4350	0.003
H. t. timareta f. contigua	Hmel219003	5250567	242	28.08	16568	0.008	5250067	200	27.78	16749	0.008
H. t. timareta f. timareta	Hmel219003	5551811	161	75.74	6270	0.003	5251121	70	59.27	8012	0.004
H. t. ssp. nov. COL	Hmel219003	5583888	553	18.15	24628	0.013	5575787	420	16.8	26600	0.014
