# Supplementary material for: Selective sweeps on novel and introgressed variation shape mimicry loci in a butterfly adaptive radiation
Source: PLoS Biol. 2020 Feb 6;18(2):e3000597. doi: 10.1371/journal.pbio.3000597 (PMC7029882; doi:10.1371/journal.pbio.3000597)
Supplement: S7 Table — Additional relevant peaks on scaffolds are also given. Data are from SweepFinder2 [74,76] runs with background SFS estimated from background and colour pattern scaffolds. CLR, composite likelihood ratio; SFS, site frequency spectrum. (PDF) [file pbio.3000597.s029.pdf]

S7 Table. Position, composite likelihood-ratio statistics (CLR) and strength of selection (α, $2N_es$, and s) for the highest CLR and the smallest α value on each colour pattern scaffold (α_{min}) for the *H. melpomene*-clade. Additional relevant peaks on scaffolds are also given. Data are from SweepFinder2 [74,76] runs with background site frequency spectrum estimated from background and colour pattern scaffolds.

Population	Locus	Scaffold	Position	CLR	α	$2N_es$	s	Position (α_{min})	CLR (α_{min})	α_{min}	$2N_es$ (α_{min})	s (α_{min})
H. besckei	*aristaless*	Hmel201011	2488332	15	245.38	844	0.001	2623264	8	78.98	2621	0.003
H. c. chioneus	*aristaless*	Hmel201011	2637797	28	162.3	3603	0.001	2637697	27	157.39	3716	0.001
H. c. cydnides	*aristaless*	Hmel201011	2613872	82	70.24	7222	0.003	2613872	82	70.24	7222	0.003
H. c. weymeri gustavi	*aristaless*	Hmel201011	2598794	52	129.02	4032	0.002	2600094	27	100.1	5197	0.002
H. c. weymeri weymeri	*aristaless*	Hmel201011	2595343	95	87.66	5334	0.003	2600144	37	62.72	7455	0.004
H. c. zelinde	*aristaless*	Hmel201011	2637640	28	163.96	3783	0.001	2637640	28	163.96	3783	0.001
H. elevatus Ecuador	*aristaless*	Hmel201011	2671769	42	162.82	5641	0.001	2671769	42	162.82	5641	0.001
H. heurippa	*aristaless*	Hmel201011	2648749	85	50.23	6880	0.005	2647699	62	46.58	7420	0.005
H. m. amaryllis	*aristaless*	Hmel201011	2538415	9	652817.2	1	0	2740662	4	338.7	2546	0.001
H. m. cythera	*aristaless*	Hmel201011	2616846	54	91.95	6473	0.003	2623397	19	49.58	12004	0.005
H. m. ECU	*aristaless*	Hmel201011	2642217	21	353.41	2165	0.001	2641617	20	247.76	3088	0.001
H. m. malleti COL	*aristaless*	Hmel201011	2563116	26	471.86	1562	0.001	2725444	9	233.21	3160	0.001
H. m. malleti ECU	*aristaless*	Hmel201011	2643054	19	657.64	1383	0	2740666	0	291.95	3116	0.001
H. m. melpomene COL	*aristaless*	Hmel201011	2623275	116	43.1	17319	0.006	2623525	112	42.4	17604	0.006
H. m. melpomene FG	*aristaless*	Hmel201011	2518352	23	521.78	1016	0	2583859	3	216.89	2444	0.001
H. m. melpomene PAN	*aristaless*	Hmel201011	2601790	31	317.04	2092	0.001	2600840	20	155.34	4271	0.002
H. m. meriana	*aristaless*	Hmel201011	2584942	15	1029.57	371	0	2483534	3	275.37	1387	0.001
H. m. nanna NORTH	*aristaless*	Hmel201011	2542627	29	206	2855	0.001	2545678	10	196.89	2987	0.001
H. m. nanna SOUTH	*aristaless*	Hmel201011	2635733	24	77.1	7628	0.003	2636533	21	75.23	7818	0.003
H. m. plesseni	*aristaless*	Hmel201011	2716853	11	667.16	1083	0	2740655	1	325.36	2220	0.001
H. m. rosina	*aristaless*	Hmel201011	2648668	40	192.06	2763	0.001	2661869	15	120.39	4408	0.002
H. m. vicina	*aristaless*	Hmel201011	2643104	19	266.38	2802	0.001	2641554	16	133.94	5573	0.002
H. m. vulcanus	*aristaless*	Hmel201011	2669653	31	319.09	1526	0.001	2661802	4	236.26	2061	0.001
H. m. xenoclea	*aristaless*	Hmel201011	2643390	16	981.57	692	0	2641640	7	250.65	2708	0.001
H. pachinus	*aristaless*	Hmel201011	2637780	52	158.95	3419	0.001	2703291	40	68.95	7882	0.003
H. t. florencia	*aristaless*	Hmel201011	2640195	141	42.99	12804	0.005	2669300	130	27.3	20158	0.009
			2669550	134	27.48	20032	0.009	2669300	130	27.3	20158	0.009
H. t. linaresi	*aristaless*	Hmel201011	2634430	114	31.16	15805	0.007	2634180	62	30.95	15915	0.008
H. t. ssp. nov. ECU	*aristaless*	Hmel201011	2666249	101	49.94	11611	0.005	2664899	79	44.49	13033	0.005
H. t. thelxinoe	*aristaless*	Hmel201011	2674221	51	113.09	4396	0.002	2631467	36	108.05	4601	0.002
H. t. timareta f. contigua	*aristaless*	Hmel201011	2674055	105	44.53	11810	0.005	2673705	102	43.95	11966	0.005
H. t. timareta f. timareta	*aristaless*	Hmel201011	2674126	53	69.97	8183	0.003	2630917	34	54.97	10415	0.004
H. t. ssp. nov. COL	*aristaless*	Hmel201011	2669729	110	36.41	12465	0.006	2672229	81	34.16	13286	0.007
H. besckei	*WntA*	Hmel210004	1559377	28	110.61	1947	0.002	1566878	21	40.39	5331	0.005

Population	Locus	Scaffold	Position	CLR	α	$2N_es$	s	Position (α_{min})	CLR (α_{min})	α_{min}	$2N_es$ (α_{min})	s (α_{min})
H. c. chioneus	*WntA*	Hmel210004	1620799	111	34.86	15432	0.006	1626500	70	32.03	16795	0.007
H. c. cydnides	*WntA*	Hmel210004	1811792	134	31.47	18160	0.007	1817892	101	25.37	22521	0.009
H. c. weymeri gustavi	*WntA*	Hmel210004	1806398	257	16.57	32081	0.013	1805198	251	16.18	32860	0.014
H. c. weymeri weymeri	*WntA*	Hmel210004	1810315	166	22.13	24964	0.01	1811415	151	21.65	25519	0.01
H. c. zelinde	*WntA*	Hmel210004	1620832	95	36.08	16055	0.006	1620482	87	35.61	16269	0.006
H. elevatus ECU	*WntA*	Hmel210004	1828331	247	29.65	28873	0.008	1827831	239	29.18	29337	0.008
H. heurippa	*WntA*	Hmel210004	1560284	186	24.93	13503	0.009	1567234	179	23.17	14532	0.009
H. m. amaryllis	*WntA*	Hmel210004	1809664	121	67.34	11727	0.003	1810164	104	58.95	13396	0.004
H. m. cythera	*WntA*	Hmel210004	1811174	96	44.81	12915	0.005	1811424	94	44.32	13059	0.005
H. m. ecuadoriensis	*WntA*	Hmel210004	1848718	232	38.28	17577	0.006	1621560	132	30.73	21896	0.007
			1808717	139	34.32	19609	0.007	1810117	85	33.01	20386	0.007
H. m. malleti COL	*WntA*	Hmel210004	1621626	164	37.02	18699	0.006	1626276	20	33.64	20578	0.007
H. m. malleti ECU	*WntA*	Hmel210004	1809624	238	34.8	24145	0.007	1810125	234	34.02	24694	0.007
H. m. melpomene COL	*WntA*	Hmel210004	1631681	334	14.47	50043	0.016	1631731	333	14.47	50070	0.016
H. m. melpomene FG	*WntA*	Hmel210004	1568298	63	25.08	16668	0.009	1567398	59	24.61	16987	0.009
H. m. melpomene PAN	*WntA*	Hmel210004	1629713	314	16.29	39705	0.014	1629363	279	16.23	39842	0.014
H. m. meriana	*WntA*	Hmel210004	1855774	88	105.24	3200	0.002	1623664	61	54.24	6209	0.004
H. m. nanna NORTH	*WntA*	Hmel210004	1806151	99	17.3	31418	0.013	1816252	65	16.76	32425	0.013
H. m. nanna SOUTH	*WntA*	Hmel210004	1783553	38	19.94	27254	0.011	1782353	8	19.78	27473	0.011
H. m. plesseni	*WntA*	Hmel210004	1851807	649	9.5	63153	0.023	1828905	631	8.18	73376	0.027
H. m. rosina	*WntA*	Hmel210004	1567842	123	29.7	15534	0.007	1568692	73	29.45	15666	0.007
H. m. vicina	*WntA*	Hmel210004	1622166	125	33.27	21769	0.007	1620716	98	31.37	23090	0.007
H. m. vulcanus	*WntA*	Hmel210004	1625201	132	23.5	17282	0.009	1626051	101	23.15	17550	0.009
H. m. xenoclea	*WntA*	Hmel210004	1811380	487	6.17	86938	0.036	1811780	485	6.17	86959	0.036
H. pachinus	*WntA*	Hmel210004	1810240	118	20.15	26224	0.011	1805240	110	17.34	30483	0.013
H. t. florencia	*WntA*	Hmel210004	1566624	406	13.48	38127	0.016	1566424	405	13.47	38158	0.016
H. t. linaresi	*WntA*	Hmel210004	1825099	156	26.85	18128	0.008	1825199	156	26.84	18132	0.008
H. t. ssp. nov. ECU	*WntA*	Hmel210004	1571479	266	24.47	19504	0.009	1567328	242	20.54	23229	0.011
H. t. thelxinoe	*WntA*	Hmel210004	1765448	124	26.67	14929	0.008	1765348	122	26.64	14946	0.008
H. t. timareta f. contigua	*WntA*	Hmel210004	1573226	148	27.87	13841	0.008	1567926	125	21.56	17892	0.01
H. t. timareta f. timareta	*WntA*	Hmel210004	1567591	197	17.02	24745	0.013	1567891	197	17	24774	0.013
H. t. ssp. nov. COL	*WntA*	Hmel210004	1567227	303	15.57	30304	0.014	1866345	169	13.17	35834	0.017
H. besckei	*cortex*	Hmel215006	662442	27	58.7	5537	0.006	925499	11	10.3	31569	0.032
H. c. chioneus	*cortex*	Hmel215006	1209730	323	20.92	40839	0.017	923867	7	19.71	43356	0.018
H. c. cydnides	*cortex*	Hmel215006	1238415	167	51.71	15293	0.007	923849	9	19.71	40124	0.018
H. c. weymeri gustavi	*cortex*	Hmel215006	1220571	444	19.58	34335	0.018	1326374	34	13.15	51123	0.026
			1329174	393	13.31	50508	0.026	1326374	34	13.15	51123	0.026
H. c. weymeri weymeri	*cortex*	Hmel215006	1337825	1791	6.21	98669	0.056	1334724	1673	6.2	98841	0.056
			1218021	254	30.64	19992	0.011	1215371	217	30.09	20361	0.011
H. c. zelinde	*cortex*	Hmel215006	1218639	189	30.72	29434	0.012	923979	36	12.76	70863	0.028

**Population**	**Locus**	**Scaffold**	**Position**	**CLR**	α	$2N_es$	s	**Position (α_{min})**	**CLR (α_{min})**	α_{min}	$2N_es$ (α_{min})	s (α_{min})
H. elevatus ECU	*cortex*	Hmel215006	1563763	150	71.78	19358	0.005	923634	6	24.91	55787	0.015
H. heurippa	*cortex*	Hmel215006	1447077	482	20.11	22385	0.017	924905	146	8.93	50418	0.038
H. m. amaryllis	*cortex*	Hmel215006	1575917	453	13.88	57985	0.025	1581267	296	12.47	64578	0.028
H. m. cythera	*cortex*	Hmel215006	1105740	828	5.43	126643	0.064	1094539	588	5.04	136563	0.069
			1231296	789	10.23	67256	0.034	1227646	592	10.01	68720	0.035
H. m. ecuadoriensis	*cortex*	Hmel215006	1459529	187	38.58	24655	0.009	923556	49	13.77	69097	0.026
H. m. malleti COL	*cortex*	Hmel215006	1105390	237	19.09	51609	0.019	924031	51	12.91	76296	0.028
H. m. malleti ECU	*cortex*	Hmel215006	1098573	150	40.38	29953	0.009	924964	73	11.52	104998	0.031
H. m. melpomene COL	*cortex*	Hmel215006	1095016	413	15.2	58430	0.023	1095166	411	15.19	58466	0.023
H. m. melpomene FG	*cortex*	Hmel215006	1557309	356	22.33	30582	0.016	827794	1	17.85	38264	0.019
H. m. melpomene PAN	*cortex*	Hmel215006	1096966	434	8.02	109921	0.044	1093116	286	7.68	114713	0.046
H. m. meriana	*cortex*	Hmel215006	1220357	193	37.36	13704	0.009	921392	18	25.52	20061	0.013
H. m. nanna NORTH	*cortex*	Hmel215006	1460407	772	11.55	63590	0.03	1065699	408	4.68	157001	0.075
			1226102	655	7.43	98857	0.047	1225352	600	7.41	99216	0.047
			1062599	561	4.72	155677	0.074	1065699	408	4.68	157001	0.075
			1578060	619	10.59	69363	0.033	1574709	411	10.42	70512	0.034
H. m. nanna SOUTH	*cortex*	Hmel215006	1130468	118	10.85	67707	0.032	1123618	22	10.65	69004	0.033
H. m. plesseni	*cortex*	Hmel215006	1237065	1564	6.45	114884	0.054	1236815	1563	6.44	114895	0.054
			1366372	635	26.2	28268	0.013	1366822	572	26.17	28298	0.013
			1447976	557	35.77	20702	0.01	1448876	542	35.58	20809	0.01
H. m. rosina	*cortex*	Hmel215006	1069405	378	4.56	137130	0.076	1069005	338	4.55	137581	0.076
H. m. vicina	*cortex*	Hmel215006	1396187	207	30.26	29354	0.012	1094225	47	11.11	79959	0.032
H. m. vulcanus	*cortex*	Hmel215006	1200505	383	18.62	32697	0.019	1106402	51	12.84	47409	0.027
H. m. xenoclea	*cortex*	Hmel215006	1543692	607	15.34	52052	0.023	1055866	165	10.76	74197	0.033
			1459937	563	18.7	42714	0.019	1459787	563	18.7	42717	0.019
H. pachinus	*cortex*	Hmel215006	1457620	321	24.43	30316	0.014	924302	41	10.62	69733	0.033
H. t. florencia	*cortex*	Hmel215006	1060668	486	10.3	73712	0.034	1060918	464	10.28	73839	0.034
H. t. linaresi	*cortex*	Hmel215006	1328458	539	14.27	49286	0.024	1329958	499	14.04	50085	0.025
H. t. ssp. nov. ECU	*cortex*	Hmel215006	1056672	368	10	72662	0.035	1057272	221	9.92	73247	0.035
H. t. thelxinoe	*cortex*	Hmel215006	1245780	150	74.7	8618	0.005	928117	8	27.04	23811	0.013
H. t. timareta f. contigua	*cortex*	Hmel215006	1457826	356	25.71	25342	0.013	1094861	327	11.82	55133	0.029
H. t. timareta f. timareta	*cortex*	Hmel215006	1098871	412	10.51	61702	0.033	1094021	383	9.85	65876	0.035
H. t. ssp. nov. COL	*cortex*	Hmel215006	1062350	410	8.82	72045	0.039	1069851	397	8.11	78320	0.043
H. besckei	*optix*	Hmel218003	736567	27	206.69	927	0.001	868677	22	69.91	2741	0.003
H. c. chioneus	*optix*	Hmel218003	804168	87	125.11	4209	0.002	788617	76	64.26	8194	0.003
H. c. cydnides	*optix*	Hmel218003	637588	98	63.47	7727	0.003	789494	78	55.25	8877	0.004
H. c. weymeri gustavi	*optix*	Hmel218003	624687	119	45.62	9309	0.005	624737	119	45.61	9309	0.005
H. c. weymeri weymeri	*optix*	Hmel218003	1019902	158	123.37	2741	0.002	786278	73	71.45	4733	0.003
H. c. zelinde	*optix*	Hmel218003	789014	76	64.69	8021	0.003	789414	74	64.1	8094	0.003
H. elevatus ECU	*optix*	Hmel218003	773075	146	150.85	4629	0.001	857481	39	51.17	13645	0.004

Population	Locus	Scaffold	Position	CLR	α	$2N_es$	s	Position (α_{min})	CLR (α_{min})	α_{min}	$2N_es$ (α_{min})	s (α_{min})
H. heurippa	*optix*	Hmel218003	857779	360	22.39	9401	0.009	854779	306	22.17	9495	0.009
			781223	341	31.12	6763	0.007	785474	300	27.81	7570	0.007
H. m. amaryllis	*optix*	Hmel218003	786133	182	59.66	7809	0.004	786483	180	59.45	7837	0.004
H. m. cythera	*optix*	Hmel218003	838195	342	21.03	19087	0.01	838995	251	20.98	19137	0.01
H. m. ECU	*optix*	Hmel218003	813087	130	83.53	5784	0.003	810887	125	73.15	6605	0.003
H. m. malleti COL	*optix*	Hmel218003	814383	234	38.35	13047	0.006	843034	74	30.45	16428	0.007
H. m. malleti ECU	*optix*	Hmel218003	814624	197	69.18	9467	0.003	842527	70	40.15	16312	0.006
H. m. melpomene COL	*optix*	Hmel218003	672284	72	192.34	2591	0.001	624731	32	75.58	6593	0.003
H. m. melpomene FG	*optix*	Hmel218003	649562	81	61.99	5967	0.003	648312	70	60.86	6077	0.004
H. m. melpomene PAN	*optix*	Hmel218003	855571	378	24.29	18797	0.009	841770	278	20.58	22189	0.011
H. m. meriana	*optix*	Hmel218003	801534	1085	10.59	31543	0.02	811585	656	10.4	32131	0.021
H. m. nanna NORTH	*optix*	Hmel218003	782525	191	50.12	9288	0.004	859480	133	38.08	12225	0.006
H. m. nanna SOUTH	*optix*	Hmel218003	726487	30	78.35	5942	0.003	727738	28	73.94	6296	0.003
H. m. plesseni	*optix*	Hmel218003	784931	1228	9.4	31127	0.023	647624	879	8.61	33962	0.025
			640874	1199	8.72	33528	0.024	647624	879	8.61	33962	0.025
			732328	673	16.42	17818	0.013	729378	612	16.1	18165	0.013
H. m. rosina	*optix*	Hmel218003	847982	281	20.56	16860	0.01	847682	279	20.55	16871	0.01
H. m. vicina	*optix*	Hmel218003	790899	210	19.94	24988	0.011	795400	106	19.06	26147	0.012
H. m. vulcanus	*optix*	Hmel218003	848105	324	14.4	19699	0.015	842254	206	13.65	20781	0.016
H. m. xenoclea	*optix*	Hmel218003	727532	519	14.82	24921	0.015	728633	409	14.79	24974	0.015
H. pachinus	*optix*	Hmel218003	648265	181	40.25	11029	0.005	646315	86	39.49	11240	0.006
H. t. florencia	*optix*	Hmel218003	705381	164	93.56	4193	0.002	679980	87	70.48	5566	0.003
H. t. linaresi	*optix*	Hmel218003	804236	288	33.26	9897	0.006	802686	159	32.42	10151	0.007
H. t. ssp. nov. ECU	*optix*	Hmel218003	705531	142	109.22	3486	0.002	671029	24	71.52	5324	0.003
H. t. thelxinoe	*optix*	Hmel218003	542307	51	385.24	843	0.001	788326	46	131.68	2467	0.002
H. t. timareta f. contigua	*optix*	Hmel218003	868890	98	68.01	4774	0.003	941544	88	64.05	5070	0.003
H. t. timareta f. timareta	*optix*	Hmel218003	864840	103	76.84	4131	0.003	864990	102	76.6	4144	0.003
H. t. ssp. nov. COL	*optix*	Hmel218003	597376	209	45.38	6643	0.005	615177	114	34.99	8616	0.006
