# Supplementary material for: Selective sweeps on novel and introgressed variation shape mimicry loci in a butterfly adaptive radiation
Source: PLoS Biol. 2020 Feb 6;18(2):e3000597. doi: 10.1371/journal.pbio.3000597 (PMC7029882; doi:10.1371/journal.pbio.3000597)
Supplement: S8 Table — Data are from SweepFinder2 [74,76] runs with background SFS estimated from background and colour pattern scaffolds. CLR, composite likelihood ratio; SFS, site frequency spectrum. (PDF) [file pbio.3000597.s030.pdf]

S8 Table. Position, composite likelihood-ratio statistics (CLR) and strength of selection (α, $2N_es$, and s) for the highest CLR and the smallest α value on each background scaffold (α_{min}) for the *H. melpomene*-clade. Data are from SweepFinder2 [74,76] runs with background site frequency spectrum estimated from background and colour pattern scaffolds.

Population	Scaffold	Position	CLR	α	$2N_es$	s	Position (α_{min})	CLR (α_{min})	α_{min}	$2N_es$ (α_{min})	s (α_{min})
H. besckei	Hmel204017	2202171	10	545.28	654	0.001	2198120	7	41.71	8554	0.008
H. c. chioneus	Hmel204017	2299755	19	697.25	1570	0.001	2298955	16	219.31	4992	0.002
H. c. cydnides	Hmel204017	2003132	31	135.83	9006	0.003	2003932	22	131.74	9286	0.003
H. c. weymeri gustavi	Hmel204017	2019892	36	220.12	5376	0.002	2004140	22	100.88	11731	0.004
H. c. weymeri weymeri	Hmel204017	2016795	23	228.35	4828	0.002	2053101	0	96.24	11455	0.004
H. c. zelinde	Hmel204017	2255583	10	533.35	2113	0.001	2117268	7	292.38	3854	0.001
H. elevatus Ecuador	Hmel204017	2119154	24	437.64	3926	0.001	2110153	5	84.32	20375	0.005
H. heurippa	Hmel204017	2097849	76	70.77	11544	0.005	2096449	71	67.12	12171	0.005
H. m. amaryllis	Hmel204017	1957699	13	253.74	6119	0.001	2108612	0	240.68	6451	0.002
H. m. cythera	Hmel204017	2070522	17	952.37	1287	0	2115325	15	194.2	6311	0.002
H. m. ECU	Hmel204017	2033793	16	823.66	1755	0	1957884	3	176.14	8205	0.002
H. m. malleti COL	Hmel204017	1940231	16	451.95	3123	0.001	1957983	9	195.98	7201	0.002
H. m. malleti ECU	Hmel204017	1940094	16	866.84	2008	0	2107461	0	258.44	6736	0.001
H. m. melpomene COL	Hmel204017	2070510	13	1260.27	1107	0	2054109	1	180.77	7720	0.002
H. m. melpomene FG	Hmel204017	2003188	23	140.16	5579	0.003	2001888	19	92.68	8437	0.004
H. m. melpomene PAN	Hmel204017	1958471	21	127.92	10911	0.003	1958371	21	127.07	10984	0.003
H. m. meriana	Hmel204017	1938159	32	336.68	1616	0.001	2054271	0	79.19	6871	0.004
H. m. nanna NORTH	Hmel204017	1962630	12	288.27	3705	0.001	2110887	0	163.6	6529	0.002
H. m. nanna SOUTH	Hmel204017	1987449	19	95.72	11159	0.004	1959497	11	49.03	21786	0.008
H. m. plesseni	Hmel204017	1960131	13	192.83	7408	0.002	1960181	12	190.87	7484	0.002
H. m. rosina	Hmel204017	2070277	14	634.92	1529	0.001	2117181	2	277.77	3495	0.001
H. m. vicina	Hmel204017	2028490	19	321.68	4339	0.001	1957980	9	100.14	13937	0.004
H. m. vulcanus	Hmel204017	1958592	29	106.22	8299	0.003	1960792	24	98.95	8909	0.004
H. m. xenoclea	Hmel204017	1940108	14	607.94	2121	0.001	2004412	3	329.68	3911	0.001
H. pachinus	Hmel204017	2113366	25	261.52	4568	0.001	2109366	12	61.19	19522	0.006
H. t. florencia	Hmel204017	1967332	29	163.58	6677	0.002	2065642	3	117.55	9292	0.003
H. t. linaresi	Hmel204017	1851421	22	765.3	1502	0	1859622	13	99.62	11540	0.004
H. t. ssp. nov. ECU	Hmel204017	1967446	14	227.52	5021	0.002	2089008	12	217.47	5252	0.002
H. t. thelxinoe	Hmel204017	1897232	12	1669.44	586	0	2199918	0	179.64	5442	0.002
H. t. timareta f. contigua	Hmel204017	1967333	21	219.12	4601	0.002	1894174	20	168.63	5979	0.002
H. t. timareta f. timareta	Hmel204017	2290611	19	396.19	2716	0.001	1967917	17	155.17	6934	0.002
H. t. ssp. nov. COL	Hmel204017	1967382	21	147.35	6899	0.003	2065945	15	82.77	12282	0.004
H. besckei	Hmel206006	623821	32	97.56	2909	0.003	446294	8	74.45	3812	0.003
H. c. chioneus	Hmel206006	552281	26	237.71	3714	0.001	635236	5	144.84	6096	0.002
H. c. cydnides	Hmel206006	621246	35	230.14	3875	0.001	636247	7	111.31	8011	0.002

Population	Scaffold	Position	CLR	α	$2N_es$	s	Position (α_{min})	CLR (α_{min})	α_{min}	$2N_es$ (α_{min})	s (α_{min})
H. c. weymeri gustavi	Hmel206006	551591	22	302.96	2709	0.001	635797	2	166.98	4916	0.002
H. c. weymeri weymeri	Hmel206006	358310	31	369.33	2141	0.001	581335	0	319.73	2473	0.001
H. c. zelinde	Hmel206006	625531	16	354.12	2294	0.001	625781	14	312.76	2598	0.001
H. elevatus Ecuador	Hmel206006	625339	44	206.24	6388	0.001	625089	34	193.59	6805	0.001
H. heurippa	Hmel206006	692324	168	74.34	7707	0.004	622715	85	66.49	8616	0.004
H. m. amaryllis	Hmel206006	792036	10	6846.76	189	0	624816	1	663.78	1952	0
H. m. cythera	Hmel206006	327697	24	363.9	2483	0.001	327697	24	363.9	2483	0.001
H. m. ECU	Hmel206006	754627	14	2689.83	387	0	604518	4	369.31	2819	0.001
H. m. malleti COL	Hmel206006	519734	13	860.75	1194	0	330368	3	377.11	2726	0.001
H. m. malleti ECU	Hmel206006	520036	17	670.59	2001	0	604349	1	256.42	5232	0.001
H. m. melpomene COL	Hmel206006	435316	57	323.64	3074	0.001	635627	2	182.56	5450	0.001
H. m. melpomene FG	Hmel206006	748824	25	446.97	1452	0.001	603265	10	113.82	5702	0.002
H. m. melpomene PAN	Hmel206006	550748	34	108.81	9072	0.002	550448	3	106.69	9253	0.003
H. m. meriana	Hmel206006	398903	39	187.53	2422	0.001	399353	28	176.16	2578	0.001
H. m. nanna NORTH	Hmel206006	624824	26	114.64	7274	0.002	624874	26	114.5	7282	0.002
H. m. nanna SOUTH	Hmel206006	697150	19	74.81	11146	0.004	593689	12	71	11745	0.004
H. m. plesseni	Hmel206006	542803	20	607.55	1666	0	604459	1	287.69	3519	0.001
H. m. rosina	Hmel206006	435387	21	579.17	1374	0	485890	4	372.9	2134	0.001
H. m. vicina	Hmel206006	596517	13	555.86	1790	0	635772	2	150.84	6596	0.002
H. m. vulcanus	Hmel206006	620745	37	204.59	3271	0.001	619745	33	161.41	4146	0.002
H. m. xenoclea	Hmel206006	602412	12	256.85	3909	0.001	602712	8	224.35	4475	0.001
H. pachinus	Hmel206006	602775	30	114.42	7436	0.002	603575	26	103.26	8240	0.003
H. t. florencia	Hmel206006	619768	54	158.05	5210	0.002	604217	12	115.02	7160	0.002
H. t. linaresi	Hmel206006	647619	30	294.32	2672	0.001	647569	28	294.19	2673	0.001
H. t. ssp. nov. ECU	Hmel206006	622609	54	148.24	5681	0.002	635360	12	134.25	6273	0.002
H. t. thelxinoe	Hmel206006	619714	49	200.32	3824	0.001	619764	48	199.77	3834	0.001
H. t. timareta f. contigua	Hmel206006	700365	20	884.42	865	0	736970	0	249.31	3068	0.001
H. t. timareta f. timareta	Hmel206006	739820	17	439.77	1821	0.001	503640	3	409.39	1956	0.001
H. t. ssp. nov. COL	Hmel206006	600699	80	63.47	10739	0.004	599899	77	62.48	10908	0.004
H. besckei	Hmel208051	754245	18	63.65	5209	0.006	755845	12	46.58	7119	0.008
H. c. chioneus	Hmel208051	1042680	19	158.11	7208	0.003	911321	11	91.25	12489	0.004
H. c. cydnides	Hmel208051	1043501	22	148.57	7166	0.003	1043501	22	148.57	7166	0.003
H. c. weymeri gustavi	Hmel208051	1039926	45	81.67	12625	0.005	934568	21	72.99	14126	0.005
H. c. weymeri weymeri	Hmel208051	1043638	21	341.79	2883	0.001	638197	9	132.55	7435	0.003
H. c. zelinde	Hmel208051	687356	18	424.56	2643	0.001	948375	7	136.57	8216	0.003
H. elevatus Ecuador	Hmel208051	1044514	49	97.68	16657	0.004	929249	9	90.21	18036	0.005
H. heurippa	Hmel208051	982755	352	13.28	56565	0.029	983505	284	12.93	58060	0.03
H. m. amaryllis	Hmel208051	1048965	13	801.01	2058	0.001	1045165	4	223.02	7391	0.002
H. m. cythera	Hmel208051	846537	21	442.22	2903	0.001	955998	17	136.1	9432	0.003
H. m. ECU	Hmel208051	1024299	26	94.47	14695	0.004	1024249	26	94.32	14719	0.004

Population	Scaffold	Position	CLR	α	$2N_es$	s	Position (α_{min})	CLR (α_{min})	α_{min}	$2N_es$ (α_{min})	s (α_{min})
H. m. malleti COL	Hmel208051	1049946	11	209.12	6954	0.002	1049946	11	209.12	6954	0.002
H. m. malleti ECU	Hmel208051	1050196	11	1123.99	1587	0	637708	0	359.68	4960	0.001
H. m. melpomene COL	Hmel208051	694915	15	955.82	1439	0	745370	1	186.47	7377	0.002
H. m. melpomene FG	Hmel208051	706418	10	602.4	1549	0.001	1029013	7	177.13	5267	0.002
H. m. melpomene PAN	Hmel208051	992145	20	446.35	3005	0.001	905339	6	175.09	7660	0.002
H. m. meriana	Hmel208051	1024688	41	85.45	7938	0.005	1053241	33	79.51	8530	0.005
H. m. nanna NORTH	Hmel208051	1037151	165	21.66	49737	0.018	1040451	129	20.15	53472	0.02
H. m. nanna SOUTH	Hmel208051	945416	17	35.09	30698	0.011	761453	5	23.47	45887	0.017
H. m. plesseni	Hmel208051	1100404	11	658.06	2288	0.001	1023896	3	135.07	11149	0.003
H. m. rosina	Hmel208051	903527	16	253.68	3794	0.002	956032	10	159.2	6045	0.002
H. m. vicina	Hmel208051	1069609	27	95.31	14433	0.004	1053206	20	78.74	17470	0.005
H. m. vulcanus	Hmel208051	981677	25	185	4633	0.002	986477	19	92.31	9286	0.004
H. m. xenoclea	Hmel208051	1048964	13	662.39	2014	0.001	1052915	4	178.7	7466	0.002
H. pachinus	Hmel208051	958623	33	124.22	8836	0.003	957523	33	78.89	13913	0.005
H. t. florencia	Hmel208051	1046270	132	32.25	31761	0.012	1031368	98	30.46	33621	0.013
H. t. linaresi	Hmel208051	1053301	48	72.19	14440	0.006	1053251	47	72.19	14440	0.006
H. t. ssp. nov. ECU	Hmel208051	1045877	92	35.49	29744	0.011	1046027	21	35.22	29971	0.011
H. t. thelxinoe	Hmel208051	909917	58	68.56	13188	0.006	910767	54	53.6	16868	0.007
H. t. timareta f. contigua	Hmel208051	1045354	53	51.84	17529	0.008	1045254	53	51.71	17571	0.008
H. t. timareta f. timareta	Hmel208051	1040433	50	47.32	20836	0.008	1043433	39	44.08	22366	0.009
H. t. ssp. nov. COL	Hmel208051	1024784	108	27.27	34225	0.015	1024684	108	27.26	34240	0.015
H. besckei	Hmel219003	5636365	10	382.71	653	0.001	5656167	9	234.87	1063	0.001
H. c. chioneus	Hmel219003	5569780	55	103.99	6858	0.002	5569780	55	103.99	6858	0.002
H. c. cydnides	Hmel219003	5396768	101	93.28	7151	0.003	5396768	101	93.28	7151	0.003
H. c. weymeri gustavi	Hmel219003	5538911	79	126.32	4931	0.002	5286326	25	109.02	5713	0.002
H. c. weymeri weymeri	Hmel219003	5553535	205	45.63	13006	0.005	5553235	164	45.5	13044	0.005
H. c. zelinde	Hmel219003	5570029	85	75.96	9187	0.003	5570329	42	75.18	9283	0.003
H. elevatus Ecuador	Hmel219003	5568930	68	314.01	3574	0.001	5576931	16	192.94	5816	0.001
H. heurippa	Hmel219003	5611280	98	154.57	2329	0.001	5428619	91	100.02	3599	0.002
H. m. amaryllis	Hmel219003	5552829	22	640.11	1581	0	5555129	19	441.36	2292	0.001
H. m. cythera	Hmel219003	5246608	183	46.31	16549	0.005	5245608	144	45.94	16683	0.005
H. m. ECU	Hmel219003	5576937	56	96.52	9250	0.003	5577787	52	95.49	9350	0.003
H. m. malleti COL	Hmel219003	5252414	26	323.26	2600	0.001	5254414	19	196.08	4287	0.001
H. m. malleti ECU	Hmel219003	5567800	39	469.27	2247	0.001	5576701	19	160.88	6554	0.002
H. m. melpomene COL	Hmel219003	5588298	23	1036.13	826	0	5254406	6	426.4	2008	0.001
H. m. melpomene FG	Hmel219003	5553326	40	184.69	2878	0.001	5554226	40	181.55	2928	0.001
H. m. melpomene PAN	Hmel219003	5254467	58	122.63	6589	0.002	5254417	58	122.44	6599	0.002
H. m. meriana	Hmel219003	5452642	46	241.13	1623	0.001	5244725	29	148.28	2639	0.002
H. m. nanna NORTH	Hmel219003	5576562	96	37.54	17732	0.006	5576712	95	37.52	17742	0.006
H. m. nanna SOUTH	Hmel219003	5597149	76	53.93	12342	0.004	5607500	18	45.49	14634	0.005

**Population**	**Scaffold**	**Position**	**CLR**	***α***	**$2N_es$**	***s***	**Position ($α_{min}$)**	**CLR ($α_{min}$)**	**$α_{min}$**	**$2N_es$ ($α_{min}$)**	***s* ($α_{min}$)**
H. m. plesseni	Hmel219003	5567856	29	333.57	2549	0.001	5254487	26	145.16	5858	0.002
H. m. rosina	Hmel219003	5551670	47	201.16	3144	0.001	5255329	30	145.08	4360	0.002
H. m. vicina	Hmel219003	5586901	36	297.39	2879	0.001	5551797	24	155.19	5517	0.002
H. m. vulcanus	Hmel219003	5553765	50	237.42	2287	0.001	5577818	26	212.72	2553	0.001
H. m. xenoclea	Hmel219003	5569609	25	323.88	2512	0.001	5576810	6	145.39	5597	0.002
H. pachinus	Hmel219003	5551837	78	116.07	5952	0.002	5555137	69	111.15	6215	0.002
H. t. florencia	Hmel219003	5715417	124	98.33	5559	0.002	5251087	41	48.88	11184	0.005
H. t. linaresi	Hmel219003	5610520	98	190.54	2748	0.001	5388490	69	89.17	5872	0.003
H. t. ssp. nov. ECU	Hmel219003	5715383	137	89.06	6202	0.003	5251238	26	77.57	7120	0.003
H. t. thelxinoe	Hmel219003	5611298	171	88.73	4321	0.003	5611098	168	88.41	4336	0.003
H. t. timareta f. contigua	Hmel219003	5250567	175	32.56	14290	0.007	5250067	132	32.27	14420	0.007
H. t. timareta f. timareta	Hmel219003	5551811	114	88.55	5363	0.003	5251171	38	75.12	6322	0.003
H. t. ssp. nov. COL	Hmel219003	5583888	412	21.17	21110	0.011	5575787	274	19.51	22910	0.012
