# Supplementary material for: Selective sweeps on novel and introgressed variation shape mimicry loci in a butterfly adaptive radiation
Source: PLoS Biol. 2020 Feb 6;18(2):e3000597. doi: 10.1371/journal.pbio.3000597 (PMC7029882; doi:10.1371/journal.pbio.3000597)
Supplement: S9 Table — (PDF) [file pbio.3000597.s031.pdf]

S9 Table. List of additional genes with significant colour pattern associations on the cortex scaffold from Nadeau *et al*. [30] that overlap with or are in proximity of selection signatures detected in this study.

**Hm gene IDs**	**He gene IDs**	**Putative gene name**	**Position relative to *cortex***	**Comment**	**Number ID in S17 Fig and S18 Fig**
HM00002	HERA000036	Acylpeptide hydrolase	downstream		6
HM00008	HERA000040		downstream		7
HM00012	HERA000042	*CG2519*	downstream		8
HM00019	HERA000052	*BmSuc2*	downstream		9
HM00026	HERA000077	Poly-A specific ribonuclease *parn*	upstream		14
HM00031	HERA000083		upstream		15
HM00032	HERA000084	Zinc phosphodiesterase	upstream		16
HM00033	HERA000085	Serine/threonine-protein kinase *LMTK1*	upstream	strong, consistent selection signal in *H. melpomene*	17
HM00034	HERA000086	WD repeat domain *Wdr13*	upstream		18
HM00035	HERA000087	*domeless*	upstream	truncated sequence	19/10
HM00036	HERA000061	*washout / WAS homologue 1*	upstream	strong evidence in Nadeau *et al*. 2016	20/11
HM00037	annotated as evm.TU.Herato1505.96 in *H. erato demophoon* v1	*domeless*	upstream	complete sequence, not considered in Nadeau *et al*. 2016	21/12
HM00038	HERA000062	*lethal (2) k05819 CG3054*	upstream	strong, consistent selection signal in *H. erato*	22/13
HM00052	HERA000076		upstream		23
