# Supplementary material for: Selective sweeps on novel and introgressed variation shape mimicry loci in a butterfly adaptive radiation
Source: PLoS Biol. 2020 Feb 6;18(2):e3000597. doi: 10.1371/journal.pbio.3000597 (PMC7029882; doi:10.1371/journal.pbio.3000597)
Supplement: S10 Table — (PDF) [file pbio.3000597.s032.pdf]

S10 Table. Sample information and genotyping statistics for all samples from the *Heliconius erato*-clade from Van Belleghem *et al*. [38].

SequenceID	EarthCapeID	Taxon name	Country	Latitude	Longitude	Accession
BC2115	BC2115	*Heliconius erato amalfreda*	Suriname	-4.946897	55.183386	SAMN05224103
BC2124	BC2124	*Heliconius erato amalfreda*	Suriname	-5.943486	55.186072	SAMN05224104
STRI_WOM_5779	STRI_WOM_5779	*Heliconius erato amalfreda*	Suriname	-5.940653	55.189922	SAMN05224208
STRI_WOM_5780	STRI_WOM_5780	*Heliconius erato amalfreda*	Suriname	-5.940653	55.189922	SAMN05224209
STRI_WOM_5781	STRI_WOM_5781	*Heliconius erato amalfreda*	Suriname	-4.932733	55.200803	SAMN05224210
STRI_WOM_0057	STRI_WOM_0057	*Heliconius erato chestertonii*	Colombia	3.884017	-76.589367	SAMN05224192
STRI_WOM_0058	STRI_WOM_0058	*Heliconius erato chestertonii*	Colombia	3.884017	-76.589367	SAMN05224193
STRI_WOM_0059	STRI_WOM_0059	*Heliconius erato chestertonii*	Colombia	3.884017	-76.589367	SAMN05224194
3661	CS003661	*Heliconius erato chestertonii*	Colombia	3.884017	-76.589367	SAMN05224096
3662	CS003662	*Heliconius erato chestertonii*	Colombia	3.884017	-76.589367	SAMN05224097
3663	CS003663	*Heliconius erato chestertonii*	Colombia	3.884017	-76.589367	SAMN05224098
3664	CS003664	*Heliconius erato chestertonii*	Colombia	3.884017	-76.589367	SAMN05224099
cyrbia_004	CYR004	*Heliconius erato cyrbia*	Ecuador	-3.726389	-79.836667	SAMN05224122
cyrbia_005	CYR005	*Heliconius erato cyrbia*	Ecuador	-3.726389	-79.836667	SAMN05224123
cyrbia_023	CYR023	*Heliconius erato cyrbia*	Ecuador	-3.726389	-79.836667	SAMN05224124
cyrbia_024	CYR024	*Heliconius erato cyrbia*	Ecuador	-3.726389	-79.836667	SAMN05224125
Pet_ED3	Pet_ED3	*Heliconius erato demophoon*	Panama	-9.129444	79.715278	SAMN05224182
Pet_ED4	Pet_ED4	*Heliconius erato demophoon*	Panama	-9.129444	79.715278	SAMN05224183
Pet_ED5	Pet_ED5	*Heliconius erato demophoon*	Panama	-9.129444	79.715278	SAMN05224184
Pet_ED6	Pet_ED6	*Heliconius erato demophoon*	Panama	-9.129444	79.715278	SAMN05224185
STRI_WOM_0033	STRI_WOM_0033	*Heliconius erato demophoon*	Panama	-9.1525	78.689722	SAMN05224188
STRI_WOM_0082	STRI_WOM_0082	*Heliconius erato demophoon*	Panama	-9.1525	78.689722	SAMN05224195
STRI_WOM_0087	STRI_WOM_0087	*Heliconius erato demophoon*	Panama	-9.1525	78.689722	SAMN05224196
STRIWOM1284	STRI_WOM_1284	*Heliconius erato demophoon*	Panama	-9.1525	78.689722	SAMN05224198
STRIWOM5353	STRI_WOM_5353	*Heliconius erato demophoon*	Panama	-9.1525	78.689722	SAMN05224202
STRIWOM5362	STRI_WOM_5362	*Heliconius erato demophoon*	Panama	-9.1525	78.689722	SAMN05224203
BC2563*	BC_2563	*Heliconius erato emma*	Peru	-5.29499	-78.381	SAMN08049955
BC2577*	BC_2577	*Heliconius erato emma*	Peru	-5.29499	-78.381	SAMN08049956
BC2578*	BC_2578	*Heliconius erato emma*	Peru	-5.29499	-78.381	SAMN08049957
BC2579*	BC_2579	*Heliconius erato emma*	Peru	-5.29499	-78.381	SAMN08049958
GS020redo	GS020	*Heliconius erato emma*	Peru	-6.181944	-76.247222	SAMN05224127
GS021redo	GS021	*Heliconius erato emma*	Peru	-6.181944	-76.247222	SAMN05224128
NCS_1671	NCS1671	*Heliconius erato emma*	Peru	-6.181944	-76.247222	SAMN05224154
NCS_1672	NCS1672	*Heliconius erato emma*	Peru	-6.181944	-76.247222	SAMN05224155
NCS_1673	NCS1673	*Heliconius erato emma*	Peru	-6.181944	-76.247222	SAMN05224156
NCS_1674	NCS1674	*Heliconius erato emma*	Peru	-6.181944	-76.247222	SAMN05224157
NCS_1675	NCS1675	*Heliconius erato emma*	Peru	-6.181944	-76.247222	SAMN05224158
NCS_2005	NCS2005	*Heliconius erato erato*	French Guiana	-4.638611	52.301667	SAMN05224160
NCS_2012	NCS2012	*Heliconius erato erato*	French Guiana	-4.638611	52.301667	SAMN05224161

SequenceID	EarthCapeID	Taxon name	Country	Latitude	Longitude	Accession
NCS_2020	NCS2020	*Heliconius erato erato*	French Guiana	-4.585	52.245556	SAMN05224162
NCS_2023	NCS2023	*Heliconius erato erato*	French Guiana	-4.638611	52.301667	SAMN05224163
NCS_2025	NCS2025	*Heliconius erato erato*	French Guiana	-4.585	52.245556	SAMN05224164
NCS_2556	NCS2556	*Heliconius erato erato*	French Guiana	-4.621944	52.376111	SAMN05224174
BC_3277	BC_3277	*Heliconius erato etylus*	Ecuador	-1.97786	-78.00945	SAMN05224110
BC_3278	BC_3278	*Heliconius erato etylus*	Ecuador	-1.97786	-78.00945	SAMN05224111
BC_3280	BC_3280	*Heliconius erato etylus*	Ecuador	-1.97786	-78.00945	SAMN05224112
BC_3281	BC_3281	*Heliconius erato etylus*	Ecuador	-1.97786	-78.00945	SAMN05224113
BC_3282	BC_3282	*Heliconius erato etylus*	Ecuador	-1.97786	-78.00945	SAMN05224114
BC2635*	BC_2635	*Heliconius erato favorinus*	Peru	-6.4174	-77.44329	SAMN08049959
BC2637*	BC_2637	*Heliconius erato favorinus*	Peru	-6.4174	-77.44329	SAMN08049960
BC2638*	BC_2638	*Heliconius erato favorinus*	Peru	-6.4174	-77.44329	SAMN08049961
BC2639*	BC_2639	*Heliconius erato favorinus*	Peru	-6.4174	-77.44329	SAMN08049962
GS012redo	GS012	*Heliconius erato favorinus*	Peru	-6.461389	-76.341944	SAMN05224126
NCS_0471	NCS0471	*Heliconius erato favorinus*	Peru	-6.474167	-76.010278	SAMN05224148
NCS_0473	NCS0473	*Heliconius erato favorinus*	Peru	-6.474167	-76.010278	SAMN05224149
NCS_0476	NCS0476	*Heliconius erato favorinus*	Peru	-6.474167	-76.010278	SAMN05224150
NCS_0478	NCS0478	*Heliconius erato favorinus*	Peru	-6.474167	-76.010278	SAMN05224151
NCS_0479	NCS0479	*Heliconius erato favorinus*	Peru	-6.474167	-76.010278	SAMN05224152
NCS_2554	NCS2554	*Heliconius erato favorinus*	Peru	-6.474167	-76.010278	SAMN05224172
NCS_2555	NCS2555	*Heliconius erato favorinus*	Peru	-6.474167	-76.010278	SAMN05224173
STRI_WOM_0042	STRI_WOM_0042	*Heliconius erato hydara*	Panama	-9.1525	78.689722	SAMN05224191
NCS_1179	NCS1179	*Heliconius erato hydara*	French Guiana	-4.703611	52.303611	SAMN05224153
NCS_1979	NCS1979	*Heliconius erato hydara*	French Guiana	-4.571667	52.223333	SAMN05224159
NCS_2080	NCS2080	*Heliconius erato hydara*	French Guiana	-4.607778	52.2725	SAMN05224165
NCS_2211	NCS2211	*Heliconius erato hydara*	French Guiana	-4.547222	52.170278	SAMN05224166
NCS_2217	NCS2217	*Heliconius erato hydara*	French Guiana	-4.544444	52.1525	SAMN05224167
STRI_WOM_0039	STRI_WOM_0039	*Heliconius erato hydara*	Panama	-9.1525	78.689722	SAMN05224189
STRI_WOM_0040	STRI_WOM_0040	*Heliconius erato hydara*	Panama	-9.1525	78.689722	SAMN05224190
STRI_WOM_0088	STRI_WOM_0088	*Heliconius erato hydara*	Panama	-9.1525	78.689722	SAMN05224197
STRI_WOM_5193	STRI_WOM_5193	*Heliconius erato hydara*	Panama	-9.1525	78.689722	SAMN05224200
STRI_WOM_5351	STRI_WOM_5351	*Heliconius erato hydara*	Panama	-9.1525	78.689722	SAMN05224201
BC_0411	BC0411	*Heliconius erato lativitta*	Ecuador	-1.098333	77.583889	SAMN05224101
lativitta_01	LAT01	*Heliconius erato lativitta*	Ecuador	-1.098333	77.583889	SAMN05224137
lativitta_02	LAT02	*Heliconius erato lativitta*	Ecuador	-1.098333	77.583889	SAMN05224138
lativitta_03	LAT03	*Heliconius erato lativitta*	Ecuador	-1.098333	77.583889	SAMN05224139
lativitta_04	LAT04	*Heliconius erato lativitta*	Ecuador	-0.7125	77.583889	SAMN05224140
BC_0410	BC_0410	*Heliconius erato notabilis*	Ecuador	-1.81337	-78.04507	SAMN05224100
BC_3223	BC_3223	*Heliconius erato notabilis*	Ecuador	-1.81337	-78.04507	SAMN05224105
BC_3224	BC_3224	*Heliconius erato notabilis*	Ecuador	-1.81337	-78.04507	SAMN05224106
BC_3225	BC_3225	*Heliconius erato notabilis*	Ecuador	-1.81337	-78.04507	SAMN05224107

SequenceID	EarthCapeID	Taxon name	Country	Latitude	Longitude	Accession
BC_3227	BC_3227	*Heliconius erato notabilis*	Ecuador	-1.82259	-78.04406	SAMN05224108
BC_3228	BC_3228	*Heliconius erato notabilis*	Ecuador	-1.82259	-78.04406	SAMN05224109
notabilis_01	NOT01	*Heliconius erato notabilis*	Ecuador	-1.399167	78.181111	SAMN05224178
notabilis_02	NOT02	*Heliconius erato notabilis*	Ecuador	-1.399167	78.181111	SAMN05224179
notabilis_03	NOT03	*Heliconius erato notabilis*	Ecuador	-1.399167	78.181111	SAMN05224180
notabilis_04	NOT04	*Heliconius erato notabilis*	Ecuador	-1.399167	78.181111	SAMN05224181
M_3654	CS003654	*Heliconius erato venus*	Colombia	3.5311	-76.753383	SAMN05224141
M_3655	CS003655	*Heliconius erato venus*	Colombia	3.5311	-76.753383	SAMN05224142
M_3656	CS003656	*Heliconius erato venus*	Colombia	3.5311	-76.753383	SAMN05224143
M_3657	CS003657	*Heliconius erato venus*	Colombia	3.5311	-76.753383	SAMN05224144
M_3659	CS003659	*Heliconius erato venus*	Colombia	3.5311	-76.753383	SAMN05224145
BC2565*	BC_2565	*Heliconius himera*	Peru	-5.43724	-78.4714	SAMN08049963
BC2566*	BC_2566	*Heliconius himera*	Peru	-5.43724	-78.4714	SAMN08049964
BC2567*	BC_2567	*Heliconius himera*	Peru	-5.43724	-78.4714	SAMN08049965
BC2570*	BC_2570	*Heliconius himera*	Peru	-5.43724	-78.4714	SAMN08049966
himera_001	HIM001	*Heliconius himera*	Ecuador	-4.276111	-79.195833	SAMN05224132
himera_002	HIM002	*Heliconius himera*	Ecuador	-4.276111	-79.195833	SAMN05224133
himera_003	HIM003	*Heliconius himera*	Ecuador	-4.276111	-79.195833	SAMN05224134
himera_006	HIM006	*Heliconius himera*	Ecuador	-4.276111	-79.195833	SAMN05224135
himera_030	HIM030	*Heliconius himera*	Ecuador	-4.276111	-79.195833	SAMN05224136
LM CI94-13	-	*Heliconius hermathena*	Brazil	-2.450000	-54.700000	SAMN05224129
LM CI94-14	-	*Heliconius hermathena*	Brazil	-2.450000	-54.700000	SAMN05224130
LM CI94-15	-	*Heliconius hermathena*	Brazil	-2.450000	-54.700000	SAMN05224131
