# Supplementary material for: Selective sweeps on novel and introgressed variation shape mimicry loci in a butterfly adaptive radiation
Source: PLoS Biol. 2020 Feb 6;18(2):e3000597. doi: 10.1371/journal.pbio.3000597 (PMC7029882; doi:10.1371/journal.pbio.3000597)
Supplement: S11 Table — Additional relevant peaks on scaffolds are also given. Data are from SweepFinder2 [74,76] runs with background SFS estimated from background scaffolds. CLR, composite likelihood ratio; SFS, site frequency spectrum. (PDF) [file pbio.3000597.s033.pdf]

S11 Table. Position, composite likelihood-ratio statistics (CLR) and strength of selection (α, $2N_es$, and s) for the highest CLR and the smallest α value on each colour pattern scaffold (α_{min}) for *H. erato*. Additional relevant peaks on scaffolds are also given. Data are from SweepFinder2 [74,76] runs with background site frequency spectrum estimated from background scaffolds.

Population	Locus	Scaffold	Position	CLR	α	$2N_es$	s	Position (α_{min})	CLR (α_{min})	α_{min}	$2N_es$ (α_{min})	s (α_{min})
H. e. amalfreda	*WntA*	Herato1001	4642379	561	24.28	39792	0.006	4646429	514	23.61	40923	0.006
H. e. cyrbiaN	*WntA*	Herato1001	4402129	123	50.94	8607	0.003	4402279	121	50.9	8613	0.003
H. e. demophoon	*WntA*	Herato1001	4410429	146	119.4	8119	0.001	4410429	146	119.4	8119	0.001
H. e. emma	*WntA*	Herato1001	4651630	289	52.24	19995	0.003	4649530	287	49.4	21146	0.003
H. e. erato	*WntA*	Herato1001	4642325	478	27.69	33496	0.005	4646375	456	26.84	34558	0.006
H. e. etylus	*WntA*	Herato1001	4646229	701	16.24	58092	0.009	4698580	696	10.22	92334	0.015
H. e. favorinus	*WntA*	Herato1001	4654380	250	28.24	36989	0.005	4668580	240	23.16	45096	0.007
H. e. hydaraFG	*WntA*	Herato1001	4668734	306	25.55	36301	0.006	4667884	298	25.4	36515	0.006
H. e. hydaraP	*WntA*	Herato1001	5302745	123	177.44	5464	0.001	4719235	46	87.15	11124	0.002
H. e. lativitta	*WntA*	Herato1001	4651584	363	41.07	23599	0.004	4649684	345	40.29	24056	0.004
H. e. notabilis	*WntA*	Herato1001	4648024	909	14.09	66925	0.011	4677475	763	9.88	95453	0.015
H. e. venus	*WntA*	Herato1001	4405179	208	40.16	15539	0.004	4403179	181	38.54	16193	0.004
H. e. amalfreda	*cortex*	Herato1505	2494767	1628	13	111467	0.017	2499467	1543	12.91	112233	0.017
H. e. cyrbiaN	*cortex*	Herato1505	2131207	436	6.16	106722	0.035	2130806	434	6.16	106744	0.035
H. e. demophoon	*cortex*	Herato1505	2277009	1050	13.99	103964	0.016	2267559	1039	13.93	104361	0.016
H. e. emma	*cortex*	Herato1505	2496694	1392	16.03	97743	0.014	2497894	1382	16	97946	0.014
H. e. erato	*cortex*	Herato1505	2493655	1483	14.43	96430	0.016	2490155	1455	14.38	96762	0.016
H. e. etylus	*cortex*	Herato1505	2494192	1312	15.07	93917	0.015	2497592	1271	15.01	94276	0.015
H. e. favorinus	*cortex*	Herato1505	2495987	2558	8.04	194973	0.028	2494987	2552	8.04	195006	0.028
H. e. hydaraFG	*cortex*	Herato1505	2490958	1552	12.45	111745	0.018	2488308	1531	12.39	112300	0.018
H. e. hydaraP	*cortex*	Herato1505	2985526	289	29.8	48802	0.008	2999826	56	21.55	67476	0.01
H. e. lativitta	*cortex*	Herato1505	2491864	1252	15.96	91073	0.014	2489914	1239	15.9	91434	0.014
H. e. notabilis	*cortex*	Herato1505	2497650	1387	15.2	93112	0.015	2501850	1354	14.76	95869	0.015
			1963287	472	49.76	28438	0.005	2065039	155	37.53	37703	0.006
H. e. venus	*cortex*	Herato1505	2069952	874	5.74	162992	0.038	2130504	791	4.82	194354	0.046
H. e. amalfreda	*optix*	Herato1801	1304730	997	6.66	172295	0.027	1304030	995	6.66	172319	0.027
			1375134	936	11.31	101441	0.016	1356333	509	10.52	109048	0.017
H. e. cyrbiaN	*optix*	Herato1801	921205	174	37.21	13990	0.005	1302732	68	23.15	22491	0.007
H. e. demophoon	*optix*	Herato1801	938505	66	340.1	3385	0.001	1296221	3	74.7	15411	0.002
H. e. emma	*optix*	Herato1801	1305984	449	13.21	93912	0.014	1303583	418	13.11	94614	0.014
H. e. erato	*optix*	Herato1801	1380478	1005	11.02	99959	0.016	1305775	913	7.61	144693	0.023
			1303875	922	7.62	144522	0.023	1305775	913	7.61	144693	0.023
H. e. etylus	*optix*	Herato1801	1304785	423	10.67	104966	0.017	1300385	408	10.58	105893	0.017
			1407742	405	21.46	52202	0.008	1384040	390	20.89	53626	0.009
H. e. favorinus	*optix*	Herato1801	1381433	285	32.38	38310	0.006	1303679	182	24.3	51053	0.007

Population	Locus	Scaffold	Position	CLR	α	$2N_es$	s	Position (α_{min})	CLR (α_{min})	α_{min}	$2N_es$ (α_{min})	s (α_{min})
			1304379	184	24.35	50937	0.007	1303679	182	24.3	51053	0.007
H. e. hydaraFG	*optix*	Herato1801	1426489	4187	3.98	276447	0.045	1431689	4152	3.98	276715	0.045
H. e. hydaraP	*optix*	Herato1801	1644203	78	234.88	4901	0.001	1300682	46	26.83	42912	0.007
H. e. lativitta	*optix*	Herato1801	1381486	480	17.87	64400	0.01	1303132	470	9.42	122169	0.019
			1304582	478	9.44	121980	0.019	1303132	470	9.42	122169	0.019
H. e. notabilis	*optix*	Herato1801	1294528	4690	3.03	370210	0.059	1305279	4604	2.99	374119	0.06
H. e. venus	*optix*	Herato1801	921654	185	24.81	29867	0.007	900003	79	10.97	67582	0.016
