# Supplementary material for: Selective sweeps on novel and introgressed variation shape mimicry loci in a butterfly adaptive radiation
Source: PLoS Biol. 2020 Feb 6;18(2):e3000597. doi: 10.1371/journal.pbio.3000597 (PMC7029882; doi:10.1371/journal.pbio.3000597)
Supplement: S12 Table — Data are from SweepFinder2 [74,76] runs with background SFS estimated from background scaffolds. CLR, composite likelihood ratio; SFS, site frequency spectrum (PDF) [file pbio.3000597.s034.pdf]

S12 Table. Position, composite likelihood-ratio statistics (CLR) and strength of selection (α, $2N_es$, and s) for the highest CLR and the smallest α value on each background scaffold (α_{min}) for *H. erato*. Data are from SweepFinder2 [74,76] runs with background site frequency spectrum estimated from background scaffolds.

Population	Scaffold	Position	CLR	α	$2N_es$	s	Position (α_{min})	CLR (α_{min})	α_{min}	$2N_es$ (α_{min})	s (α_{min})
H. e. amalfreda	Herato0411	4991530	21	1207.76	1158	0	4298985	13	51.96	26924	0.004
H. e. cyrbiaN	Herato0411	4206446	18	1408.09	451	0	4303500	3	72.97	8699	0.003
H. e. demophoon	Herato0411	4306907	14	64.78	21670	0.003	4302807	8	59.06	23769	0.004
H. e. emma	Herato0411	4938642	16	1762.73	858	0	4303620	4	58.4	25900	0.004
H. e. erato	Herato0411	4346808	21	163.79	8199	0.001	4301157	1	56.05	23961	0.004
H. e. etylus	Herato0411	4938611	20	737.55	1852	0	4300691	12	51.04	26762	0.004
H. e. favorinus	Herato0411	4985282	12	1269.09	1192	0	4346208	0	315.84	4789	0.001
H. e. hydaraFG	Herato0411	4303406	28	48.89	27472	0.004	4301606	26	48.17	27877	0.005
H. e. hydaraP	Herato0411	4996536	19	942.33	1490	0	4298358	7	50.07	28036	0.004
H. e. lativitta	Herato0411	4276250	14	4479.58	313	0	4301551	1	60.29	23277	0.004
H. e. notabilis	Herato0411	4276241	15	4491.75	304	0	4299742	10	43.57	31350	0.005
H. e. venus	Herato0411	4892869	14	1623.69	556	0	4296935	1	190.87	4734	0.001
H. e. amalfreda	Herato0601	1736795	36	245.53	4550	0.001	831240	3	234.12	4771	0.001
H. e. cyrbiaN	Herato0601	1370053	68	122.11	4151	0.001	1366802	65	100.36	5051	0.002
H. e. demophoon	Herato0601	1517280	30	516.29	2171	0	1517330	29	508.59	2204	0
H. e. emma	Herato0601	1764992	24	613.16	1970	0	1354931	22	300.93	4014	0.001
H. e. erato	Herato0601	1313021	23	945.95	1134	0	1354974	11	346.54	3095	0.001
H. e. etylus	Herato0601	1764630	42	225.83	4830	0.001	1763930	34	213.31	5113	0.001
H. e. favorinus	Herato0601	1398537	32	1419.11	851	0	1355285	13	294.82	4097	0.001
H. e. hydaraFG	Herato0601	1650955	64	382.41	2804	0	831954	2	246.67	4348	0.001
H. e. hydaraP	Herato0601	1398435	30	1031.75	1086	0	1763700	9	447.92	2503	0
H. e. lativitta	Herato0601	1651545	35	1311.02	855	0	831454	2	293.39	3820	0.001
H. e. notabilis	Herato0601	1495484	22	2175.42	501	0	831140	0	429.41	2540	0
H. e. venus	Herato0601	1370059	95	82.5	8746	0.002	1370259	91	82.14	8784	0.002
H. e. amalfreda	Herato0821	2893797	82	148.54	8876	0.001	2896497	55	117.8	11192	0.002
H. e. cyrbiaN	Herato0821	2736672	45	184.13	3249	0.001	2972682	24	99.09	6038	0.002
H. e. demophoon	Herato0821	2900543	31	703.49	1881	0	2972045	12	317.23	4170	0.001
H. e. emma	Herato0821	2893838	50	477.6	2985	0	2889088	12	199.64	7140	0.001
H. e. erato	Herato0821	2887040	32	495.24	2556	0	2889040	11	182.64	6930	0.001
H. e. etylus	Herato0821	2972094	13	242.72	5303	0.001	2971894	12	240.33	5356	0.001
H. e. favorinus	Herato0821	2885842	26	476.22	2993	0	2889092	13	199.31	7152	0.001
H. e. hydaraFG	Herato0821	2015021	19	1657.86	763	0	2499406	3	399.99	3164	0.001
H. e. hydaraP	Herato0821	2738537	32	331.44	3991	0.001	2897492	10	247.83	5338	0.001
H.e. lativitta	Herato0821	2896842	35	184.24	7179	0.001	2896542	33	182.13	7262	0.001

**Population**	**Scaffold**	**Position**	**CLR**	**α**	**$2N_es$**	**s**	**Position (α_{min})**	**CLR (α_{min})**	**α_{min}**	**$2N_es$ (α_{min})**	**s (α_{min})**
H. e. notabilis	Herato0821	2271120	10	1398.73	920	0	2889095	0	402.25	3200	0.001
H. e. venus	Herato0821	2735284	36	249.95	3407	0.001	2735434	35	247.62	3439	0.001
H. e. amalfreda	Herato1901	3171719	44	406.91	2473	0	2646389	4	113.64	8856	0.001
H. e. cyrbiaN	Herato1901	2846944	60	193.71	2358	0.001	2646632	30	68.6	6657	0.002
H. e. demophoon	Herato1901	3396907	196	34.93	28911	0.004	3395457	187	34.29	29448	0.005
H. e. emma	Herato1901	3171790	68	227.96	4773	0.001	3438258	11	86.67	12554	0.002
H. e. erato	Herato1901	3171726	50	489.04	1976	0	2646607	6	129.18	7479	0.001
H. e. etylus	Herato1901	3171722	84	234.95	4182	0.001	3366129	51	103.61	9484	0.002
H. e. favorinus	Herato1901	3171441	51	333.83	3259	0	2647807	5	153.49	7089	0.001
H. e. hydaraFG	Herato1901	3171813	55	409.96	2357	0	3367468	26	88.6	10905	0.002
H. e. hydaraP	Herato1901	3396556	234	25.21	40051	0.006	3396806	234	25.21	40065	0.006
H. e. lativitta	Herato1901	3171698	54	328.19	3076	0	3366606	27	128.31	7868	0.001
H. e. notabilis	Herato1901	3171700	61	312.25	3147	0.001	3396758	9	161.06	6101	0.001
H. e. venus	Herato1901	3337404	95	106.9	6081	0.001	2991894	67	44.62	14567	0.003
