# Supplementary material for: Selective sweeps on novel and introgressed variation shape mimicry loci in a butterfly adaptive radiation
Source: PLoS Biol. 2020 Feb 6;18(2):e3000597. doi: 10.1371/journal.pbio.3000597 (PMC7029882; doi:10.1371/journal.pbio.3000597)
Supplement: S13 Table — Additional relevant peaks on scaffolds are also given. Data are from SweepFinder2 [74,76] runs with background SFS estimated from background and colour pattern scaffolds. CLR, composite likelihood ratio; SFS, site frequency spectrum. (PDF) [file pbio.3000597.s035.pdf]

S13 Table. Position, composite likelihood-ratio statistics (CLR) and strength of selection (*α*, 2*N_es*, and *s*) for the highest CLR and the smallest *α* value on each colour pattern scaffold (*$α_{min}$*) for *H. erato*. Additional relevant peaks on scaffolds are also given. Data are from SweepFinder2 [74,76] runs with background site frequency spectrum estimated from background and colour pattern scaffolds.

Population	Locus	Scaffold	Position	CLR	*α*	2*N_es*	*s*	Position (*$α_{min}$*)	CLR (*$α_{min}$*)	*$α_{min}$*	2*N_es* (αmin)	*s* (*$α_{min}$*)
H. e. amalfreda	*WntA*	Herato1001	4642279	434	29.58	32666	0.005	4644829	411	28.92	33408	0.005
H. e. cyrbiaN	*WntA*	Herato1001	5466098	69	155.66	2816	0.001	4402079	57	72.47	6050	0.002
H. e. demophoon	*WntA*	Herato1001	4410429	99	141.61	6846	0.001	4410479	98	141.6	6847	0.001
H. e. emma	*WntA*	Herato1001	4649730	216	56.76	18404	0.003	4649680	216	56.75	18406	0.003
H. e. erato	*WntA*	Herato1001	4642175	361	33.42	27754	0.004	4644875	332	32.74	28331	0.005
H. e. etylus	*WntA*	Herato1001	4645879	511	20.92	45087	0.007	4685579	313	13.76	68569	0.011
H. e. favorinus	*WntA*	Herato1001	5465699	150	122.65	8517	0.001	4649330	93	48.77	21419	0.003
H. e. hydaraFG	*WntA*	Herato1001	4668834	166	41.74	22222	0.004	4668134	160	41.18	22522	0.004
H. e. hydaraP	*WntA*	Herato1001	5302745	92	215.84	4492	0.001	4719285	21	120.98	8013	0.001
H. e. lativitta	*WntA*	Herato1001	4651634	293	46.52	20833	0.003	4649634	275	45.42	21339	0.003
H. e. notabilis	*WntA*	Herato1001	4642524	554	22.37	42160	0.007	4685525	249	15.91	59304	0.009
H. e. venus	*WntA*	Herato1001	4405179	119	57.41	10869	0.003	4403229	94	53.93	11571	0.003
H. e. amalfreda	*cortex*	Herato1505	2494717	1306	14.75	98269	0.015	2499417	1224	14.63	99040	0.015
H. e. cyrbiaN	*cortex*	Herato1505	2453566	167	47.95	13714	0.004	2131107	132	16.5	39848	0.013
H. e. demophoon	*cortex*	Herato1505	2277009	723	20.21	71962	0.011	2267559	704	19.84	73287	0.011
H. e. emma	*cortex*	Herato1505	2496694	1120	18.13	86428	0.013	2497944	1110	18.07	86717	0.013
H. e. erato	*cortex*	Herato1505	2493705	1151	16.7	83292	0.013	2491005	1134	16.67	83477	0.014
H. e. etylus	*cortex*	Herato1505	2494192	1016	17.5	80873	0.013	2497192	986	17.43	81167	0.013
H. e. favorinus	*cortex*	Herato1505	2496137	1734	9.92	157969	0.023	2494937	1725	9.91	158072	0.023
H. e. hydaraFG	*cortex*	Herato1505	2493808	1107	15.64	88979	0.014	2488308	1081	15.31	90896	0.015
H. e. hydaraP	*cortex*	Herato1505	2985526	205	35.05	41484	0.006	2985526	205	35.05	41484	0.006
H. e. lativitta	*cortex*	Herato1505	2491914	1030	17.92	81126	0.013	2490014	1018	17.85	81455	0.013
H. e. notabilis	*cortex*	Herato1505	2497600	909	18.52	76386	0.012	2501750	879	17.92	78973	0.013
			1963287	311	60.24	23489	0.004	1962437	301	59.92	23612	0.004
H. e. venus	*cortex*	Herato1505	2069802	429	9.57	97818	0.023	2130154	252	8.34	112198	0.026
H. e. amalfreda	*optix*	Herato1801	1375134	663	15.79	72638	0.011	1303280	544	8.22	139501	0.022
			1304780	552	8.23	139362	0.022	1303280	544	8.22	139501	0.022
H. e. cyrbiaN	*optix*	Herato1801	916554	102	74.8	6960	0.002	921655	96	49.41	10537	0.003
H. e. demophoon	*optix*	Herato1801	938505	47	408.01	2822	0	1293521	4	117.67	9784	0.002
H. e. emma	*optix*	Herato1801	1381539	287	39.53	31378	0.005	1303933	216	16.34	75896	0.011
H. e. erato	*optix*	Herato1801	1380328	672	14.57	75574	0.012	1305325	467	9.61	114604	0.019
			1303775	472	9.62	114444	0.019	1305325	467	9.61	114604	0.019
H. e. etylus	*optix*	Herato1801	1382440	261	32.26	34721	0.006	1300235	172	15.3	73220	0.012
			1305285	193	15.56	71968	0.011	1300235	172	15.3	73220	0.012
H. e. favorinus	*optix*	Herato1801	1381333	142	44.88	27639	0.004	1304029	55	37.14	33398	0.005

Population	Locus	Scaffold	Position	CLR	α	$2N_es$	s	Position (α_{min})	CLR (α_{min})	α_{min}	$2N_eS$ (αmin)	s (α_{min})
			1251876	88	139.79	8874	0.001	1304029	55	37.14	33398	0.005
H. e. hydaraFG	*optix*	Herato1801	1425389	2890	4.95	222666	0.036	1429539	2881	4.94	223002	0.036
H. e. hydaraP	*optix*	Herato1801	1643303	55	356.23	3232	0.001	1305282	5	70.22	16395	0.003
H. e. lativitta	*optix*	Herato1801	1381286	350	24.23	47504	0.007	1302982	247	12.27	93822	0.015
			1304732	258	12.31	93489	0.015	1302982	247	12.27	93822	0.015
H. e. notabilis	*optix*	Herato1801	1293428	2938	3.74	299762	0.048	1305629	2836	3.67	305008	0.049
H. e. venus	*optix*	Herato1801	1067013	94	89.46	8283	0.002	1300277	9	30.24	24506	0.006
