# Supplementary material for: Selective sweeps on novel and introgressed variation shape mimicry loci in a butterfly adaptive radiation
Source: PLoS Biol. 2020 Feb 6;18(2):e3000597. doi: 10.1371/journal.pbio.3000597 (PMC7029882; doi:10.1371/journal.pbio.3000597)
Supplement: S14 Table — Data are from SweepFinder2 [74,76] runs with background SFS estimated from background and colour pattern scaffolds. CLR, composite likelihood ratio; SFS, site frequency spectrum. (PDF) [file pbio.3000597.s036.pdf]

S14 Table. Position, composite likelihood-ratio statistics (CLR) and strength of selection (α, $2N_es$, and s) for the highest CLR and the smallest α value on each background scaffold (α_{min}) for *H. erato*. Data are from SweepFinder2 [74,76] runs with background site frequency spectrum estimated from background and colour pattern scaffolds.

Population	Scaffold	Position	CLR	α	$2N_es$	s	Position (α_{min})	CLR (α_{min})	α_{min}	$2N_es$ (α_{min})	s (α_{min})
H. e. amalfreda	Herato0411	4991530	16	1371.79	1020	0	4300335	2	69.4	20157	0.003
H. e. cyrbiaN	Herato0411	4206446	13	1604.88	396	0	4308951	0	188.29	3371	0.001
H. e. demophoon	Herato0411	4990346	8	5043.62	278	0	4251454	3	444	3162	0
H. e. emma	Herato0411	4938642	13	2186.33	692	0	4305120	0	186.18	8124	0.001
H. e. erato	Herato0411	4346608	11	191.65	7007	0.001	4303307	1	180.59	7437	0.001
H. e. etylus	Herato0411	4938611	14	1015.4	1345	0	4305191	3	81.76	16705	0.003
H. e. favorinus	Herato0411	4985282	8	1409.71	1073	0	4862378	1	600.3	2520	0
H. e. hydaraFG	Herato0411	4326307	14	2276.13	590	0	4302656	2	66.65	20150	0.003
H. e. hydaraP	Herato0411	4271057	14	1313.24	1069	0	4307208	2	162.05	8662	0.001
H. e. lativitta	Herato0411	4276250	12	5194.78	270	0	4306401	1	183.19	7661	0.001
H. e. notabilis	Herato0411	4202989	11	8102.94	169	0	4311093	0	402.78	3391	0.001
H. e. venus	Herato0411	4892869	11	1942.13	465	0	4766762	5	240.52	3757	0.001
H. e. amalfreda	Herato0601	1398524	24	1608.56	694	0	1736845	18	302.21	3696	0.001
H. e. cyrbiaN	Herato0601	1370053	39	178.88	2834	0.001	1366752	28	158.91	3190	0.001
H. e. demophoon	Herato0601	1517230	21	688.49	1628	0	1517330	20	668.58	1677	0
H. e. emma	Herato0601	1398482	18	3321.79	364	0	1354881	15	334.98	3606	0.001
H. e. erato	Herato0601	1313021	19	1029.58	1042	0	1354924	6	413.26	2595	0
H. e. etylus	Herato0601	1764630	28	289.3	3770	0.001	1763880	17	259.31	4206	0.001
H. e. favorinus	Herato0601	1398537	25	1625.82	743	0	1355285	2	367.87	3283	0
H. e. hydaraFG	Herato0601	1650955	46	472.99	2267	0	1650005	32	357.85	2997	0
H. e. hydaraP	Herato0601	1398435	23	1204.59	931	0	1763450	9	575.82	1947	0
H. e. lativitta	Herato0601	1651545	30	1450.78	772	0	831854	1	364.85	3072	0
H. e. notabilis	Herato0601	1495484	16	2463.33	443	0	1353925	2	852.72	1279	0
H. e. venus	Herato0601	1370059	49	117.58	6137	0.001	1370159	48	117.29	6152	0.001
H. e. amalfreda	Herato0821	2893797	62	208.41	6326	0.001	2896597	34	139.75	9434	0.001
H. e. cyrbiaN	Herato0821	2736672	28	220.18	2717	0.001	2736722	28	220.07	2718	0.001
H. e. demophoon	Herato0821	2900543	22	804.48	1644	0	2972095	4	390.28	3390	0.001
H. e. emma	Herato0821	2893838	43	514.73	2769	0	2889038	2	236.65	6023	0.001
H. e. erato	Herato0821	2887040	28	567.55	2230	0	2896540	8	230.79	5484	0.001
H. e. etylus	Herato0821	2893790	9	2444.68	527	0	2971794	2	336.69	3823	0.001
H. e. favorinus	Herato0821	2885842	19	589.22	2419	0	2888292	6	287.11	4965	0.001
H. e. hydaraFG	Herato0821	2015021	14	1921.28	659	0	2499056	0	634.99	1993	0
H. e. hydaraP	Herato0821	2738537	20	508.65	2601	0	2736737	11	384.27	3443	0.001
H. e. lativitta	Herato0821	2896842	24	207.54	6373	0.001	2896592	23	205.36	6440	0.001

Population	Scaffold	Position	CLR	α	$2N_es$	s	Position (α_{min})	CLR (α_{min})	α_{min}	$2N_es$ (α_{min})	s (α_{min})
H. e. notabilis	Herato0821	2115614	7	3396.92	379	0	2888345	1	734.68	1752	0
H. e. venus	Herato0821	2735284	23	289.64	2940	0.001	2735434	22	287.56	2961	0.001
H. e. amalfreda	Herato1901	3171719	35	466.22	2159	0	2648039	2	171.54	5867	0.001
H. e. cyrbiaN	Herato1901	2846944	38	231.72	1971	0.001	2647282	9	86.68	5268	0.002
H. e. demophoon	Herato1901	3397207	126	42.94	23520	0.004	3395757	119	41.88	24111	0.004
H. e. emma	Herato1901	3171790	56	338.86	3211	0	3437758	3	98.34	11064	0.002
H. e. erato	Herato1901	3171726	42	539.69	1790	0	2647507	2	176.06	5488	0.001
H. e. etylus	Herato1901	3171722	65	268.26	3663	0.001	3366479	30	132.22	7431	0.001
H. e. favorinus	Herato1901	3172741	33	942.19	1155	0	3366954	16	259.96	4186	0.001
H. e. hydaraFG	Herato1901	3171813	39	486.51	1986	0	3366818	3	153.76	6283	0.001
H. e. hydaraP	Herato1901	3396656	155	31.3	32265	0.005	3396656	155	31.3	32265	0.005
H. e. lativitta	Herato1901	3171698	45	359.63	2807	0	3366706	15	153.9	6560	0.001
H. e. notabilis	Herato1901	3171700	41	364.14	2698	0	3366257	12	302.85	3245	0.001
H. e. venus	Herato1901	3337404	62	143.11	4542	0.001	2646034	16	72.63	8950	0.002
