# Supplementary material for: Selective sweeps on novel and introgressed variation shape mimicry loci in a butterfly adaptive radiation
Source: PLoS Biol. 2020 Feb 6;18(2):e3000597. doi: 10.1371/journal.pbio.3000597 (PMC7029882; doi:10.1371/journal.pbio.3000597)
Supplement: S15 Table — (PDF) [file pbio.3000597.s037.pdf]

S15 Table. Per-population and per-scaffold summary statistics estimates and standard deviation for colour pattern scaffolds in the *H. melpomene* - clade.

	**Hmel201011:2476100-2790900**						**Hmel210004:1378000-2198000**					
	pi	**sd**	**Taj*D***	**sd**	***ZnS***	**sd**	***pi***	**sd**	**Taj*D***	**sd**	***ZnS***	**sd**
H. m. aglaope	0.0237	0.0112	-0.2430	0.5407	0.2548	0.0632	0.0246	0.0108	-0.2300	0.5516	0.2653	0.0687
H. m. amaryllis	0.0194	0.0081	-1.2187	0.4871	0.0437	0.0142	0.0201	0.0079	-1.1036	0.5898	0.0501	0.0235
H. m. malleti (Colombia)	0.0209	0.0088	-0.6575	0.5362	0.0737	0.0189	0.0211	0.0088	-0.6242	0.6388	0.0935	0.0580
H. m. malleti (Ecuador)	0.0202	0.0082	-1.0410	0.5080	0.0525	0.0134	0.0204	0.0082	-0.9652	0.5597	0.0594	0.0249
H. m. plesseni	0.0207	0.0098	-0.3558	0.5799	0.1218	0.0330	0.0194	0.0100	-0.1984	0.8663	0.1685	0.0969
H. m. xenoclea	0.0186	0.0078	-0.7164	0.5310	0.1144	0.0258	0.0165	0.0078	-0.5156	0.6647	0.1408	0.0548
H. m. meriana	0.0184	0.0083	0.3607	0.7072	0.2105	0.0574	0.0175	0.0075	0.0937	0.7168	0.2303	0.0769
H. m. melpomene (Colombia)	0.0213	0.0103	-0.4307	0.6500	0.1271	0.0781	0.0223	0.0091	-0.3829	0.6938	0.1226	0.0544
H. m. melpomene (French Guyana)	0.0202	0.0078	-0.5488	0.6263	0.2452	0.1135	0.0194	0.0076	-0.3142	0.6874	0.2489	0.1306
H. m. nanna (North)	0.0171	0.0087	-0.3838	0.6519	0.1782	0.0581	0.0169	0.0084	-0.3289	0.7199	0.1977	0.0840
H. m. nanna (South)	0.0055	0.0051	-0.3484	1.0160	0.2932	0.1624	0.0053	0.0044	-0.2013	0.8841	0.2712	0.1347
H. c. weymeri gustavi	0.0162	0.0082	0.1179	0.6859	0.1629	0.0512	0.0185	0.0087	0.1603	0.7178	0.1729	0.0604
H. c. weymeri weymeri	0.0154	0.0086	0.3383	0.7781	0.1912	0.0643	0.0190	0.0090	0.0311	0.7615	0.1708	0.0595
H. c. cydnides	0.0166	0.0084	0.1990	0.7090	0.1758	0.0637	0.0196	0.0088	0.0597	0.6373	0.1538	0.0524
H. melpomene (Panama)	0.0209	0.0095	-0.0763	0.7015	0.2170	0.0847	0.0210	0.0103	-0.1879	0.7320	0.2131	0.0872
H. m. rosina	0.0197	0.0103	-0.1025	0.7957	0.2038	0.1024	0.0195	0.0095	-0.0016	0.7478	0.2085	0.0981
H. m. vulcanus	0.0205	0.0102	0.2712	0.7555	0.2141	0.0942	0.0191	0.0090	0.3304	0.7695	0.2291	0.1085
H. m. cythera	0.0194	0.0096	0.1306	0.7037	0.2062	0.0935	0.0205	0.0093	0.2177	0.7027	0.1974	0.0888
H. m. ecuadorensis	0.0221	0.0098	-0.3545	0.5477	0.1302	0.0318	0.0213	0.0095	-0.1659	0.7492	0.1618	0.0744
H. c. chioneus	0.0185	0.0091	-0.5978	0.5379	0.1096	0.0304	0.0203	0.0086	-0.4393	0.5659	0.1105	0.0308
H. c. zelinde	0.0195	0.0099	-0.5098	0.5393	0.1234	0.0389	0.0215	0.0089	-0.2662	0.5521	0.1286	0.0347
H. pachinus	0.0151	0.0080	-0.4668	0.7118	0.1610	0.0649	0.0171	0.0074	-0.2267	0.6766	0.1616	0.0632
H. t. thelxinoe	0.0170	0.0084	0.0468	0.8196	0.1089	0.0555	0.0152	0.0081	0.0816	1.0467	0.1564	0.0947
H. t. contigua	0.0163	0.0081	-0.0996	0.8248	0.1647	0.0664	0.0135	0.0077	-0.0196	0.8992	0.1778	0.0694
H. t. timareta	0.0176	0.0087	-0.0977	0.6831	0.1618	0.0858	0.0143	0.0081	-0.1213	0.8791	0.1699	0.0685
H. t. spp (Colombia)	0.0153	0.0094	0.2284	0.8364	0.2849	0.1291	0.0160	0.0088	0.0306	1.0244	0.2876	0.1301
H. t. spp (Ecuador)	0.0171	0.0087	-0.2111	0.7647	0.0978	0.0343	0.0144	0.0082	-0.3840	1.0039	0.1277	0.0802
H. heurippa	0.0126	0.0085	0.4203	1.2504	0.2248	0.1090	0.0129	0.0085	0.4156	1.2946	0.2182	0.1081
H. t. florencia	0.0177	0.0095	-0.1433	0.9345	0.1331	0.0776	0.0169	0.0088	-0.2396	0.9658	0.1556	0.0799
H. t. linaresi	0.0168	0.0093	0.1272	0.9198	0.1363	0.0595	0.0169	0.0087	0.0681	0.8861	0.1484	0.0601

	**Hmel215006:560500-1931800**						**Hmel218003:509700-1194700**					
	pi	**sd**	**Taj*D***	**sd**	***ZnS***	**sd**	***pi***	**sd**	**Taj*D***	**sd**	***ZnS***	**sd**
H. m. aglaope	0.0209	0.0107	-0.3005	0.6007	0.2713	0.0701	0.0143	0.0082	-0.2993	0.6533	0.2700	0.082
H. m. amaryllis	0.0128	0.0081	-0.9440	0.8052	0.1021	0.0623	0.0108	0.0055	-0.8952	0.7402	0.0919	0.05
H. m. malleti (Colombia)	0.0181	0.0091	-0.5801	0.6760	0.0980	0.0475	0.0131	0.0069	-0.6757	0.6733	0.0928	0.043
H. m. malleti (Ecuador)	0.0181	0.0085	-0.8546	0.6658	0.0671	0.0280	0.0138	0.0068	-1.0729	0.5761	0.0697	0.042
H. m. plesseni	0.0152	0.0106	0.0345	1.1809	0.2232	0.1484	0.0101	0.0073	0.2854	1.1516	0.2597	0.13
H. m. xenoclea	0.0151	0.0087	-0.4885	0.7533	0.1595	0.0722	0.0107	0.0062	-0.3227	0.8610	0.1660	0.07
H. m. meriana	0.0171	0.0082	0.1679	0.7190	0.2297	0.0897	0.0119	0.0069	-0.0903	1.0294	0.2765	0.123
H. m. melpomene (Colombia)	0.0168	0.0088	-0.2741	0.7884	0.1510	0.0789	0.0130	0.0068	-0.5739	0.7169	0.1410	0.064
H. m. melpomene (French Guyana)	0.0185	0.0082	-0.2221	0.8425	0.3023	0.1678	0.0152	0.0064	-0.1333	0.8626	0.3413	0.168
H. m. nanna (North)	0.0129	0.0086	-0.5347	0.8882	0.2043	0.0978	0.0114	0.0059	-0.5898	0.6929	0.1853	0.07
H. m. nanna (South)	0.0047	0.0045	-0.3654	0.8474	0.2673	0.1495	0.0042	0.0032	-0.2261	0.8288	0.2529	0.127
H. c. weymeri gustavi	0.0145	0.0079	0.2759	0.8097	0.1977	0.0881	0.0132	0.0066	-0.0970	0.6632	0.1660	0.053
H. c. weymeri weymeri	0.0135	0.0081	0.2823	1.0611	0.2242	0.1086	0.0116	0.0064	0.3349	0.9691	0.2094	0.083
H. c. cydnides	0.0159	0.0084	0.0714	0.7108	0.1645	0.0703	0.0139	0.0068	-0.4011	0.6238	0.1401	0.044
H. melpomene (Panama)	0.0171	0.0094	-0.1877	0.7563	0.2204	0.0994	0.0127	0.0072	-0.3645	0.8003	0.2282	0.094
H. m. rosina	0.0150	0.0091	-0.0558	0.8807	0.2190	0.1100	0.0115	0.0069	-0.1874	0.8961	0.2159	0.108
H. m. vulcanus	0.0157	0.0091	0.2159	0.9332	0.2602	0.1476	0.0100	0.0069	-0.0581	0.8886	0.2754	0.141
H. m. cythera	0.0146	0.0092	0.2169	0.9775	0.2513	0.1353	0.0117	0.0073	-0.2932	0.8212	0.2223	0.114

	Hmel215006:560500-1931800						Hmel218003:509700-1194700					
	pi	**sd**	**Taj*D***	**sd**	***ZnS***	**sd**	***pi***	**sd**	**Taj*D***	**sd**	***ZnS***	**sd**
H. m. ecuadorensis	0.0183	0.0096	-0.1196	0.8082	0.1697	0.0737	0.0146	0.0071	-0.0849	0.7286	0.1755	0.061
H. c. chioneus	0.0171	0.0086	-0.6257	0.6101	0.1146	0.0499	0.0146	0.0069	-0.8068	0.5503	0.1116	0.033
H. c. zelinde	0.0178	0.0089	-0.5417	0.6121	0.1282	0.0465	0.0149	0.0069	-0.5656	0.5760	0.1320	0.041
H. pachinus	0.0137	0.0075	-0.3998	0.7473	0.1706	0.0631	0.0126	0.0066	-0.4421	0.7327	0.1569	0.062
H. t. thelxinoe	0.0162	0.0075	0.6088	0.8863	0.1568	0.0857	0.0119	0.0061	0.3751	0.9868	0.1417	0.08
H. t. contigua	0.0137	0.0082	0.0147	0.9471	0.1884	0.0792	0.0105	0.0060	0.1253	0.9594	0.1889	0.085
H. t. timareta	0.0134	0.0081	-0.0452	0.9425	0.1901	0.0861	0.0102	0.0060	0.0908	0.9430	0.1959	0.089
H. t. spp (Colombia)	0.0136	0.0095	0.0859	0.9873	0.2716	0.1223	0.0097	0.0067	-0.1169	1.1142	0.2780	0.124
H. t. spp (Ecuador)	0.0137	0.0084	-0.3028	1.0285	0.1162	0.0575	0.0106	0.0062	-0.3934	0.9839	0.1183	0.06
H. heurippa	0.0108	0.0085	0.4122	1.3760	0.2262	0.1119	0.0078	0.0061	0.3184	1.5081	0.2515	0.134
H. t. florencia	0.0160	0.0092	-0.1105	1.0305	0.1455	0.0668	0.0113	0.0065	-0.3609	0.9827	0.1479	0.07
H. t. linaresi	0.0143	0.0088	-0.0416	1.0354	0.1637	0.0748	0.0107	0.0065	0.0542	1.0812	0.1778	0.084
