# Supplementary material for: Selective sweeps on novel and introgressed variation shape mimicry loci in a butterfly adaptive radiation
Source: PLoS Biol. 2020 Feb 6;18(2):e3000597. doi: 10.1371/journal.pbio.3000597 (PMC7029882; doi:10.1371/journal.pbio.3000597)
Supplement: S16 Table — (PDF) [file pbio.3000597.s038.pdf]

S16 Table. Per-population and per-scaffold summary statistics estimates and standard deviation for neutral background scaffolds in the *H. melpomene* - clade.

	**Hmel204017:1850700-2350950**						**Hmel206006:294700-795100**					
	pi	**sd**	**Taj*D***	**sd**	***ZnS***	**sd**	***pi***	**sd**	**Taj*D***	**sd**	***ZnS***	**sd**
H. m. aglaope	0.0288	0.0094	-0.2174	0.4229	0.2420	0.0383	0.0252	0.0090	-0.3679	0.4777	0.2441	0.0426
H. m. amaryllis	0.0188	0.0057	-1.3056	0.3914	0.0357	0.0069	0.0192	0.0059	-1.3638	0.4236	0.0383	0.0076
H. m. malleti (Colombia)	0.0217	0.0062	-0.7849	0.4240	0.0602	0.0110	0.0205	0.0068	-0.7276	0.4820	0.0692	0.0146
H. m. malleti (Ecuador)	0.0209	0.0059	-1.1941	0.4079	0.0439	0.0085	0.0207	0.0064	-1.2135	0.4347	0.0477	0.0096
H. m. plesseni	0.0233	0.0071	-0.5418	0.4642	0.1049	0.0188	0.0216	0.0075	-0.4573	0.5241	0.1191	0.0285
H. m. xenoclea	0.0204	0.0063	-0.7699	0.4339	0.1037	0.0175	0.0200	0.0066	-0.8235	0.4870	0.1093	0.0184
H. m. meriana	0.0175	0.0067	0.1863	0.5623	0.1990	0.0864	0.0173	0.0069	0.3481	0.6783	0.2292	0.0597
H. m. melpomene (Colombia)	0.0244	0.0071	-0.2604	0.4714	0.0893	0.0276	0.0220	0.0068	-0.3130	0.5718	0.1073	0.0340
H. m. melpomene (French Guyana)	0.0171	0.0061	-0.3428	0.6010	0.1938	0.1002	0.0173	0.0064	-0.4137	0.6242	0.1909	0.0821
H. m. nanna (North)	0.0176	0.0069	-0.3702	0.5298	0.1586	0.0426	0.0172	0.0067	-0.4706	0.5764	0.1633	0.0478
H. m. nanna (South)	0.0069	0.0050	-0.1420	0.9092	0.2744	0.1337	0.0056	0.0040	-0.3536	0.8500	0.2639	0.1337
H. c. weymeri gustavi	0.0219	0.0075	0.0006	0.5654	0.1395	0.0394	0.0198	0.0068	-0.0610	0.6108	0.1456	0.0408
H. c. weymeri weymeri	0.0210	0.0070	0.1397	0.5918	0.1554	0.0368	0.0198	0.0068	0.0984	0.5305	0.1589	0.0601
H. c. cydnides	0.0229	0.0077	0.0011	0.4572	0.1243	0.0247	0.0207	0.0074	-0.1711	0.5303	0.1273	0.0312
H. melpomene (Panama)	0.0255	0.0078	-0.1762	0.4488	0.1616	0.0336	0.0229	0.0078	-0.1724	0.5308	0.1909	0.0588
H. m. rosina	0.0226	0.0082	0.0907	0.4851	0.1487	0.0449	0.0216	0.0069	-0.0962	0.5565	0.1655	0.0596
H. m. vulcanus	0.0205	0.0078	0.0620	0.5891	0.1597	0.0502	0.0202	0.0075	0.3450	0.6096	0.1939	0.0766
H. m. cythera	0.0237	0.0075	-0.0071	0.4456	0.1432	0.0464	0.0221	0.0072	0.0030	0.5786	0.1686	0.0556
H. m. ecuadorensis	0.0241	0.0074	-0.3972	0.4523	0.1132	0.0210	0.0222	0.0073	-0.4265	0.5582	0.1232	0.0257
H. c. chioneus	0.0207	0.0078	-0.5247	0.4764	0.0950	0.0160	0.0201	0.0067	-0.5836	0.4834	0.1038	0.0240
H. c. zelinde	0.0224	0.0082	-0.2426	0.4285	0.1202	0.0168	0.0204	0.0072	-0.2114	0.4901	0.1304	0.0265
H. pachinus	0.0218	0.0071	-0.1551	0.4779	0.1220	0.0213	0.0198	0.0067	-0.2031	0.5432	0.1399	0.0475
H. t. thelxinoe	0.0190	0.0059	-0.0810	0.5827	0.0722	0.0304	0.0189	0.0062	-0.0693	0.6023	0.0780	0.0236
H. t. contigua	0.0190	0.0060	-0.0331	0.5949	0.1371	0.0373	0.0187	0.0063	-0.0347	0.5802	0.1512	0.0472
H. t. timareta	0.0198	0.0061	-0.0837	0.6080	0.1317	0.0370	0.0192	0.0064	-0.0694	0.5732	0.1443	0.0401
H. t. spp (Colombia)	0.0198	0.0075	0.1243	0.6389	0.2097	0.0636	0.0179	0.0082	0.2100	0.8212	0.2350	0.0824
H. t. spp (Ecuador)	0.0197	0.0064	-0.2249	0.5668	0.0758	0.0227	0.0189	0.0069	-0.2770	0.6722	0.0871	0.0349
H. heurippa	0.0173	0.0076	0.4316	1.0247	0.1600	0.0614	0.0162	0.0078	0.4216	1.2018	0.1799	0.0735
H. t. florencia	0.0205	0.0073	-0.1037	0.6220	0.0985	0.0367	0.0197	0.0069	-0.1325	0.7058	0.1050	0.0362
H. t. linaresi	0.0211	0.0069	-0.0737	0.5686	0.0972	0.0292	0.0202	0.0071	0.1733	0.7088	0.1192	0.0463

	**Hmel208051:635350-1134550**						**Hmel219003:5223750-5722800**					
	pi	**sd**	**Taj*D***	**sd**	***ZnS***	**sd**	***pi***	**sd**	**Taj*D***	**sd**	***ZnS***	**sd**
H. m. aglaope	0.0227	0.0089	-0.3399	0.4762	0.2417	0.0449	0.0231	0.0094	-0.3827	0.5488	0.2602	0.0592
H. m. amaryllis	0.0163	0.0058	-1.3814	0.4101	0.0385	0.0104	0.0191	0.0060	-1.3095	0.4436	0.0486	0.0271
H. m. malleti (Colombia)	0.0184	0.0064	-0.8732	0.4581	0.0655	0.0182	0.0188	0.0068	-0.7975	0.5449	0.0837	0.0362
H. m. malleti (Ecuador)	0.0181	0.0061	-1.2723	0.4189	0.0467	0.0111	0.0190	0.0070	-1.1785	0.5036	0.0593	0.0239
H. m. plesseni	0.0197	0.0074	-0.7738	0.5161	0.1039	0.0215	0.0203	0.0084	-0.5672	0.6013	0.1288	0.0506
H. m. xenoclea	0.0173	0.0064	-0.9570	0.4553	0.1044	0.0206	0.0179	0.0069	-0.9683	0.5208	0.1172	0.0365
H. m. meriana	0.0163	0.0065	-0.2253	0.5842	0.1780	0.0534	0.0169	0.0073	0.1161	0.6771	0.2004	0.0590
H. m. melpomene (Colombia)	0.0204	0.0071	-0.3410	0.4895	0.1032	0.0425	0.0217	0.0075	-0.3318	0.5919	0.1109	0.0416
H. m . melpomene (French Guyana)	0.0162	0.0057	-0.3999	0.5987	0.2321	0.1082	0.0163	0.0062	-0.4373	0.6488	0.2157	0.1121
H. m. nanna (North)	0.0151	0.0070	-0.4006	0.6118	0.1723	0.0607	0.0154	0.0073	-0.5508	0.7090	0.1858	0.0762
H. m. nanna (South)	0.0048	0.0041	-0.2595	0.8836	0.2540	0.1271	0.0059	0.0050	-0.2452	0.9914	0.3013	0.1655
H. c. weymeri gustavi	0.0166	0.0070	-0.0984	0.6108	0.1465	0.0424	0.0184	0.0077	0.1495	0.6995	0.1687	0.0639
H. c. weymeri weymeri	0.0168	0.0069	0.1226	0.6129	0.1543	0.0414	0.0181	0.0079	0.2612	0.6731	0.1737	0.0568
H. c. cydnides	0.0174	0.0072	-0.0517	0.6227	0.1410	0.0431	0.0193	0.0076	0.1053	0.6693	0.1547	0.0473
H. melpomene (Panama)	0.0210	0.0073	-0.1852	0.4841	0.1739	0.0517	0.0218	0.0082	-0.1588	0.6262	0.2261	0.0917
H. m. rosina	0.0183	0.0070	-0.0033	0.5666	0.1620	0.0637	0.0210	0.0083	0.1511	0.7287	0.2184	0.1111
H. m. vulcanus	0.0169	0.0065	0.1931	0.5944	0.1890	0.0786	0.0194	0.0089	0.4287	0.8013	0.2448	0.1218
H. m. cythera	0.0198	0.0068	-0.2486	0.5880	0.1643	0.0685	0.0217	0.0081	-0.0271	0.6675	0.1969	0.0972

	Hmel208051:635350-1134550						Hmel219003:5223750-5722800					
	pi	**sd**	**Taj*D***	**sd**	***ZnS***	**sd**	***pi***	**sd**	**Taj*D***	**sd**	***ZnS***	**sd**
H. m. ecuadorensis	0.0195	0.0071	-0.5250	0.4918	0.1243	0.0339	0.0212	0.0080	-0.5652	0.6263	0.1460	0.0619
H. c. chioneus	0.0166	0.0068	-0.7819	0.4989	0.1006	0.0221	0.0198	0.0074	-0.4428	0.5327	0.1134	0.0409
H. c. zelinde	0.0174	0.0069	-0.4935	0.4943	0.1399	0.0461	0.0221	0.0069	-0.2626	0.5010	0.1364	0.0424
H. pachinus	0.0174	0.0067	-0.1542	0.5515	0.1371	0.0441	0.0191	0.0074	-0.0915	0.6349	0.1598	0.0531
H. t. thelxinoe	0.0157	0.0062	0.0242	0.6236	0.0901	0.0435	0.0123	0.0065	0.2051	1.2645	0.1854	0.0922
H. t. contigua	0.0158	0.0068	0.1109	0.6172	0.1593	0.0523	0.0138	0.0067	0.0090	0.9632	0.1988	0.0805
H. t. timareta	0.0163	0.0069	-0.0628	0.5813	0.1427	0.0456	0.0143	0.0067	0.0987	0.9383	0.2062	0.0838
H. t. spp (Colombia)	0.0160	0.0078	0.0980	0.6522	0.2214	0.0762	0.0134	0.0085	0.0332	1.1453	0.2876	0.1462
H. t. spp (Ecuador)	0.0162	0.0070	-0.2520	0.6931	0.0881	0.0391	0.0151	0.0069	-0.1595	0.9122	0.1301	0.0652
H. heurippa	0.0139	0.0070	0.3350	1.1354	0.1755	0.0873	0.0126	0.0071	0.6984	1.3255	0.2476	0.1233
H. t. florencia	0.0166	0.0071	-0.1427	0.7788	0.1100	0.0466	0.0150	0.0074	-0.1668	1.0112	0.1791	0.1091
H. t. linaresi	0.0174	0.0068	0.0646	0.6984	0.1149	0.0597	0.0160	0.0077	0.2760	1.1671	0.1848	0.1085
