# Supplementary material for: Selective sweeps on novel and introgressed variation shape mimicry loci in a butterfly adaptive radiation
Source: PLoS Biol. 2020 Feb 6;18(2):e3000597. doi: 10.1371/journal.pbio.3000597 (PMC7029882; doi:10.1371/journal.pbio.3000597)
Supplement: S17 Table — (PDF) [file pbio.3000597.s039.pdf]

S17 Table. Per-population and per-scaffold summary statistics estimates and standard deviation for colour pattern scaffolds in the *H. erato* - clade.

	**Herato1001:3499501-5500000**						**Herato1505:999501-3000000**					
	pi	**sd**	**Taj*D***	**sd**	***ZnS***	**sd**	***pi***	**sd**	**Taj*D***	**sd**	***ZnS***	**sd**
H. e. demophoon	0.0256	0.0091	-0.8829	0.4917	0.1287	0.0388	0.0227	0.0097	-0.4757	0.6853	0.1727	0.0737
H. e. notabilis	0.0238	0.0102	-0.7391	0.6014	0.1464	0.0832	0.0232	0.0113	-0.5472	0.6911	0.1665	0.1001
H. e. etylus	0.0250	0.0107	-0.4591	0.5793	0.2477	0.0943	0.0261	0.0131	-0.4260	0.6069	0.2467	0.0982
H. e. lativitta	0.0273	0.0104	-0.4165	0.5248	0.2287	0.0625	0.0271	0.0129	-0.3362	0.5693	0.2366	0.0862
H. e. cyrbia	0.0142	0.0073	-0.7897	0.6945	0.1457	0.0744	0.0147	0.0114	-0.5188	0.8100	0.1880	0.1058
H. e. venus	0.0163	0.0085	-0.0668	0.7000	0.2770	0.1095	0.0202	0.0109	-0.3693	0.7096	0.2630	0.1029
H. e. emma	0.0251	0.0099	-1.1229	0.5013	0.1152	0.0533	0.0251	0.0105	-0.9082	0.5293	0.1253	0.0678
H. e. favorinus	0.0225	0.0101	-0.5646	0.5999	0.1360	0.0593	0.0216	0.0108	-0.5740	0.6928	0.1631	0.0977
H. e. erato	0.0263	0.0108	-0.6439	0.5304	0.1879	0.0765	0.0267	0.0111	-0.4640	0.5810	0.1970	0.0795
H. e. hydara (French Guyana)	0.0248	0.0107	-0.2940	0.6585	0.2509	0.0875	0.0273	0.0114	-0.3753	0.5994	0.2332	0.0854
H. e. hydara (Panama)	0.0270	0.0099	-0.5016	0.5019	0.2285	0.0677	0.0260	0.0102	-0.3721	0.5912	0.2608	0.0840
H. e.amalfreda	0.0275	0.0109	-0.5331	0.5393	0.2186	0.0764	0.0273	0.0113	-0.3674	0.5542	0.2381	0.0896
H. e. chestertonii	0.0076	0.0073	-0.0936	1.1263	0.2759	0.1754	0.0100	0.0102	-0.1283	1.2337	0.3371	0.1983
H. himera	0.0103	0.0086	0.2031	1.1418	0.2748	0.1625	0.0107	0.0083	-0.9693	1.1588	0.3467	0.1620

	**Herato1801:899501-1800000**					
	pi	**sd**	**Taj*D***	**sd**	***ZnS***	**sd**
H. e. demophoon	0.0253	0.0090	-0.7641	0.5026	0.1366	0.0444
H. e. notabilis	0.0145	0.0097	-0.3351	0.9988	0.2533	0.1466
H. e. etylus	0.0243	0.0111	-0.6403	0.5271	0.2130	0.0761
H. e. lativitta	0.0241	0.0110	-0.5093	0.5344	0.2130	0.0659
H. e. cyrbia	0.0132	0.0073	-0.7480	0.7211	0.1550	0.0914
H. e. venus	0.0152	0.0088	-0.0996	0.7425	0.2507	0.0951
H. e. emma	0.0226	0.0099	-1.0467	0.4951	0.1099	0.0357
H. e. favorinus	0.0196	0.0091	-0.3753	0.6853	0.1474	0.0667
H. e. erato	0.0218	0.0107	-0.2190	0.7191	0.2136	0.0859
H. e. hydara (French Guyana)	0.0195	0.0104	0.0509	0.8806	0.3229	0.1388
H. e. hydara (Panama)	0.0260	0.0093	-0.4152	0.5271	0.2338	0.0782
H. e.amalfreda	0.0229	0.0110	-0.2654	0.6592	0.2417	0.0905
H. e. chestertonii	0.0045	0.0055	-0.2863	1.2052	0.2630	0.2038
H. himera	0.0084	0.0073	0.2283	1.1827	0.2784	0.1556
