# Supplementary material for: Selective sweeps on novel and introgressed variation shape mimicry loci in a butterfly adaptive radiation
Source: PLoS Biol. 2020 Feb 6;18(2):e3000597. doi: 10.1371/journal.pbio.3000597 (PMC7029882; doi:10.1371/journal.pbio.3000597)
Supplement: S18 Table — (PDF) [file pbio.3000597.s040.pdf]

S18 Table. Per-population and per-scaffold summary statistics estimates and standard deviation for neutral background scaffolds in the *H. erato* - clade.

	Herato0411:4001001-5000000						Herato0601:800001-1800000					
	pi	**sd**	**Taj*D***	**sd**	***ZnS***	**sd**	***pi***	**sd**	**Taj*D***	**sd**	***ZnS***	**sd**
H. e. demophoon	0.0274	0.0100	-1.0591	0.3995	0.1146	0.0345	0.0286	0.0091	-0.9415	0.3905	0.1190	0.0333
H. e. notabilis	0.0255	0.0095	-1.2358	0.4158	0.1141	0.0285	0.0277	0.0091	-1.0943	0.3614	0.1164	0.0317
H. e. etylus	0.0278	0.0107	-0.7390	0.4449	0.2215	0.0727	0.0292	0.0098	-0.6419	0.4279	0.2145	0.0569
H. e. lativitta	0.0290	0.0106	-0.5249	0.4405	0.2211	0.0550	0.0305	0.0097	-0.5785	0.3992	0.2069	0.0486
H. e. cyrbia	0.0193	0.0085	-0.8725	0.5322	0.1353	0.0623	0.0166	0.0079	-0.8030	0.6438	0.1511	0.0773
H. e. venus	0.0248	0.0098	-0.0861	0.5414	0.2564	0.0810	0.0223	0.0092	-0.4008	0.6021	0.2406	0.0736
H. e. emma	0.0255	0.0098	-1.2534	0.3681	0.1097	0.0453	0.0280	0.0090	-1.3739	0.3087	0.0967	0.0228
H. e. favorinus	0.0260	0.0097	-1.1568	0.3840	0.1073	0.0355	0.0283	0.0090	-1.1963	0.3728	0.1009	0.0291
H. e. erato	0.0280	0.0104	-0.7805	0.4236	0.1742	0.0512	0.0298	0.0096	-0.7880	0.3975	0.1704	0.0466
H. e. hydara (French Guyana)	0.0284	0.0107	-0.6626	0.4355	0.2111	0.0708	0.0298	0.0100	-0.5404	0.4391	0.2156	0.0572
H. e. hydara (Panama)	0.0294	0.0163	-0.6805	0.4194	0.2138	0.0667	0.0306	0.0098	-0.6037	0.4038	0.2083	0.0421
H. e.amalfreda	0.0289	0.0104	-0.6014	0.4160	0.2090	0.0513	0.0309	0.0100	-0.5056	0.3837	0.2084	0.0474
H. e. chestertonii	0.0119	0.0085	-0.0675	1.1368	0.3153	0.1810	0.0083	0.0079	0.0561	1.2239	0.3144	0.1782
H. himera	0.0155	0.0092	0.2659	1.0176	0.2583	0.1240	0.0141	0.0091	0.1344	1.1802	0.2626	0.1255
	**Herato0821:1999501-3000000**						**Herato1901:2462001-3460500**					
	pi	**sd**	**Taj*D***	**sd**	***ZnS***	**sd**	***pi***	**sd**	**Taj*D***	**sd**	***ZnS***	**sd**
H. e. demophoon	0.0270	0.0102	-1.0282	0.3980	0.1143	0.0338	0.0274	0.0102	-0.9277	0.4918	0.1326	0.0575
H. e. notabilis	0.0257	0.0100	-1.1413	0.3811	0.1139	0.0398	0.0280	0.0103	-1.0824	0.5063	0.1247	0.0581
H. e. etylus	0.0279	0.0123	-0.7041	0.4123	0.2084	0.0571	0.0300	0.0107	-0.7132	0.4665	0.2187	0.0644
H. e. lativitta	0.0280	0.0109	-0.4215	0.4326	0.2253	0.0457	0.0300	0.0108	-0.4025	0.4675	0.2308	0.0763
H. e. cyrbia	0.0181	0.0085	-0.7960	0.5866	0.1442	0.0635	0.0153	0.0078	-0.7189	0.7000	0.1503	0.0648
H. e. venus	0.0227	0.0097	0.0144	0.6677	0.2594	0.0815	0.0193	0.0092	-0.2978	0.7211	0.2498	0.0899
H. e. emma	0.0257	0.0099	-1.2375	0.3514	0.1009	0.0321	0.0280	0.0104	-1.2004	0.4708	0.1092	0.0512
H. e. favorinus	0.0260	0.0102	-1.1275	0.3829	0.1040	0.0321	0.0279	0.0097	-0.9503	0.5135	0.1198	0.0566
H. e. erato	0.0271	0.0107	-0.7108	0.4316	0.1740	0.0418	0.0296	0.0159	-0.7486	0.4581	0.1828	0.0572
H. e. hydara (French Guyana)	0.0280	0.0109	-0.5844	0.4139	0.2072	0.0512	0.0301	0.0116	-0.5983	0.4993	0.2211	0.0714
H. e. hydara (Panama)	0.0291	0.0108	-0.6519	0.4198	0.2087	0.0518	0.0291	0.0117	-0.6019	0.5096	0.2333	0.0839
H. e.amalfreda	0.0288	0.0110	-0.5988	0.4195	0.2049	0.0506	0.0307	0.0112	-0.6207	0.4649	0.2102	0.0581
H. e. chestertonii	0.0088	0.0079	-0.2771	1.1824	0.2893	0.1614	0.0117	0.0084	0.1160	1.2878	0.3420	0.1749
H. himera	0.0153	0.0091	0.1630	1.0580	0.2532	0.1061	0.0115	0.0089	0.1519	1.1610	0.3002	0.1657
